# Norcorroles as antiaromatic π-electronic systems that form dimension-controlled assemblies†

**DOI:** 10.1039/d4sc01633e

**Published:** 2024-04-16

**Authors:** Soh Ishikawa, Kazuhisa Yamasumi, Shinya Sugiura, Shunsuke Sato, Go Watanabe, Yun Hee Koo, Shu Seki, Yuya Bando, Yohei Haketa, Hiroshi Shinokubo, Hiromitsu Maeda

**Affiliations:** a Department of Applied Chemistry, College of Life Sciences, Ritsumeikan University Kusatsu 525-8577 Japan maedahir@ph.ritsumei.ac.jp; b Department of Physics, School of Science, Kitasato University Sagamihara 252-0373 Japan; c Department of Data Science, School of Frontier Engineering, Kitasato University Sagamihara 252-0373 Japan; d Department of Molecular Engineering, Graduate School of Engineering, Kyoto University Kyoto 615-8510 Japan; e Department of Molecular and Macromolecular Chemistry, Graduate School of Engineering, Nagoya University Nagoya 464-8603 Japan

## Abstract

Norcorrole derivatives with 3,4,5-trialkoxyphenyl moieties at the *meso* positions were synthesized to form various stacking assemblies in single crystals and thermotropic liquid crystals (LCs) depending on aliphatic chain lengths. Triple-decker stacking structures were formed *via* the interactions between the antiaromatic systems formed for the butoxy and dodecyloxy derivatives in the single-crystal and LC states, respectively. In particular, the LC state exhibited discotic columnar structures comprising triple deckers to exhibit high electric conductivity, as supported by molecular dynamics simulations.

## Abstract 2

Norcorroles with 3,4,5-trialkoxyphenyl moieties were synthesized to form thermotropic liquid crystals (LCs), in which triple-decker stacking structures were formed. The LC state exhibited discotic columnar structures to exhibit high electric conductivity.

## Graphical abstract

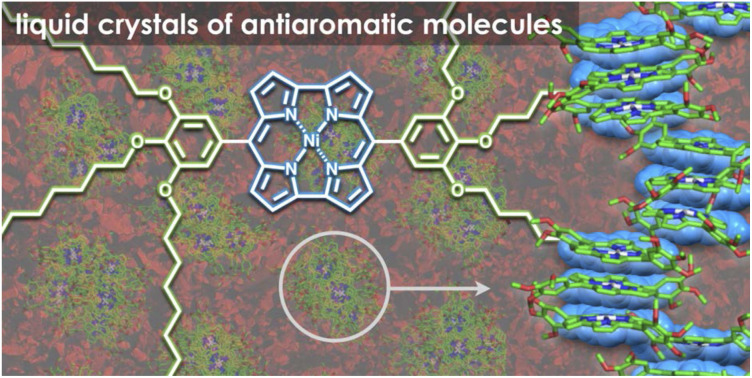

## Introduction

The electronic and electrooptical properties emerging from assemblies of π-electronic molecules are controlled by the arrangement of the building units.^1^ π-Electronic systems form stacking structures *via* π–π interactions, which are mainly derived from dispersion forces.^2^ The additional interactions that occur due to peripheral substituents, such as the van der Waals interactions among aliphatic chains, are effective in modulating the stacking structures and resulting properties for applications in molecular electronics.^3^ In particular, antiaromatic systems with peripheral aliphatic chains are potentially promising candidates for electric conducting materials due to the excellent redox properties derived from the small HOMO–LUMO gaps. However, only a few antiaromatic systems have been considered as the π-electronic building units of dimension-controlled assemblies with stacking structures such as liquid crystals.^4^ Moreover, their assembling behaviours were similar to those of ordinary aromatic systems because of the small antiaromaticity of the building units.

**Fig. 1:**
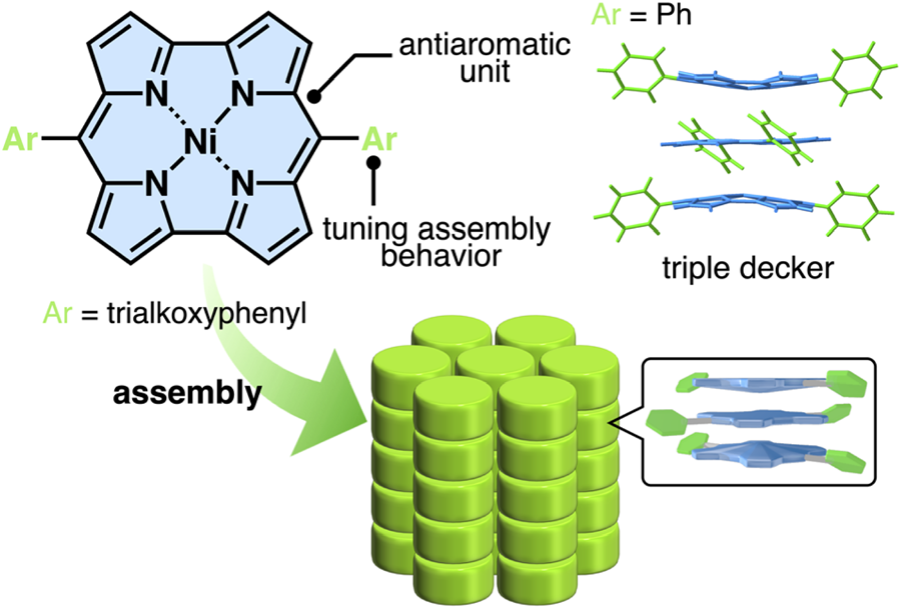
Dimension-controlled assemblies of Ni^II^ norcorroles based on the interactions between antiaromatic systems.

Norcorroles, porphyrin analogues whose two *meso* carbons are missing (Fig. 1 top), are promising antiaromatic π-electronic systems for forming stacking structures.^5,6^ The antiaromatic property of norcorroles is derived from the 16 π-electrons in the conjugated circuit, producing attractive interactions between the antiaromatic systems through stacking, inducing stacked-ring aromaticity, for which face-to-face stacking is crucial.^6*b*,*d*,*g*,*j*^ A triple-decker structure, stabilized by the stacked-ring aromaticity, was formed in the crystal structure of *meso*-phenyl-substituted Ni^II^ norcorrole, which contained outer units with concave geometries (Fig. 1 right).^6*b*^ The assembling behaviour was modulated by the introduction of amide-substituted side chains, resulting in the formation of 1D supramolecular polymers with higher charge-carrier mobility than the aromatic counterpart.^6*h*^ The norcorrole exhibited no obvious near infrared absorption characteristic of the face-to-face stacking in the supramolecular polymer, suggesting the deviation from face-to-face stacked arrangement. Therefore, the norcorrole assemblies through face-to-face stacking in dimension-controlled assemblies have not been achieved to date. The deviation from face-to-face stacked arrangement would be derived from significant hydrogen-bonding interactions between the amide units of side chains. Introduction of other side interacting moieties that have less directionality would enhance the stacking between norcorrole units. This report shows the assembly of norcorroles *via* the simple introduction of aliphatic chains, which induce van der Waals interactions, to the *meso*-aryl moieties (Fig. 1 bottom). The synergetic effects of the stacked norcorroles and introduced aliphatic chains were observed in a liquid crystal columnar assembly,^7^ which exhibited nonconventional nanoscale phase-segregation as a norcorrole-based superstructure, along with enhanced electric conductivity.

## Results and discussion

### Synthesis and characterization

**Fig. 2:**
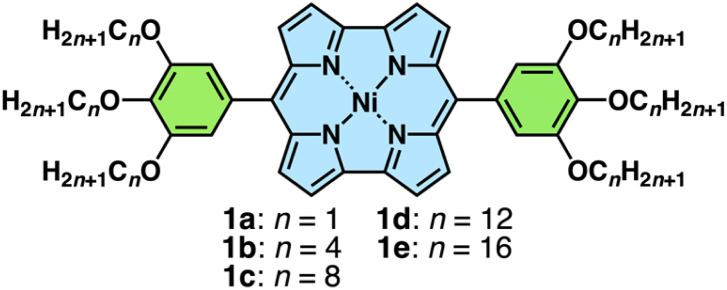
Ni^II^*meso*-(3,4,5-trialkoxyphenyl)norcorroles 1a–e.

Norcorrole Ni^II^ complexes 1a–e, bearing alkoxy units with different chain lengths at the *meso*-aryl units, were synthesized from the corresponding dipyrromethanes according to procedures found in the literature (Fig. 2 and S1†).^6*b*^ Ni^II^, as a diamagnetic d^8^ state, was selected as a metal centre because it allowed the facile production of norcorrole frameworks. The synthesized compounds were characterized using ^1^H and ^13^C NMR and MALDI/ESI-TOF-MS. The ^1^H NMR of 1a–e in CDCl_3_ showed antiaromatic properties as suggested by the significant upfield shifts of the β-CH signals (1.95–2.52 ppm) in contrast to those of aromatic porphyrins (9.04–9.18 ppm) (Fig. S9–13 and 17–19†). Their UV/vis absorption spectra in CH_2_Cl_2_ exhibited absorption bands at 427, 497 and 524 nm for 1a and 426 and 528 nm for 1b–e, with the alkoxy chains of four or more CH_2_ units (Fig. S20†). Norcorroles 1a–e exhibited a weak absorption band at up to ∼900 nm, which was characteristic of antiaromatic compounds.^6^ TD-DFT calculation for the optimized structure of 1a at the B3LYP/6-31G(d,p)-SDD level suggested that the absorption at 519 nm could be assigned to a CT transition band from trialkoxyphenyl units to the norcorrole core part (Fig. S42†).^8^

Upon cooling, the β-CH ^1^H NMR signals of 1d at 2.51 and 2.07 ppm at 20 °C in CD_2_Cl_2_ (1 mM) were shifted downfield to 3.22 and 2.80 ppm, respectively, at −30 °C, suggesting that the stacked-ring aromaticity was induced by stacking of the antiaromatic core (Fig. S77†).^6*b*^ This result was consistent with the larger values in harmonic oscillator model of aromaticity (HOMA: 0.53, estimated based on the internal ring comprising 14 atoms) and more negative nucleus-independent chemical shift (NICS) estimated for an optimized structure of the double decker of 1a compared to those of the monomeric 1a (HOMA: 0.36) (Fig. S38 and 46†). The diatropic ring current indicated with clockwise arrows in the anisotropy of the induced current density (ACID) plot of the stacked 1a dimer also supported the stacked-ring aromaticity (Fig. S48†).^8–11^ The UV/vis absorption band of 1d at ∼850 nm, which is characteristic of closely stacked norcorroles,^6*b*,*d*,*g*,*i*,*j*^ in CH_2_Cl_2_ (1 mM) appeared upon cooling to −40 °C, with drastic changes at short wavelengths, suggesting the aggregation (Fig. S74†). The absorption band in the long wavelength region theoretically supported the formation of an oligomeric aggregate (Fig. S43 and 44†).^8^ In contrast, 1e rapidly formed precipitates at ∼0 °C. The cyclic voltammogram (CV) of 1b in CH_2_Cl_2_ exhibited three reversible oxidation (0.16, 0.40 and 1.07 V *vs.* Ag/Ag^+^) and two reversible reduction waves (−0.64 and −1.32 V) in the −2.0 to +1.3 V region (Fig. S21†). The intensities of the first two oxidation waves were approximately half of those of the other redox waves, which was similar to the previously reported results for *ortho*-unsubstituted *meso*-arylnorcorroles, suggesting the formation of a mixed-valence dimer in the first oxidation process.^6*g*^

### Solid-state assemblies

**Fig. 3:**
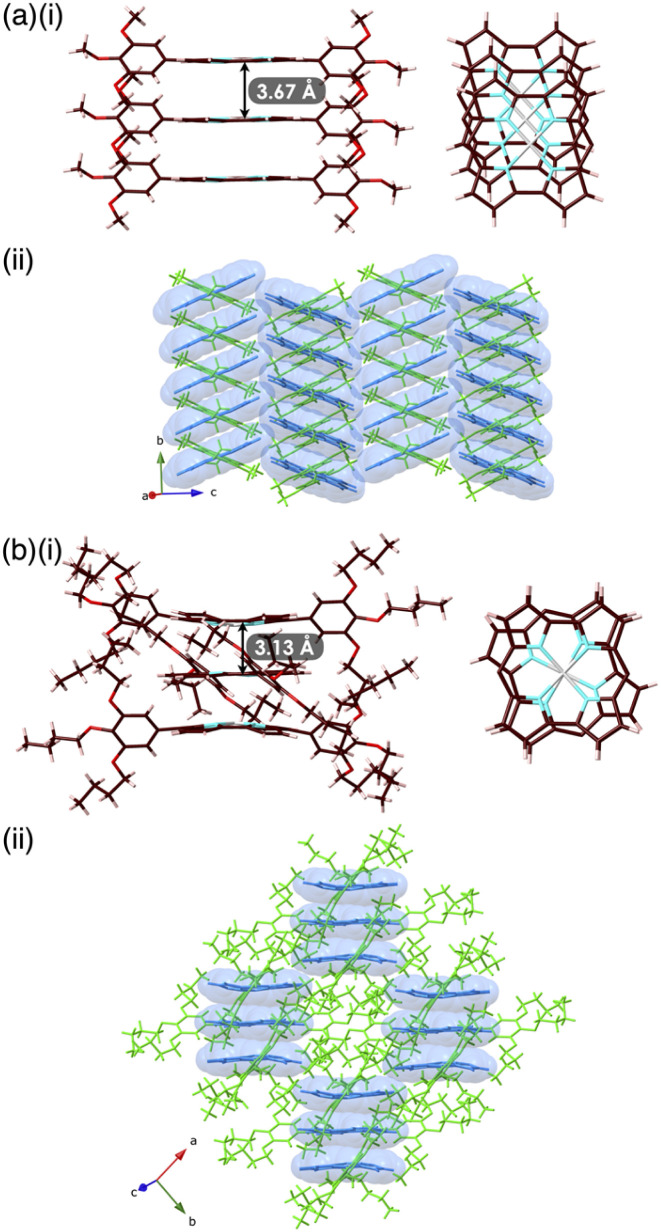
(a) Single-crystal X-ray structures of (a) 1a and (b) 1b as (i) slip-stacked trimer and triple decker, respectively, showing the distances between two mean planes (side and core top views) and (ii) representative packing structures (blue: core unit, green: aryl unit). Atom colour codes in (i): brown, pink, cyan, red and grey refer to carbon, hydrogen, nitrogen, oxygen and nickel, respectively.

**Fig. 4:**
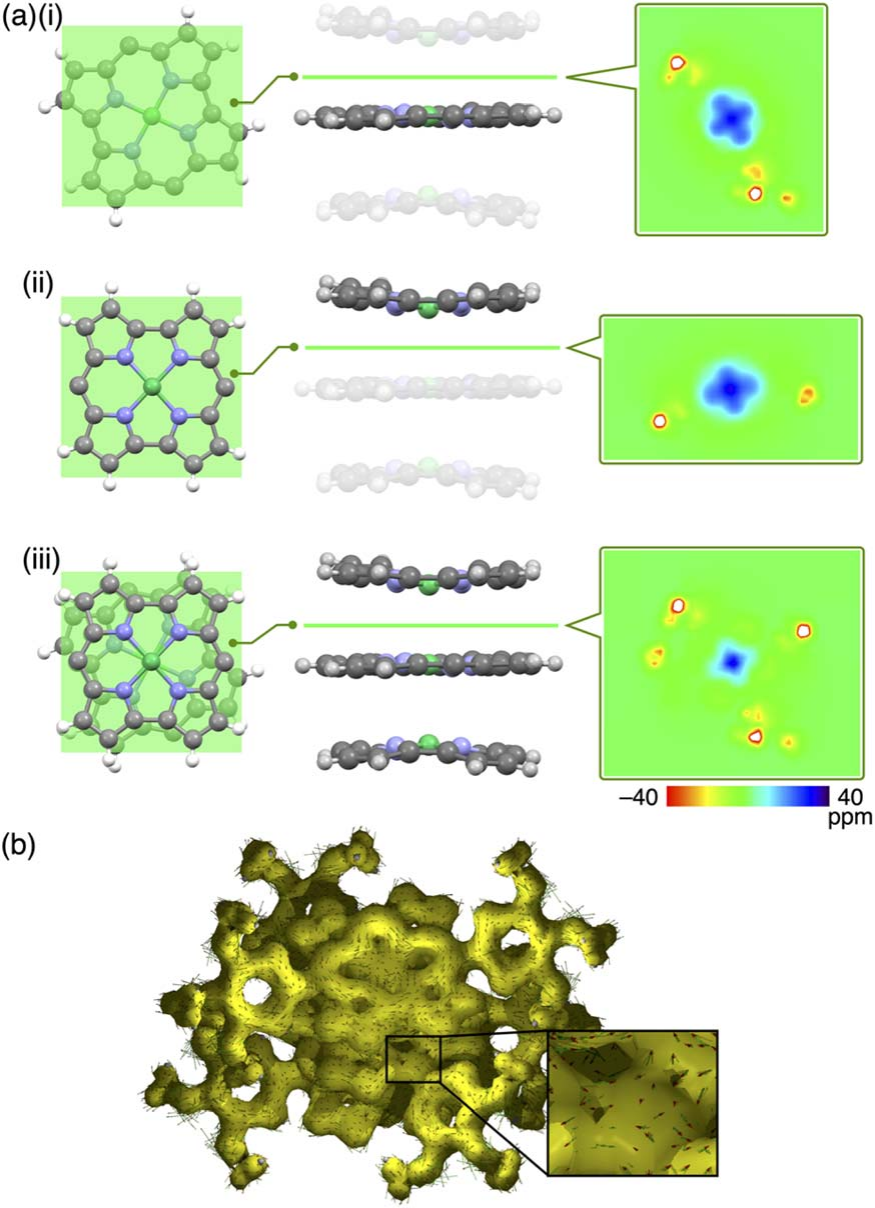
(a) Arrangements of the norcorrole units in the crystal structure of 1b as top (left) and side (centre) views and 2D NICS plots (right, ppm), estimated for the planes (left) and lines (centre), as (i) monomer (centre), (ii) monomer (top) and (iii) stacked trimer at BHLYP/6-31G(d)-SDD level and (b) ACID plot of 1b trimer in the crystal structure. The translucent parts in the side views in (a) (i, ii) were not included in the calculations. *Meso*-aryl groups in (a) were omitted for clarity, and the butoxy groups were replaced with methoxy groups for facile calculations.

Single crystals of 1a, b were prepared from C_6_H_5_Cl/MeOH and used for X-ray analysis.^12^ In the solid state, 1a had a square planar Ni^II^ coordination geometry with the N–Ni distances of 1.78–1.79 Å and a deviation of 0.05 Å for the mean plane (core 23 atoms), to which the *meso*-aryl rings were tilted at 45.3° (Fig. 3a(i)). In the packing diagram, stacking along the *b* axis was observed with an interplane distance of 3.67 Å and the *meso*-aryl units also showed π–π-stacking with an interplane distance of 3.56 Å. The Ni⋯Ni distance and angle of the 1a plane in the columnar direction were 3.93 Å and 69.0°, respectively, with a slip distance of 1.41 Å. The long axis of the 1a crystal corresponded to the *b* axis in the unit cell, along which the norcorroles were stacked (Fig. 3a(ii) and S29†). On the other hand, X-ray analysis of 1b showed a triple-decker stacking structure (Fig. 3b(i)) similar to that of *meso*-phenyl-substituted norcorrole.^6*b*^ In 1b, the centre norcorrole was relatively planar with a deviation of 0.05 Å for the mean plane and staggered by 72.6° relative to the outer stacked molecules. The outer norcorrole units adopted a bowl-shaped conformation and a mean-plane deviation of 0.16 Å. The bowl depth defined by a Ni···mean-plane (eight β-C atoms) distance was 0.49 Å. The Ni⋯Ni distance and that between the two mean planes (four N atoms) of the centre and outer norcorroles were 3.00 and 3.13 Å, respectively. The close stacking of 1b was observed with a larger overlap between the core π-planes compared to 1a. The *meso*-aryl rings of the centre and outer 1b were tilted at 41.9° and 48.9°/52.4°, respectively, to the mean planes (23 atoms). In the crystal of 1b, the long axis corresponded to the *a* axis, which was the direction of the columnar assemblies of 1b_3_ with a tilt (Fig. 3b(ii) and S31†). The HOMA values of 1b (top and centre of triple decker: 0.46 and 0.53, respectively) were larger than that of 1a (0.35), indicating that the triple decker of 1b exhibited the stacked-ring aromaticity in contrast to slip-stacked 1a (Fig. S32†). NICS^9,11^ values in the norcorrole macrocycles of the crystal structure of 1a (slip-stacked trimer 1a_3_) at BHLYP/6-31G(d)-SDD level^8^ were comparable to that of monomeric 1a, whereas that of the crystal structure of 1b (triple decker 1b_3_, whose butyl groups were replaced with methyl groups) were significantly lower than those of monomeric 1a (Fig. 4a, b, S46 and 47†). In addition, arrows indicating the current density in the ACID plot of 1a_3_ were directed counterclockwise similar to monomeric 1a, while those on the central norcorrole unit of 1b_3_ were partially directed clockwise (Fig. 4b and S48†).^10^ These results supported the contribution of stacked ring aromaticity of 1b_3_.

### Columnar liquid crystals based on triple deckers

**Fig. 5:**
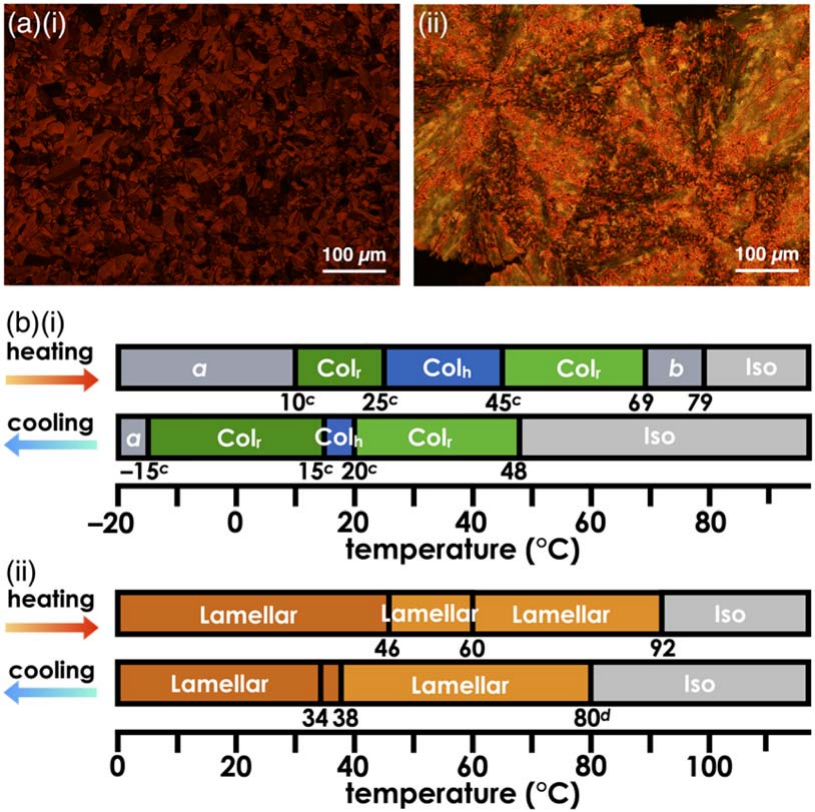
(a) POM textures of (i) 1d at 40 °C and (ii) 1e at 80 °C upon cooling and (b) schematic diagrams of the phase transition behaviours of (i) 1d and (ii) 1e. Phase *a* showed no obvious peaks ascribable to the less ordered structure, whereas phase *b* exhibited a complicated diffraction pattern derived from the unidentified structure. Transition temperatures were determined by DSC without labels and also by XRD and POM labelled with *c* and *d*, respectively. Scan rates in DSC were 5 °C min^−1^ except for 1d at 2 °C min^−1^, which provided the transition temperatures closer to those determined by XRD.

**Fig. 6:**
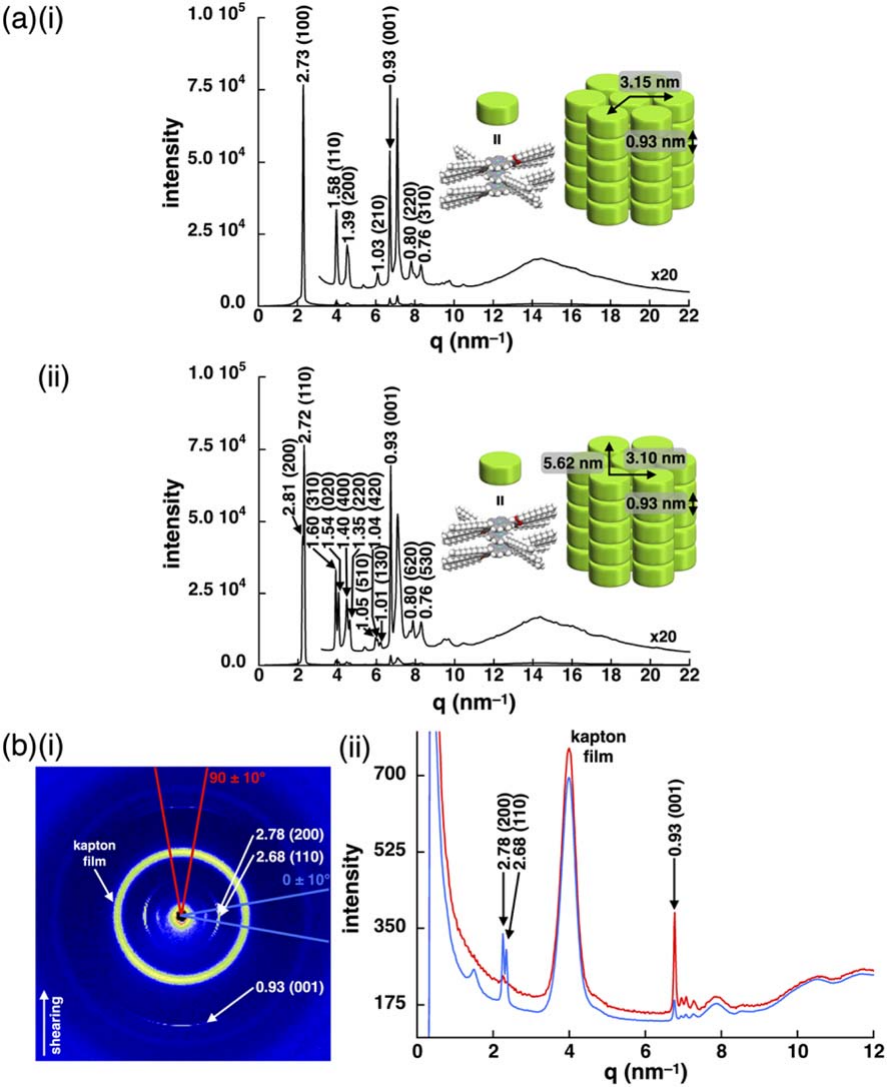
(a) XRD patterns of 1d at (i) 30 °C and (ii) 50 °C upon heating and possible assembled models and (b) XRD patterns of 1d sheared between Kapton (polyimide) films at approximately 85 °C and cooled to r.t., including (i) 2D XRD diffraction pattern and (ii) a combined diagram showing meridional (sheared) (red) and equatorial (blue) directions. An unassigned peak close to 0.93 nm may be derived from the side aryl moieties.

Introduction of aliphatic chains to norcorrole provided mesophases (Fig. 5). Mosaic textures of polarized optical microscopy (POM) were observed for 1d upon cooling from isotropic liquid state (Fig. 5a(i) and S51†). The mesophases formed in the dodecyloxy-substituted 1d had transition temperatures at 10/25/45/69/79 °C (heating) and 48/20/15/–15 °C (cooling) as revealed by differential scanning calorimetry (DSC) and synchrotron X-ray diffraction (XRD), which further revealed the assembled structures in the mesophases (Fig. 6, S49, 57–59 and Table S4†). A schematic diagram of the phase transition behaviour is shown in Fig. 5b(i). In particular, the diffraction peaks were intensified upon heating, suggesting the formation of a highly ordered columnar phase (Fig. S57p–u†). The mesophase at 30 °C upon heating showed a diffraction pattern ascribable to the hexagonal columnar (Col_h_) phase with the parameters *a* = 3.15 nm, *c* = 0.93 nm and *Z* = 3 (*ρ* = 0.99) (Fig. 6a(i)), and that at 50 °C upon heating was the rectangular columnar (Col_r_) phase with the parameters *a* = 5.62 nm, *b* = 3.10 nm, *c* = 0.93 nm and *Z* = 6 (*ρ* = 0.99) (Fig. 6a(ii)), which were observed in two separated temperature ranges. The assignment of 0.93 nm as the stacking distance was confirmed by the increased intensity of the diffraction in the shearing direction, in contrast to the augmentation of other diffractions derived from the rectangular pattern in the horizontal direction (Fig. 6b). The value of 0.93 nm, which was included in both phases, could be ascribed to the repeating distance of the stacking trimeric units, as also seen in the crystal structure of 1b (Fig. 3b). In addition, the film-state 1d in the Col_r_ phase at the higher temperature exhibited an absorption band at ∼1130 nm (Fig. S75a†), which was red-shifted compared to those of previously reported stacked dimers and aggregates (*λ*_max_ = 800–900 nm).^6*b*,*d*,*g*,*i*,*j*^ The significantly red-shifted absorption for the 1d film compared to the norcorrole double deckers was consistent with the red-shifted absorption suggested by TD-DFT calculation (Fig. S43 and 44†), supporting the formation of the stacked trimer. In the higher temperature region at 69–79 °C upon heating, high fluidity was observed in the phase, which showed complicated diffractions (Fig. S57w†).

**Fig. 7:**
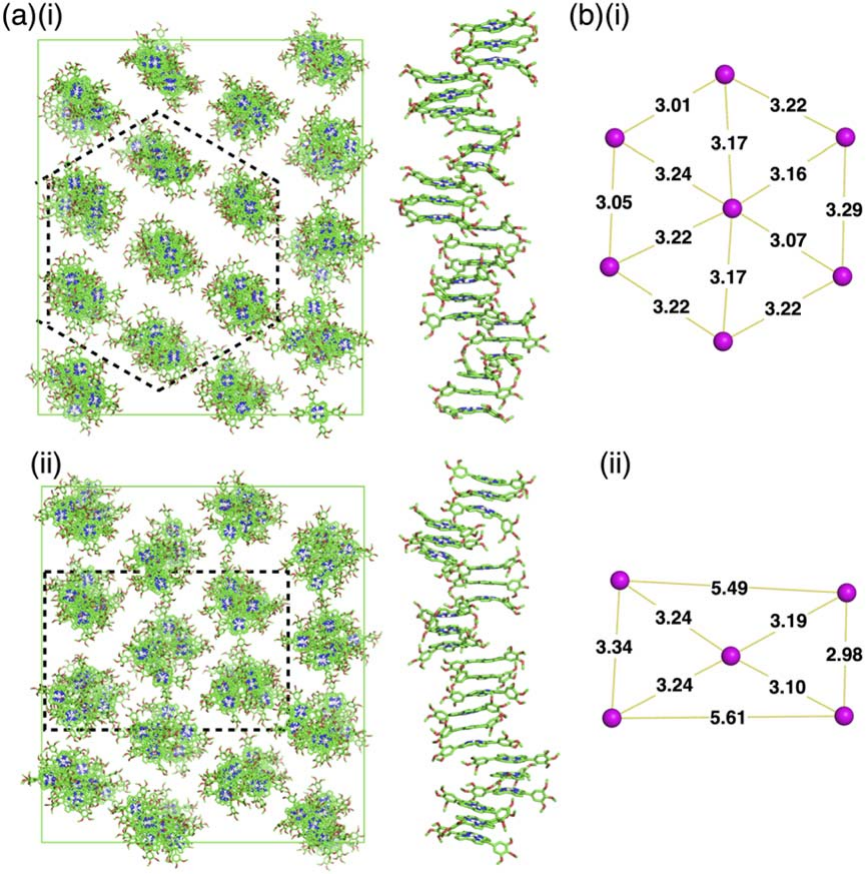
(a) Snapshots of MD simulation results for 1d and (b) arrangement of the columns with the distances (nm) (the locations are indicated with dashed lines in (a)) after 100 ns of the equilibration run (i) at 30 °C and (ii) 50 °C. The alkyl chains and hydrogens are omitted for clarity.

The packing structures of 1d in the Col_h_ and Col_r_ phases at 30 and 50 °C, respectively, were clearly demonstrated in all-atom molecular dynamics (MD) simulations after 100 ns of the equilibration run (Fig. 7). The structures obtained by MD simulation were consistent with those determined by XRD analysis. The concave face of outer norcorrole units in 1d_3_ were unsuitable for stacking with another triple decker.^13^ Thus, the triple deckers were not arranged in a straight line in the columnar structures (Fig. 7a right). The assembly mode was distinct from that for conventional discotic columnar structures, where π-electronic parts and aliphatic chains contribute to a 1D ordered assembly, producing more efficient intermolecular interactions and high entropy effects that exhibit fluidity, respectively. In 1d, the proximal locations of the norcorrole units and dodecyl chains were required to convert from the Col_h_ to Col_r_ phase (Fig. 7a and b (left)). In this study, the concave geometries of the norcorrole triple decker resulted in nonconventional assembled structures in combination with aliphatic chains.

### Effects of substituents and frameworks on assembled structures

The lengths of the aliphatic chains were essential factors in controlling the assembled structures. Hexadecyloxy-substituted 1e exhibited the formation of mesophases with transition temperatures of 46/60/92 °C (heating) and 85/38/34 °C (cooling), as revealed by DSC and POM (Fig. 5a, b(ii), S49 and 52†). The synchrotron XRD results showed the formation of lamellar phases with repeating distances of 5.32–5.44 nm, which were consistent with the molecular lengths (Fig. S60 and 61†). The diffractions at ∼0.40 nm instead of that at 0.93 nm were ascribed to the stacking distances of 1e, suggesting that 1e did not form triple deckers in the bulk state in contrast to 1d.^14^ Compared to norcorroles 1d, e with long aliphatic chains, 1c had a higher melting point at 126 °C, at which it was decomposed upon the conversion to an isotropic liquid (Fig. S49†). The synchrotron XRD results for 1c in the state obtained by recrystallization showed the formation of the Col_r_ phase, with a repeating distance of 0.93 nm (Fig. S54†), which was similar to those of 1b and 1d in the single-crystal and mesophase forms, respectively. In 1c with shorter aliphatic chains, the interactions between the core π-electronic systems were predominant in forming triple deckers and their assemblies, resulting in higher crystallinity. In contrast, 1e, with longer aliphatic chains, exhibited mesophases, wherein the stacking distance between the π units was relatively long (0.40 nm) in the lamellar phases. These longer aliphatic chains more significantly influenced on the formation of assemblies, resulting in difficulty with the close stacking of π units because of the relatively smaller interactions between the core π-electronic systems. On the other hand, 1d with aliphatic chains of a moderate length could also form mesophases, wherein the core π-electronic system played an essential role in the aggregate formation, as Col_h_ and Col_r_ structures with close stacking.

The phase transition behaviours of analogous porphyrins 2c–e with the corresponding aryl moieties to those of 1c–e at the 5,15-positions, respectively, were also examined (Fig. S53b†). In contrast to 1d, dodecyloxy 2d formed a lamellar structure instead of columnar phases (Fig. S64†). Hexadecyloxy 2e formed a lamellar structure, with a repeating distance similar to that of 1e, which was maintained during the cooling and heating processes (Fig. S66†). On the other hand, octyloxy-substituted 2c, which had a lower melting point (68 °C) than 1c, showed no ordering behaviour during the cooling process (Fig. S62†), and a crystal-like POM texture was observed during the heating process (Fig. S50†). These observations suggested that, for the derivatives with longer aliphatic substituents, the interactions between the aliphatic groups were more significant than those between the π-planes, inducing a similar tendency to form lamellar structures. For the derivatives with moderately long aliphatic chains, distinct assembled structures were formed according to the interactions between π-planes in the norcorroles and porphyrins.

### Electric conductive property

**Fig. 8:**
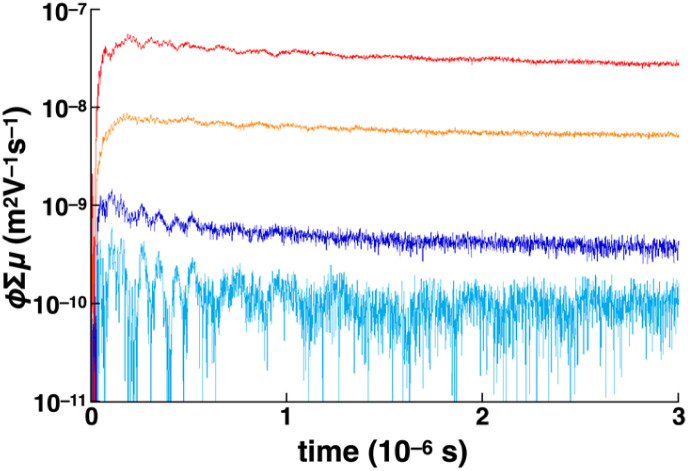
Kinetic traces of transient photoconductivity observed for 1a (light blue), 1b (orange), 1d (red) and 1e (blue) upon excitation at 355 nm, 2.7 × 10^15^ photons cm^−2^, at 20, 20, 25 and 50 °C, respectively.

Owing to the close stacking modes in the Col_h_ and Col_r_ phases of 1d with the wide range of continuous mesophases, the electric conductive nature of the phases was examined using flash-photolysis time-resolved microwave conductivity (FP-TRMC) measurements upon charge carrier photo-injection. The liquid crystalline sample of 1d was investigated after heating to the isotropic liquid state and processes for phase stability at each step. The short-range proximity of the Ni^II^ norcorrole framework of crystal-state 1b as a reference was confined to the trimer forms in the condensed phase, disrupting the 1D electric conductive pathways. The advantages of the 1D continuous stacking modes in the mesophases of 1d were clearly shown at 25 °C (Fig. 8). The maximum photo-conductivity of 1d (5.2 × 10^−8^ m^2^ V^−1^ s^−1^) was over five times of that of 1b and greater than those of other reported norcorrole triple deckers, including that of *meso*-phenyl derivative, in the form of single crystals.^6*c*,*f*^ These observations suggested that alkoxy chains contributed to the arrangement of triple deckers, resulting into the enhanced electric conductivity. In addition, the photo-conductivities of 1d and 1b were significantly larger than those of slip-stacked 1e (liquid crystal) and 1a (crystal), respectively, probably due to the ordered arrangement of tightly stacked triple deckers. The conductivity of 1d was also higher than the previously reported value for a norcorrole supramolecular polymer (1 × 10^−8^ m^2^ V^−1^ s^−1^).^6*h*^ Upon heating 1d from 25 °C, partial disruption of the conducting pathways was observed with an initial rapid decay until a few microseconds due to the quenching of electrons *via* residual oxygen molecules in the system (Fig. S80a†).

## Conclusions

Ni^II^ norcorrole derivatives with alkoxy chains having various lengths exhibited unique assembling behaviours and enhanced electric conductivity. The norcorroles with specific alkoxy chains formed triple-decker structures, derived from the stacked-ring aromaticity, in the crystal and mesophase states. As observed in the crystal structure, concave faces of the bowl-shaped top and bottom units of the triple deckers hindered effective stacking of the norcorrole trimer. In addition, the norcorrole trimer core was surrounded by alkyl chains, resulting in a double concave discotic mesogen. In the LC state, the stacked triple-decker structure was in contact with adjacent triple deckers at the edges of both concave faces, providing a novel nanoscale phase-segregated columnar assembly *via* the interactions between the edges of the norcorrole concave faces, along with those between the core π-electronic units and side aliphatic chains. The assembling modes in the LC states were substantially different from those of ordinary aromatic systems, showing more ordered segregated structures *via* independent interactions between the core π-electronic units and aliphatic chains. Even a slight overlap between the triple deckers led to high electric conductivity owing to the dynamic behaviours with a suitable arrangement in the LC state. Further modifications of antiaromatic π-electronic systems to obtain functional electronic materials are currently under investigation.

## Data availability

Data supporting the work in this publication are available *via* the ESI† and associated crystallographic data.

## Author contributions

H. M. designed and conducted the project. S. I., K. Y., S. Su., Y. H. and Y. B. carried out the synthesis, characterization and property examinations. S. Sa. and G. W. conducted the MD calculations. Y. H. K. and S. Se. evaluated electric conductivities. H. S. supported the synthesis.

## Conflicts of interest

There are no conflicts of interest to declare.

## Supplementary Material

SC-015-D4SC01633E-s001

SC-015-D4SC01633E-s002
