# Supplementary material for: Norcorroles as antiaromatic π-electronic systems that form dimension-controlled assemblies
Source: Chem Sci. 2024 Apr 16;15(20):7603–9. doi: 10.1039/d4sc01633e (PMC11110129; doi:10.1039/d4sc01633e)
Supplement: SC-015-D4SC01633E-s001 [file SC-015-D4SC01633E-s001.pdf]

Electronic Supplementary Material (ESI) for Chemical Science.
This journal is © The Royal Society of Chemistry 2024

Electronic Supplementary Information

Norcorroles as antiaromatic π-electronic systems that form dimension-controlled assemblies

Soh Ishikawa, Kazuhisa Yamasumi, Shinya Sugiura, Shunsuke Sato, Go Watanabe, Yun Hee Koo, Shu Seki, Yuya Bando, Yohei Haketa, Hiroshi Shinokubo and Hiromitsu Maeda*

Department of Applied Chemistry, College of Life Sciences, Ritsumeikan University, Kusatsu 525–8577, Japan, Fax: +81 77 561 2659; Tel: +81 77 561 5969; E-mail: maedahir@ph.ritsumei.ac.jp, Department of Physics, School of Science, Kitasato University, Sagamihara 252–0373, Japan, Department of Data Science, School of Frontier Engineering, Kitasato University, Sagamihara 252–0373, Japan, Department of Molecular Engineering, Graduate School of Engineering, Kyoto University, Kyoto 615–8510, Japan, and Department of Molecular and Macromolecular Chemistry, Graduate School of Engineering, Nagoya University, Nagoya 464–8603, Japan

Table of contents

1. Synthetic procedures and spectroscopic data S2
Fig. S1 Synthesis of *meso*-aryl-substituted norcorroles. S2
Fig. S2–19 ^{1}H and ^{13}C NMR spectra. S10
Fig. S20,21 UV/vis absorption spectra and cyclic voltammogram. S28
2. X-ray crystallographic data S29
Fig. S22–25 Ortep drawings. S30
Fig. S26–31 Packing diagrams. S31
Fig. S32 Bond lengths and HOMA. S34
Fig. S33–35 Hirshfeld surfaces. S34
3. Theoretical studies S37
Fig. S36,37 Optimized structures. S37
Fig. S38 Bond lengths and HOMA. S37
Fig. S39 ESP mapping. S38
Fig. S40–44 Molecular orbitals and TD-DFT calculations. S39
Fig. S45–47 Theoretical calculation for 2D NICS. S43
Fig. S48 Theoretical calculation for ACID. S46
Cartesian coordination of optimized structures S46
4. Assembled behaviors S51
Fig. S49 DSC thermograms. S52
Fig. S50–52 POM images. S52
Fig. S53 Details of phase transition behaviors. S55
Fig. S54–66 XRD patterns and proposed packing models. S56
Fig. S67–73 MD simulation S83
Fig. S74–76 UV/vis absorption spectra and XRD analysis of assemblies. S86
Fig. S77–79 ^{1}H NMR spectra of assemblies. S88
5. Electric conductivity S91
Fig. S80,81 Transient photoconductivity and photocurrent. S91

1. Synthetic procedures and spectroscopic data

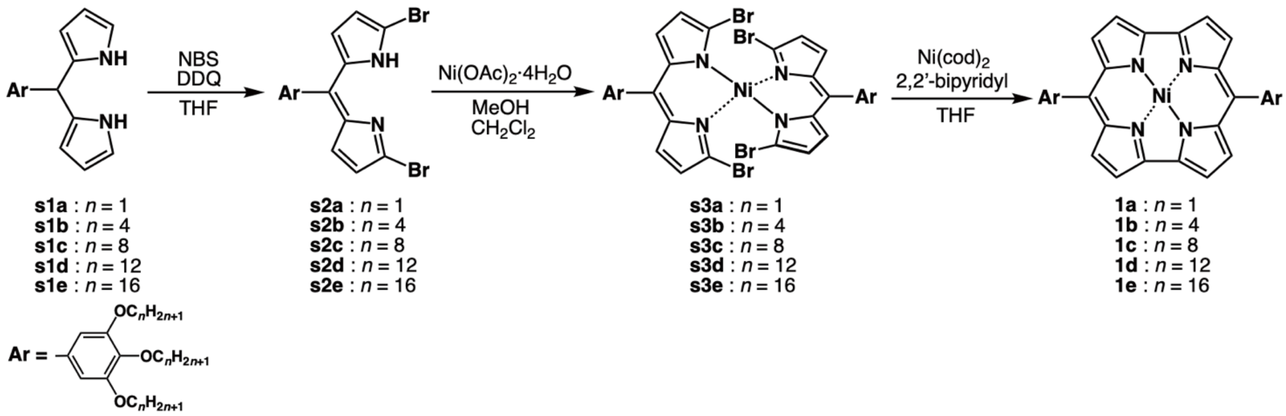

Fig. S1 Synthesis of *meso*-aryl-substituted norcorroles.

General Procedures. Starting materials were purchased from FUJIFILM Wako Pure Chemical Corp., Nacalai Tesque Inc., and Sigma-Aldrich Co. and were used without further purification unless otherwise stated. NMR spectra used in the characterization of products were recorded on a JEOL ECA-600 600 MHz spectrometers. All NMR spectra were referenced to solvent. UV-visible absorption spectra were recorded on a Hitachi U-3500 spectrometer. Matrix-assisted laser desorption ionization time-of-flight mass spectrometry (MALDI-TOF-MS) was recorded on a Shimadzu Axima-CFRplus. Electrospray ionization mass spectrometry (ESI-MS) was recorded on a BRUKER microTOF using ESI-TOF method. TLC analyses were carried out on aluminum sheets coated with silica gel 60 (Merck 5554). Column chromatography was performed on Wakogel C-300, Merck silica gel 60, and activated alumina. HPLC separation was a recycling preparative HPLC (Japan Analytical Industry Co., Ltd.) series connection of JAIGEL-1H-A and JAIGEL-2H-A (GPC) and UV detector using $CHCl_3$ as an eluent at 3.5 mL/min flow rate.

5-(3,4,5-Tributoxyphenyl)dipyrromethane, s1b. A solution of 3,4,5-tributoxybenzaldehyde[S1] (4.846 g, 15.0 mmol) in pyrrole (348 mL) was degassed by bubbling with N_2 for 30 min, followed by the addition of TFA (1.05 mL). The solution was stirred at r.t. for 20 min, then was quenched by adding NaOH (15.0 g) and was filtered to remove NaOH. After the removal of NaOH, pyrrole was recovered by distillation under vacuum. The residue was purified by silica gel column chromatography (Wakogel C-300; eluent: CH_2Cl_2) and was recrystallized from CH_2Cl_2/MeOH to afford **s1b** (5.014 g, 11.4 mmol, 76%) as a white solid. R_f = 0.67 (CH_2Cl_2). ^{1}H NMR (600 MHz, $CDCl_3$, 20 °C): δ (ppm) 7.94 (br, 2H, NH), 6.70 (m, 2H, pyrrole-H), 6.40 (s, Ar-CH), 6.16 (dd, J = 5.4 and 3.0 Hz, 2H, pyrrole-H), 5.96–5.95 (m, 2H, pyrrole-H), 5.38 (s, 1H, *meso*-CH), 3.94 (t, J = 6.6 Hz, 2H, OCH_2), 3.88 (t, J = 6.6 Hz, 4H, OCH_2), 1.75–1.69 (m, 6H, $OCH_2\mathit{CH_2}$), 1.54–1.43 (m, 6H, $OC_2H_4\mathit{CH_2}$), 0.954 (t, J = 7.2 Hz, 3H, OCH_3), 0.945 (t, J = 7.2 Hz, 6H, OCH_3). ^{13}C{^{1}H} NMR (151 MHz, $CDCl_3$, 20 °C): δ(ppm) 152.98, 137.35, 136.59, 132.64, 117.20, 108.21, 107.17, 106.78, 73.26, 68.66, 44.06, 32.32, 31.49, 19.35, 19.25, 14.00, 13.95. ESI-TOF-MS (HR): 437.2809. Calcd for $C_{27}H_{37}N_2O_3$ ([M – H]$^-$): 437.2810.

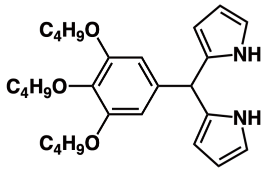

5-(3,4,5-Trioctyloxyphenyl)dipyrromethane, s1c. A solution of 3,4,5-trioctyloxybenzaldehyde[S2] (4.980 g, 10.1 mmol) in pyrrole (235 mL) was degassed by bubbling with N_2 for 30 min, followed by the addition of TFA (0.71 mL). The solution was stirred at r.t. for 20 min, then was quenched by adding NaOH (12.0 g) and was filtered to remove NaOH. After the removal of NaOH, pyrrole was recovered by distillation under vacuum. The residue was purified by silica gel column chromatography (Wakogel C-300; eluent: 5% EtOAc/*n*-hexane) to afford **s1c** (5.106 g, 8.41 mmol, 83%) as a brown oil. R_f = 0.24 (5% EtOAc/*n*-hexane). ^{1}H NMR (600 MHz, $CDCl_3$, 20 °C): δ (ppm) 7.93 (br, 2H, NH), 6.70 (m, 2H, pyrrole-H), 6.40 (s, 2H, Ar-CH), 6.16 (dd, J = 6.0 and 3.0 Hz, 2H, pyrrole-H), 5.95 (m, 2H, pyrrole-H), 5.38 (s, 1H, *meso*-CH), 3.92 (t, J = 7.2 Hz, 2H, OCH_2), 3.87 (t, J = 6.6 Hz, 4H, OCH_2), 1.76–1.71 (m, 6H, $OCH_2\mathit{CH_2}$), 1.47–1.40 (m, 6H, $OC_2H_4\mathit{CH_2}$), 1.31–1.27 (m, 24H, $OC_3H_6\mathit{C_4H_8}$), 0.88 (t, J = 7.2 Hz, 9H, OCH_3). ^{13}C{^{1}H} NMR (151 MHz, $CDCl_3$, 20 °C): δ(ppm) 153.18, 137.17, 136.96, 132.62, 117.20, 108.42, 107.22, 106.98, 73.62, 69.13, 44.20, 32.03, 31.96, 30.43, 29.69, 29.53, 29.42, 26.23, 22.83, 22.80, 14.24 (some signals of octyl chains were overlapped). ESI-TOF-MS (HR): 605.4685. Calcd for $C_{39}H_{61}N_2O_3$ ([M – H]$^-$): 605.4688.

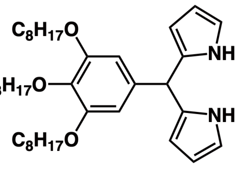

2,8-Dibromo-5-(3,4,5-trimethoxyphenyl)dipyrrin, s2a. According to the literature procedure,[S3] a two-necked flask containing 5-(3,4,5-trimethoxyphenyl)dipyrromethane[S4] **s1a** (821.5 mg, 2.63 mmol) was evacuated and then was refilled with N_2. To the flask, dry THF (50 mL) was added and the solution was cooled to –78 °C. After N-bromosuccinimide (NBS) (982.5 mg, 5.52 mmol) was added to the solution in three portions at a 5-min interval, the mixture was stirred for 1 h. Then 2,3-dichloro-5,6-dicyano-1,4-benzoquinone (DDQ) (896.7 mg, 3.95 mmol) was added. After stirred at –78 °C for 15 min, the reaction mixture was quickly filtered through a short pad of alumina column (eluent: CH_2Cl_2). The evaporation to dryness and recrystallization from CH_2Cl_2/MeOH afforded **s2a** (1.227 g, 2.62 mmol, 99%) as an orange solid. R_f = 0.31 (CH_2Cl_2). ^{1}H NMR (600 MHz, $CDCl_3$, 20 °C): δ (ppm) 12.46 (br, 1H, NH), 6.68 (s, 2H, Ar-CH), 6.59 (d, J = 3.6 Hz, 2H, pyrrole-H), 6.35 (d, J = 4.2 Hz, 2H, pyrrole-H), 3.93 (s, 3H, CH_3), 3.85 (s, 6H, CH_3). ^{13}C{^{1}H} NMR (151 MHz, $CDCl_3$, 20 °C): δ (ppm) 152.69, 140.28, 140.12, 139.33, 130.17, 129.82, 129.62, 120.55, 108.51, 61.12, 56.39. MALDI-TOF-MS: m/z (% intensity): 467.0 (78), 468.0 (44), 469.0 (100). Calcd for $C_{18}H_{17}Br_2N_2O_3$ ([M + H]$^+$): 466.96. This compound was further characterized by single-crystal X-ray diffraction analysis.

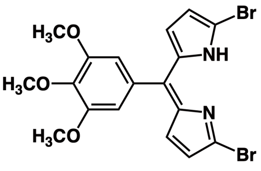

2,8-Dibromo-5-(3,4,5-tributoxyphenyl)dipyrrin, s2b. According to the literature procedure,[S3] a two-necked flask containing **s1b** (2.307 g, 5.26 mmol) was evacuated and then was refilled with N_2. To the flask, dry THF (100 mL) was added and the solution was cooled to –78 °C. After NBS (1.962 g, 11.0 mmol) was added to the solution in two portions at a 10-min interval, the mixture was stirred for 1 h. Then DDQ (1.792 g, 7.90 mmol) was added. After stirred at –78 °C for 15 min, the reaction mixture was quickly filtered through a short pad of alumina column (eluent: CH_2Cl_2). The mixture was washed with $Na_2S_2O_3\cdot5H_2O$ aq., and was dried over anhydrous Na_2SO_4, followed by the filtration and evaporation to dryness. The residue was purified by silica gel column chromatography (Wakogel C-300; eluent: 5% EtOAc/*n*-hexane) to afford **s2b** (2.989 g, 5.03 mmol, 96%) as an orange oil. R_f = 0.28 (5% EtOAc/*n*-hexane). ^{1}H NMR (600 MHz, $CDCl_3$, 20 °C): δ (ppm) 12.45 (br, 1H, NH), 6.64 (s, 2H, Ar-CH), 6.60 (d, J = 4.2 Hz, 2H, pyrrole-H), 6.35 (d, J = 4.8 Hz, 2H, pyrrole-H), 4.04 (t, J = 6.6 Hz, 2H, OCH_2), 3.95 (t, J = 6.6 Hz, 4H, OCH_2), 1.79–1.74 (m, 6H, OCH_2CH_2), 1.57–1.46 (m, 6H, OCH_2CH_2), 0.98 (t, J = 7.2 Hz, 3H, CH_3), 0.96 (t, J = 7.8 Hz, 6H, CH_3). ^{13}C{^{1}H} NMR (151 MHz, $CDCl_3$, 20 °C): δ (ppm) 152.59, 140.33, 139.87, 139.41, 130.41, 130.32, 129.41, 120.45, 109.93, 73.33, 69.09, 32.53, 31.56, 19.41, 19.35, 14.08, 14.02. MALDI-TOF-MS: m/z (% intensity): 593.1 (78), 594.1 (42), 595.1 (100). Calcd for $C_{27}H_{35}Br_2N_2O_3$ ([M + H]$^+$): 593.10.

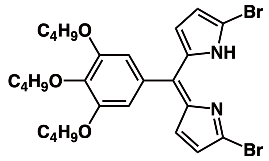

2,8-Dibromo-5-(3,4,5-trioctyloxyphenyl)dipyrrin, s2c. According to the literature procedure,[S3] a two-necked flask containing **s1c** (801.1 g, 1.32 mmol) was evacuated and then was refilled with N_2. To the flask, dry THF (30 mL) was added and the solution was cooled to –78 °C. After NBS (491.3 mg, 2.76 mmol) was added to the solution in two portions at a 10-min interval, the mixture was stirred for 1 h. Then DDQ (449.5 mg, 1.98 mmol) was added. After stirred at –78 °C for 15 min, the reaction mixture was quickly filtered through a short pad of alumina column (eluent: CH_2Cl_2). The mixture was washed with $Na_2S_2O_3\cdot5H_2O$ aq., and was dried over anhydrous Na_2SO_4, followed by the filtration and evaporation to dryness. The residue was purified by silica gel column chromatography (Wakogel C-300; eluent: 2% EtOAc/*n*-hexane) to afford **s2c** (606.6 mg, 0.795 mmol, 60%) as an orange oil. R_f = 0.19 (2% EtOAc/*n*-hexane). ^{1}H NMR (600 MHz, $CDCl_3$, 20 °C): δ (ppm) 12.45 (br, 1H, NH), 6.63 (s, 2H, Ar-CH), 6.59 (d, J = 4.2 Hz, 2H, pyrrole-H), 6.34 (d, J = 4.2 Hz, 2H, pyrrole-H), 4.03 (t, J = 7.2 Hz, 2H, OCH_2), 3.94 (t, J = 6.6 Hz, 4H, OCH_2). 1.81–1.76 (m, 6H, OCH_2CH_2), 1.52–1.43 (m, 6H, $OC_2H_4CH_2$), 1.37–1.24 (m, 24H, $OC_3H_6C_4H_8$), 0.90–0.87 (m, 9H, CH_3). ^{13}C{^{1}H} NMR (151 MHz, $CDCl_3$, 20 °C): δ (ppm) 152.58, 140.34, 139.88, 139.42, 130.39, 130.32, 129.41, 120.44, 109.93, 73.71, 69.40, 32.07, 31.97, 30.53, 29.71, 29.55, 29.53, 29.43, 26.27, 26.22, 22.87, 22.82, 14.28, 14.26 (a signal of octyl chains was overlapped). MALDI-TOF-MS: m/z (% intensity): 761.3 (70), 762.3 (28), 763.3 (100). Calcd for $C_{39}H_{59}Br_2N_2O_3$ ([M + H]$^+$): 761.29.

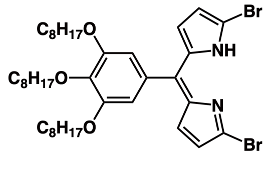

2,8-Dibromo-5-(3,4,5-tridodecyloxyphenyl)dipyrrin, s2d. According to the literature procedure,[S3] a two-necked flask containing 5-(3,4,5-tridodecyloxyphenyl)dipyrromethane[S5] **s1d** (2.038 g, 2.63 mmol) was evacuated and then was refilled with N_2. To the flask, dry THF (158 mL) was added and the solution was cooled to –60 °C. After NBS (982.5 mg, 5.52 mmol) was added to the solution in two portions at a

10-min interval, the mixture was stirred for 1 h. Then DDQ (896.7 mg, 3.95 mmol) was added. After stirred at –60 °C for 15 min, the reaction mixture was quickly filtered through a short pad of alumina column (eluent: CH_2Cl_2). The mixture was washed with $Na_2S_2O_3 \cdot 5H_2O$ aq., and was dried over anhydrous Na_2SO_4, followed by the filtration and evaporation to dryness. The residue was purified by silica gel column chromatography (Wakogel C-300; eluent: CH_2Cl_2/*n*-hexane = 1:2) and was recrystallized from CH_2Cl_2/MeOH to afford **s2d** (2.104 g, 2.26 mmol, 86%) as an orange solid. R_f = 0.36 (CH_2Cl_2/*n*-hexane = 2:3). ^{1}H NMR (600 MHz, $CDCl_3$, 20 °C): δ(ppm) 12.46 (br, 1H, NH), 6.63 (s, 2H, Ar-CH), 6.59 (d, J = 4.8 Hz, 2H, pyrrole-H), 6.34 (d, J = 3.6 Hz, 2H, pyrrole-H), 4.03 (t, J = 6.6 Hz, 2H, OCH_2), 3.94 (t, J = 6.6 Hz, 4H, OCH_2), 1.81–1.75 (m, 6H, $OCH_2\mathit{CH_2}$), 1.50 (quin, J = 7.2 Hz, 2H, $OC_2H_4\mathit{CH_2}$), 1.45 (quin, J = 7.8 Hz, 4H, $OC_2H_4\mathit{CH_2}$), 1.37–1.25 (m, 48H, $OC_3H_6\mathit{C_8H_{16}}$), 0.880 (t, J = 7.2 Hz, 3H, CH_3), 0.876 (t, J = 7.2 Hz, 6H, CH_3). ^{13}C{^{1}H} NMR (151 MHz, $CDCl_3$, 20 °C): δ(ppm) 152.58, 140.34, 139.86, 139.45, 130.39, 130.29, 129.39, 120.43, 109.98, 73.70, 69.42, 32.10, 32.07, 30.54, 29.92, 29.90, 29.85, 29.81, 29.77, 29.57, 29.55, 29.52, 26.28, 26.23, 22.84, 14.26 (some signals of dodecyl chains were overlapped). MALDI-TOF-MS: *m/z* (% intensity): 929.4 (56), 930.4 (27), 931.4 (100). Calcd for $C_{51}H_{83}Br_2N_2O_3$ ([M + H]$^+$): 929.48.

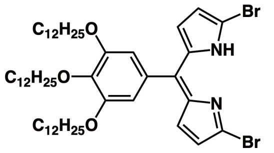

2,8-Dibromo-5-(3,4,5-trihexadecyloxyphenyl)dipyrrin, s2e. According to the literature procedure,[S3] a two-necked flask containing 5-(3,4,5-trihexadecyloxyphenyl)dipyrromethane[S6] **s1e** (2.450 g, 2.59 mmol) was evacuated and then was refilled with N_2. To the flask, dry THF (260 mL) was added and the solution was cooled to –35 °C. After NBS (968 mg, 5.44 mmol) was added to the solution in two portions at a 10-min interval, the mixture was stirred for 1 h. Then DDQ (883 mg, 3.89 mmol) was added. After stirred at –35 °C for 15 min, the reaction mixture was quickly filtered through a short pad of alumina column (eluent: CH_2Cl_2). The mixture was washed with $Na_2S_2O_3 \cdot 5H_2O$ aq., and was dried over anhydrous Na_2SO_4, followed by the filtration and evaporation to dryness. The residue was purified by silica gel column chromatography (Wakogel C-300; eluent: CH_2Cl_2/*n*-hexane = 1:2) and was recrystallized from CH_2Cl_2/MeOH to afford **s2e** (2.036 g, 1.85 mmol, 71%) as an orange solid. R_f = 0.42 (CH_2Cl_2/*n*-hexane = 2:3). ^{1}H NMR (600 MHz, $CDCl_3$, 20 °C): δ(ppm) 12.47 (br, 1H, NH), 6.63 (s, 2H, Ar-CH), 6.59 (d, J = 4.2 Hz, 2H, pyrrole-H), 6.34 (d, J = 3.6 Hz, 2H, pyrrole-H), 4.03 (t, J = 6.6 Hz, 2H, OCH_2), 3.94 (t, J = 6.6 Hz, 4H, OCH_2), 1.81–1.75 (m, 6H, $OCH_2\mathit{CH_2}$), 1.50 (quin, J = 7.8 Hz, 2H, $OC_2H_4\mathit{CH_2}$), 1.45 (quin, J = 7.8 Hz, 4H, $OC_2H_4\mathit{CH_2}$), 1.37–1.25 (m, 72H, $OC_3H_6\mathit{C_{12}H_{24}}$), 0.879 (t, J = 7.2 Hz, 3H, CH_3), 0.877 (t, J = 7.2 Hz, 6H, CH_3). ^{13}C{^{1}H} NMR (151 MHz, $CDCl_3$, 20 °C): δ (ppm) 152.58, 140.35, 139.85, 139.46, 130.39, 130.29, 129.39, 120.43, 109.98, 73.70, 69.42, 32.08, 30.54, 29.91, 29.87, 29.82, 29.78, 29.58, 29.53, 26.29, 26.23, 22.84, 14.26 (some signals of hexadecyl chains were overlapped). MALDI-TOF-MS: *m/z* (% intensity): 1097.7 (51), 1098.7 (46), 1099.7 (100). Calcd for $C_{63}H_{107}Br_2N_2O_3$ ([M + H]$^+$): 1097.66.

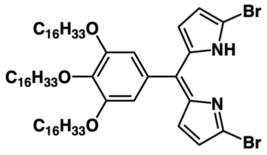

2,8-Dibromo-5-(3,4,5-trimethoxyphenyl)dipyrrin Ni^{II} complex, s3a. According to the literature procedure,[S3] to the solution of **s2a** (1.227 g, 2.62 mmol) dissolved in CH_2Cl_2 (35 mL) were added NaOAc (324 mg, 3.95 mmol) and subsequently a solution of $Ni(OAc)_2 \cdot 4H_2O$ (329 mg, 1.32 mmol) in MeOH (20 mL). The mixture was stirred at r.t. for 2 h. After the solvent was removed under reduced pressure, the residue was recrystallized from CH_2Cl_2/MeOH to afford **s3a** (1.195 g, 1.20 mmol, 92%) as a green solid. R_f = 0.57 (5% EtOAc/CH_2Cl_2). The NMR spectra of this compound were not observed owing to the paramagnetic nature.[S7] ESI-TOF-MS (HR): 988.8324. Calcd for $C_{36}H_{31}Br_4N_4NiO_6$ ([M + H]$^+$): 988.8325. This compound was further characterized by single-crystal X-ray diffraction analysis.

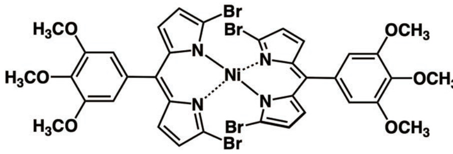

2,8-Dibromo-5-(3,4,5-tributoxyphenyl)dipyrrin Ni^{II} complex, s3b. According to the literature procedure,[S3] to the solution of **s2b** (1.563 g, 2.63 mmol) dissolved in CH_2Cl_2 (35 mL) were added NaOAc (324 mg, 3.95 mmol) and subsequently a solution of $Ni(OAc)_2 \cdot 4H_2O$ (327 mg, 1.32 mmol) in MeOH (20 mL). The mixture was stirred at r.t. for 2 h. After the solvent was removed under reduced pressure, the residue was purified by silica gel column chromatography (Wakogel C-300; eluent: 5% EtOAc/CH_2Cl_2) to afford **s3b** (1.431 g, 1.15 mmol, 87%) as a dark red oil. R_f = 0.38 (5% EtOAc/CH_2Cl_2). The NMR spectra of this compound were not observed owing to the paramagnetic nature.[S7] ESI-TOF-MS (HR): 1241.1144. Calcd for $C_{54}H_{67}Br_4N_4NiO_6$ ([M + H]$^+$): 1241.1142.

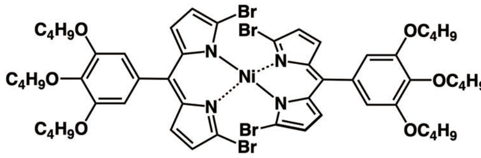

2,8-Dibromo-5-(3,4,5-trioctyloxyphenyl)dipyrrin Ni^{II} complex, s3c. According to the literature procedure,[S3] to the solution of **s2c** (509.5 mg, 0.668 mmol) dissolved in CH_2Cl_2 (10 mL) were added NaOAc (84.9 mg, 1.03 mmol) and subsequently a solution of $Ni(OAc)_2 \cdot 4H_2O$

(96.5 mg, 0.388 mmol) in MeOH (5 mL). The mixture was stirred at r.t. for 2 h. After the solvent was removed under reduced pressure, the residue was purified by silica gel column chromatography (Wakogel C-300; eluent: 2% EtOAc/CH_2Cl_2) to afford **s3c** (501.4 mg, 0.317 mmol, 95%) as a dark red oil. R_f = 0.25 (2% EtOAc/CH_2Cl_2). The NMR spectra of this compound were not observed owing to the paramagnetic nature.[S7] ESI-TOF-MS (HR): 1577.4896. Calcd for $C_{78}H_{115}Br_4N_4NiO_6$ ($[M + H]^+$): 1577.4898.

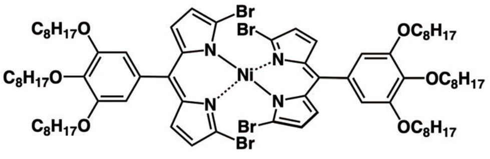

2,8-Dibromo-5-(3,4,5-tridodecyloxyphenyl)dipyrrin Ni^{II} complex, s3d. According to the literature procedure,[S3] to the solution of **s2d** (1.874 g, 2.01 mmol) dissolved in CH_2Cl_2 (92 mL) were added NaOAc (249 mg, 3.04 mmol) and subsequently a solution of $Ni(OAc)_2 \cdot 4H_2O$ (301 mg, 1.21 mmol) in MeOH (15 mL). The mixture was stirred at r.t. for 2 h. After the solvent was removed under reduced pressure, the residue was purified by silica gel column chromatography (Wakogel C-300; eluent: CH_2Cl_2/*n*-hexane = 1:2) and was recrystallized from CH_2Cl_2/MeOH to afford **s3d** (1.890 g, 0.985 mmol, 98%) as a dark red solid. R_f = 0.29 (CH_2Cl_2/*n*-hexane = 2:3). The NMR spectra of this compound were not observed owing to the paramagnetic nature.[S7] ESI-TOF-MS: *m/z* (% intensity): 1913.9 (12), 1914.9 (20), 1915.9 (46), 1916.9 (54), 1917.9 (92), 1918.9 (91), 1919.9 (100). Calcd for $C_{102}H_{163}Br_4N_4NiO_6$ ($[M + H]^+$): 1913.86.

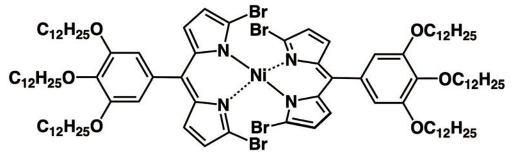

2,8-Dibromo-5-(3,4,5-trihexadecyloxyphenyl)dipyrrin Ni^{II} complex, s3e. According to the literature procedure,[S3] to the solution of **s2e** (962 mg, 0.875 mmol) dissolved in CH_2Cl_2 (45 mL) were added NaOAc (108 mg, 1.31 mmol) and subsequently a solution of $Ni(OAc)_2 \cdot 4H_2O$ (131 mg, 0.526 mmol) in MeOH (7 mL). The mixture was stirred at r.t. for 2 h. After the solvent was removed under reduced pressure, the residue was purified by silica gel column chromatography (Wakogel C-300; eluent: CH_2Cl_2/*n*-hexane = 1:2) and was recrystallized from CH_2Cl_2/MeOH to afford **s3e** (948.9 mg, 0.421 mmol, 96%) as a dark red solid. R_f = 0.40 (CH_2Cl_2/*n*-hexane = 2:3). The NMR spectra of this compound were not observed owing to the paramagnetic nature.[S7] ESI-TOF-MS: *m/z* (% intensity): 2249.2 (33), 2250.2 (35), 2251.2 (47), 2252.2(62), 2253.2 (82), 2254.2 (90), 2255.2 (100). Calcd for $C_{126}H_{210}Br_4N_4NiO_6$ ($[M]^+$): 2249.23.

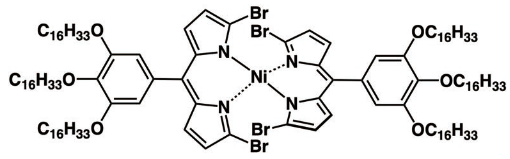

***meso*-(3,4,5-Trimethoxyphenyl)norcorrole Ni^{II} complex, 1a.** According to the literature procedure,[S3] **s3a** (595.8 mg, 0.600 mmol), $Ni(cod)_2$ (cod: 1,5-cyclooctadiene) (412.6 mg, 1.50 mmol), and 2,2′-bipyridyl (234.2 mg, 1.50 mmol) were dissolved in dehydrated THF (90 mL) in the glove box. After taking the flask out of the glove box, the solution was stirred at r.t. for 5 h. The reaction mixture was immediately filtered through a short pad of alumina column (eluent: $CHCl_3$), and the solvent was removed in vacuo. The residue was purified by silica gel column chromatography (Wakogel C-300; eluent: 5% EtOAc/CH_2Cl_2) and was recrystallized from CH_2Cl_2/MeOH to afford **1a** (132.4 mg, 0.197 mmol, 32%) as a black solid. R_f = 0.21 (5% EtOAc/CH_2Cl_2). ^1H NMR (600 MHz, $CDCl_3$, 20 °C): δ (ppm) 5.31 (s, 4H, Ar-CH), 3.54 (s, 6H, CH_3), 3.47 (s, 12H, CH_3), 2.52 (d, J = 4.2 Hz, 4H, β-CH), 2.08 (d, J = 4.8 Hz, 4H, β-CH). $^{13}C\{^1H\}$ NMR (151 MHz, $CDCl_3$, 20 °C): δ (ppm) 166.05, 156.13, 152.21, 146.37, 140.38, 130.73, 127.90, 115.56, 99.67, 60.76, 55.87. UV/vis (CH_2Cl_2, λ_{max}[nm] (ε, 10^4 $M^{-1}cm^{-1}$)): 427 (4.2), 497 (4.1), 524 (4.2). MALDI-TOF-MS: *m/z* (% intensity): 672.1 (100), 673.1 (47). Calcd for $C_{36}H_{30}N_4NiO_6$ ($[M]^+$): 672.15.

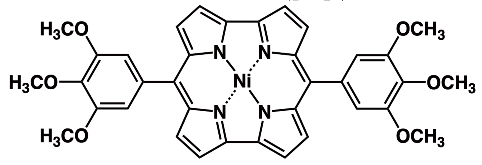

***meso*-(3,4,5-Tributoxyphenyl)norcorrole Ni^{II} complex, 1b.** According to the literature procedure,[S3] **s3b** (728.0 mg, 0.585 mmol), $Ni(cod)_2$ (402.4 mg, 1.463 mmol), and 2,2′-bipyridyl (228.5 mg, 1.463 mmol) were dissolved in dehydrated THF (117 mL) in the glove box. After taking the flask out of the glove box, the solution was stirred at r.t. for 3.5 h. The reaction mixture was immediately filtered through a short pad of alumina column (eluent: $CHCl_3$), and the solvent was removed in vacuo. The residue was purified by silica gel column chromatography (Wakogel C-300; eluent: 5% EtOAc/*n*-hexane) and flash silica gel column chromatography (eluent: 5% EtOAc/*n*-hexane). The solvent was removed under vacuum. The residue was purified with a recycling preparative HPLC (JAIGEL-1H-A and JAIGEL-2H-A; eluent: $CHCl_3$) and was recrystallized from CH_2Cl_2/MeOH to afford **1b** (46.7 mg, 0.0504 mmol, 8.6%) as a black solid. R_f = 0.31 (10% EtOAc/*n*-hexane). ^1H NMR (600 MHz, $CDCl_3$, 20 °C): δ (ppm) 5.23 (s, 4H, Ar-CH), 3.62 (t, J = 6.6 Hz, 4H, OCH_2), 3.54 (t, J = 7.2 Hz, 8H, OCH_2), 2.41 (d, J = 4.2 Hz, 4H, β-CH), 1.97 (d, J = 4.2 Hz, 4H, β-CH), 1.56 (quin, J = 7.2 Hz, 8H, $OCH_2\mathit{CH_2}$), 1.47 (quin, J = 7.2 Hz, 4H, $OCH_2\mathit{CH_2}$), 1.39–1.25 (m, 12H, $OC_2H_4\mathit{CH_2}$), 0.89 (t, J = 7.8 Hz, 12H, CH_3), 0.80 (t, J = 7.8 Hz, 6H, CH_3). $^{13}C\{^1H\}$ NMR (151 MHz, $CDCl_3$, 20 °C): δ

(ppm) 167.06, 158.51, 152.05, 146.58, 141.23, 130.88, 126.37, 114.59, 99.12, 72.96, 68.57, 32.22, 31.28, 19.26, 19.08, 13.92, 13.87. UV/vis (CH_2Cl_2, λ_{max}[nm] (ε, 10^4 $M^{-1}cm^{-1}$)): 426 (4.1), 528 (4.7). MALDI-TOF-MS: *m/z* (% intensity): 924.4 (100), 925.4 (66). Calcd for $C_{54}H_{66}N_4NiO_6$ ($[M]^+$): 924.43.

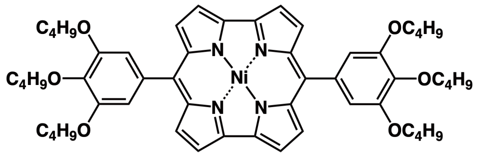

***meso*-(3,4,5-Trioctyloxyphenyl)norcorrole Ni^{II} complex, 1c.** According to the literature procedure,[S3] **s3c** (448.1 mg, 0.283 mmol), Ni(cod)$_2$ (194.7 mg, 0.708 mmol), and 2,2′-bipyridyl (110.6 mg, 0.708 mmol) were dissolved in dehydrated THF (57 mL) in the glove box. After taking the flask out of the glove box, the solution was stirred at r.t. for 3.5 h. The reaction mixture was immediately filtered through a short pad of alumina column (eluent: $CHCl_3$), and the solvent was removed in vacuo. The residue was purified by silica gel column chromatography (Wakogel C-300; eluent: 3% EtOAc/*n*-hexane) and flash silica gel column chromatography (eluent: 2.5% EtOAc/*n*-hexane). The solvent was removed under vacuum. The residue was purified with a recycling preparative HPLC (JAIGEL-1H-A and JAIGEL-2H-A; eluent: $CHCl_3$) and was recrystallized from CH_2Cl_2/MeOH to afford **1c** (48.37 mg, 0.0383 mmol, 14%) as a black solid. R_f = 0.41 (5% EtOAc/*n*-hexane). ^{1}H NMR (600 MHz, $CDCl_3$, 20 °C): δ (ppm) 5.26 (s, 4H, Ar-CH), 3.62 (t, J = 6.6 Hz, 4H, OCH_2), 3.52 (t, J = 6.6 Hz, 8H, OCH_2), 2.50 (d, J = 4.2 Hz, 4H, β-CH), 2.06 (d, J = 3.6 Hz, 4H, β-CH), 1.57 (quin, J = 6.6 Hz, 8H, OCH_2CH_2), 1.48 (quin, J = 7.2 Hz, 4H, OCH_2CH_2), 1.31–1.17 (m, 60H, $OC_2H_4C_5H_{10}$), 0.89 (t, J = 7.2 Hz, 12H, CH_3), 0.82 (t, J = 7.2 Hz, 6H, CH_3). $^{13}C\{^1H\}$ NMR (151 MHz, $CDCl_3$, 20 °C): δ (ppm) 167.01, 158.46, 152.04, 146.56, 141.22, 130.88, 126.40, 114.61, 99.16, 73.34, 68.89, 31.96, 30.23, 29.52, 29.44, 29.42, 29.38, 29.26, 26.10, 26.00, 22.82, 22.78, 14.27, 14.21 (some signals of octyl chains were overlapped). UV/vis (CH_2Cl_2, λ_{max}[nm] (ε, 10^4 $M^{-1}cm^{-1}$)): 426 (4.0), 528 (4.6). MALDI-TOF-MS: *m/z* (% intensity): 1260.9 (100), 1261.9 (90). Calcd for $C_{78}H_{114}N_4NiO_6$ ($[M]^+$): 1260.81.

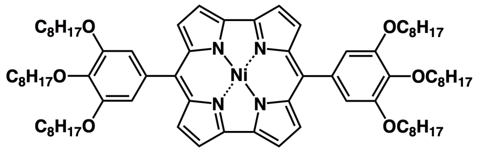

***meso*-(3,4,5-Tridodecyloxyphenyl)norcorrole Ni^{II} complex, 1d.** According to the literature procedure,[S3] **s3d** (575.6 mg, 0.300 mmol), Ni(cod)$_2$ (206.3 mg, 0.750 mmol), and 2,2′-bipyridyl (117.1 mg, 0.750 mmol) were dissolved in dehydrated THF (60 mL) in the glove box. After taking the flask out of the glove box, the solution was stirred at r.t. for 3.5 h. The reaction mixture was immediately filtered through a short pad of alumina column (eluent: $CHCl_3$), and the solvent was removed in vacuo. The residue was purified by silica gel column chromatography (Wakogel C-300; eluent: 2% CH_2Cl_2/*n*-hexane) and the solvent was removed under vacuum. The residue was purified with a recycling preparative HPLC (JAIGEL-1H-A and JAIGEL-2H-A; eluent: $CHCl_3$) and flash silica gel column chromatography (eluent: 2% CH_2Cl_2/*n*-hexane) and was recrystallized from CH_2Cl_2/MeOH to afford **1d** (172.4 mg, 0.108 mmol, 36%) as a dark brown solid. R_f = 0.45 (5% EtOAc/*n*-hexane). ^{1}H NMR (600 MHz, $CDCl_3$, 20 °C): δ (ppm) 5.24 (s, 4H, Ar-CH), 3.61 (t, J = 6.6 Hz, 4H, OCH_2), 3.52 (t, J = 6.6 Hz, 8H, OCH_2), 2.46 (d, J = 4.2 Hz, 4H, β-CH), 2.02 (d, J = 4.2 Hz, 4H, β-CH), 1.56 (quin, J = 7.2 Hz, 8H, OCH_2CH_2), 1.48 (quin, J = 7.2 Hz, 4H, OCH_2CH_2), 1.33–1.17 (m, 108H, $OC_2H_4C_9H_{18}$), 0.90 (t, J = 7.2 Hz, 12H, CH_3), 0.86 (t, J = 7.2 Hz, 6H, CH_3). $^{13}C\{^1H\}$ NMR (151 MHz, $CDCl_3$, 20 °C): δ (ppm) 167.31, 158.85, 152.06, 146.71, 141.31, 130.93, 126.22, 114.47, 98.94, 73.35, 68.93, 32.11, 32.06, 30.22, 29.86, 29.83, 29.78, 29.77, 29.74, 29.57, 29.55, 29.49, 29.27, 26.09, 26.00, 22.87, 22.82, 14.29, 14.24 (some signals of dodecyl chains were overlapped). UV/vis (CH_2Cl_2, λ_{max}[nm] (ε, 10^4 $M^{-1}cm^{-1}$)): 426 (4.1), 528 (4.7). MALDI-TOF-MS: *m/z* (% intensity): 1597.2 (71), 1598.2 (100). Calcd for $C_{102}H_{162}N_4NiO_6$ ($[M]^+$): 1597.18.

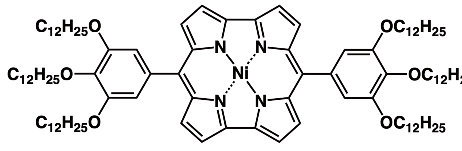

***meso*-(3,4,5-Trihexadecyloxyphenyl)-substituted norcorrole Ni^{II} complex, 1e.** According to the literature procedure,[S3] **s3e** (338.3 mg, 0.150 mmol), Ni(cod)$_2$ (103.2 mg, 0.375 mmol), and 2,2′-bipyridyl (58.6 mg, 0.375 mmol) were dissolved in dehydrated THF (22.5 mL) in the glove box. After taking the flask out of the glove box, the solution was stirred at r.t. for 3.5 h. The reaction mixture was immediately filtered through a short pad of alumina column (eluent: $CHCl_3$), and the solvent was removed in vacuo. The residue was purified by silica gel column chromatography (Wakogel C-300; eluent: CH_2Cl_2/*n*-hexane = 1:2 and EtOAc/CH_2Cl_2/*n*-hexane = 1:24:75) and the solvent was removed under vacuum. The residue was purified with a recycling preparative HPLC (JAIGEL-1H-A and JAIGEL-2H-A; eluent: $CHCl_3$) and was recrystallized from CH_2Cl_2/MeOH to afford **1e** (32.33 mg, 0.0167 mmol, 11%) as a dark brown solid. R_f = 0.34 (5% EtOAc/*n*-hexane). ^{1}H NMR (600 MHz, $CDCl_3$, 20 °C): δ (ppm) 5.22 (s, 4H, Ar-CH), 3.61 (t, J = 6.6 Hz, 4H, OCH_2), 3.52 (t, J = 6.6 Hz, 8H, OCH_2), 2.39 (d, J = 4.2 Hz, 4H, β-CH), 1.95 (d, J = 4.2 Hz, 4H, β-CH), 1.57 (quin, J = 7.8 Hz, 8H, OCH_2CH_2), 1.48 (quin, J = 7.8 Hz, 4H, OCH_2CH_2), 1.28–1.17 (m, 156H, $OC_2H_4C_{13}H_{26}$), 0.90 (t, J = 7.2 Hz, 12H, CH_3), 0.88 (t, J = 7.2 Hz, 6H, CH_3). $^{13}C\{^1H\}$ NMR (151 MHz, $CDCl_3$, 20 °C): δ (ppm) 167.09, 158.55, 152.04, 146.61, 141.23, 130.89, 126.38, 114.57, 99.13, 73.34, 68.89, 32.12, 32.09, 30.24, 29.92, 29.90, 29.88, 29.86, 29.83, 29.78, 29.76, 29.58, 29.56, 29.54, 29.51, 29.27,

26.11, 26.02, 22.88, 22.85, 14.30, 14.28 (some signals of hexadecyl chains were overlapped). UV/vis (CH_2Cl_2, λ_{max}[nm] (ε, 10^4 $M^{-1}cm^{-1}$)): 426 (4.1), 528 (4.7). MALDI-TOF-MS: *m/z* (% intensity): 1933.7 (69), 1934.6 (100). Calcd for $C_{126}H_{210}N_4NiO_6$ ($[M]^+$): 1933.56.

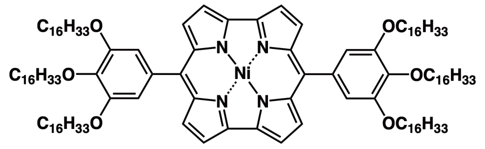

5,15-Bis(3,4,5-trioctyloxyphenyl)porphyrin, 2c′. According to literature procedures,[S8] a solution of di(pyrrol-2-yl)methane[S9] (500 mg, 3.42 mmol) and 3,4,5-trioctyloxybenzaldehyde[S6] (1.68 g, 3.42 mmol) in CH_2Cl_2 (600 mL) was stirred under N_2 with shielding from light. Trifluoroacetic acid (0.16 mL, 2.13 mmol) was added, and the solution was stirred at r.t. for 2.5 h. DDQ (1.17 g, 5.13 mmol) was added to the solution, and the resulting solution was stirred for an additional 2 h. After the reaction mixture was neutralized by triethylamine and was passed over alumina column, the solvent was removed. The residue was then chromatographed over a silica gel column (Wakogel C-300, eluent: 50% CH_2Cl_2/*n*-hexane) and was recrystallized from CH_2Cl_2/MeOH to give **2c′** (611 mg, 0.50 mmol, 29%) as a purple solid. R_f = 0.45 (50% CH_2Cl_2/*n*-hexane). ^{1}H NMR (600 MHz, $CDCl_3$, 20 °C): δ(ppm): 10.30 (s, 2H, *meso*-CH), 9.39 (d, J = 4.8 Hz, 4H, β-CH), 9.18 (d, J = 4.8 Hz, 4H, β-CH), 7.48 (s, 4H, Ar-CH), 4.32 (t, J = 7.2 Hz, 4H, OCH_2), 4.14 (t, J = 7.2 Hz, 8H, OCH_2), 2.00 (quin, J = 7.2 Hz, 4H, OCH_2CH_2), 1.90 (quin, J = 7.2 Hz, 8H, OCH_2CH_2), 1.69 (quin, J = 7.2 Hz, 4H, $OC_2H_4CH_2$), 1.53–1.24 (m, 56H, $OC_2H_4C_5H_{10}$), 0.95 (t, J = 7.2 Hz, 6H, CH_3), 0.85 (t, J = 7.2 Hz, 12H, CH_3), –3.12 (s, 2H, NH). $^{13}C\{^1H\}$ NMR (151 MHz, $CDCl_3$, 20 °C): δ(ppm) 151.66, 147.37, 145.37, 138.22, 136.46, 131.66, 131.26, 119.41, 114.68, 105.37, 73.96, 69.55, 32.18, 31.97, 30.77, 29.88, 29.70, 29.58, 29.44, 26.49, 26.32, 22.95, 22.81, 14.35, 14.24 (a signal of octyl chains was overlapped). UV/vis (CH_2Cl_2, λ_{max}[nm] (ε, 10^5 $M^{-1}cm^{-1}$)): 411 (3.6). MALDI-TOF-MS: *m/z* (% intensity): 1231.0 (100). Calcd for $C_{80}H_{118}N_4O_6$ ($[M]^+$): 1230.90.

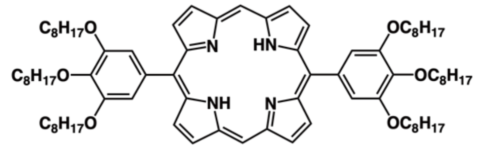

5,15-Bis(3,4,5-tridodecyloxyphenyl)porphyrin, 2d′. According to literature procedures,[S8] a solution of di(pyrrol-2-yl)methane[S9] (350 mg, 2.39 mmol) and 3,4,5-tridodecyloxybenzaldehyde[S6] (1.58 g, 2.39 mmol) in CH_2Cl_2 (420 mL) was stirred under N_2 with shielding from light. Trifluoroacetic acid (0.11 mL, 1.49 mmol) was added, and the solution was stirred at r.t. for 2 h. DDQ (0.82 g, 3.59 mmol) was added to the solution, and the resulting solution was stirred for an additional 20 min. After the reaction mixture was neutralized by triethylamine and was passed over alumina column, the solvent was removed. The residue was then chromatographed over a silica gel column (Wakogel C-300, eluent: 40% CH_2Cl_2/*n*-hexane) and was recrystallized from CH_2Cl_2/MeOH to give **2d′** (929 mg, 0.59 mmol, 49%) as a dark red solid. R_f = 0.40 (40% CH_2Cl_2/*n*-hexane). ^{1}H NMR (600 MHz, $CDCl_3$, 20 °C): δ(ppm): 10.30 (s, 2H, *meso*-CH), 9.39 (d, J = 4.8 Hz, 4H, β-CH), 9.18 (d, J = 4.8 Hz, 4H, β-CH), 7.48 (s, 4H, Ar-CH), 4.32 (t, J = 6.6 Hz, 4H, OCH_2), 4.14 (t, J = 6.6 Hz, 8H, OCH_2), 2.00 (quin, J = 7.2 Hz, 4H, OCH_2CH_2), 1.89 (quin, J = 7.2 Hz, 8H, OCH_2CH_2), 1.68 (quin, J = 7.2 Hz, 4H, $OC_2H_4CH_2$), 1.52–1.21 (m, 104H, $OC_2H_4C_9H_{18}$), 0.90 (t, J = 7.2 Hz, 6H, CH_3), 0.85 (t, J = 7.2 Hz, 12H, CH_3), –3.12 (s, 2H, NH). $^{13}C\{^1H\}$ NMR (151 MHz, $CDCl_3$, 20 °C): δ(ppm) 151.66, 147.36, 145.36, 138.24, 136.46, 131.66, 131.25, 119.41, 114.71, 105.36, 73.97, 69.57, 32.16, 32.06, 30.79, 30.06, 30.01, 29.95, 29.85, 29.80, 29.70, 29.64, 29.62, 29.50, 26.51, 26.33, 22.89, 22.83, 14.30, 14.25 (some signals of dodecyl chains were overlapped). UV/vis (CH_2Cl_2, λ_{max}[nm] (ε, 10^5 $M^{-1}cm^{-1}$)): 411 (3.6). MALDI-TOF-MS: *m/z* (% intensity): 1568.3 (100). Calcd for $C_{104}H_{166}N_4O_6$ ($[M]^+$): 1568.28.

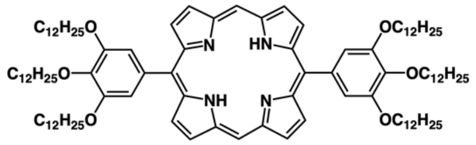

5,15-Bis(3,4,5-trihexadecyloxyphenyl)porphyrin, 2e′. According to literature procedures,[S8] a solution of di(pyrrol-2-yl)methane[S9] (114 mg, 0.78 mmol) and 3,4,5-trihexadecyloxybenzaldehyde[S6] (644 mg, 0.78 mmol) in dry CH_2Cl_2 (134 mL) was stirred under N_2 with shielding from light. Trifluoroacetic acid (37 μL, 0.48 mmol) was added, and the solution was stirred at r.t. for 2 h. DDQ (265 mg, 1.17 mmol) was added to the solution, and the resulting solution was stirred for an additional 2 h. After the reaction mixture was neutralized by triethylamine and was passed over alumina column, the solvent was removed. The residue was then chromatographed over a silica gel column (Wakogel C-300, eluent: 50% CH_2Cl_2/*n*-hexane) and was recrystallized from CH_2Cl_2/MeOH to give **2e′** (269 mg, 0.14 mmol, 36%) as a dark red solid. R_f = 0.25 (50% CH_2Cl_2/*n*-hexane). ^{1}H NMR (600 MHz, $CDCl_3$, 20 °C): δ(ppm): 10.30 (s, 2H, *meso*-CH), 9.38 (d, J = 4.2 Hz, 4H, β-CH), 9.18 (d, J = 4.2 Hz, 4H, β-CH), 7.48 (s, 4H, Ar-CH), 4.32 (t, J = 6.6 Hz, 4H, OCH_2), 4.14 (t, J = 6.6 Hz, 8H, OCH_2), 1.98 (quin, J = 7.2 Hz, 4H, OCH_2CH_2), 1.89 (quin, J = 7.4 Hz, 8H, OCH_2CH_2), 1.68 (quin, J = 7.2 Hz, 4H, $OC_2H_4CH_2$), 1.54–1.22 (m, 152H, $OC_2H_4C_{13}H_{26}$), 0.88 (t, J = 7.2 Hz, 6H, CH_3), 0.86 (t, J = 7.2 Hz, 12H, CH_3), –3.13 (s, 2H, NH). $^{13}C\{^1H\}$ NMR (151 MHz, $CDCl_3$, 20 °C): δ(ppm) 151.66, 147.39, 145.36, 138.24, 136.46, 131.64, 131.25, 119.41, 114.71, 105.36, 73.95, 69.57, 32.11, 32.07, 30.79, 30.06, 30.03, 30.00, 29.97, 29.95, 29.86, 29.80, 29.71, 29.65, 29.56, 29.51, 26.51, 26.33, 22.87, 22.84, 14.29, 14.27 (some signals of hexadecyl chains were overlapped). UV/vis (CH_2Cl_2, λ_{max}[nm] (ε, 10^5 $M^{-1}cm^{-1}$)): 411 (3.7). MALDI-TOF-MS: *m/z* (% intensity): 1904.6 (100). Calcd for $C_{128}H_{214}N_4O_6$ ($[M]^+$): 1904.66.

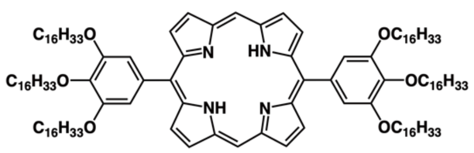

Ni^{II} complex of 5,15-bis(3,4,5-trioctyloxyphenyl)porphyrin, 2c. To a DMF (42 mL) solution of **2c′** (80 mg, 0.065 mmol) as added Ni(acac)$_2$·nH$_2$O (250 mg), and the mixture was refluxed for 1 h. The mixture was cooled to r.t., was diluted with *n*-hexane, and was washed with water. The aqueous phase was extracted with *n*-hexane. Combined organic phase was washed with water and brine, was dried over anhydrous Na_2SO_4, and was evaporated. The residue was then chromatographed over a silica gel column (Wakogel C-300, eluent: 50% CH_2Cl_2/*n*-hexane) to give **2c** (75 mg, 0.058 mmol, 90%) as a red solid. R_f = 0.51 (50% CH_2Cl_2/*n*-hexane). ^{1}H NMR (600 MHz, $CDCl_3$, 20 °C): δ (ppm): 9.94 (s, 2H, *meso*-CH), 9.18 (d, J = 4.8 Hz, 4H, β-CH), 9.04 (d, J = 4.8 Hz, 4H, β-CH), 7.28 (s, 4H, Ar-CH), 4.27 (t, J = 6.6 Hz, 4H, OCH$_2$), 4.07 (t, J = 6.6 Hz, 8H, OCH$_2$), 1.96 (quin, J = 7.2 Hz, 4H, OCH$_2$*CH$_2$*), 1.86 (quin, J = 7.2 Hz, 8H, OCH$_2$*CH$_2$*), 1.66 (quin, J = 7.2 Hz, 4H, OC$_2$H$_4$*CH$_2$*), 1.51–1.23 (m, 56H, OC$_2$H$_4$*C$_5$H$_{10}$*), 0.94 (t, J = 7.2 Hz, 6H, CH$_3$), 0.85 (t, J = 7.2 Hz, 12H, CH$_3$). ^{13}C{^{1}H} NMR (151 MHz, $CDCl_3$, 20 °C): δ (ppm) 151.55, 143.12, 142.08, 138.08, 136.11, 132.64, 132.06, 118.69, 113.66, 105.26, 73.90, 69.46, 32.17, 31.96, 30.73, 29.85, 29.66, 29.63, 29.55, 29.43, 26.46, 26.28, 22.94, 22.80, 14.33, 14.24. UV/vis (CH_2Cl_2, λ_{max}[nm] (ε, 10^5 $M^{-1}cm^{-1}$)): 412 (2.2). MALDI-TOF-MS: *m*/*z* (% intensity): 1287.1 (100). Calcd for $C_{80}H_{118}N_4O_6Ni$ ([M]$^+$): 1286.82.

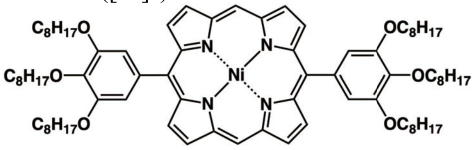

Ni^{II} complex of 5,15-bis(3,4,5-tridodecyloxyphenyl)porphyrin, 2d. To a DMF (33 mL) solution of **2d′** (80 mg, 0.051 mmol) was added Ni(acac)$_2$·nH$_2$O (197 mg), and the mixture was refluxed for 1 h. The mixture was cooled to r.t., was diluted with *n*-hexane, and was washed with water. The aqueous phase was extracted with *n*-hexane. Combined organic phase was washed with water and brine, was dried over anhydrous Na_2SO_4, and was evaporated. The residue was then chromatographed over a silica gel column (Wakogel C-300, eluent: 50% CH_2Cl_2/*n*-hexane) to give **2d** (73.9 mg, 0.045 mmol, 89%) as a red solid. R_f = 0.67 (50% CH_2Cl_2/*n*-hexane). ^{1}H NMR (600 MHz, $CDCl_3$, 20 °C): δ (ppm): 9.94 (s, 2H, *meso*-CH), 9.18 (d, J = 4.2 Hz, 4H, β-CH), 9.04 (d, J = 4.8 Hz, 4H, β-CH), 7.28 (s, 4H, Ar-CH), 4.27 (t, J = 6.6 Hz, 4H, OCH$_2$), 4.07 (t, J = 6.6 Hz, 8H, OCH$_2$), 1.96 (quin, J = 7.2 Hz, 4H, OCH$_2$*CH$_2$*), 1.86 (quin, J = 7.2 Hz, 8H, OCH$_2$*CH$_2$*), 1.65 (quin, J = 7.8 Hz, 4H, OC$_2$H$_4$*CH$_2$*), 1.54–1.22 (m, 104H, OC$_2$H$_4$*C$_9$H$_{18}$*), 0.90 (t, J = 7.2 Hz, 6H, CH$_3$), 0.85 (t, J = 7.2 Hz, 12H, CH$_3$). ^{13}C{^{1}H} NMR (151 MHz, $CDCl_3$, 20 °C): δ (ppm) 151.55, 143.11, 142.79, 138.08, 136.10, 132.66, 132.06, 118.70, 113.66, 105.25, 73.90, 69.45, 32.14, 32.05, 30.73, 30.03, 30.00, 29.92, 29.83, 29.78, 29.63, 29.61, 29.50, 26.47, 26.29, 22.89, 22.82, 14.30, 14.25 (some signals of dodecyl chains were overlapped). UV/vis (CH_2Cl_2, λ_{max}[nm] (ε, 10^5 $M^{-1}cm^{-1}$)): 405 (2.3). MALDI-TOF-MS: *m*/*z* (% intensity): 1624.4 (100). Calcd for $C_{104}H_{164}N_4O_6Ni$ ([M]$^+$): 1624.21.

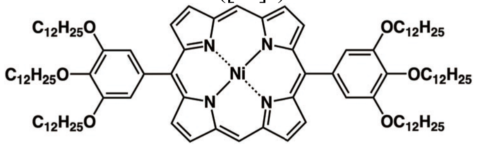

Ni^{II} complex of 5,15-bis(3,4,5-trihexadecyloxyphenyl)porphyrin, 2e. To a DMF (14 mL) solution of **2e′** (40 mg, 0.021 mmol) was added Ni(acac)$_2$·nH$_2$O (81 mg), and the mixture was refluxed for 1 h. The mixture was cooled to r.t., was diluted with *n*-hexane, and was washed with water. The aqueous phase was extracted with *n*-hexane. Combined organic phase was washed with water and brine, was dried over anhydrous Na_2SO_4, and was evaporated. The residue was then chromatographed over a silica gel column (Wakogel C-300, eluent: 50% CH_2Cl_2/*n*-hexane) to give **2e** (35.6 mg, 0.018 mmol, 86%) as a red solid. R_f = 0.63 (50% CH_2Cl_2/*n*-hexane). ^{1}H NMR (600 MHz, $CDCl_3$, 20 °C): δ (ppm): 9.94 (s, 2H, *meso*-CH), 9.18 (d, J = 4.8 Hz, 4H, β-CH), 9.04 (d, J = 4.8 Hz, 4H, β-CH), 7.28 (s, 4H, Ar-CH), 4.27 (t, J = 6.6 Hz, 4H, OCH$_2$), 4.07 (t, J = 6.6 Hz, 8H, OCH$_2$), 1.96 (quin, J = 7.4 Hz, 4H, OCH$_2$*CH$_2$*), 1.86 (quin, J = 7.2 Hz, 8H, OCH$_2$*CH$_2$*), 1.65 (quin, J = 6.6 Hz, 4H, OC$_2$H$_4$*CH$_2$*), 1.50–1.22 (m, 152H, OC$_2$H$_4$*C$_{13}$H$_{26}$*), 0.88 (t, J = 7.2 Hz, 6H, CH$_3$), 0.86 (t, J = 7.2 Hz, 12H, CH$_3$). ^{13}C{^{1}H} NMR (151 MHz, $CDCl_3$, 20 °C): δ (ppm) 151.55, 143.12, 142.80, 138.08, 136.11, 132.66, 132.06, 118.70, 113.67, 105.26, 73.89, 69.47, 32.11, 32.07, 30.73, 30.04, 30.01, 29.96, 29.94, 29.92, 29.85, 29.80, 29.64, 29.62, 29.55, 29.51, 26.48, 26.30, 22.87, 22.83, 14.27 (some signals of hexadecyl chains were overlapped). UV/vis (CH_2Cl_2, λ_{max}[nm] (ε, 10^5 $M^{-1}cm^{-1}$)): 405 (2.2). MALDI-TOF-MS: *m*/*z* (% intensity): 1960.6 (100). Calcd for $C_{128}H_{212}N_4O_6Ni$ ([M]$^+$): 1960.63.

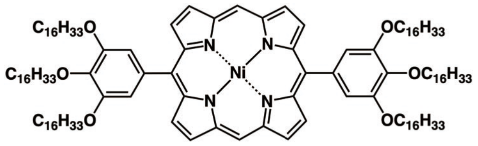

[S1] S. Yorsaeng, Y. Kato, K. Tsutsumi, A. Inagaki, B. Kitiyanan, M. Fujiki and K. Nomura, *Chem. Eur. J.*, 2015, **21**, 16764–16768.

[S2] K. D. Katariya, S. R. Shah and D. Reddy, *Bioorg. Chem.*, 2020, **94**, 103406.

[S3] S. Liu, H. Tanaka, R. Nozawa, N. Fukui and H. Shinokubo, *Chem. Eur. J.*, 2019, **25**, 7618–7622.

[S4] H. Ke, H. Wang, W.-K. Wong, N.-K. Mak, D. W. J. Kwong, K.-L. Wong and H.-L. Tam, *Chem. Commun.*, 2010, **46**, 6678–6680.

[S5] R. A. Krüger, A. S. Terpstra and T. C. Sutherland, *Can. J. Chem.*, 2011, **89**, 214–220.

[S6] F. Cheng and A. Adronov, *Chem. Eur. J.*, 2006, **12**, 5053–5059.

[S7] R. Nozawa, H. Tanaka, W.-Y. Cha, Y. Hong, I. Hisaki, S. Shimizu, J.-Y. Shin, T. Kowalczyk, S. Irle, D. Kim and H. Shinokubo, *Nat. Commun.*, 2016, **7**, 13620.
[S8] N. Aratani, A. Takagi, Y. Yanagawa, T. Matsumoto, T. Kawai, Z. S. Yoon, D. Kim and A. Osuka, *Chem. Eur. J.*, 2005*,* **11**, 3389–3404.
[S9] J.-W. Ka and C.-H. Lee, *Tetrahedron Lett.*, 2000, **41**, 4609–4613.

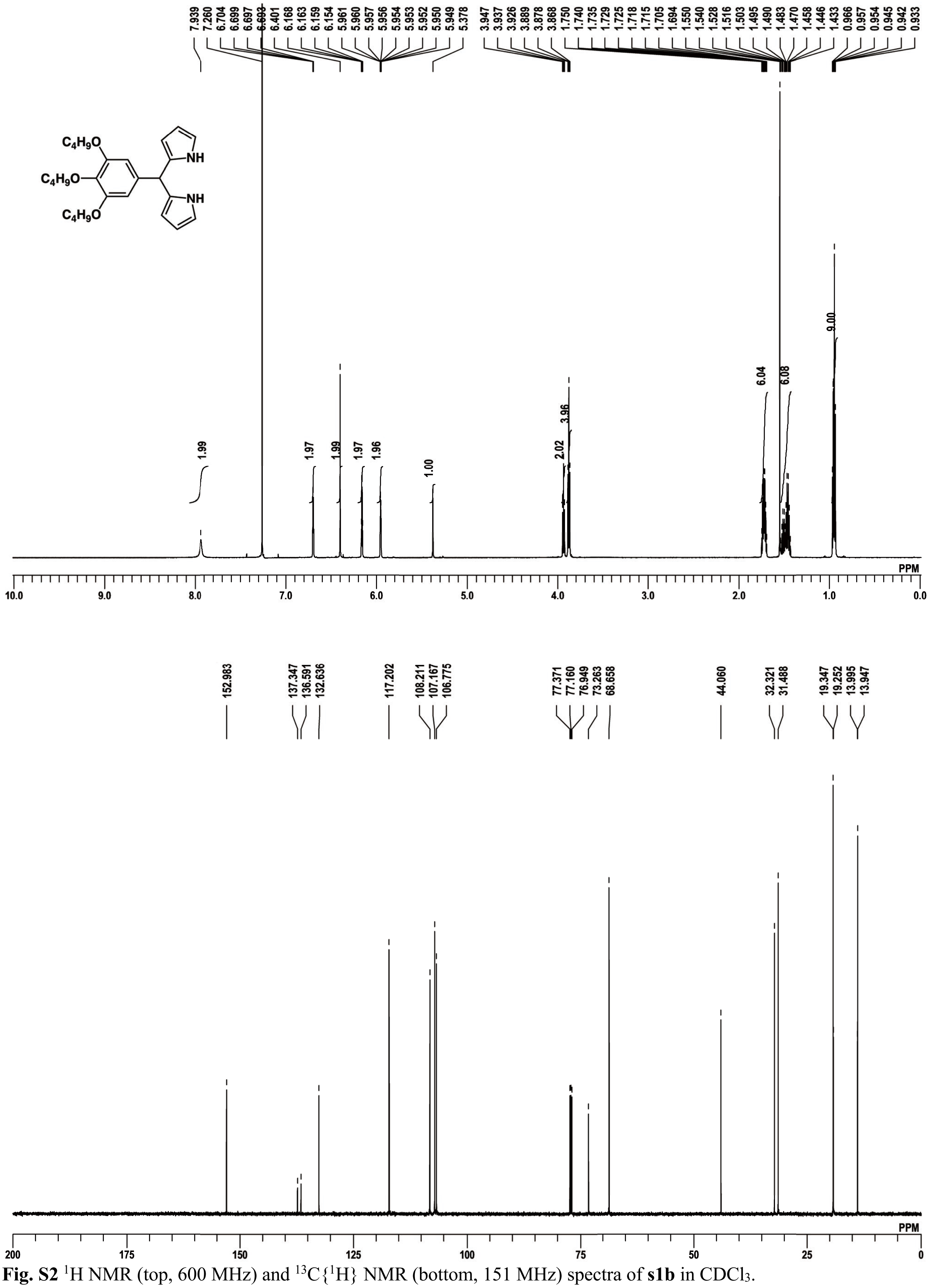

Fig. S2 ^{1}H NMR (top, 600 MHz) and $^{13}C\{^{1}H\}$ NMR (bottom, 151 MHz) spectra of **s1b** in $CDCl_3$.

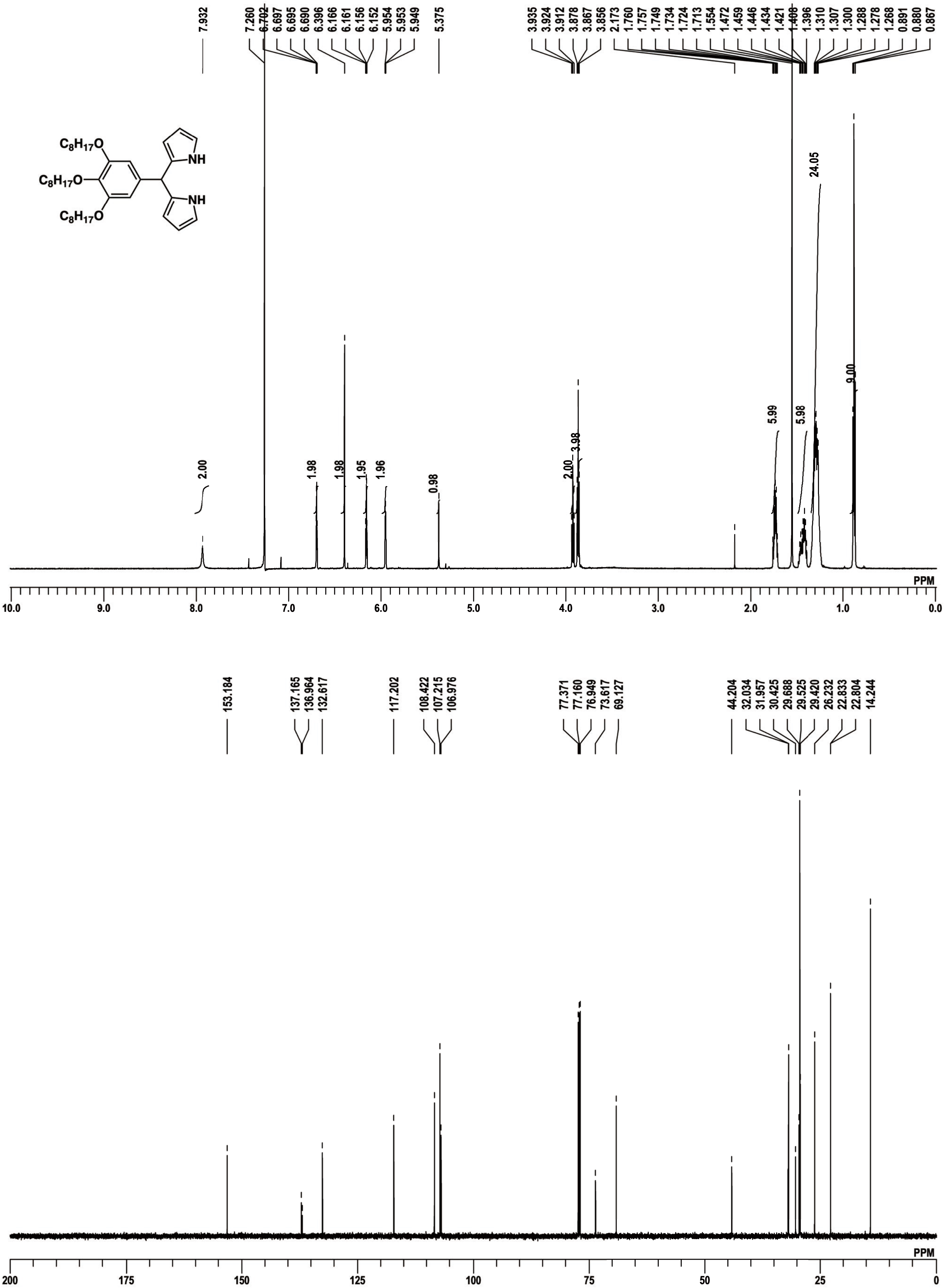

Fig. S3 ^{1}H NMR (top, 600 MHz) and $^{13}C\{^{1}H\}$ NMR (bottom, 151 MHz) spectra of **s1c** in $CDCl_3$.

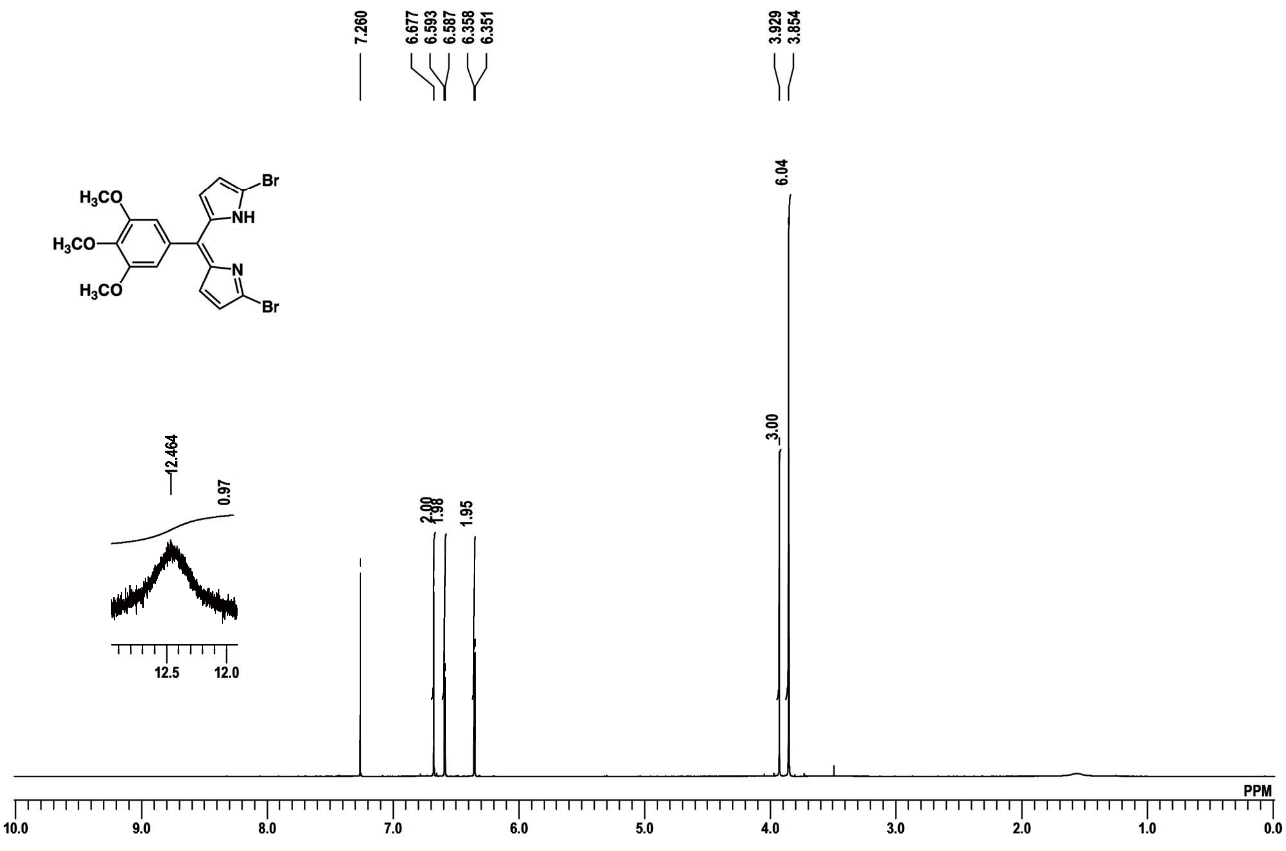

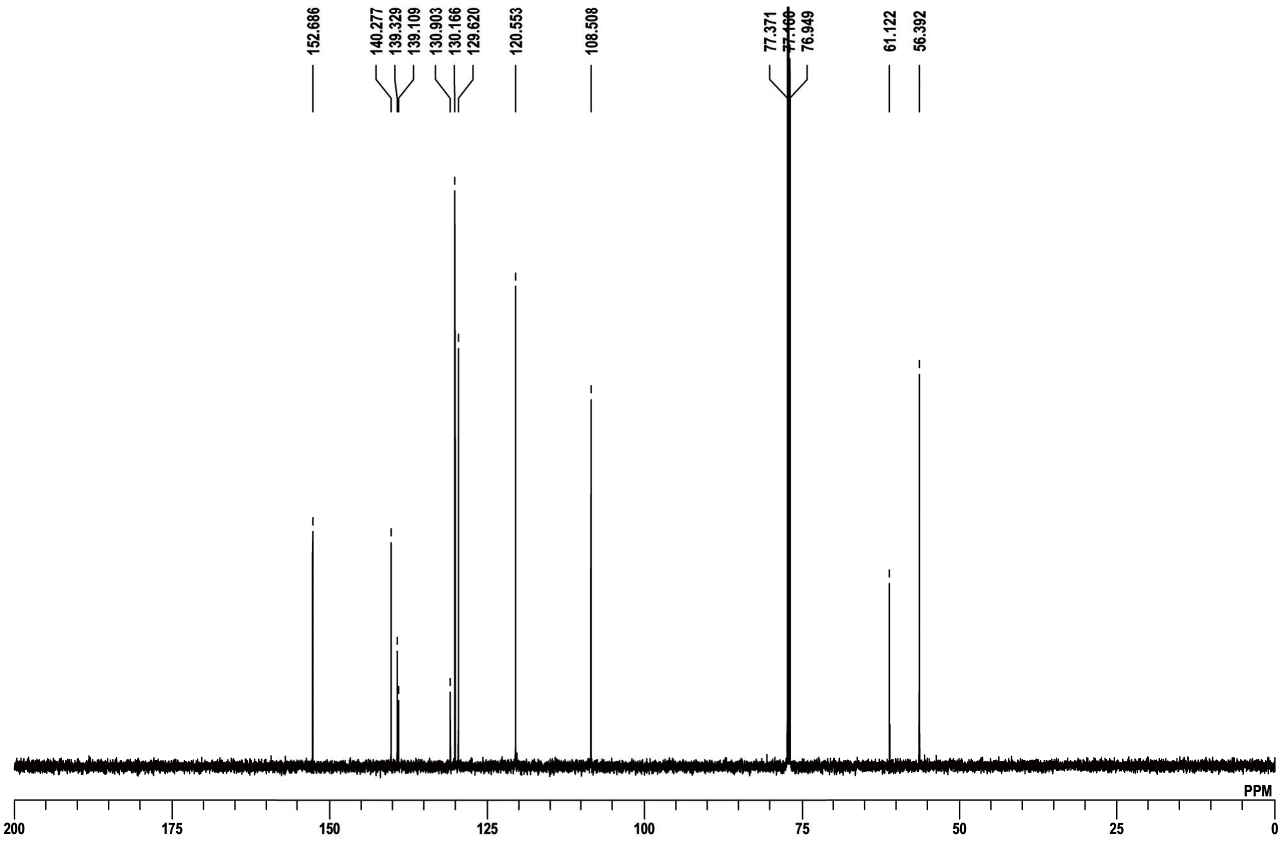

Fig. S4 ^{1}H NMR (top, 600 MHz) and ^{13}C{^{1}H} NMR (bottom, 151 MHz) spectra of **s2a** in $CDCl_3$.

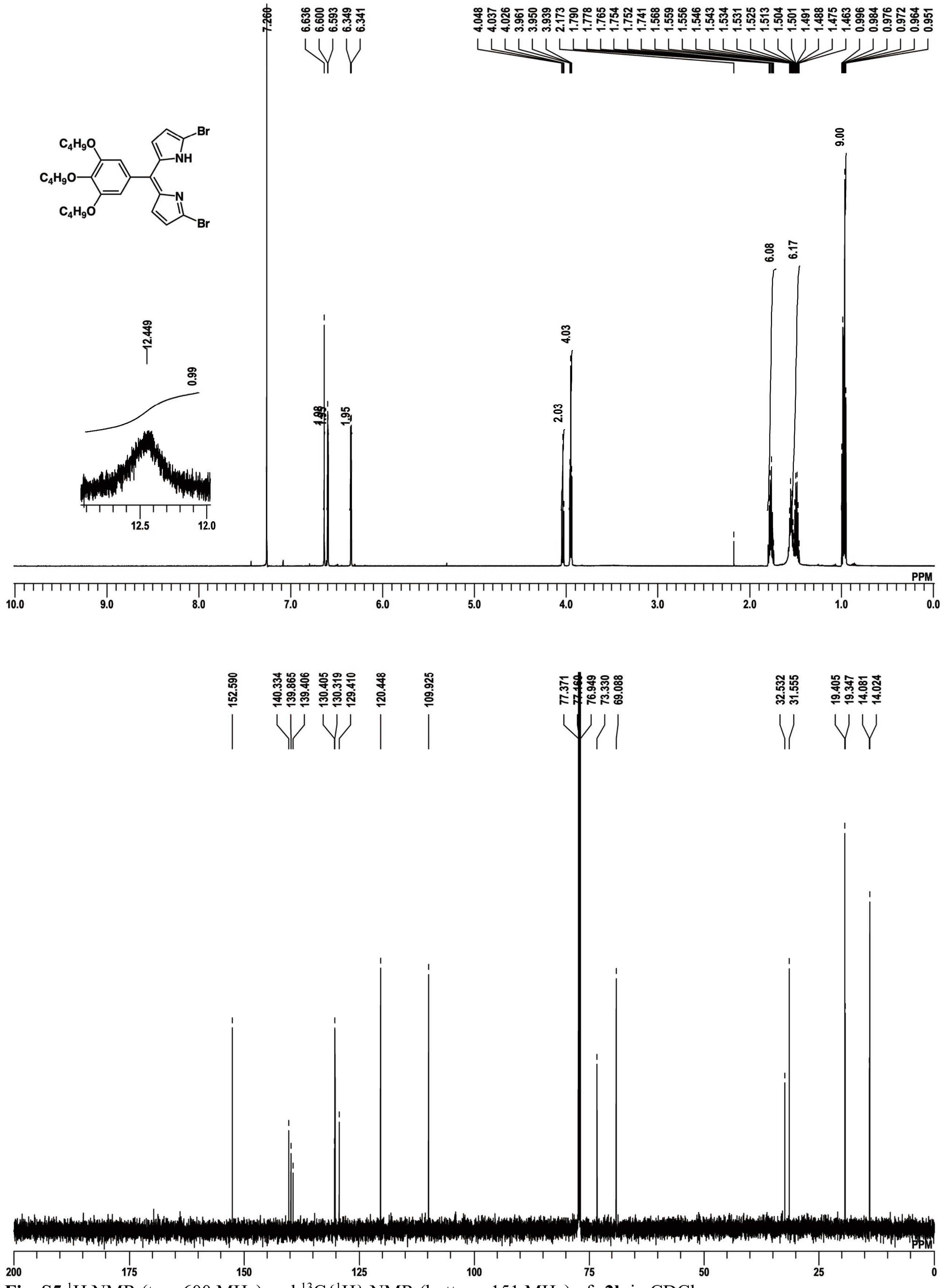

Fig. S5 ^{1}H NMR (top, 600 MHz) and ^{13}C{^{1}H} NMR (bottom, 151 MHz) of **s2b** in $CDCl_3$.

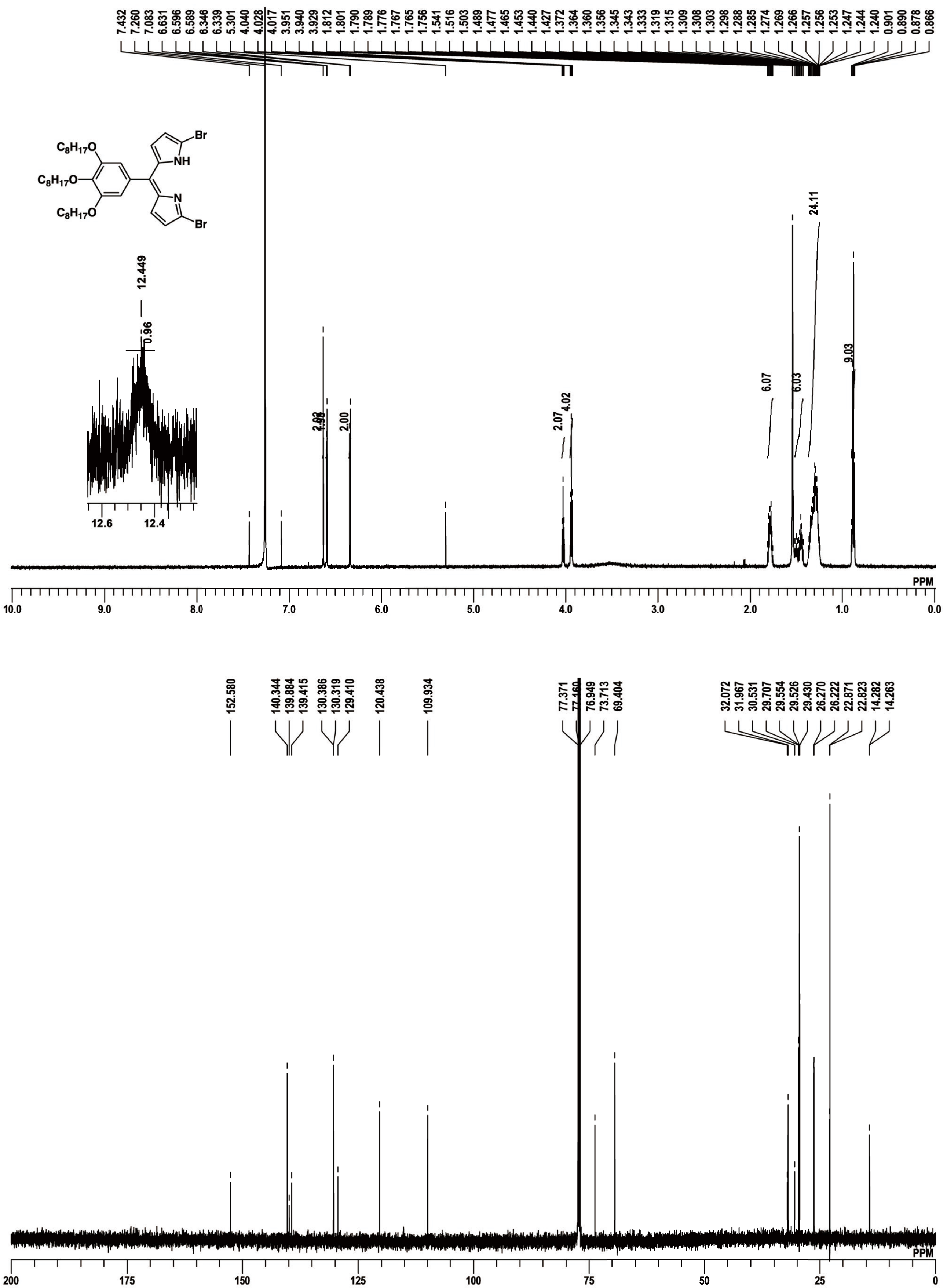

Fig. S6 ^{1}H NMR (top, 600 MHz) and ^{13}C{^{1}H} NMR (bottom, 151 MHz) spectra of **s2c** in $CDCl_3$.

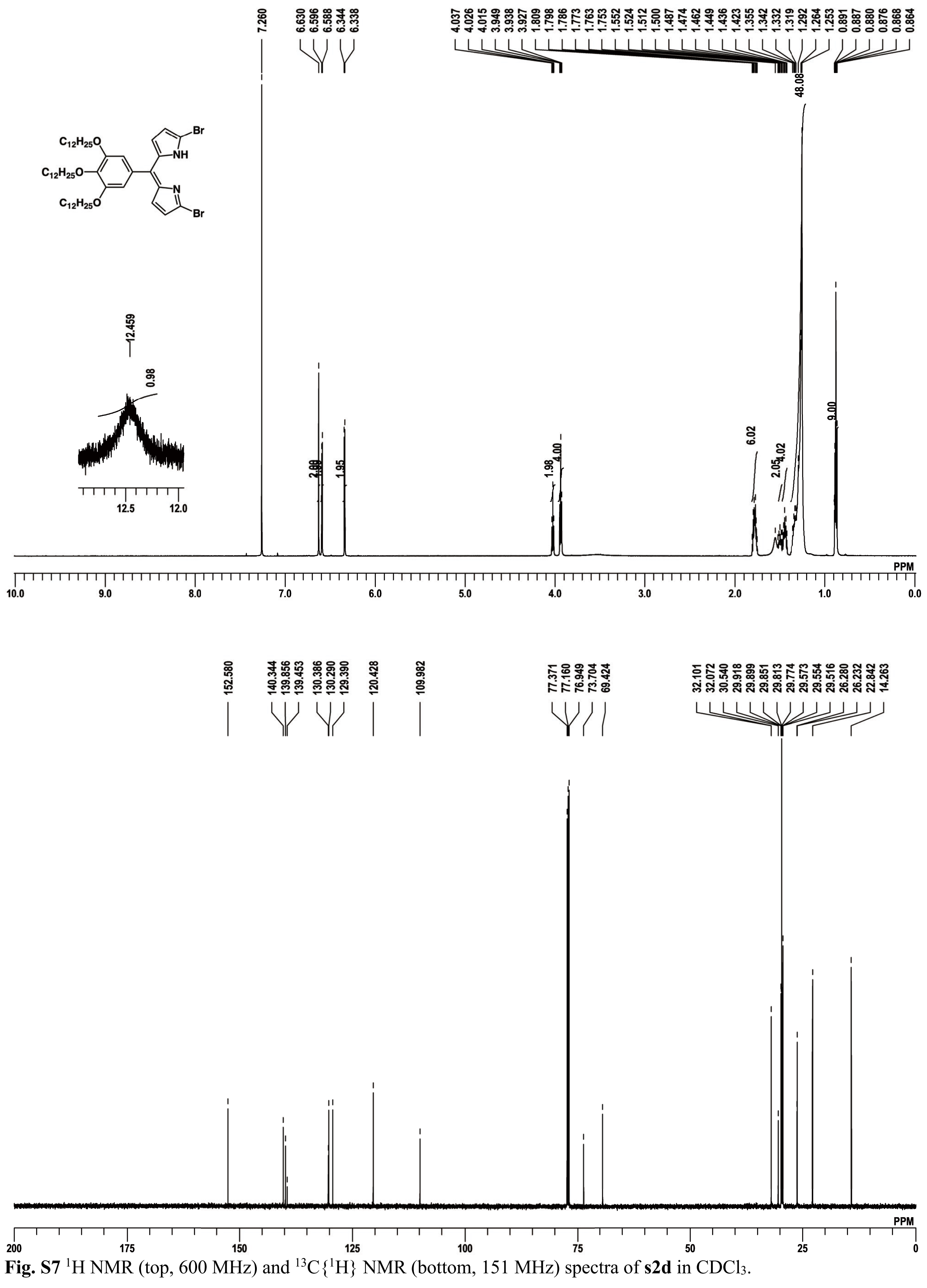

Fig. S7 ^{1}H NMR (top, 600 MHz) and $^{13}C\{^{1}H\}$ NMR (bottom, 151 MHz) spectra of **s2d** in $CDCl_3$.

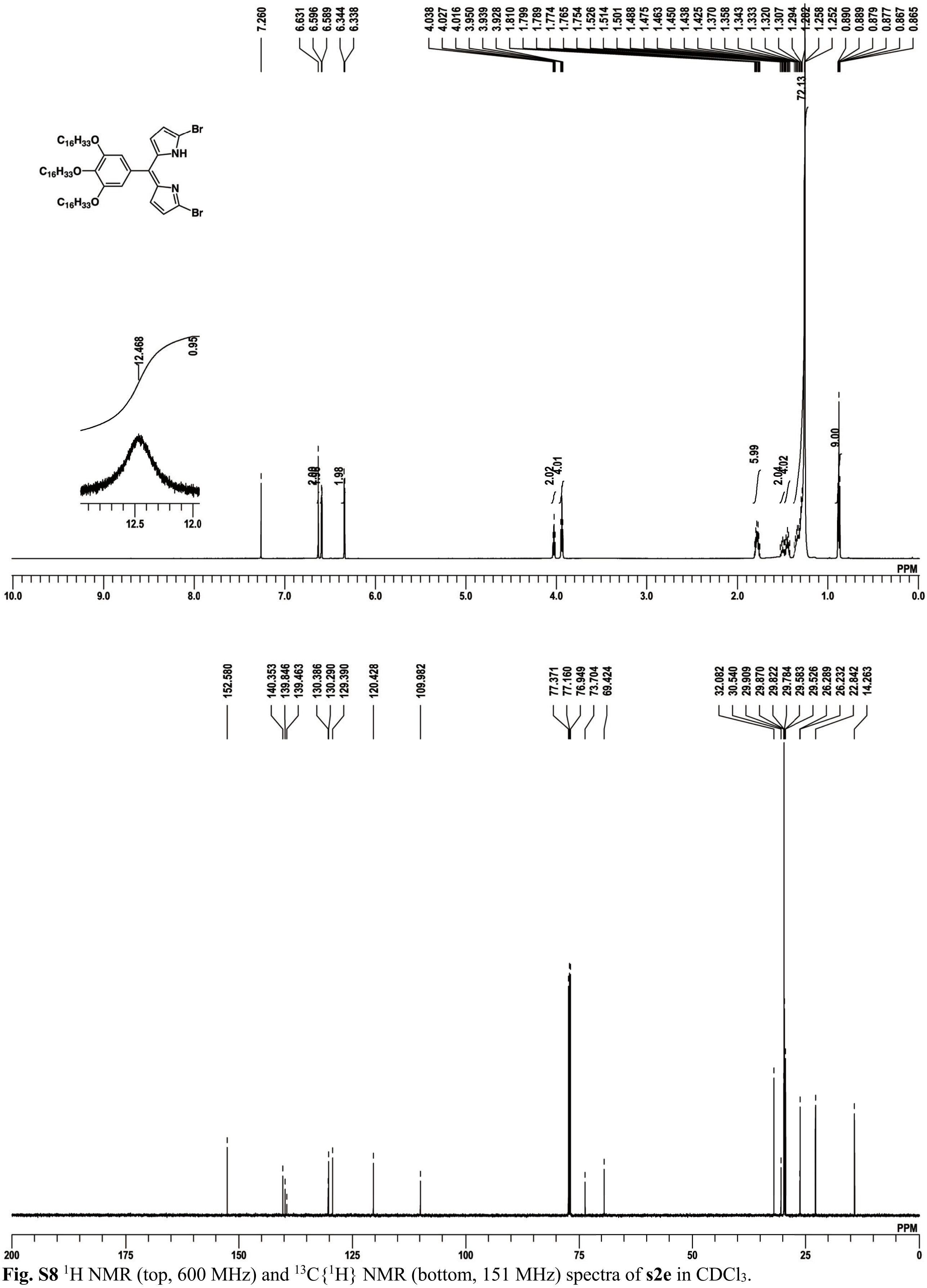

Fig. S8 ^{1}H NMR (top, 600 MHz) and $^{13}C\{^1H\}$ NMR (bottom, 151 MHz) spectra of **s2e** in $CDCl_3$.

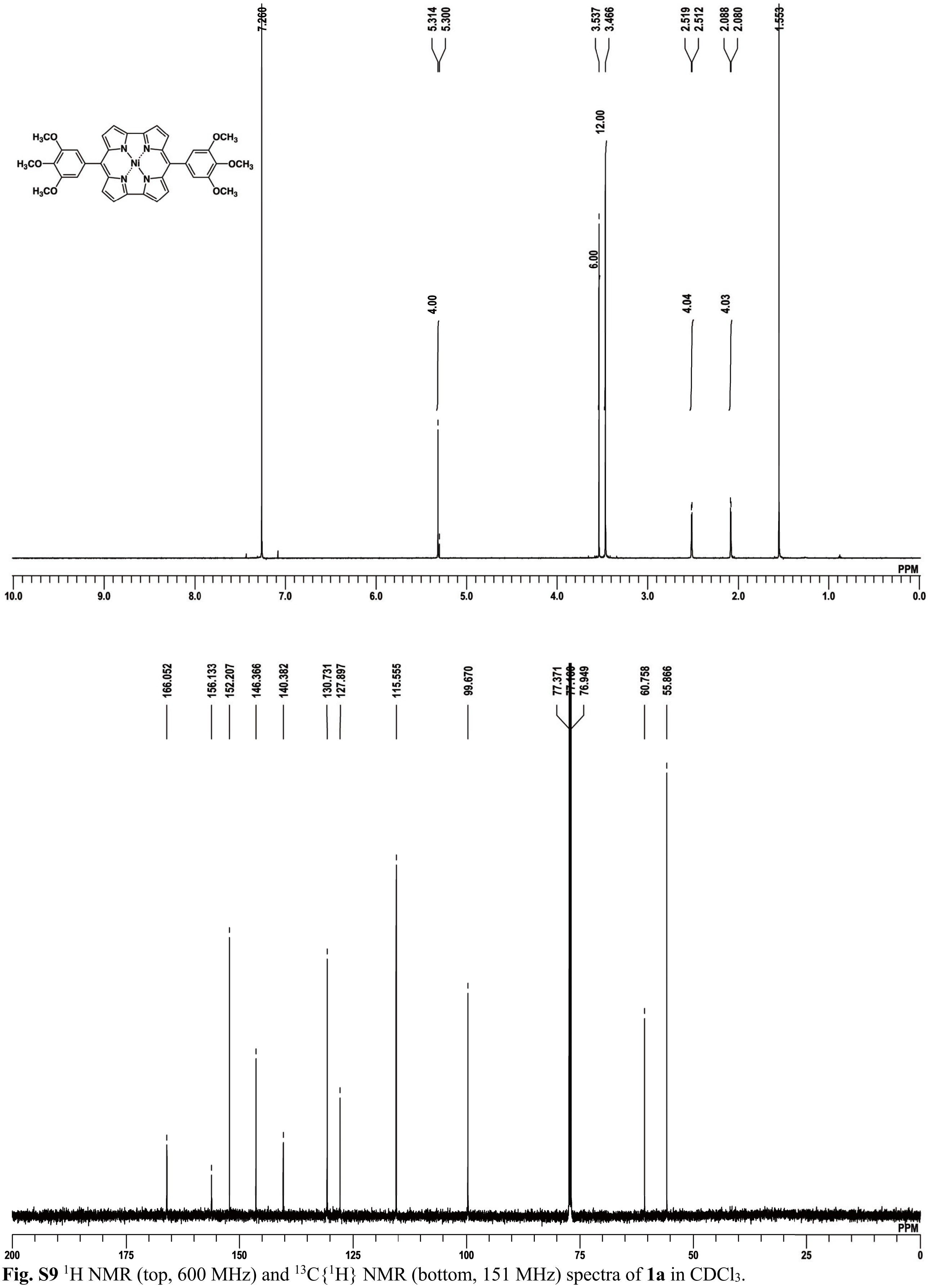

Fig. S9 ^{1}H NMR (top, 600 MHz) and ^{13}C{^{1}H} NMR (bottom, 151 MHz) spectra of **1a** in $CDCl_3$.

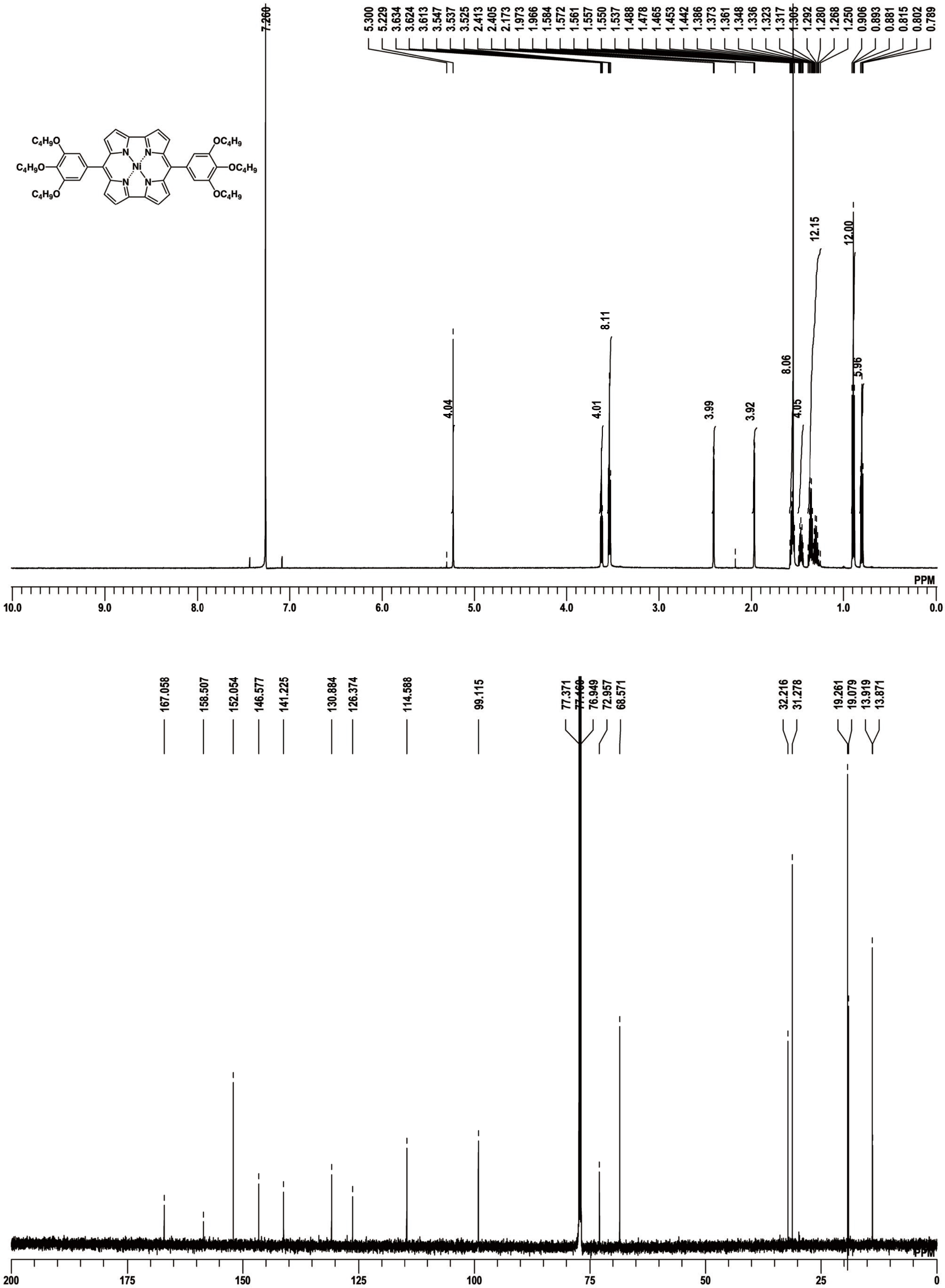

Fig. S10 ^{1}H NMR (top, 600 MHz) and $^{13}C\{^{1}H\}$ NMR (bottom, 151 MHz) spectra of **1b** in $CDCl_3$.

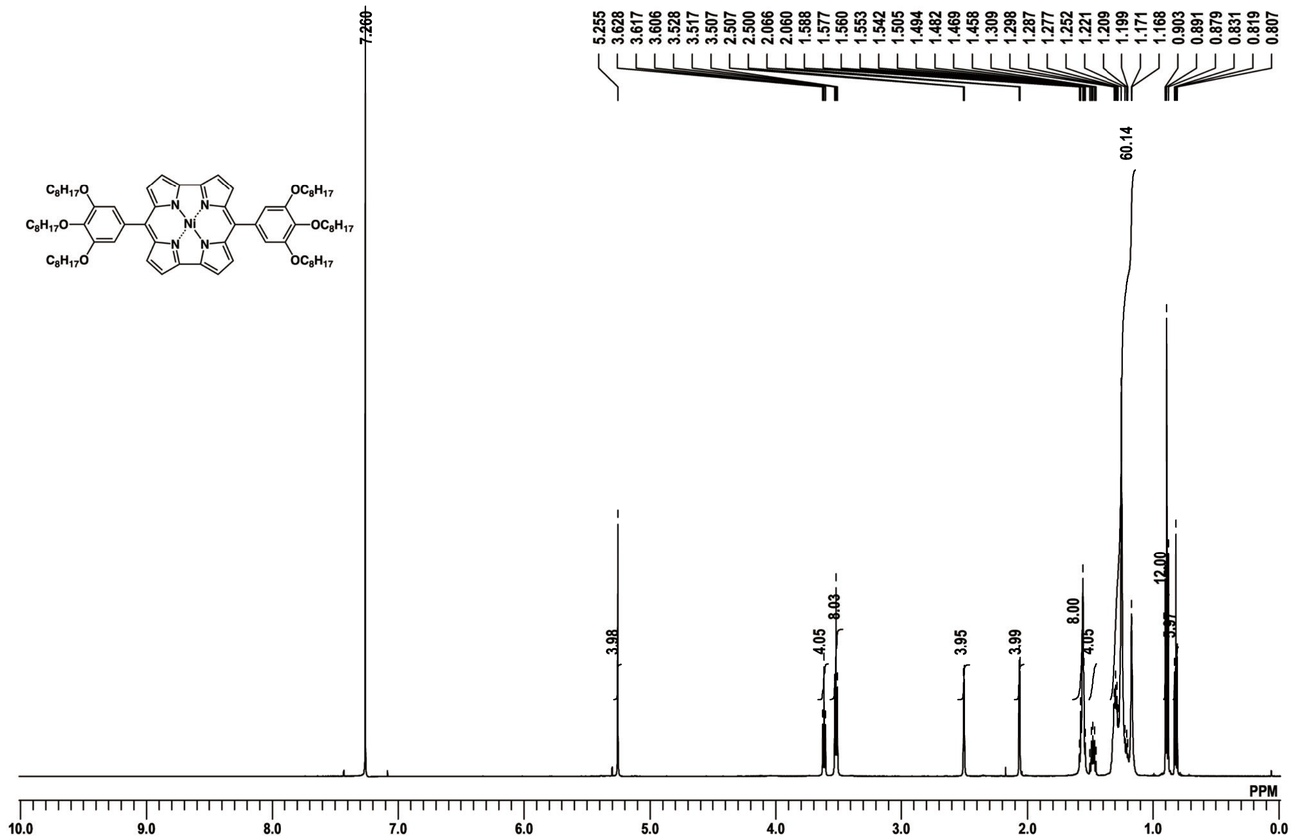

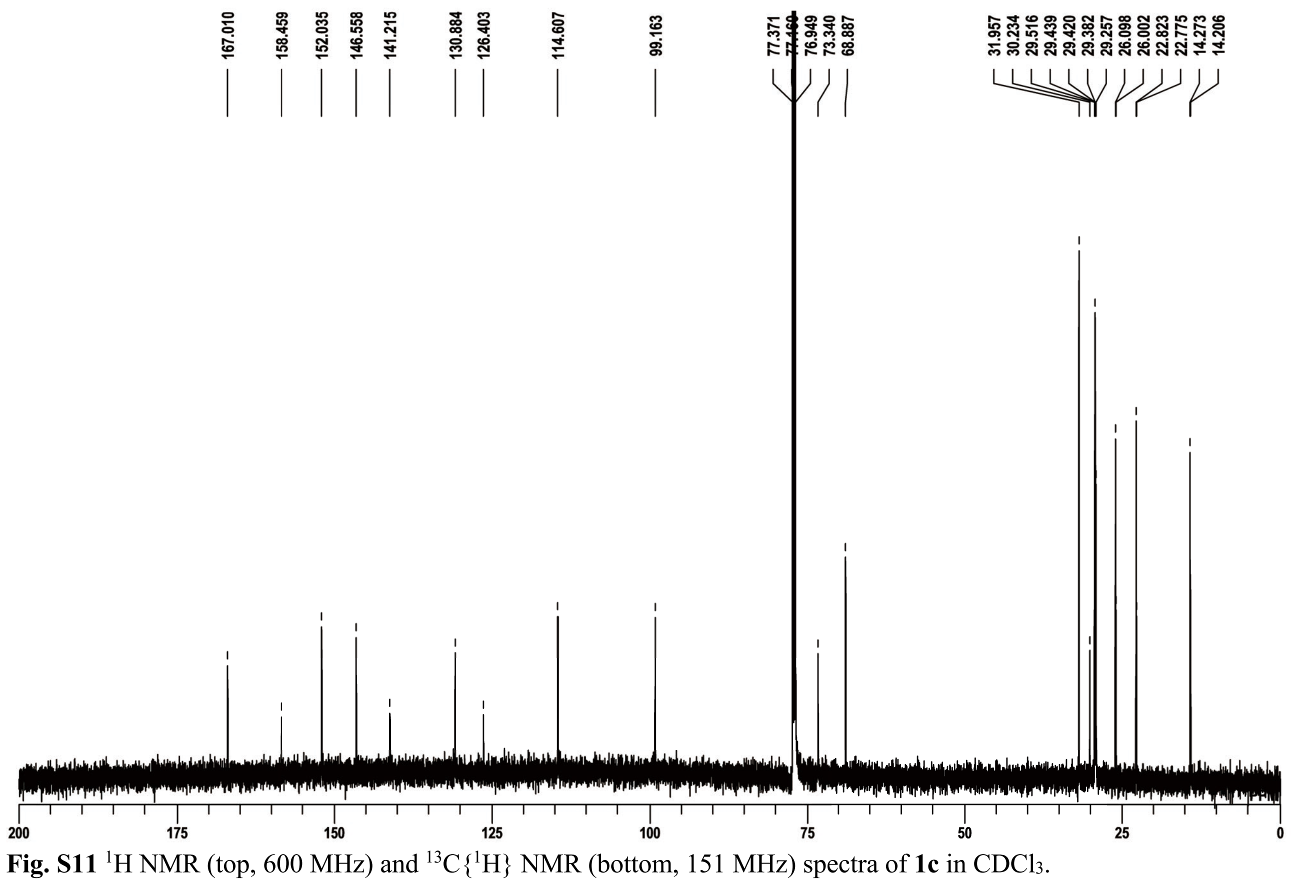

Fig. S11 ^{1}H NMR (top, 600 MHz) and $^{13}C\{^{1}H\}$ NMR (bottom, 151 MHz) spectra of **1c** in $CDCl_3$.

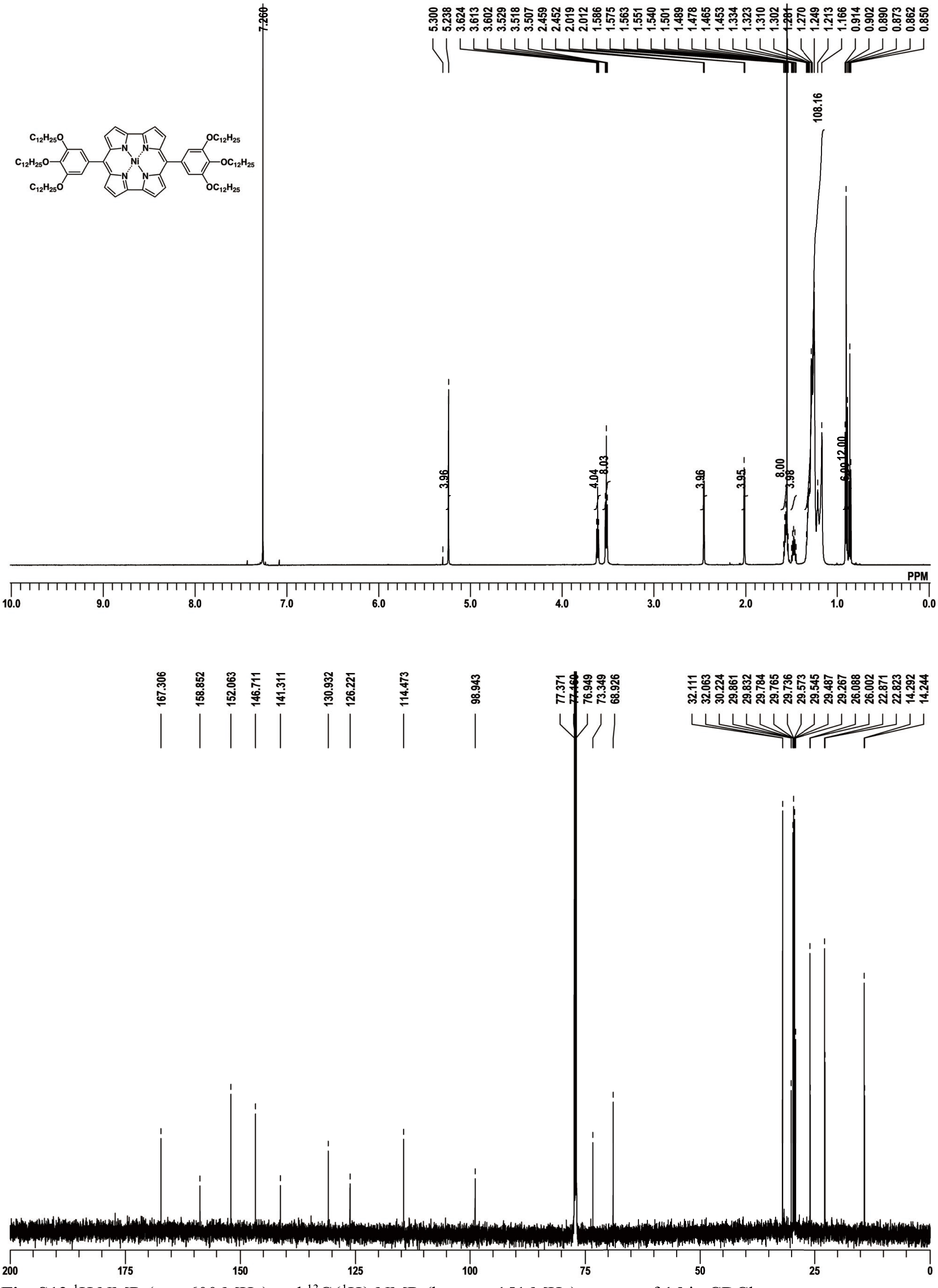

Fig. S12 ^{1}H NMR (top, 600 MHz) and $^{13}C\{^{1}H\}$ NMR (bottom, 151 MHz) spectra of **1d** in $CDCl_3$.

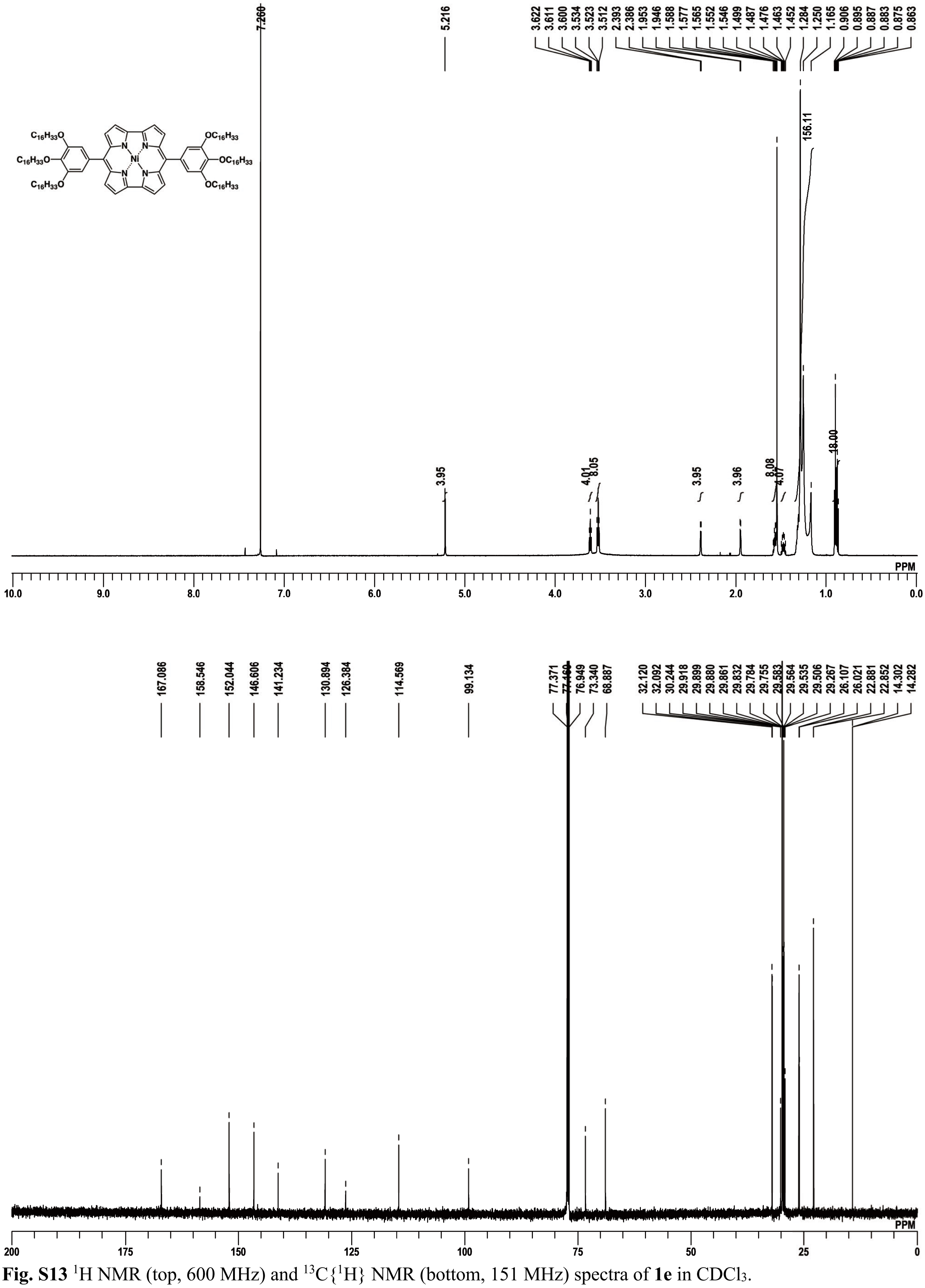

Fig. S13 ^{1}H NMR (top, 600 MHz) and $^{13}C\{^{1}H\}$ NMR (bottom, 151 MHz) spectra of **1e** in $CDCl_3$.

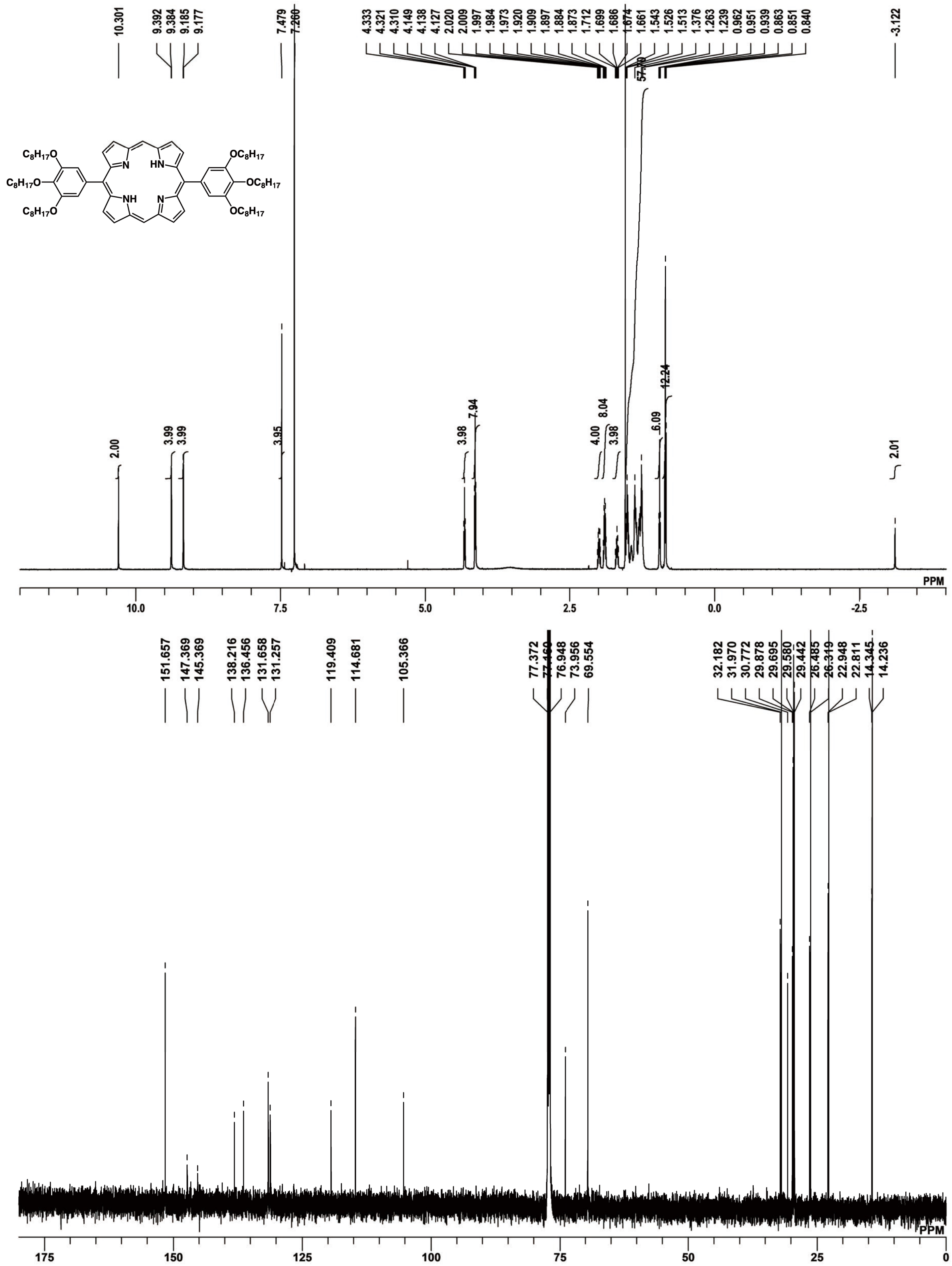

Fig. S14 ^{1}H NMR (top, 600 MHz) and $^{13}C\{^{1}H\}$ NMR (bottom, 151 MHz) spectra of **2c′** in $CDCl_3$ at 20 °C.

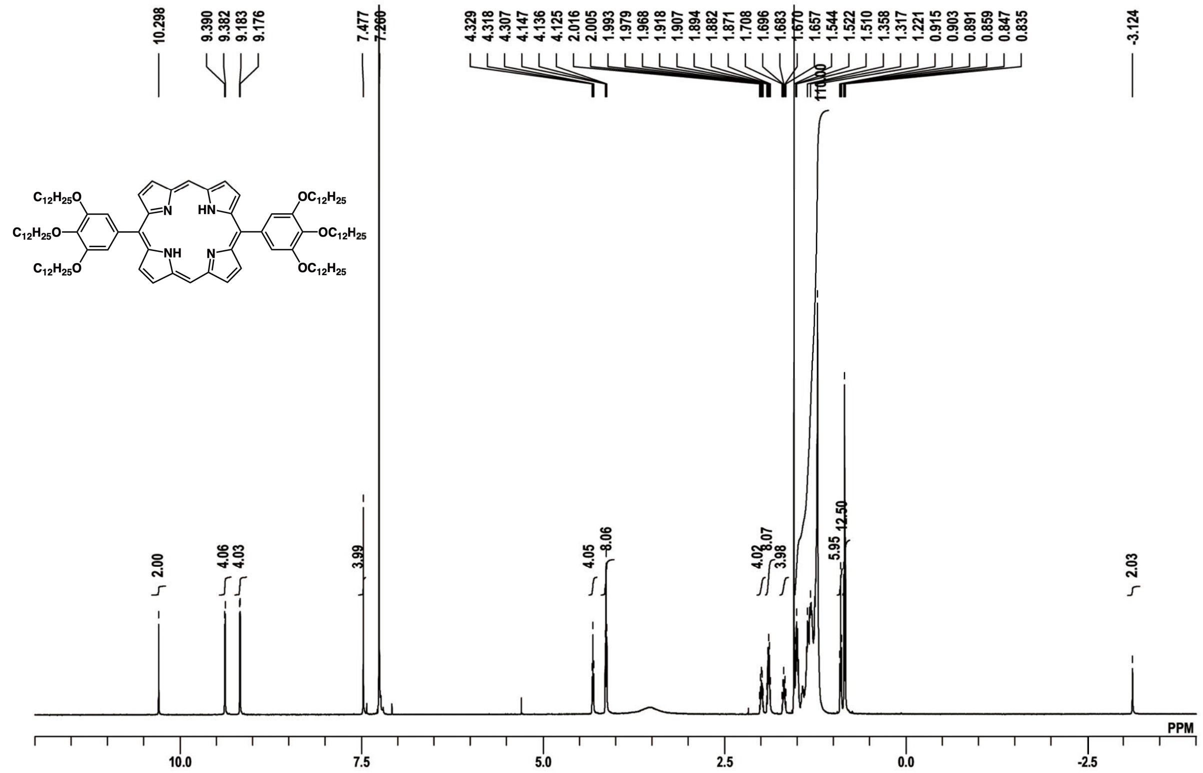

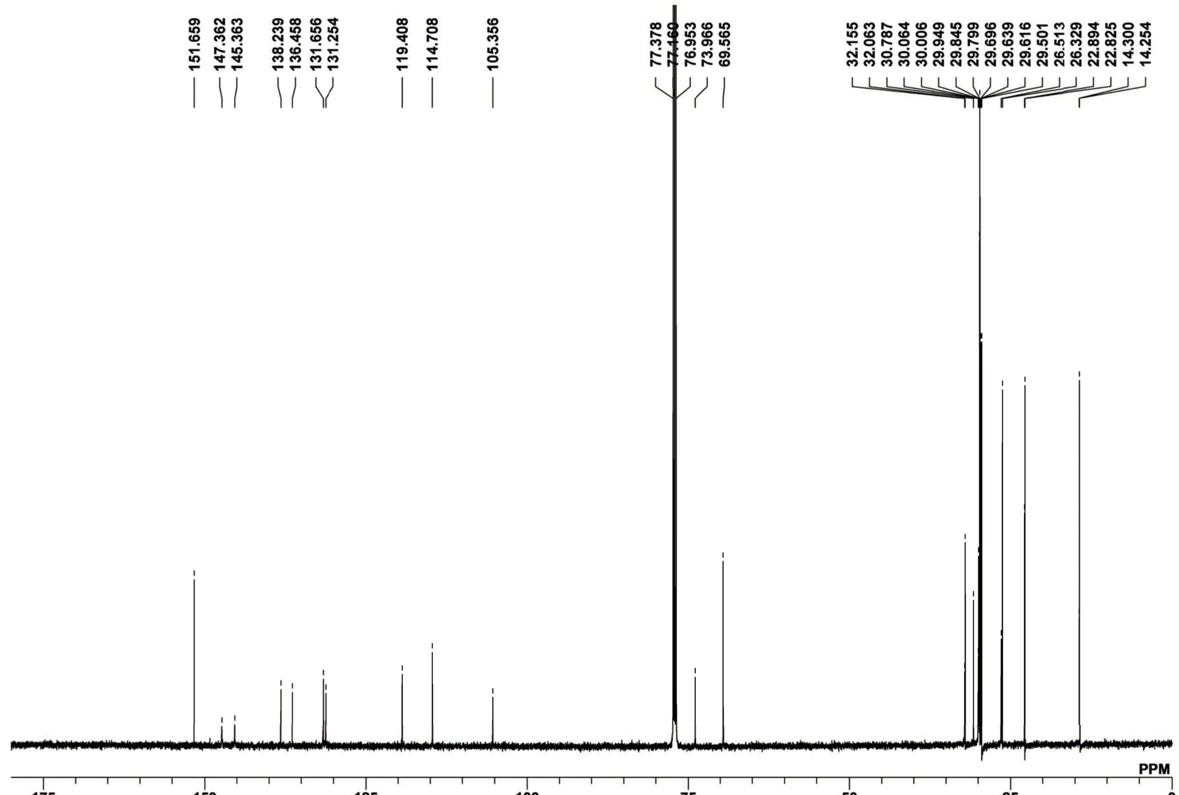

Fig. S15 ^{1}H NMR (top, 600 MHz) and $^{13}C\{^{1}H\}$ NMR (bottom, 151 MHz) spectra of **2d´** in $CDCl_3$ at 20 °C.

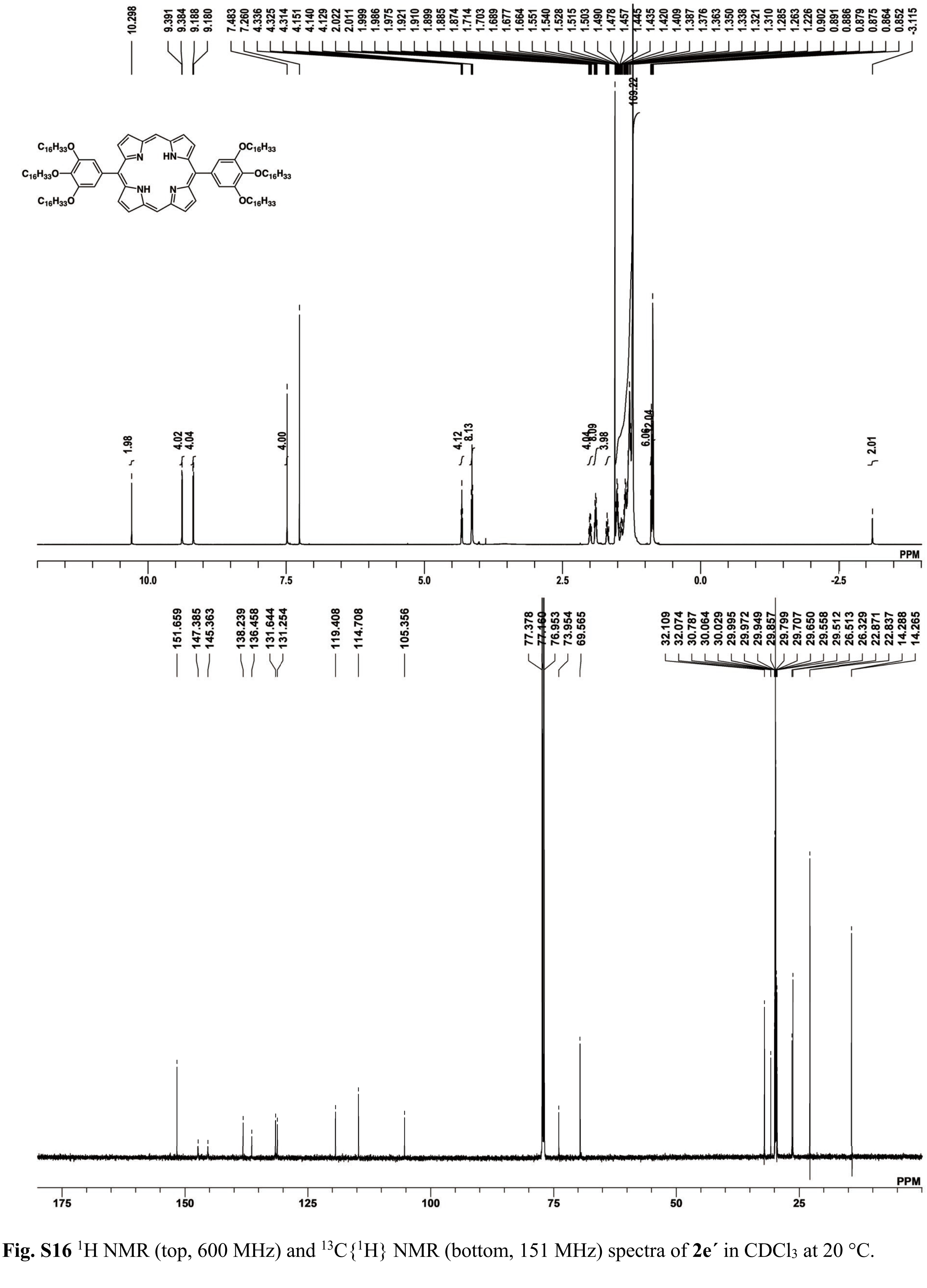

Fig. S16 ^{1}H NMR (top, 600 MHz) and $^{13}C\{^{1}H\}$ NMR (bottom, 151 MHz) spectra of **2e´** in $CDCl_3$ at 20 °C.

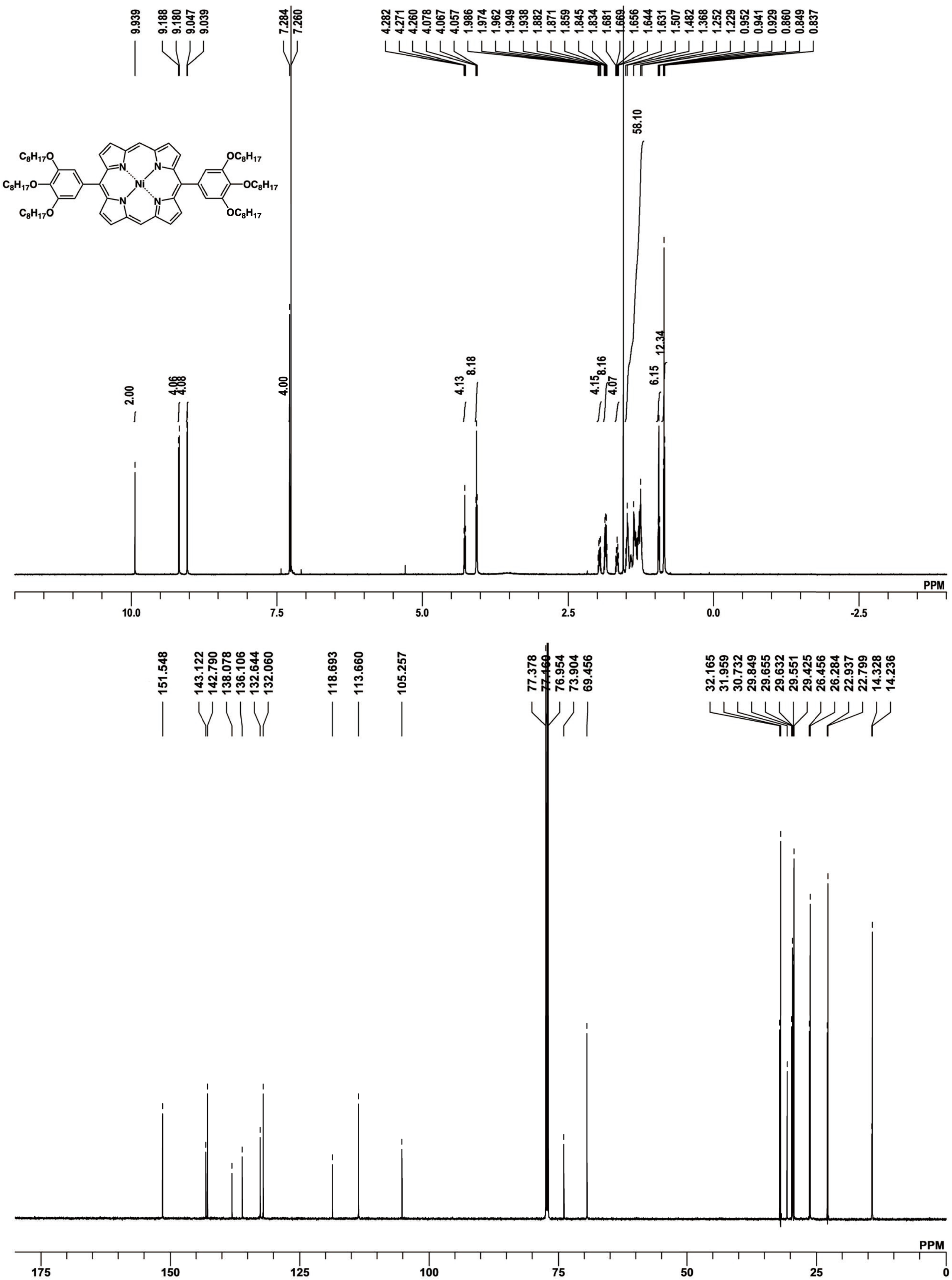

Fig. S17 ^{1}H NMR (top, 600 MHz) and ^{13}C{^{1}H} NMR (bottom, 151 MHz) spectra of **2c** in $CDCl_3$ at 20 °C.

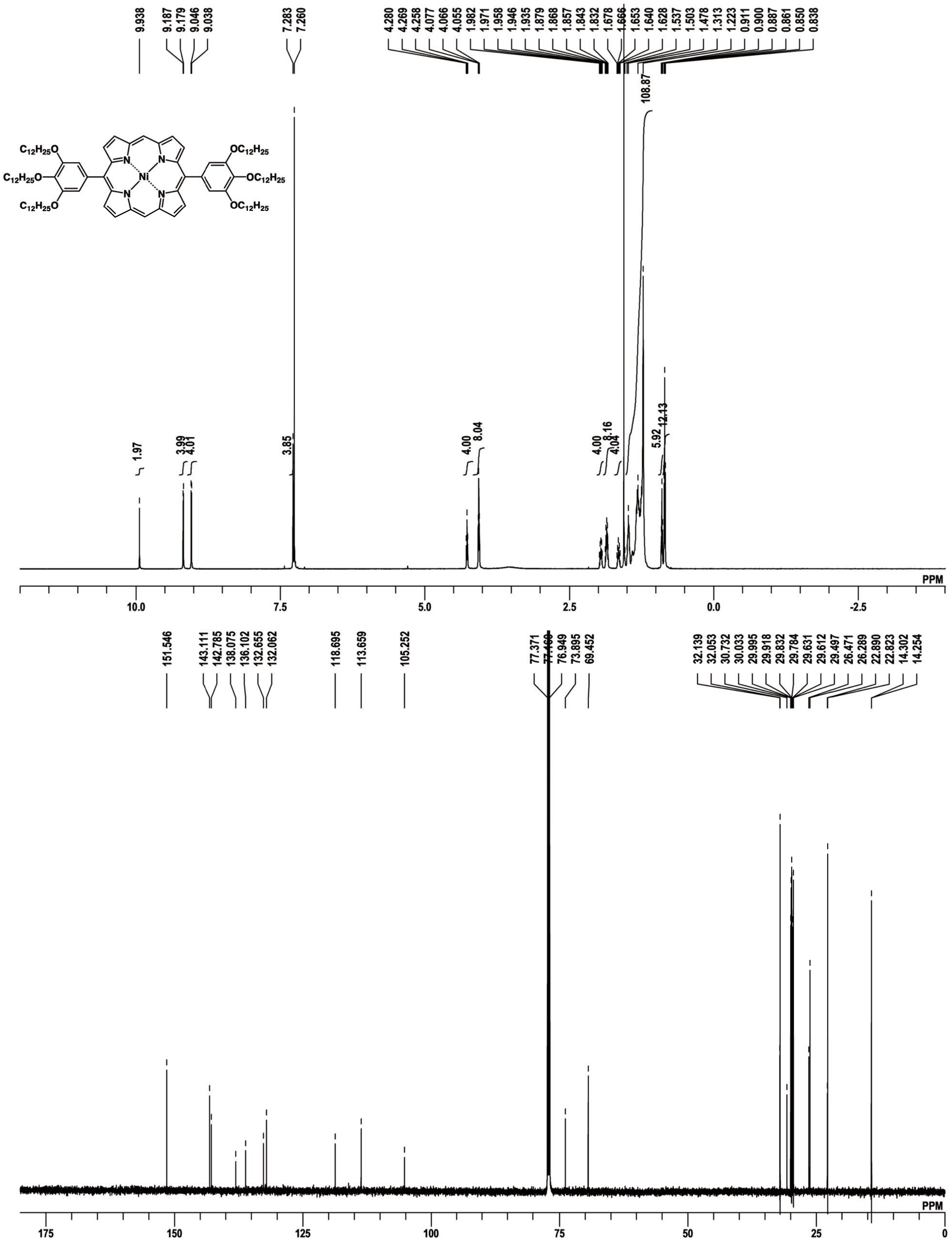

Fig. S18 ^{1}H NMR (top, 600 MHz) and $^{13}C\{^{1}H\}$ NMR (bottom, 151 MHz) spectra of **2d** in $CDCl_3$ at 20 °C.

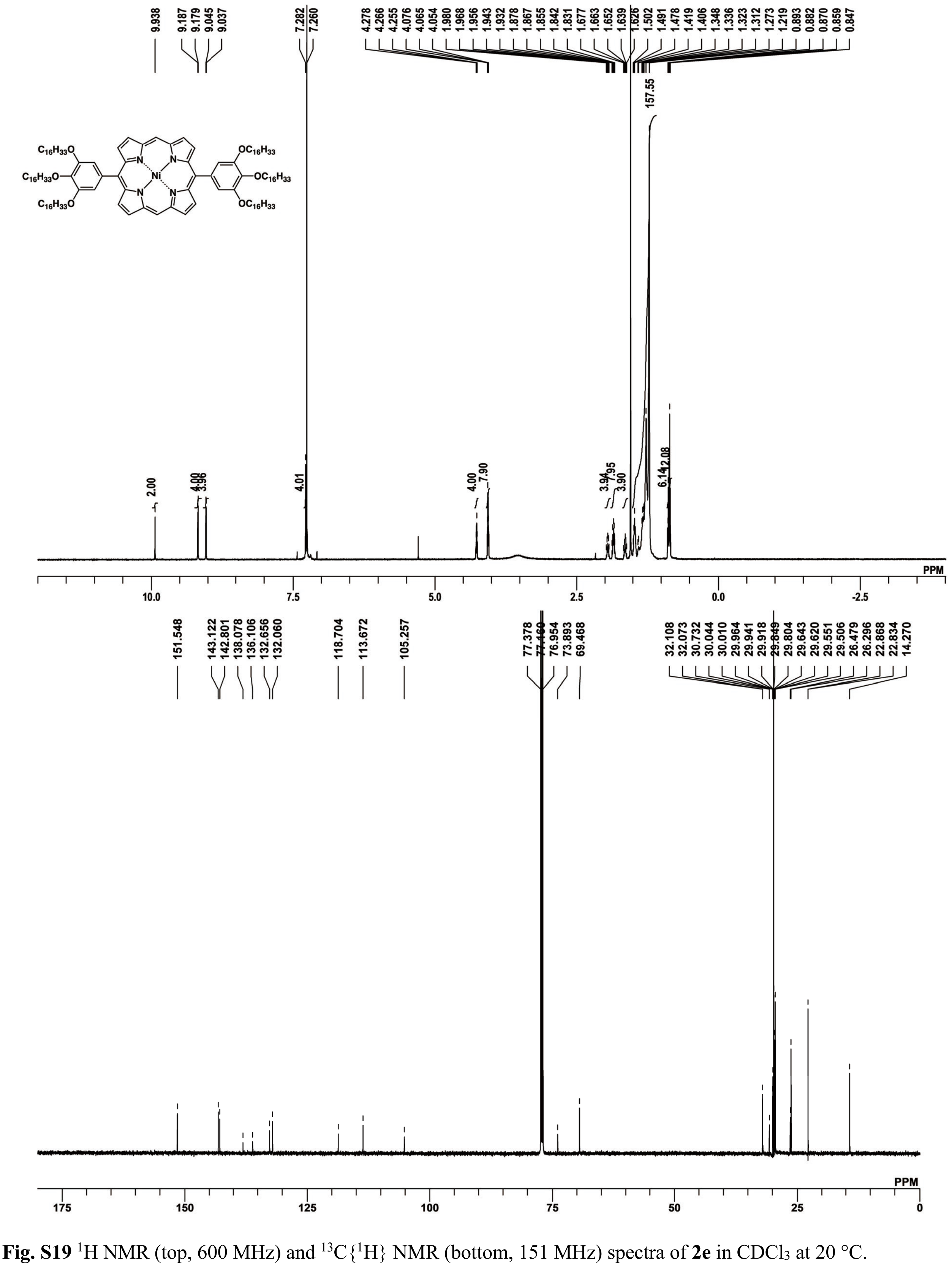

Fig. S19 ^{1}H NMR (top, 600 MHz) and ^{13}C{^{1}H} NMR (bottom, 151 MHz) spectra of **2e** in $CDCl_3$ at 20 °C.

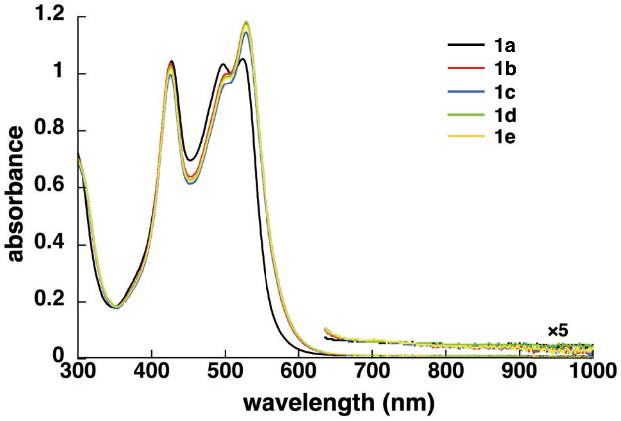

Fig. S20 UV/vis absorption spectra of **1a** (black), **1b** (red), **1c** (blue), **1d** (green), and **1e** (yellow) in CH_2Cl_2 (2.5×10^{-5} M).

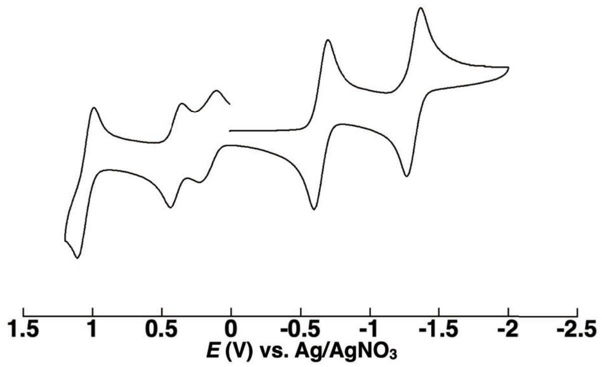

Fig. S21 Cyclic voltammogram (CV) of **1b** in CH_2Cl_2 (1.0×10^{-3} M) containing $TBAPF_6$ (0.1 M) as a supporting electrolyte at a scan rate of 100 mV/s. CV was measured under Ar atmosphere using an ALS/CH Instruments 720 electrochemical analyzer with a glassy-carbon disk working electrode (3-mm diameter), an $Ag/AgNO_3$ (0.010 M) reference electrode, and a Pt counter electrode. The reversible waves in the anodic and cathodic scans confirmed that the reduction at –0.64 and –1.32 V and the oxidation at 0.16, 0.40, and 1.07 V were reversible processes. Especially, intensities of first and second oxidation waves were almost half of the other three redox waves, indicating formation of a mixed-valence dimer as discussed in the previous report.[S10]

[S10] H. Kawashima, S. Ukai, R. Nozawa, N. Fukui, G. Fitzsimmons, T. Kowalczyk, H. Fliegl and H. Shinokubo, *J. Am. Chem. Soc.*, 2021, **143**, 10676–10685.

2. X-ray crystallographic data

Method for single-crystal X-ray analysis. Crystallographic data are summarized in Table S1. A single crystal of **s2a** was obtained by vapor diffusion of MeOH into a $CHCl_3$ solution. The data crystal was an orange prism of approximate dimensions 0.80 mm × 0.30 mm × 0.30 mm. A single crystal of **s3a** was obtained by vapor diffusion of *n*-hexane into a $CHCl_3$ solution. The data crystal was a green prism of approximate dimensions 0.45 mm × 0.18 mm × 0.01 mm. A single crystal of **1a** was obtained by vapor diffusion of MeOH into a chlorobenzene solution. The data crystal was a black prism of approximate dimensions 0.08 mm × 0.05 mm × 0.02 mm. A single crystal of **1b** was obtained by vapor diffusion of MeOH into a chlorobenzene solution. The data crystal was a black prism of approximate dimensions 0.12 mm × 0.04 mm × 0.01 mm. The data of **s2a** and **1b** were collected at 90 K on a DECTRIS EIGER X 1M diffractometer with Si (111) monochromated synchrotron radiation (λ = 0.81032 and 0.81082 Å, respectively) at BL40XU (SPring-8),[S11] whereas that of **s3a** was collected at 93 K on a Rigaku XtaLAB P200 diffractometer with graphite monochromated CuKα radiation (λ = 1.54184 Å). The data of **1a** was collected at 90 K on a Bruker D8 Venture diffractometer with MoKα radiation (λ = 0.71073 Å) focused by multilayer confocal mirror. All the structures were solved by dual-space method. The structures were refined by a full-matrix least-squares method by using a SHELXL 2014[S12] (Yadokari-XG).[S13] In each structure, the non-hydrogen atoms were refined anisotropically. CIF files (CCDC-2288764–2288767) can be obtained free of charge from the Cambridge Crystallographic Data Centre via www.ccdc.cam.ac.uk/data_request/cif.

Table S1 Crystallographic details.

	**s2a**	**s3a**	**1a**	**1b**
formula	$C_{18}H_{16}Br_2N_2O_3$	$C_{36}H_{30}Br_4N_4NiO_6$	$C_{36}H_{30}N_4NiO_6$	$C_{54}H_{66}N_4NiO_6·0.67CH_4O$
fw	468.15	992.93	673.35	947.18
crystal size, mm	0.80 × 0.30 × 0.30	0.45 × 0.18 × 0.01	0.08 × 0.05 × 0.02	0.12 × 0.04 × 0.01
crystal system	orthorhombic	monoclinic	monoclinic	triclinic
space group	*Pbca* (no. 61)	$P2_1/n$ (no. 14)	$P2_1/c$ (no. 14)	*P*-1 (no. 2)
a, Å	8.3326(2)	8.98210(10)	19.513(8)	13.0251(4)
b, Å	12.8054(3)	31.6135(3)	3.9317(17)	13.0786(4)
c, Å	32.7329(8)	12.7862(2)	18.474(7)	22.9482(9)
α, °	90	90	90	95.536(2)
β, °	90	90.9760(10)	96.947(15)	105.627(2)
γ, °	90	90	90	95.810(3)
V, Å^3	3492.67(14)	3630.19(8)	1406.9(10)	3714.0(2)
ρ_{calcd}, g cm^{-3}	1.781	1.817	1.589	1.270
Z	8	4	2	3
T, K	90(2)	93(2)	90(2)	90(2)
μ, mm^{-1}	6.459 ^{a}	6.361 ^{b}	0.750 ^{c}	0.632 ^{a}
no. of reflns	32477	22881	14567	39849
no. of unique reflns	3101	7103	5039	13559
variables	229	486	217	949
λ, Å	0.81032 ^{a}	1.54184 ^{b}	0.71073 ^{c}	0.81082 ^{a}
R_1 ($I > 2\sigma(I)$)	0.0500	0.0362	0.0834	0.0875
wR_2 ($I > 2\sigma(I)$)	0.1231	0.0941	0.1605	0.2344
GOF	1.121	1.075	1.150	1.034

^{a} Synchrotron radiation. ^{b} CuKα radiation. ^{c} MoKα radiation.

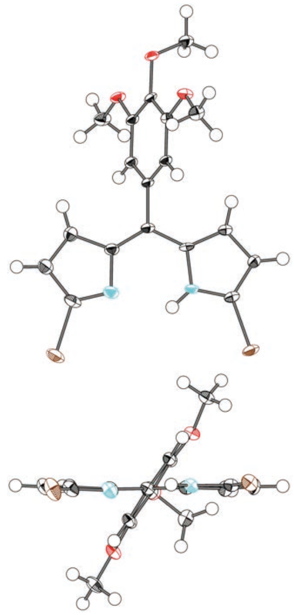

Fig. S22 Ortep drawing of single-crystal X-ray structure (top and side views) of **s2a**. Thermal ellipsoids are scaled to the 50% probability level. Atom color code: black, white (sphere), blue, red, and brown refer to carbon, hydrogen, nitrogen, oxygen, and bromine, respectively.

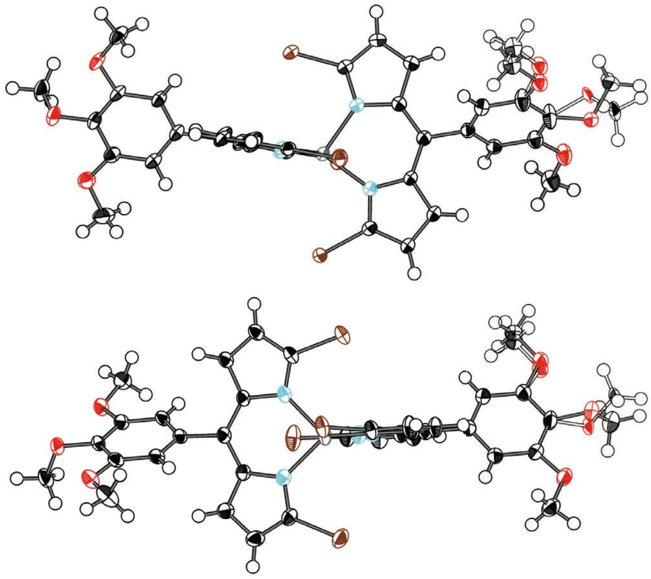

Fig. S23 Ortep drawing of single-crystal X-ray structure (top and side views) of **s3a**. Thermal ellipsoids are scaled to the 50% probability level. Alkoxy groups have disordered structures in the ratio of 69 (black bond) : 31 (white bond) and 65 (black bond) : 35 (white bond). Atom color code: black, white (sphere), blue, red, brown, and gray refer to carbon, hydrogen, nitrogen, oxygen, bromine, and nickel, respectively.

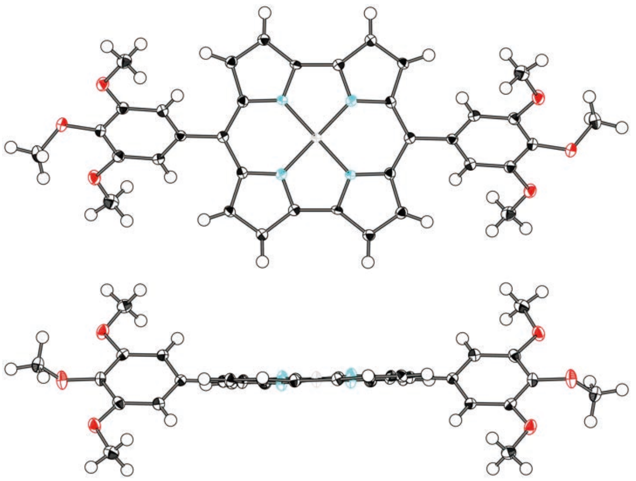

Fig. S24 Ortep drawing of single-crystal X-ray structure (top and side views) of **1a**. Thermal ellipsoids are scaled to the 50% probability level. Atom color code: black, white (sphere), blue, red, and gray refer to carbon, hydrogen, nitrogen, oxygen, and nickel, respectively.

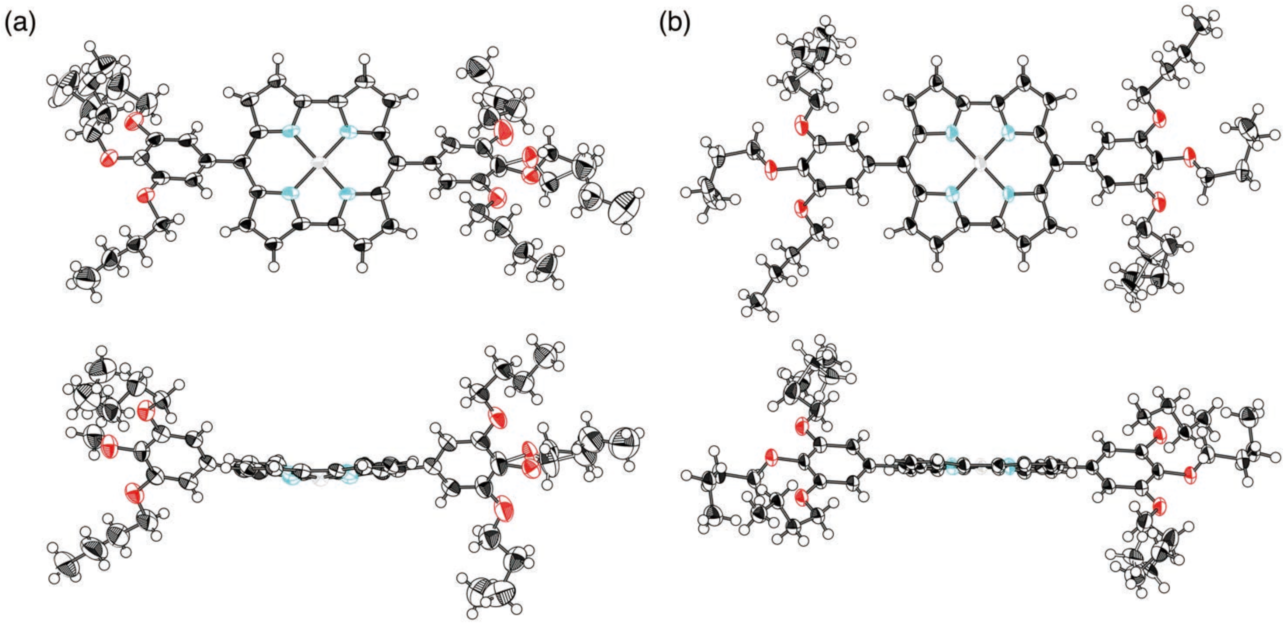

Fig. S25 Ortep drawing of single-crystal X-ray structure (top and side views) of **1b** as two independent structures (a,b). Thermal ellipsoids are scaled to the 50% probability level. An alkoxy group in (a) has disordered structures in the ratio of 62 (black bond) : 38 (white bond), whereas that in (b) has disordered structures in the ratio of 85 (black bond) : 15 (white bond). Solvent molecules are omitted for clarity. Atom color code: black, white (sphere), blue, red, and gray refer to carbon, hydrogen, nitrogen, oxygen, and nickel, respectively.

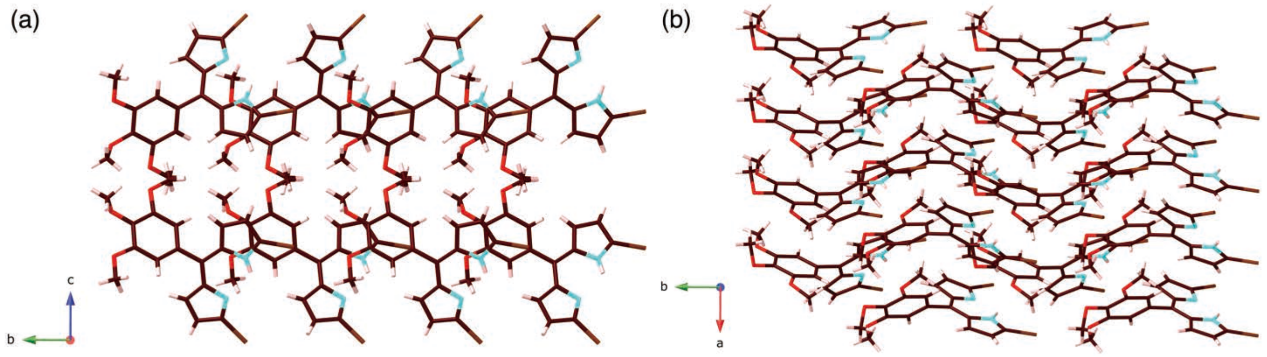

Fig. S26 Packing diagrams of **s2a** through (a) *a* and (b) *c* axes. Atom color code: brown, pink, cyan, red, and light brown refer to carbon, hydrogen, nitrogen, oxygen, and bromine, respectively.

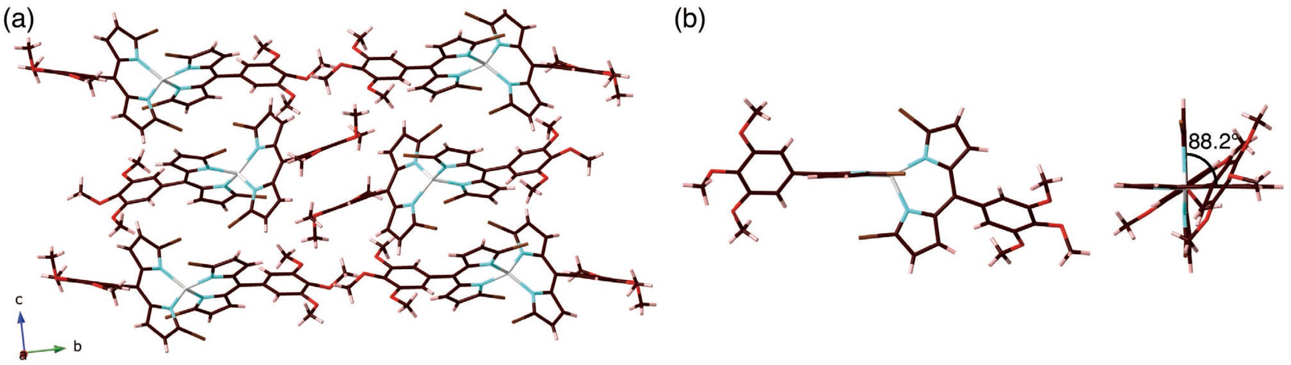

Fig. S27 (a) Packing diagram of **s3a** through *a* axis and (b) constituent monomer. Dihedral angle between two dipyrrin units is 88.2°. Atom color code: brown, pink, cyan, red, light brown, and gray refer to carbon, hydrogen, nitrogen, oxygen, bromine, and nickel, respectively.

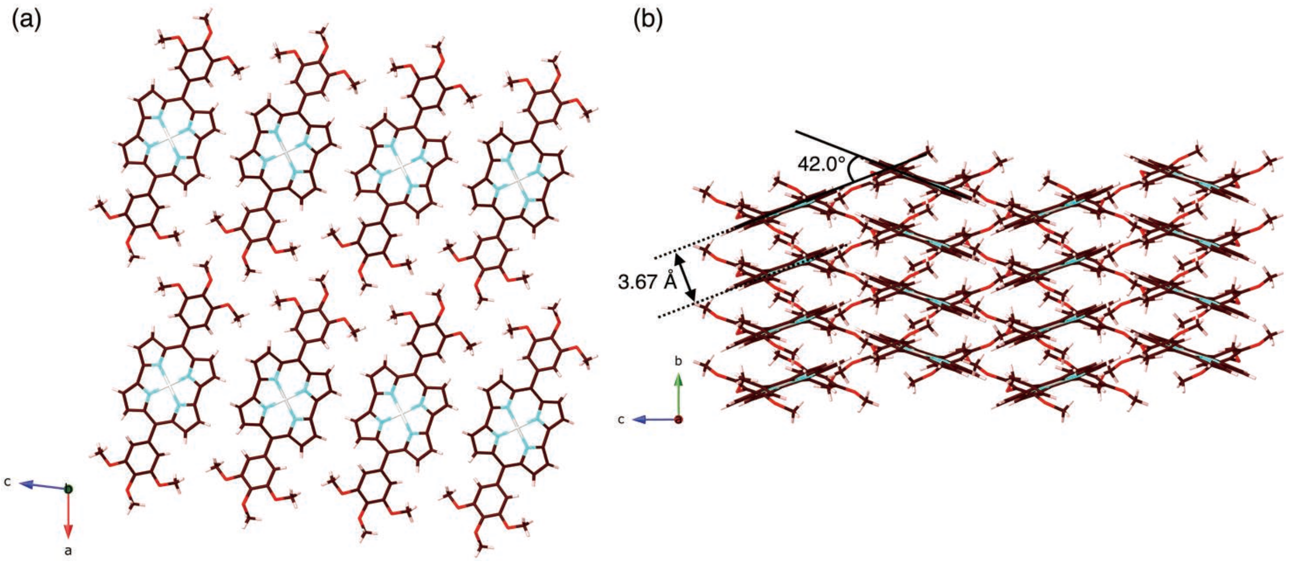

Fig. S28 Packing diagrams of **1a** through (a) *b* and (b) *a* axes. The distance between the two nickel centers is 3.93 Å and the distance between two mean planes of **1a** (core 23 atoms including Ni) is 3.67 Å. Dihedral angles between two mean planes of **1a** located in adjacent column is 42.0°. Atom color code: brown, pink, cyan, red, and gray refer to carbon, hydrogen, nitrogen, oxygen, and nickel, respectively.

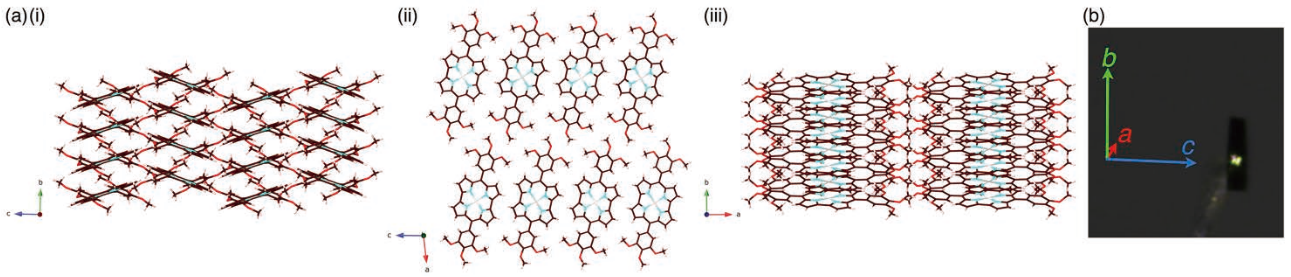

Fig. S29 (a) Packing diagrams of **1a** through (i) *a*, (ii) *b*, and (iii) *c* axes and (b) photograph of the single crystal. The unit cell axis corresponding to the long axis of the crystal is the *b* axis, as determined by the indexing crystal diffraction pattern.

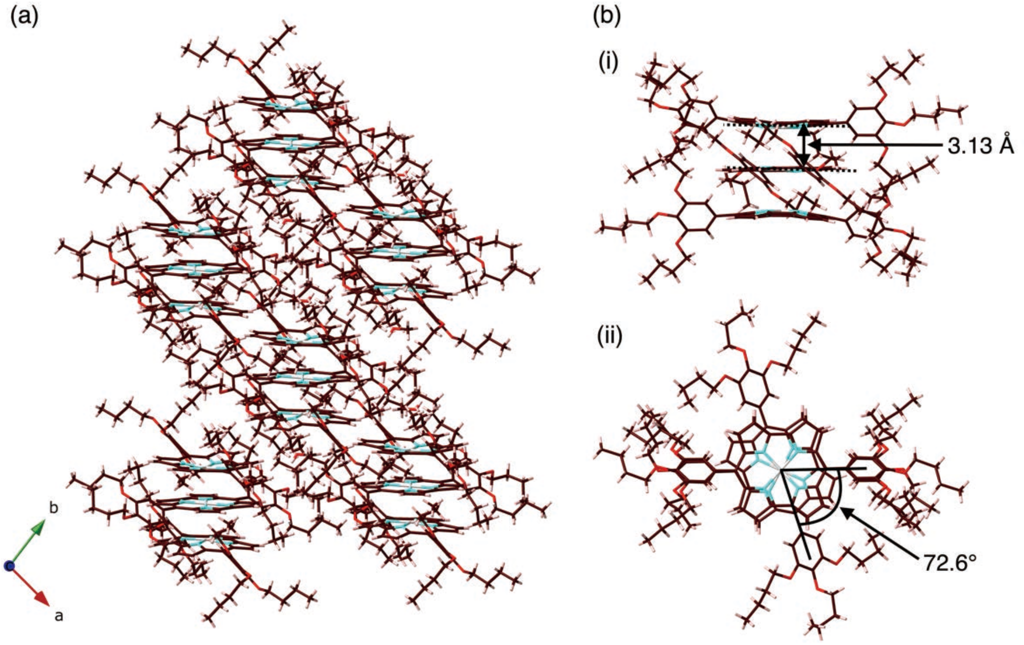

Fig. S30 (a) Packing diagrams of **1b** through *c* axis and (b) constituent trimers as (i) side and (ii) top views. The distance between the two nickel centers is 3.00 Å, and the distance between two **1b** (four N atom plane) is 3.13 Å. The center molecule is rotated at 72.6° relative to the outer two molecules. Solvent molecules are omitted for clarity. Atom color code: brown, pink, cyan, red, and gray refer to carbon, hydrogen, nitrogen, oxygen, and nickel, respectively.

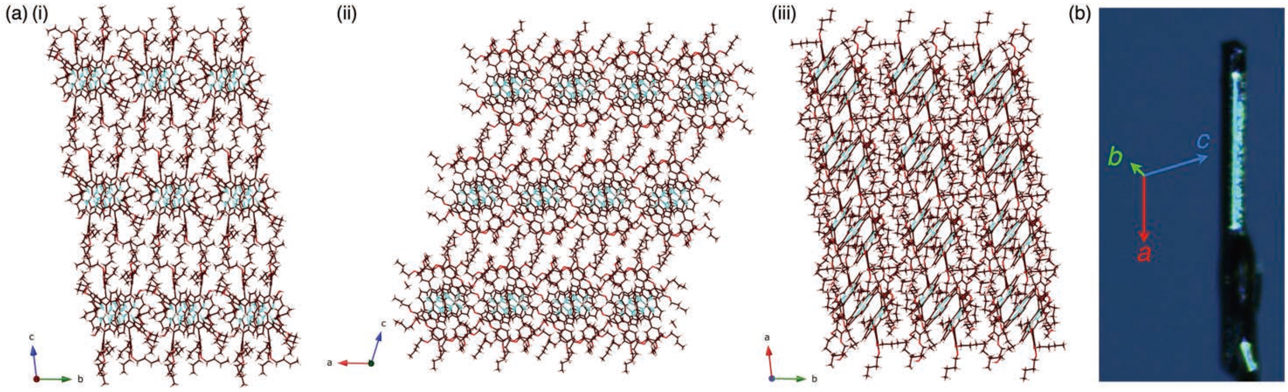

Fig. S31 (a) Packing diagrams of **1b** through (i) *a*, (ii) *b*, and (iii) *c* axes and (b) photograph of the single crystal. The unit cell axis corresponding to the long axis of the crystal is the *a* axis, as determined by the indexing crystal diffraction pattern.

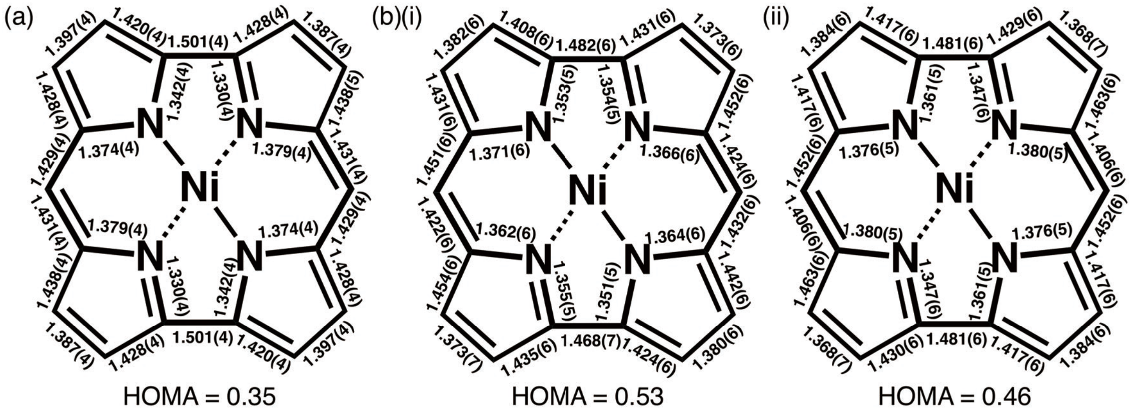

Fig. S32 Bond lengths and harmonic oscillator model of aromaticity (HOMA) values[S14] of (a) **1a** and (b) **1b** located at (i) top and (ii) center units of the triple decker in their X-ray crystal structures. The HOMA values were calculated based on 14 bonds indicated with bold lines following a related report.[S15] The HOMA value of **1a** was comparable to that of optimized structure of antiaromatic **1a** monomer (0.36, Fig. S38), whereas those of **1b** were larger due to the contribution of stacked-ring aromaticity of **1b**. These results were consistent with (anti)aromaticity supported by 2D NICS plots (Fig. S46,47).

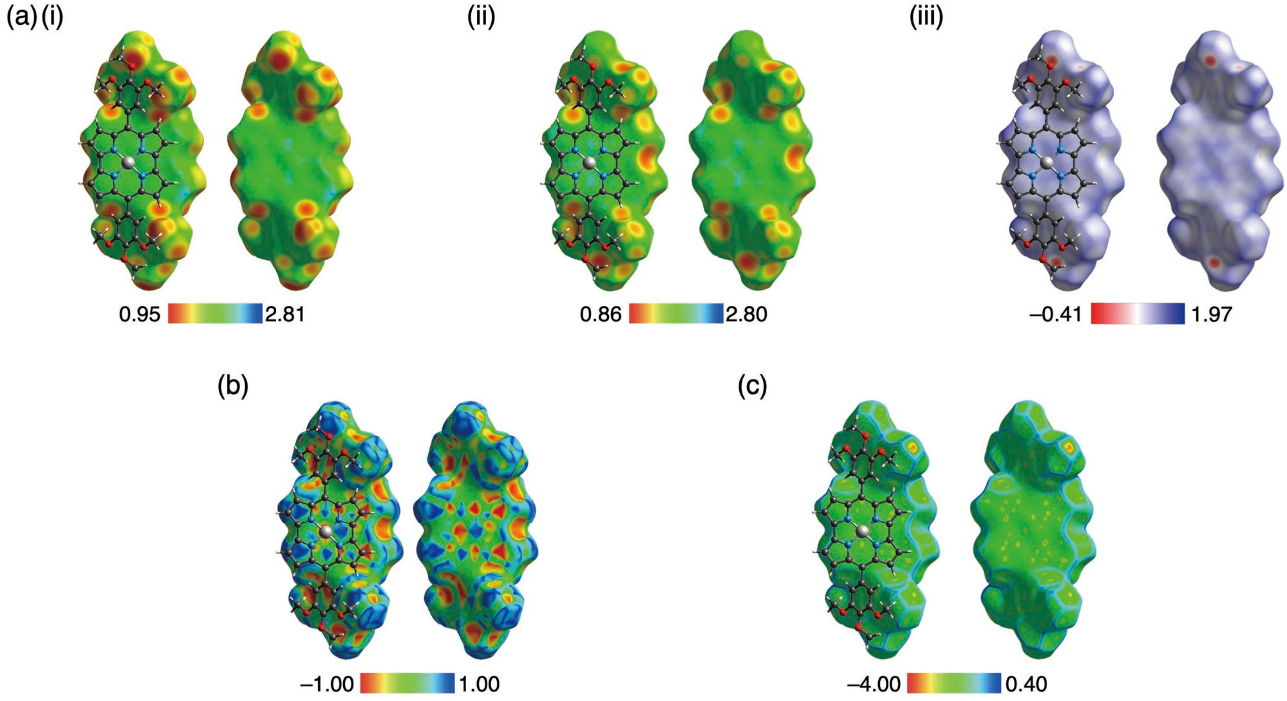

Fig. S33 Hirshfeld surface[S16] of **1a** from the crystal structure (Fig. S28): (a) mapped with (i) d_{i}, (ii) d_{e}, and (iii) d_{norm}, (b) mapped with shape index, and (c) mapped with curvedness. Atom color code: black, white, blue, red, and gray refer to carbon, hydrogen, nitrogen, oxygen, and nickel, respectively. Hirshfeld surface mapped with shape index exhibits red and blue triangles arranged in bow-tie shape, indicating the π–π stackings of norcorroles.

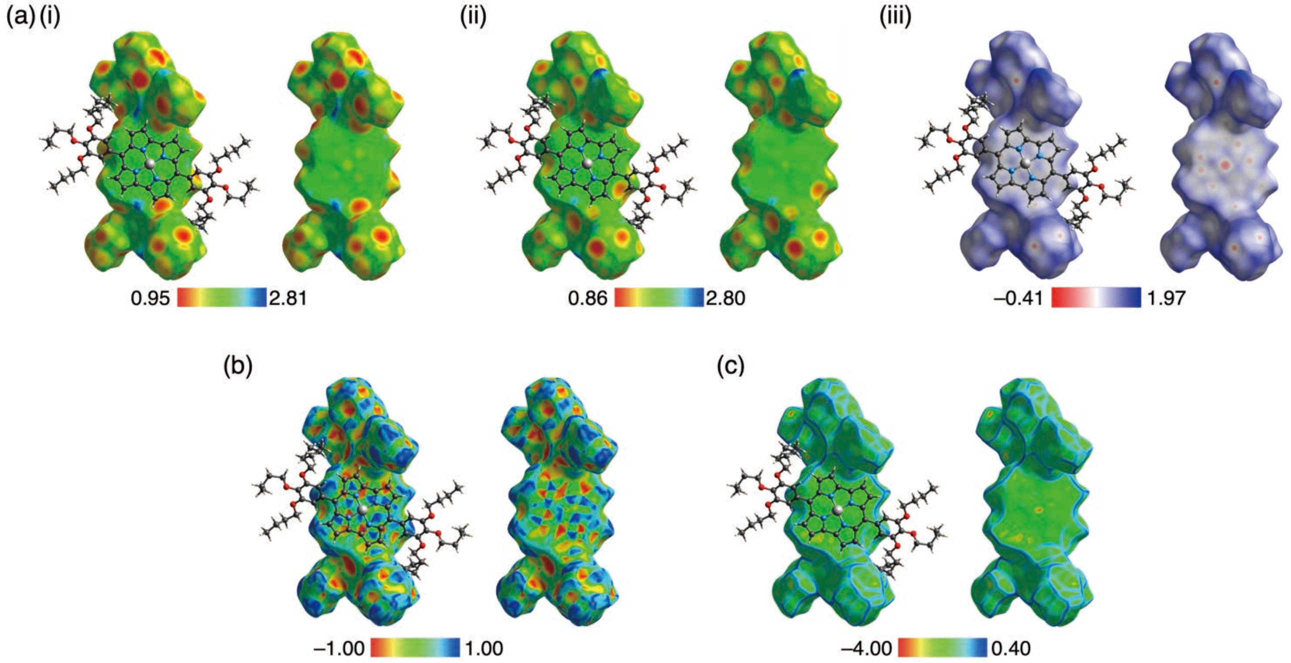

Fig. S34 Hirshfeld surface[S16] of **1b** (the outer **1b** in the triple decker) from the crystal structure (Fig. S30): (a) mapped with (i) d_i, (ii) d_e, and (iii) d_norm, (b) mapped with shape index, and (c) mapped with curvedness. Solvent molecules are omitted for clarity. Atom color code: black, white, blue, red, and gray refer to carbon, hydrogen, nitrogen, oxygen, and nickel, respectively. Hirshfeld surface mapped with shape index exhibits red and blue triangles arranged in bow-tie shape, indicating the π–π stackings of norcorroles.

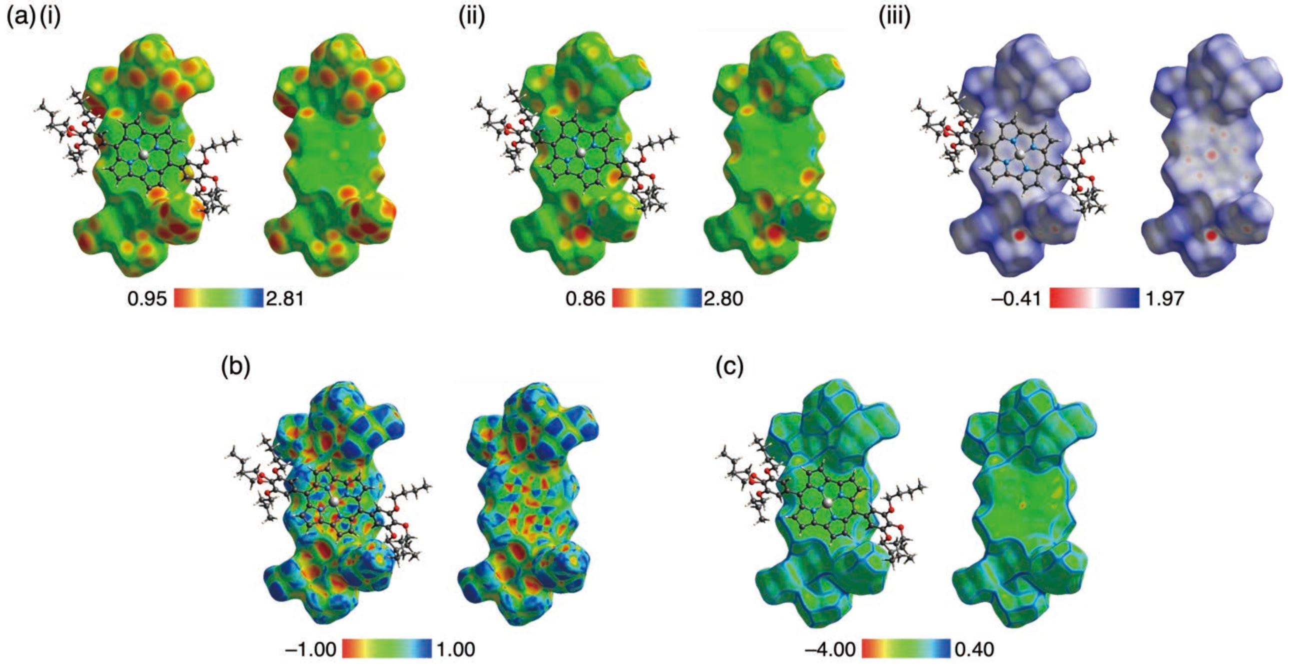

Fig. S35 Hirshfeld surface[S16] of **1b** (the center **1b** in the triple decker) from the crystal structure (Fig. S30): (a) mapped with (i) d_i, (ii) d_e, and (iii) d_norm, (b) mapped with shape index, and (c) mapped with curvedness. Solvent molecules are omitted for clarity. Atom color code: black, white, blue, red, and gray refer to carbon, hydrogen, nitrogen, oxygen, and nickel, respectively. Hirshfeld surface mapped with shape index exhibits red and blue triangles arranged in bow-tie shape, indicating the π–π stackings of norcorroles.

[S11] (a) N. Yasuda, H. Murayama, Y. Fukuyama, J. E. Kim, S. Kimura, K. Toriumi, Y. Tanaka, Y. Moritomo, Y. Kuroiwa, K. Kato, H. Tanaka and M. Takata, *J. Synchrotron Rad.*, 2009, **16**, 352–357; (b) N. Yasuda, Y. Fukuyama, K. Toriumi, S. Kimura and M. Takata, *AIP Conf. Proc.*, 2010, **1234**, 147–150.
[S12] G. M. Sheldrick, *Acta Crystallogr. Sect. A*, 2008, **64**, 112–122.

[S13] (a) K. Wakita, *Yadokari-XG*, *Software for Crystal Structure Analyses*, 2001; (b) C. Kabuto, S. Akine, T. Nemoto and E. Kwon, *J. Cryst. Soc. Jpn.*, 2009, **51**, 218–224.
[S14] T. M. Krygowski and M. K. Cyrański, *Chem. Rev.*, 2001, **101**, 1385–1420.
[S15] R. Nozawa, J. Kim, J. Oh, A. Lamping, Y. Wang, S. Shimizu, I. Hisaki, T. Kowalczyk, H. Fliegl, D. Kim and H. Shinokubo, *Nat. Commun.*, 2019, **10**, 3576.
[S16] P. R. Spackman, M. J. Turner, J. J. McKinnon, S. K. Wolff, D. J. Grimwood, D. Jayatilaka and M. A. Spackman, *J. Appl. Cryst.*, 2021, **54**, 1006–1011.

3. Theoretical studies

DFT calculations. DFT calculations were carried out by using the *Gaussian 16* program.[S17]

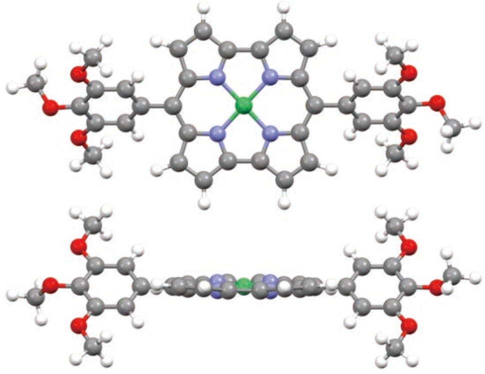

Fig. S36 Optimized structure (top and side views) of **1a** at B3LYP/6-31G(d,p) with B3LYP/SDD for Ni based on the crystal structure.

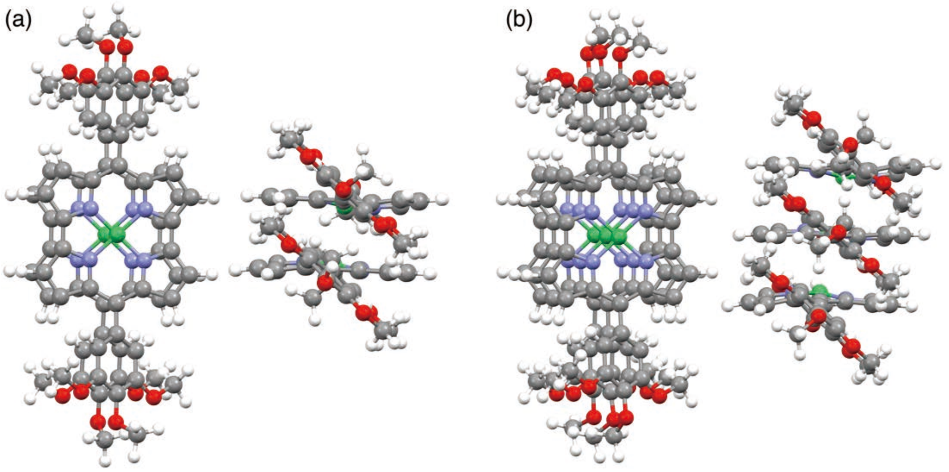

Fig. S37 Optimized structures (top and side views) of **1a** as (a) stacked dimer (double decker) and (b) stacked trimer (triple decker) at B97D3/6-31G(d,p) with B97D3/SDD for Ni based on the crystal structure. B97D3 was used for optimizing stacked structures as a functional to consider dispersion forces.

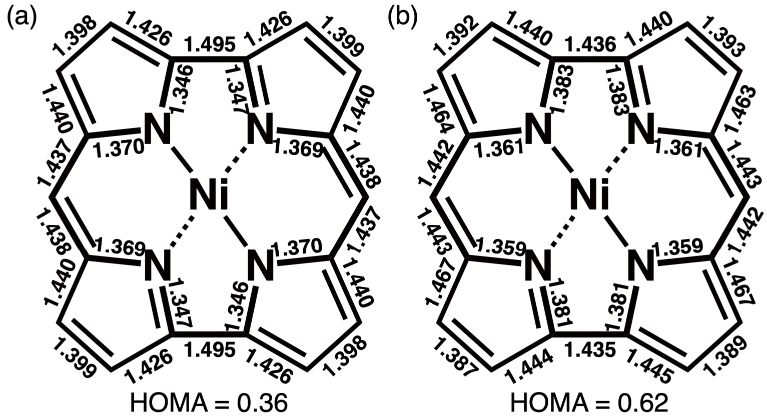

Fig. S38 Bond lengths (Å) and harmonic oscillator model of aromaticity (HOMA) values based on **1a** as (a) monomer and (b) stacked dimer (one of the norcorrole units was shown as both monomeric structures were almost identical) shown in Fig. S37.

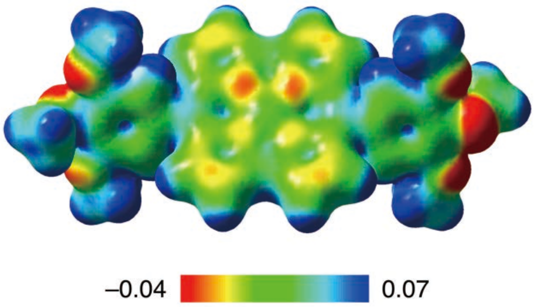

Fig. S39 Electrostatic potentials (ESP) of **1a** mapped onto the electron density isosurfaces (δ = 0.01) at B3LYP/6-31+G(d,p) with B3LYP/SDD for Ni.

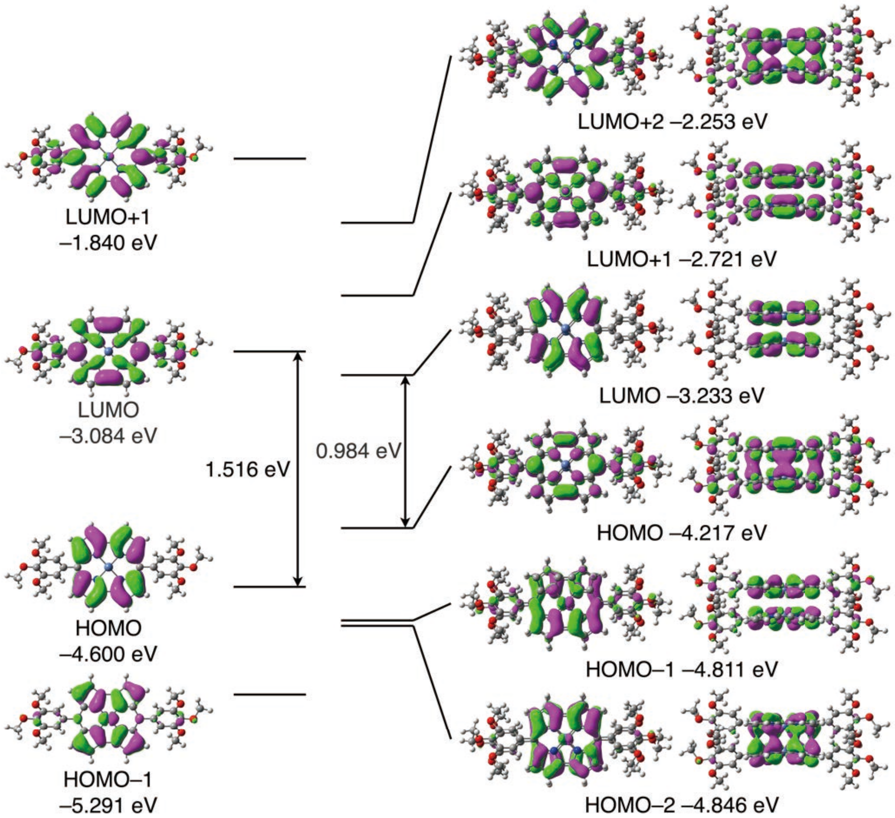

Fig. S40 Molecular orbitals (MOs) of optimized structures of monomeric **1a** at B3LYP/6-31G(d,p) with B3LYP/SDD for Ni and stacked dimer of **1a** at B3LYP/6-31G(d,p)//B97D3/6-31G(d,p) with B3LYP/SDD//B97D3/SDD for Ni. In-phase and out-of-phase combination of two LUMOs of monomeric **1a** afforded HOMO and LUMO+1 of stacked dimer of **1a**, respectively, although the orbitals derived from those of two HOMOs of monomeric **1a** were ambiguous (presumably HOMO–2 or HOMO–1 and LUMO of the stacked dimer). Significant destabilization of the orbital in out-of-phase combination of the two HOMOs and stabilization of the orbital in in-phase combination of the two LUMOs can be ascribed to strong orbital interactions, which cause the inversion of energy levels of these orbitals as discussed for the previously reported norcorrole dimer.[S10]

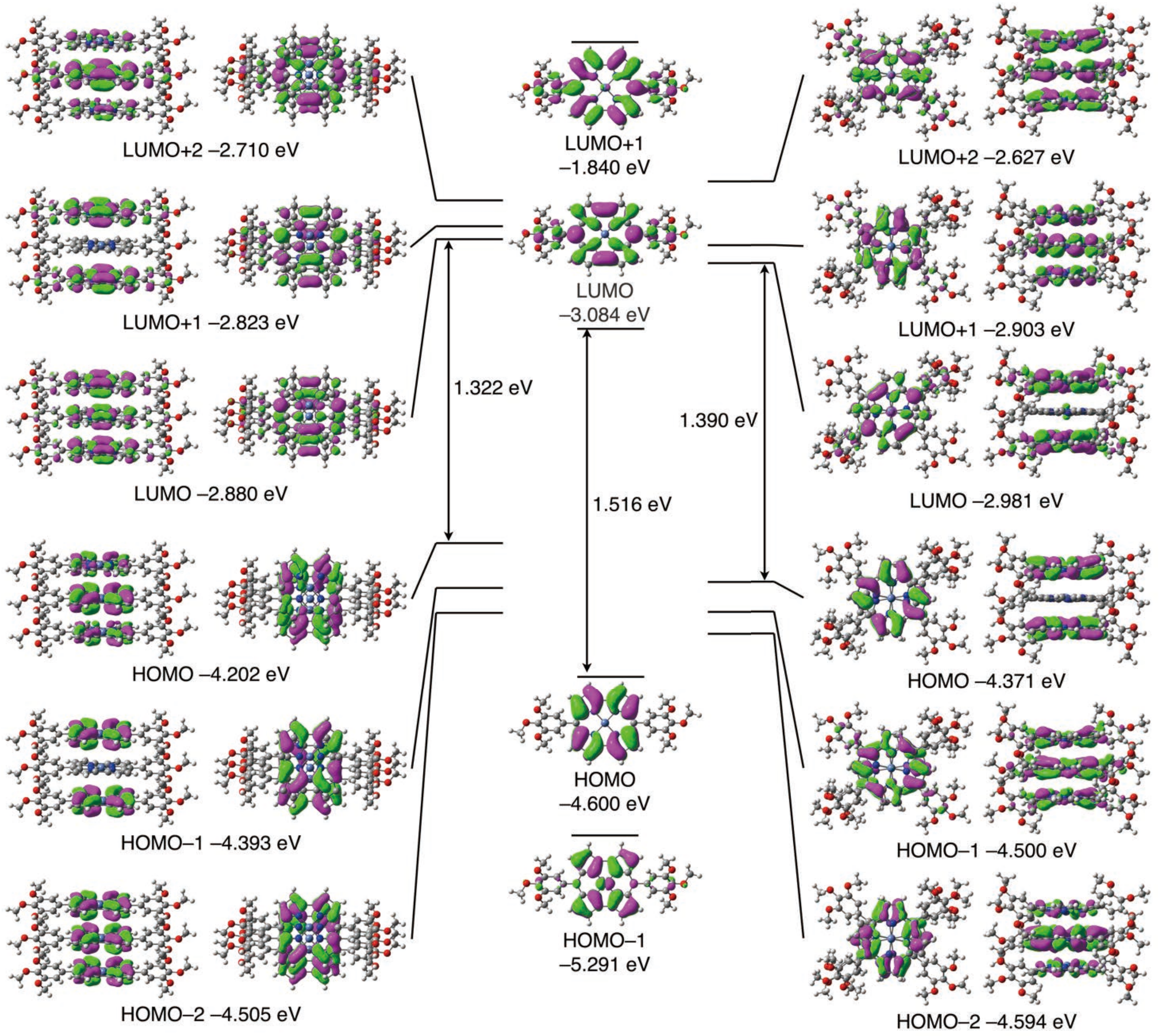

Fig. S41 MOs of monomeric **1a** (optimized structure, center), stacked trimer observed in the crystal structures of **1a** (left, slip-stacked trimer), and **1b**, in which butoxy groups are replaced with methoxy groups (right, face-to-face stacked trimer), at B3LYP/6-31G(d,p) with B3LYP/SDD for Ni. Similar to three-center bonds, orbital interactions among three MOs can provide bonding (comprising three MOs in in-phase combination), non-bonding (comprising two MOs of both terminal units), and antibonding orbitals (comprising three MOs in out-of-phase combination). In fact, the three occupied (HOMO–2, HOMO–1, and HOMO) and three unoccupied orbitals (LUMO, LUMO+1, and LUMO+2) of the slip-stacked trimer consist of three HOMOs and three LUMOs of monomeric **1a**, respectively, and can be ascribed to bonding, nonbonding, and antibonding orbitals, respectively. On the other hand, HOMO and LUMO of the face-to-face stacked trimer were ascribed to the nonbonding orbital consisting of the two HOMOs of both terminal units and that of the two LUMOs, respectively. The difference between the two stacking modes is presumably ascribed to the absence or presence of the significant orbital interactions caused by face-to-face stacking, which is crucial for stacked-ring aromaticity.[S10]

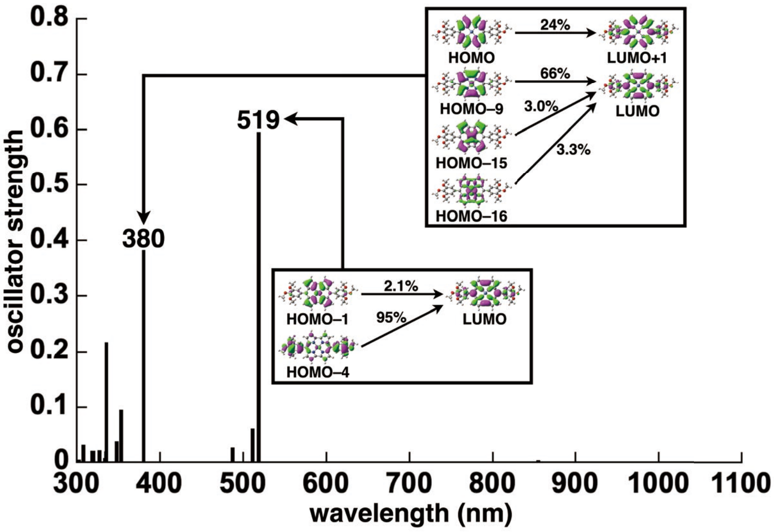

Fig. S42 TD-DFT-based UV/vis absorption stick spectra of **1a** with the transitions correlated with MOs estimated at B3LYP/6-31G(d,p) with B3LYP/SDD for Ni. UV/vis absorption of **1a** at 524 nm (Fig. S20) can be assigned to the CT transition band from the trialkoxyphenyl units to the norcorrole core.

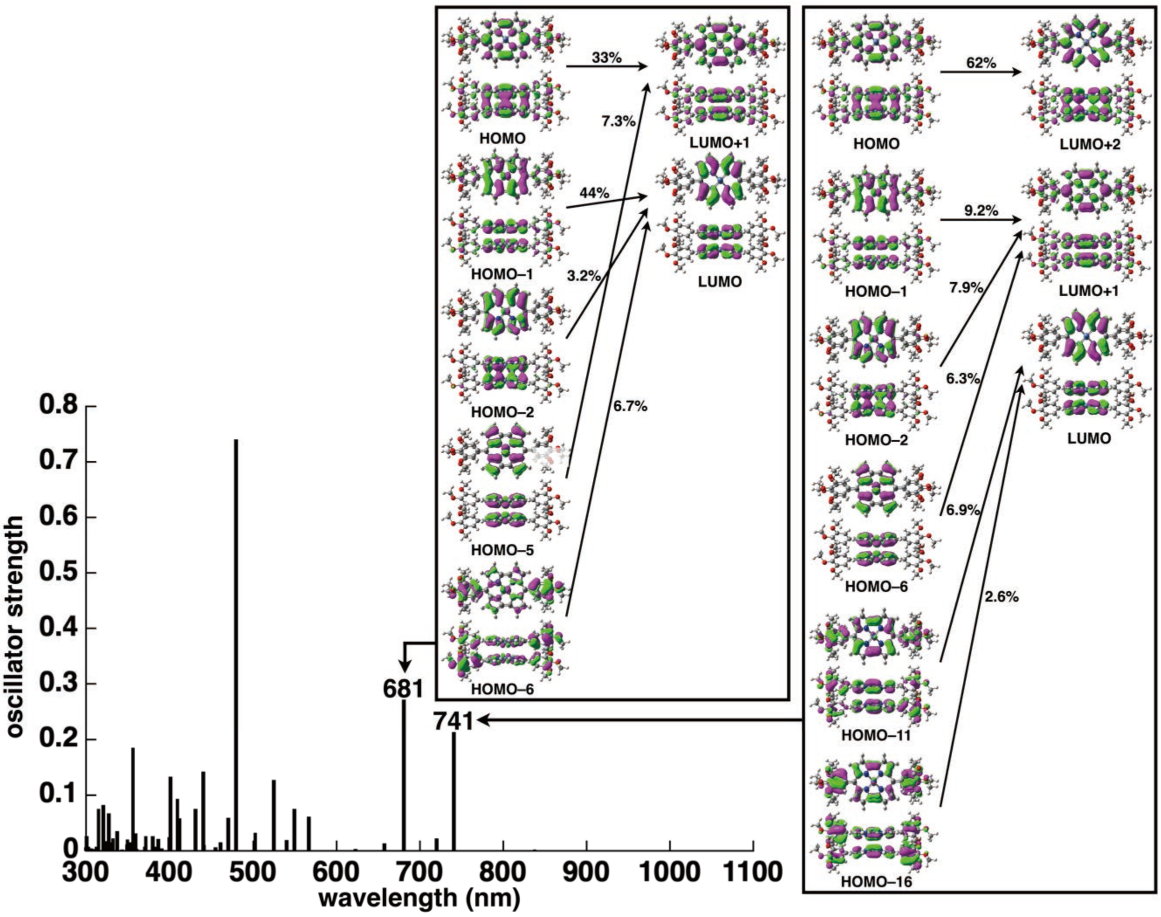

Fig. S43 TD-DFT-based UV/vis absorption stick spectra of **1a** as the optimized structure of the stacked dimer (Fig. S37), with the transitions correlated with MOs, estimated at B3LYP/6-31G(d,p)//B97D3/6-31G(d,p) with B3LYP/SDD//B97D3/SDD for Ni.

(a)

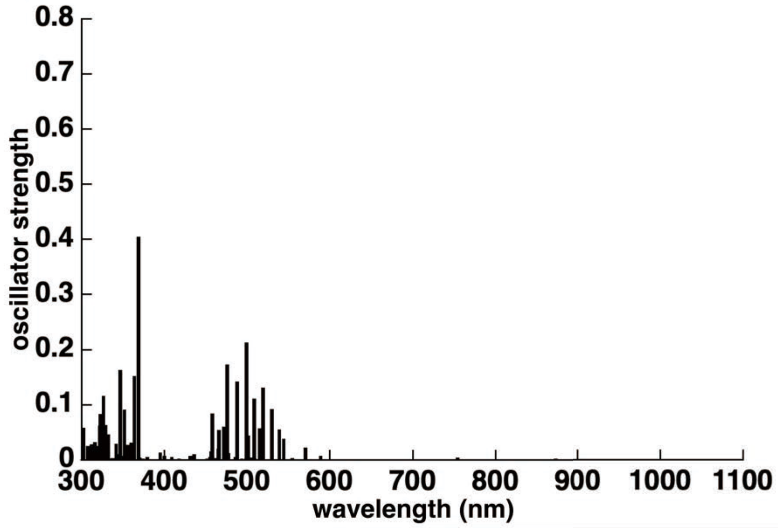

(b)

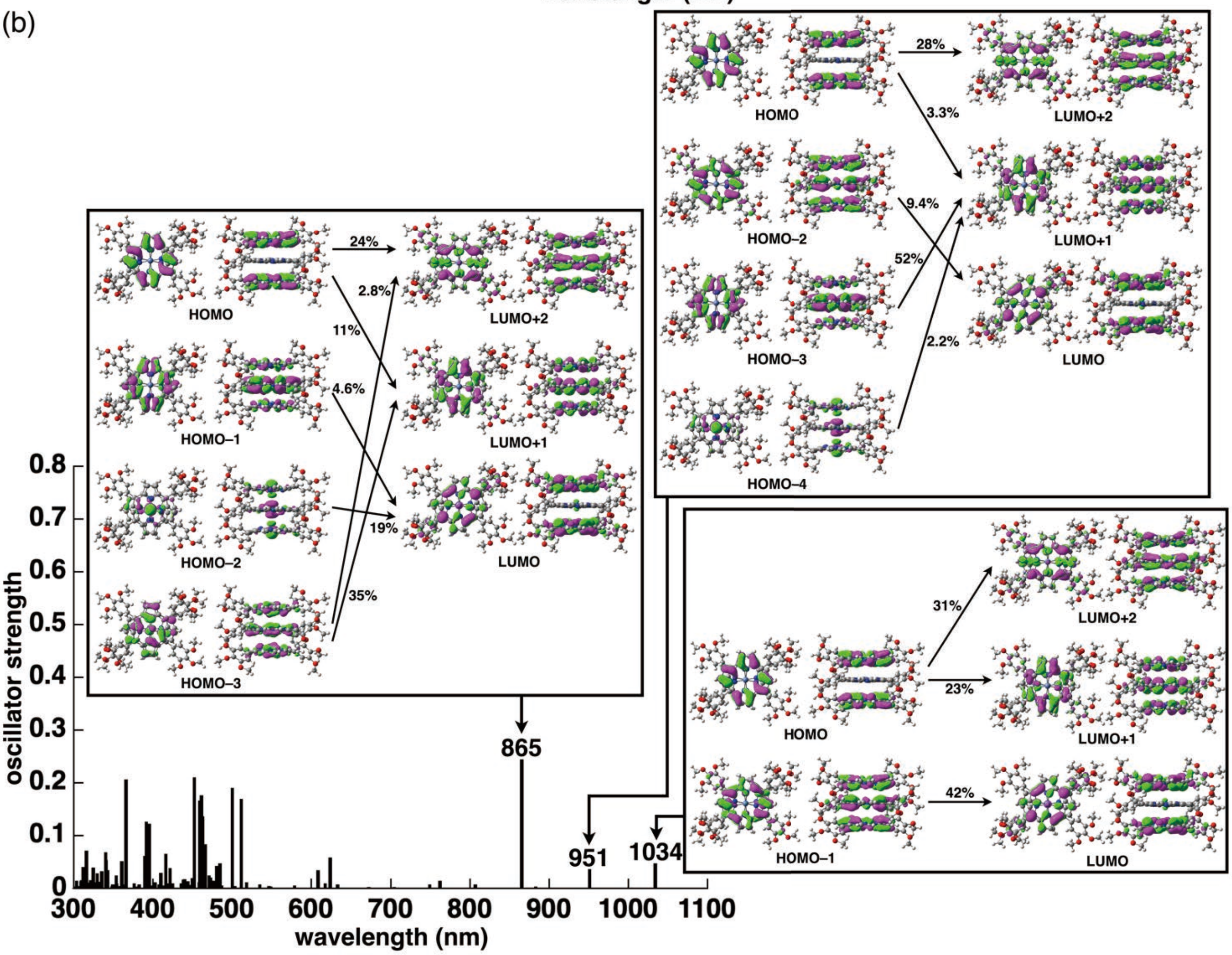

Fig. S44 TD-DFT-based UV/vis absorption stick spectra of (a) **1a** as the crystal-state stacked trimer (Fig. S28) and (b) **1b** as the crystal-state stacked trimer (Fig. S30), in which butoxy groups are replaced with methoxy groups, with the transitions correlated with MOs, estimated at B3LYP/6-31G(d,p) with B3LYP/SDD for Ni.

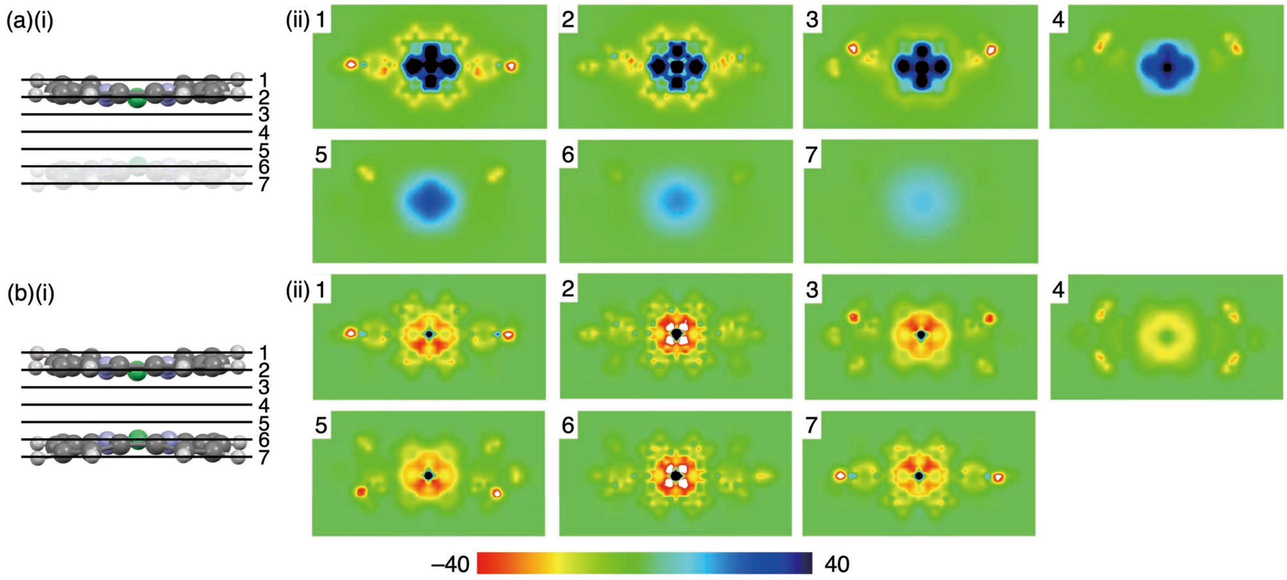

Fig. S45 (i) Arrangements of the optimized structure of stacked **1a** dimer and (ii) 2D NICS plots as (a) monomer and (b) stacked dimer at BHLYP/6-31G(d)//B97D3/6-31G(d,p) with BHLYP/SDD//B97D3/SDD for Ni.[S18] The plots were obtained at the plane indicated in the optimized structure. *Meso*-aryl groups in (i) were omitted for clarity. B3LYP functional tends to overestimate the paramagnetic character, which is relatively accurately evaluated by BHLYP functional.[S19] Negative NICS values in (b) indicated the stacked-ring aromaticity.

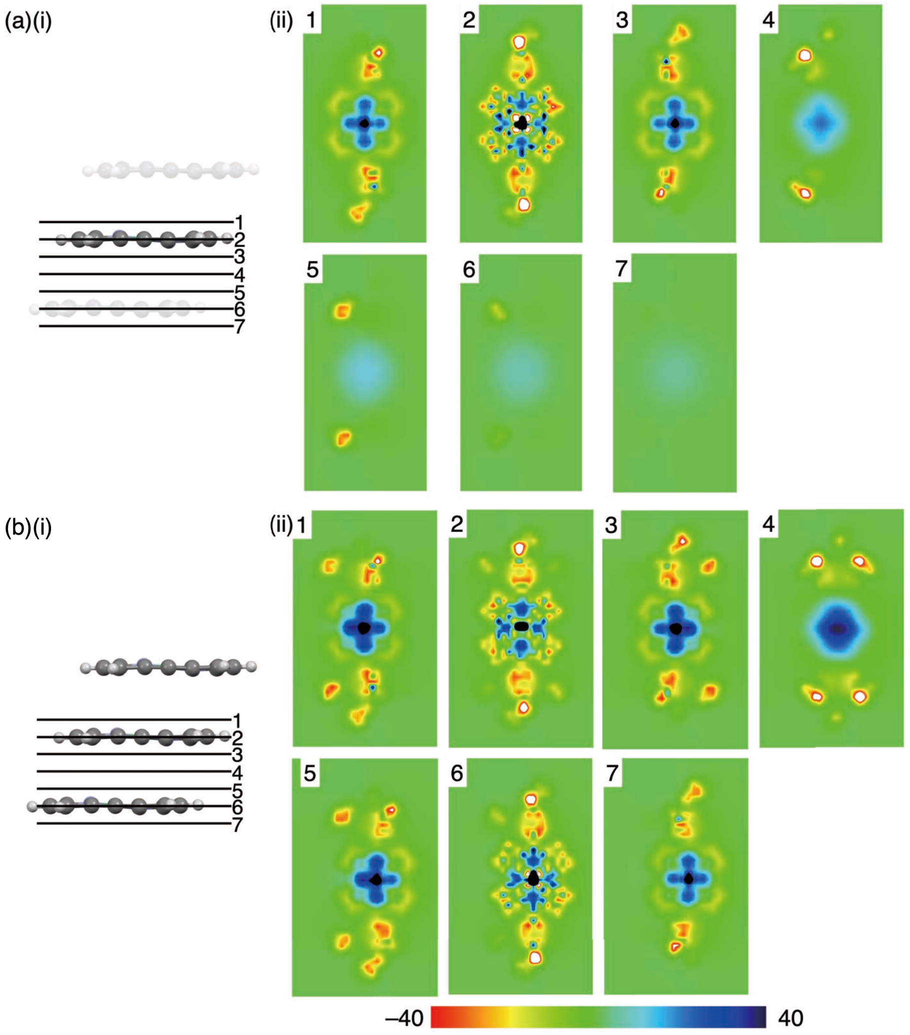

Fig. S46 (i) Arrangements in the crystal structure of **1a** and (ii) 2D NICS plots as (a) monomer and (b) stacked trimer at BHLYP/6-31G(d) with BHLYP/SDD for Ni.[S18] The plots were obtained at the plane indicated in the crystal structures. *Meso*-aryl groups in (i) were omitted for clarity. Larger positive NICS values around nickel center in (b) than those in (a) indicated that the antiaromaticity of each norcorrole unit was maintained.

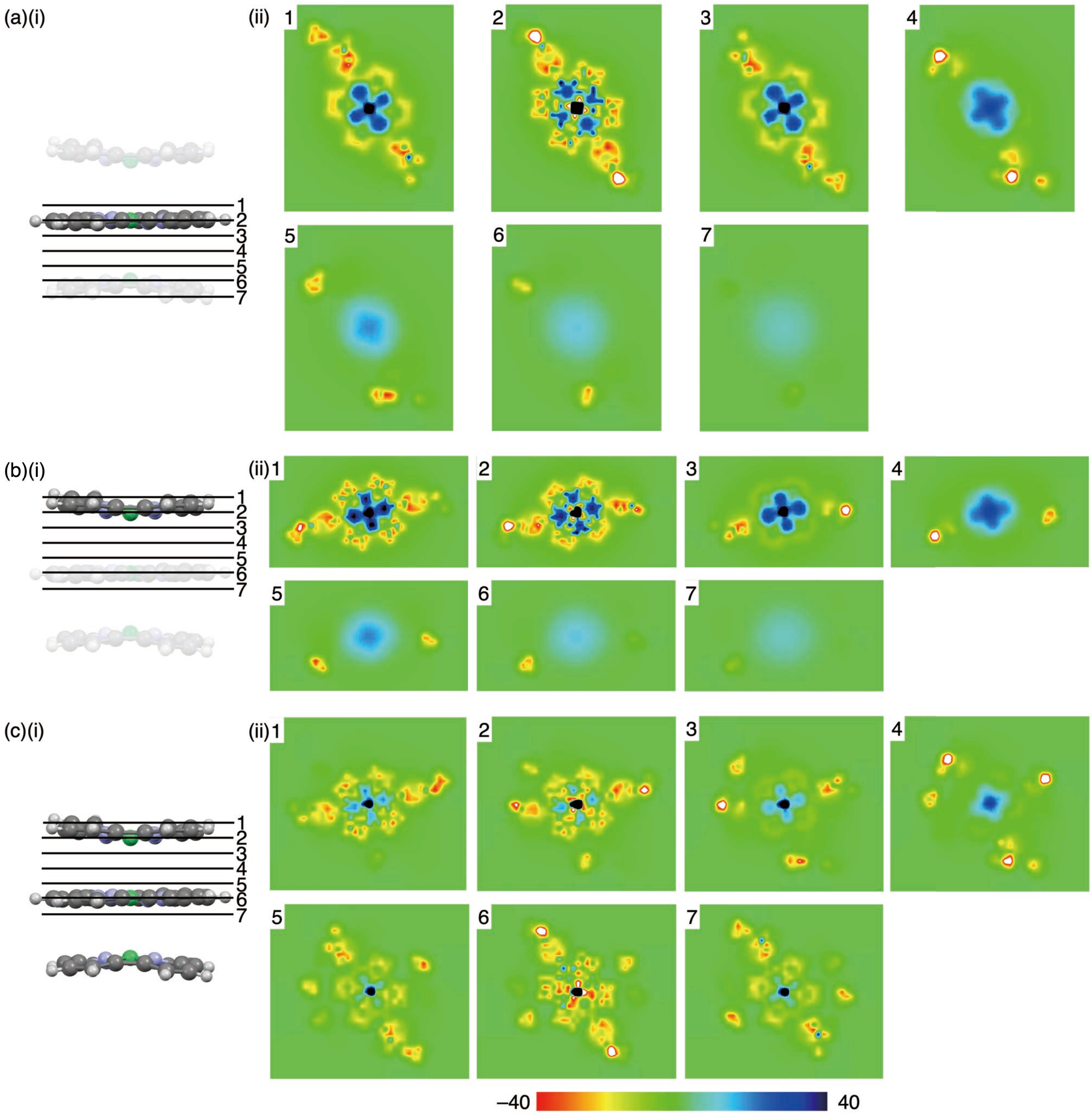

Fig. S47 (i) Arrangements in the crystal structure of **1b**, in which butoxy groups are replaced with methoxy groups, and (ii) 2D NICS plots as (a) monomer (center), (b) monomer (top), and (c) stacked trimer at BHLYP/6-31G(d) with BHLYP/SDD for Ni.[S18] The plots were obtained at the plane indicated in the crystal structure. *Meso*-aryl groups in (i) were omitted for clarity. Smaller positive NICS values around nickel center in (c) indicated that the antiaromaticity of each norcorrole unit was weakened.

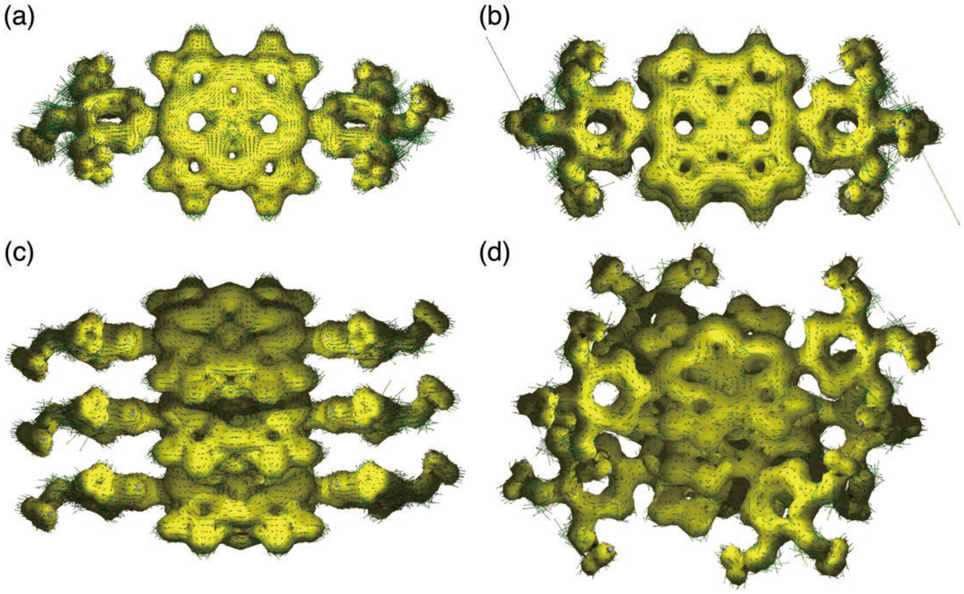

Fig. S48 Anisotropy of the induced current density (ACID)[S20] of (a) **1a** (optimized monomer structure, Fig. S36), (b) **1a** dimer (optimized structure, Fig. S37), (c) **1a** trimer in the crystal structure (Fig. S28), and (d) **1b** trimer, in which butoxy groups are replaced with methoxy groups, in the crystal structure (Fig. S30) at BHLYP/6-31G(d) with BHLYP/SDD for Ni. The arrows indicating current density in (b) and (d) (around *meso*-positions of the central norcorrole unit) were inverted, indicating contribution of the stacked-ring aromaticity.

Cartesian Coordination of 1a

-2231.1678447 hartree
C,-0.6877964781,-2.4150785322,0.9275129617
C,-1.7882449923,-3.1909279031,1.3989818742
H,-1.7391972838,-4.1546046423,1.8873603384
C,-2.9468322702,-2.457816751,1.1280516353
H,-3.9567772601,-2.7477702642,1.3810324244
C,-2.5554053004,-1.2325893397,0.4803122794
C,-3.2694077266,-0.0651063468,0.0428014586
C,-2.613554614,1.1303178617,-0.4131685724
C,-3.0697533104,2.3385343644,-1.0494407008
H,-4.0966566119,2.5886598385,-1.2752016031
C,-1.9482555114,3.1171287492,-1.350138235
H,-1.9500805936,4.0819099341,-1.8388315522
C,-0.8064940207,2.3857943166,-0.9080607258
C,-4.7467760002,-0.09461408,0.0614367653
C,-5.4360458982,-1.2084203445,-0.4482666554
H,-4.8694429397,-2.0219804309,-0.8800654585
C,-6.8341318818,-1.230140817,-0.4453588486
C,-6.9218290161,-3.3712802686,-1.4914004511
H,-6.299422092,-3.1000067242,-2.3537898237
H,-7.7072366486,-4.0552300326,-1.8162134789
H,-6.2957703682,-3.8704646263,-0.7410866731
C,-7.5584901161,-0.1529433899,0.1016228755
C,-9.6327340216,0.5726152799,-0.7711591381
H,-9.4209472508,1.6371212792,-0.6270215539
H,-10.6931011418,0.3853773652,-0.5904999162
H,-9.3846293365,0.2809166861,-1.7990575303
C,-6.8627868548,0.9572411083,0.6186431393
C,-7.0109880166,3.073933198,1.7157070036
H,-6.3747302699,2.7861597107,2.561689305
H,-7.8147997998,3.7190369605,2.0731372764
H,-6.4063468319,3.6218315272,0.98190169
C,-5.4661808571,0.9919614157,0.5887420158
H,-4.922133421,1.8296370963,1.0030834692
N,-1.1902959785,-1.289128278,0.3879741607
N,-1.2501571525,1.2408270416,-0.356266742
O,-7.5904848773,-2.2425802475,-0.9490594002
O,-8.9204107877,-0.2311025827,0.1734831984
O,-7.6479736909,1.9450873045,1.1346173547
Ni,-0.0000086537,0.0000030624,0.0000154435
C,0.6877776581,2.4150872008,-0.927476341
C,1.7882249184,3.1909336102,-1.3989522642
H,1.7391765283,4.1546057853,-1.8873394094
C,2.9468158542,2.4578421748,-1.1279849735
H,3.9567627728,2.7478062282,-1.3809460408
C,2.5553871771,1.2325984023,-0.4802777291
C,3.2693913838,0.065112693,-0.0427755088
C,2.6135383575,-1.1303090472,0.4132054699
C,3.0697309432,-2.3385053019,1.0495214543
H,4.0966302479,-2.5886168489,1.2753150209
C,1.9482357102,-3.1171187009,1.350180402
H,1.9500608328,-4.0819012962,1.8388709769
C,0.8064757765,-2.3857845385,0.9081003503
C,4.7467597541,0.0946378796,-0.0613908913
C,5.4360082528,1.2084351389,0.4483618845
H,4.8693886007,2.0219679333,0.880190348
C,6.8340936728,1.2301789478,0.4454672072
C,6.9217462889,3.3712729885,1.4916054495
H,6.2993378913,3.0999476447,2.3539773962
H,7.7071396115,4.0552210283,1.8164564343
H,6.2956840722,3.8704825208,0.7413109722
C,7.558475057,0.1530166178,-0.1015530283
C,9.6327297588,-0.572553463,0.7711982424
H,9.4209285228,-1.637052305,0.6270307645
H,10.6930950061,-0.3853223619,0.5905203936

H,9.3846524062,-0.2808853861,1.7991122654
C,6.8627937053,-0.9571556259,-0.6186296264
C,7.0110378627,-3.0737922331,-1.7157950358
H,6.3747797159,-2.7859902558,-2.5617678333
H,7.8148626124,-3.7188656633,-2.0732511814
H,6.4064016226,-3.6217358078,-0.9820193676
C,5.4661883026,-0.991900726,-0.5887414792
H,4.9221592628,-1.8295629945,-1.0031338832
N,1.1902780774,1.2891353386,-0.387941086
N,1.2501402529,-1.2408193988,0.3563025631
O,7.5904255347,2.2426106314,0.9492153208
O,8.9203943401,0.2312046391,-0.1734000488
O,7.6480005315,-1.9449629474,-1.1346487975

Cartesian Coordination of 1a dimer

-4460.7254256 hartree
H,-6.0361823474,-5.3874203647,1.01712393
H,-7.4752518992,-5.8219006002,0.0394736242
H,10.7520528537,1.4025309336,-0.9460655168
H,10.6052226322,-1.8896857937,2.3334446813
H,6.0355177703,5.3882109421,-1.016825764
H,7.4746530194,5.8227647486,-0.0393128097
C,0.6824889945,-0.1356638076,-3.1695243612
C,-0.716109043,2.5999166649,1.2427776046
C,1.8016876559,-0.337166322,-4.060131387
C,-1.8562490275,3.0231910046,2.0127982961
C,2.9359818958,0.1286869049,-3.4100476743
C,-2.9982692068,2.583694694,1.346886592
C,2.537625036,0.622252938,-2.0872737927
C,-2.5803405807,1.8846896779,0.1307479247
C,3.2593665658,1.3185639533,-1.0498072313
C,-3.2930773019,1.2460950779,-0.9477445719
C,2.5648953249,1.9424502083,0.049788053
C,-2.5902803277,0.5672204711,-2.0088879909
C,3.0045382392,2.6548864523,1.2503300021
C,-3.018166659,0.0630288461,-3.3184104538
C,1.8745475569,3.0689244332,1.9519826405
C,-1.8943124571,-0.3786241631,-4.0029603974
C,0.7208038983,2.6161280864,1.2200325281
C,-0.7530107387,-0.1509359462,-3.1477219862
C,4.7200884838,1.4532099057,-1.1835710477
C,-4.7608104701,1.3490843583,-1.0349688531
C,5.4906718503,0.3658332834,-1.6438674397
C,-5.4221760988,2.5459579197,-0.6844237803
C,6.878647128,0.4820722283,-1.7889194022
C,-6.8212791407,2.6256933094,-0.7530158106
C,7.0584802067,-1.7294984615,-2.678101744
C,-6.8264077435,4.8931784574,-0.009101889
C,7.5398092394,1.6842121018,-1.438929355
C,-7.5829439396,1.5130299584,-1.177745192
C,9.7367601048,1.020200165,-0.7912558462
C,-9.5188349548,1.8552192851,-2.47590446
C,6.7617067572,2.7748977459,-0.9764786227
C,-6.9144165856,0.3312729003,-1.5658063082
C,6.7322991757,5.042574027,-0.2366724915
C,-7.0899623117,-1.8906876926,-2.4316204305
C,5.3673283821,2.6665354307,-0.8658483416
C,-5.5195105463,0.2457544545,-1.4776052819
H,1.7566204598,-0.757691178,-5.0595591678
H,-1.8295409551,3.567076107,2.9510688017
H,3.942669591,0.1542598256,-3.8095516278
H,-4.0211769701,2.7315079972,1.6711176587
H,4.0337145222,2.8323472183,1.5378003823
H,-4.0372020842,0.0631415551,-3.6863228627
H,1.8648171874,3.6153929015,2.8890976459
H,-1.8708628839,-0.8000459711,-5.0027676726
H,4.9960162525,-0.5787800767,-1.8301857353
H,-4.8284282063,3.4114466558,-0.4110181146
H,6.5430391956,-2.2167264164,-1.8388503114
H,-6.1463354329,5.2596867255,-0.7944151157
H,9.459174038,1.0927184525,0.2672236339
H,-9.265103645,1.0581442166,-3.1898629378
H,6.5294636881,2.3551785984,1.6091272055
H,4.7661265622,3.5189024419,-0.5685574846
H,-5.0092255919,-0.6865608075,-1.6822109758
H,7.8685318898,-2.3744353841,-3.0326005871
H,-7.5818099014,5.6559586717,0.2052160987
H,6.3404796145,-1.5513701039,-3.4949844712
H,-6.2410987432,4.6917301093,0.9029081198
H,9.6746854154,-0.0280448335,-1.1034929968
H,-9.168125072,2.8251435491,-2.8612011163
H,7.9015889586,2.5558366074,2.741813999
H,6.1606305258,4.8412612882,0.6850308555
H,-6.4135462493,-1.7049728925,-3.2811848869
N,1.1955964611,0.4219008276,-2.0148605131
N,-1.219975748,1.9264443173,0.1451570227
N,1.2051998304,1.9522458396,0.107745337
N,-1.2426526605,0.3966724649,-1.9782586487
Ni,-0.0133680245,1.0728564664,-0.86265598
O,7.6848103959,-0.5166076131,-2.2660786189
O,-7.5504199544,3.7411824907,-0.4340871281
O,8.8842823421,1.8652894598,-1.5975347739
O,-8.9553369709,1.5879810992,-1.1811466122
O,7.4732443775,3.9025911796,-0.6595536077
O,-7.7241545899,-0.6844674317,-2.0025980304
C,0.7159639328,-2.5997865207,-1.242901268
C,-0.6823376225,0.1358630001,3.1694783611
C,1.8560529957,-3.0229889243,-2.0130358815
C,-1.8015015512,0.3374683021,4.0600960685
C,2.9981178787,-2.5835329039,-1.3471686965
C,-2.935857577,-0.1282728044,3.4100258052
C,2.5802691795,-1.8846241974,-0.1309548543
C,-2.5375479475,-0.6219694741,2.0872914328
C,3.2930799431,-1.2460451079,0.9475229363
C,-3.2593745856,-1.3182020293,1.0498049546
C,2.5903619709,-0.5671670122,2.0086885681
C,-2.5649735705,-1.9421478926,-0.0497860779
C,3.0183398637,-0.0628493433,3.318139365
C,-3.0046864284,-2.6544301922,-1.2504012788
C,1.8945302309,0.3787980612,4.002755344
C,-1.8747400269,-3.0685199749,-1.9520918911
C,0.753162007,0.1510509169,3.1476088193
C,-0.7209481432,-2.6159281309,-1.2200937642
C,4.7608268157,-1.3490238127,1.0346406794
C,-4.7201116836,-1.4526556772,1.1835406443
C,5.4221784824,-2.5458726896,0.6839711969
C,-5.4905576651,-0.3651567822,1.6438035118
C,6.8212901429,-2.6255762114,0.7524187076
C,-6.8785522221,-0.4811981566,1.7888066857
C,6.8264130954,-4.8929603669,0.0081969932

C,-7.0581089391,1.7304164947,2.6779072136
C,7.5829797964,-1.5129031799,1.1770749168
C,-7.5398683096,-1.6832565074,1.4388111175
C,9.518990525,-1.8558657141,2.4747932768
C,-9.7366393172,-1.019279759,0.7906715282
C,6.9144635231,-0.3312259026,1.5653750659
C,-6.7619132219,-2.774037275,0.9763834586
C,7.0900375285,1.8906786277,2.4313044138
C,-6.7327928718,-5.041803555,0.2368091854
C,5.5195473398,-0.2457148693,1.4772806099
C,-5.3675124395,-2.665887312,0.8658094887
H,1.8292798982,-3.566805235,-2.9513445154
H,-1.7563919085,0.7580210264,5.0595101629
H,4.0210091428,-2.7312543355,-1.6714873107
H,-3.9425448122,-0.1537204152,3.8095406375
H,4.0374027005,-0.0629102142,3.6859756793
H,-4.0338695369,-2.8318012205,-1.5379041839
H,1.8711491624,0.8002864575,5.0025356987
H,-1.8650763743,-3.6148821444,-2.8892690956
H,4.8284178424,-3.4113463717,0.4105469105
H,-4.9957507616,0.5793816229,1.8301072704
H,6.1463731746,-5.2596199902,0.7934661876
H,-6.542708472,2.2175981487,1.8385978901
H,9.2651635307,-1.059369127,3.1893722269
H,-9.4595494393,-1.0930912862,-0.2678525288
H,-6.5293837983,-2.3551401614,-1.6094219041
H,5.0092882146,0.6865880224,1.6819968877
H,-4.7664264091,-3.5183622407,0.5685949956
H,7.581812052,-5.6556972159,-0.2062855175
H,-7.868058901,2.3754238438,3.0325125091
H,6.2410705973,-4.6913358327,-0.9037519183
H,-6.3400324337,1.5522242082,3.4947072882
H,9.1684569788,-2.8261372109,2.8593703748
H,-9.6737232637,0.0292437796,1.1017942243
H,-7.9015006106,-2.5558783768,-2.7420916726
H,6.4136261759,1.704931193,3.2808641497
H,-6.1609564781,-4.8407611204,-0.6848494801
N,1.2199060657,-1.926402227,-0.1452605003
N,-1.1955140627,-0.4217344938,2.0148511764
N,1.2427219237,-0.3966521457,1.9781626256
N,-1.2052759088,-1.9521096388,-0.1077337194
Ni,0.0133718564,-1.0728134194,0.862639528
O,7.5504338111,-3.7410338019,0.4333669036
O,-7.6845948814,0.5175956145,2.2659307536
O,8.9553800516,-1.5877102404,1.1802772298
O,-8.8843858473,-1.8641238847,1.5974514362
O,7.7242170113,0.6844686665,2.0022239177
O,-7.4736043554,-3.9016234717,0.6593861121
H,-10.6050668905,1.8893276806,-2.3346276575
H,-10.7521271875,-1.4007353405,0.9463381643

Cartesian Coordination of 1a trimer

-6691.1505559 hartree
C,7.2858407229,0.2255302726,20.7588802214
C,8.0356538751,-0.3360793593,21.8490929492
C,9.3824464089,-0.2704902246,21.4974015727
C,9.4768589122,0.3460299314,20.1817179532
C,10.5768515848,0.4759152308,19.2605571541
C,4.676109809,0.5396762572,18.3760655627
C,3.8392335531,-0.0519154431,19.4096970703
C,4.6130238064,-0.2037173486,20.5597550598
C,5.9302577868,0.2779948899,20.2491607106
C,11.9258404563,0.1331195203,19.7433537994
C,14.5134246538,-0.5236254773,20.6866153476
C,14.08844102,-0.943342553,19.4040212898
C,12.7998212937,-0.6340264724,18.9432243707
H,7.6243015027,-0.757943255,22.7605329485
H,10.2190629263,-0.6412445273,22.0774516325
H,2.8048261492,-0.3499876493,19.2863493411
H,4.2927354401,-0.6332601572,21.5036156964
N,8.1924597149,0.6398375606,19.8150108298
N,5.8998005369,0.7315072661,18.953334669
O,15.7450687167,-0.90032271,21.1455910867
O,15.0156268499,-1.6514166493,18.6855907868
C,7.2706419836,3.425684434,20.9659965377
C,8.0325045076,3.047238987,22.1287951946
C,9.3788483779,3.1350658085,21.7914604961
C,9.4784710695,3.5850253449,20.401247732
C,10.590177802,3.8061281193,19.5060779987
C,4.6693385205,3.7856563867,18.5713157793
C,3.7873024011,3.381968818,19.6690626099
C,4.5599356096,3.2052048754,20.8117951357
C,5.9276913103,3.4815663304,20.4543808261
C,11.9636477867,3.7497265023,20.0389475724
C,12.3463312417,0.5559792337,21.0209614393
C,13.6219709064,0.223351102,21.494308402
C,13.2005696959,1.201870359,23.6293690847
C,14.5903665299,3.5870734359,21.1031106457
C,16.7304705555,0.1559181817,21.1519313627
C,14.3096097404,3.1162512449,19.8005673427
C,-0.2324199143,1.8978422705,18.6680731624
C,13.0119095046,3.1918837914,19.2762128607
H,7.6281297038,2.7296921293,23.0834673203
H,10.2139218866,2.9063357481,22.4410337606
H,2.7148138701,3.2523004511,19.6016404426
H,4.2035782964,2.895259763,21.7878808034
H,11.6898898419,1.1932705564,21.6001183095
H,12.8413198355,2.1620559197,23.2374881293
H,16.9618518008,0.467868088,20.1229943669
H,0.3882541688,2.8026847574,18.6274765044
H,2.2011802191,1.6150501935,18.0904644539
H,13.7741737747,1.3768158856,24.5444430579
H,12.339599826,0.5483193029,23.8443916126
H,16.3844280149,1.026465139,21.720379776
H,-1.2333686173,2.1569361241,19.026778691
H,0.2310724667,1.1657548056,19.3484183745
N,8.1924452712,3.7325683629,19.9848849309
N,5.9148658636,3.8220117471,19.1163580781
Ni,7.5068809827,1.0395714771,18.195141413
O,14.1055920993,0.5740253391,22.7222340739
O,15.8125179731,3.4053189192,21.6938770243
O,15.3805355629,2.5774495688,19.1316749121
C,7.2983086076,6.6013947795,21.1598890329
C,8.0496746915,6.4828683118,22.3757880482
C,9.40090342,6.6112090492,22.0429808873
C,9.4961649882,6.8318765984,20.6077553137
C,10.5809275706,7.1191206937,19.7121275405
C,4.6726620246,7.040169723,18.7952100028
C,3.7835299091,6.8645637127,19.9330883976
C,4.5674807245,6.6424101091,21.0689175088

C,5.9388406038,6.6587751036,20.6494966826
C,11.9472258511,7.2971068697,20.2336454255
C,12.2475486055,4.2487616,21.3281613477
C,13.5446178004,4.1733234126,21.852387928
C,12.8828248154,5.1967947763,23.9028382656
C,14.580502972,7.5649522405,21.2247762982
C,16.8740368807,4.265642297,21.2297528262
C,14.346466245,6.9484955298,19.9760411031
C,-0.0967220356,5.8348039813,18.8398713691
C,13.0406957152,6.800429057,19.4959331545
H,7.6448870062,6.3098092398,23.3673702115
H,10.23221889,6.5571555791,22.7347298922
H,2.7016743414,6.9044111634,19.9046937351
H,4.2059801015,6.4741350162,22.0779457193
H,11.460849032,4.7552752292,21.872421697
H,12.4450566504,6.0843285732,23.4249813668
H,17.0576080295,4.1196141645,20.1570438696
H,0.6292994649,6.6570778165,18.7899207153
H,2.2684861634,5.1246160266,18.3553996679
H,13.3732440793,5.4890462049,24.8366904562
H,12.0819621519,4.4692742058,24.116629999
H,16.6350959646,5.3170820325,21.4283815871
H,-1.0627055407,6.2201830783,19.181123737
H,0.2648432953,5.0682915548,19.54492207
N,8.2062243409,6.7848175098,20.1495960624
N,5.9362008376,6.8747182949,19.2957022844
Ni,7.5219598381,3.9318197061,18.3384119945
O,13.9062544127,4.6272823743,23.0922939235
O,15.8467537153,7.6401410657,21.754462863
O,15.4648977196,6.5176306836,19.3127806918
C,12.1798553885,7.958457585,21.4584842104
C,13.4864142722,8.0867470663,21.9524199693
C,12.7455148486,9.2743014994,23.8848243448
C,16.6458087372,8.7040063827,21.208669169
C,0.4237359855,9.9887234255,19.2732654233
H,11.3396461209,8.4021241218,21.9808868691
H,12.2217916437,10.0623815556,23.3208669124
H,16.7865908963,8.5776618061,20.1256973942
H,1.2755268953,10.6838736777,19.2053839341
H,2.5997495803,8.8684311345,18.6874582841
H,13.2113255703,9.7093281264,24.774803695
H,12.0133768626,8.5101039479,24.1922042474
H,16.1812647125,9.6807287521,21.4131336738
H,-0.4465208448,10.5170405611,19.6759944566
H,0.6945286255,9.1607753897,19.9496767753
Ni,7.5373131348,6.8240596963,18.4815729818
O,13.8128957069,8.7064548509,23.1291354511
H,17.6229557494,-0.276083596,21.6186948906
H,17.7608213994,3.9648872518,21.7994675048
H,17.6154665314,8.646510651,21.7156696098
C,7.75862886,7.6380177354,15.9178403401
C,7.0089588045,8.1996980107,14.8275698891
C,5.66212612,8.1342168034,15.1791509507
C,5.5675523718,7.5176962277,16.4948234067
C,4.4674566109,7.3877560824,17.4158535018
C,10.3681444798,7.3237388605,18.3008796853
C,11.2051356905,7.9152979384,17.2673246366
C,10.4314473451,8.0671427953,16.1171986065
C,9.114174299,7.5854622457,16.4276737559
C,3.1184813664,7.730292287,16.9328279329
C,0.5311474743,8.3867044651,15.9887931894
C,0.955575312,8.8062675944,17.2716064415
C,2.2440759217,8.4970807753,17.7328380442
H,7.4204189037,8.6215551355,13.9161757192
H,4.8256088481,8.5050808621,14.5990275274
H,12.2395438264,8.2133317565,17.3907608265
H,10.7518371111,8.4966715454,15.1733662553
N,6.8519068791,7.2237865346,16.8616322491
N,9.1444919347,7.1319551791,17.7234990987
O,-0.7002228331,8.7635203794,15.5291366808
O,0.0280158182,9.5141353101,17.9897668355
H,-2.5715905811,-0.7829214071,14.9618520582
H,-2.7171000633,3.8985579319,14.8781061263
H,-2.5784065177,8.1395980132,15.0567777686
C,2.8640315291,-0.0952007454,15.218719653
C,1.5574087367,-0.2234463802,14.7249397106
C,2.2980088433,-1.4112457081,12.792569438
C,-1.6020315151,-0.8402535334,15.4690595508
C,14.6194093304,-2.1259433704,17.4022249523
H,3.7041519283,-0.538964974,14.6962593465
H,2.8217484576,-2.1992954652,13.3565529061
H,-1.7430094794,-0.7135378057,16.5519620909
H,13.7675414742,-2.8209730931,17.4703816598
H,12.4437722583,-1.005423609,17.9887636941
H,1.8320634683,-1.8463435445,11.9026960155
H,3.0301588607,-0.6471310707,12.4850132027
H,-1.1374602932,-1.8170489779,15.2650151646
H,15.4894559133,-2.6543859414,16.9992068606
H,14.3485111126,-1.2979526813,16.7259082948
O,1.2307556846,-0.8432437956,13.5483199103
C,7.7456551548,1.262117079,15.516788325
C,6.9941947179,1.3804888335,14.300941471
C,5.6430037198,1.2520793509,14.6338525193
C,5.5478611911,1.0315132003,16.0691024301
C,4.4631880957,0.7442530012,16.9648360826
C,10.3715032659,0.8234944904,17.8812294567
C,11.2605280931,0.9989492145,16.7432419412
C,10.4764743339,1.2210802535,15.6074919991
C,9.1051565205,1.2048591691,16.0270674331
C,3.096834277,0.5662415634,16.4434722972
C,2.796335895,3.6145757007,15.3486944027
C,1.4992543182,3.6899685061,14.8244980219
C,2.160976734,2.6663108791,12.7741236583
C,0.4634373606,0.2985181845,15.4526384962
C,-1.8302284789,3.5977752219,15.4476664802
C,0.6976451306,0.9150405952,16.7013048967
C,15.140702883,2.0288214927,17.8368932966
C,2.0034761782,1.0630364519,17.1812704558
H,7.3988984978,1.5534839804,13.309315484
H,4.8116528088,1.3059446585,13.9421324704
H,12.3423808759,0.9589330824,16.7715166111
H,10.8378700297,1.389235959,14.5984074994
H,3.5829939009,3.1079197088,14.8045141599
H,2.5987978726,1.7788506491,13.252063534
H,-2.0134900706,3.7440966841,16.5203818175
H,14.4146657039,1.2065693268,17.8869174118
H,12.7755473508,2.7391130878,18.3212802521
H,1.6705162096,2.3739302312,11.8403339377
H,2.9618130438,3.3938209525,12.5601958512
H,-1.5914909414,2.546251563,15.2492165037

H,16.1066775811,1.6433911293,17.4956762479
H,14.7791663503,2.7952931281,17.1317830847
N,6.8378262684,1.0786883473,16.5271638606
N,9.1079292257,0.989052469,17.3808783115
O,1.1375801332,3.2358995898,13.5846472675
O,-0.8028642491,0.2234224708,14.9230524293
O,-0.4206860142,1.346041906,17.3646475955
C,7.7732726432,4.4378102176,15.7107917883
C,7.0113928476,4.8159997257,14.5479153241
C,5.6650512142,4.7280823699,14.8852358444
C,5.5654527743,4.2783900363,16.2755318661
C,4.4537477449,4.057352091,17.170704682
C,10.3745772388,4.077908529,18.1054878217
C,11.2566125087,4.4815750924,17.0077314207
C,10.4839828601,4.6583108435,15.864997105
C,9.1162235367,4.3819669852,16.222415445
C,3.0802804041,4.113750622,16.6378408097
C,2.6984497078,7.3074148699,15.6550679726
C,1.4228428771,7.6397036549,15.1814015258
C,1.844678152,6.6605586578,13.0467526774
C,0.453518514,4.2762429337,15.5737765168
C,-1.6860880824,7.707735623,15.5240004541
C,0.7343470264,4.7473157161,16.876218577
C,15.276814111,5.9659760011,18.0092637893
C,2.0320631782,4.671742766,17.4005313953
H,7.4157574239,5.1333707853,13.5931811257
H,4.8299067018,4.9564183095,14.2356134948
H,12.3290887997,4.6113034182,17.0751818935
H,10.8403427654,4.968223998,14.8889036486
H,3.355588507,6.6708365653,15.075933591
H,2.204164308,5.7007210976,13.4392772201
H,-1.9176279544,7.3969767458,16.5532699906
H,14.6561960496,5.061087498,18.0496696177
H,12.8431632154,6.2484652775,18.5866718464
H,1.2711466155,6.4848795533,12.1317750542
H,2.7054938077,7.3141839059,12.8313288629
H,-1.3404012443,6.8364754941,14.9564096009
H,16.2778211989,5.7069917751,17.6506429501
H,14.8133569797,6.6981225228,17.3289583911
N,6.8514812629,4.1310297664,16.6919391336
N,9.1290465574,4.0415780462,17.5604482587
O,0.9394755767,7.2887458686,13.9534699085
O,-0.7686695187,4.4578019776,14.9830448624
O,-0.336536458,5.2862863259,17.5450498208

[S17 (the complete form of ref. 8)] M. J. Frisch, G. W. Trucks, H. B. Schlegel, G. E. Scuseria, M. A. Robb, J. R. Cheeseman, G. Scalmani, V. Barone, G. A. Petersson, H. Nakatsuji, X. Li, M. Caricato, A. V. Marenich, J. Bloino, B. G. Janesko, R. Gomperts, B. Mennucci, H. P. Hratchian, J. V. Ortiz, A. F. Izmaylov, J. L. Sonnenberg, D. Williams-Young, F. Ding, F. Lipparini, F. Egidi, J. Goings, B. Peng, A. Petrone, T. Henderson, D. Ranasinghe, V. G. Zakrzewski, J. Gao, N. Rega, G. Zheng, W. Liang, M. Hada, M. Ehara, K. Toyota, R. Fukuda, J. Hasegawa, M. Ishida, T. Nakajima, Y. Honda, O. Kitao, H. Nakai, T. Vreven, K. Throssell, J. A. Montgomery, Jr., J. E. Peralta, F. Ogliaro, M. J. Bearpark, J. J. Heyd, E. N. Brothers, K. N. Kudin, V. N. Staroverov, T. A. Keith, R. Kobayashi, J. Normand, K. Raghavachari, A. P. Rendell, J. C. Burant, S. S. Iyengar, J. Tomasi, M. Cossi, J. M. Millam, M. Klene, C. Adamo, R. Cammi, J. W. Ochterski, R. L. Martin, K. Morokuma, O. Farkas, J. B. Foresman and D. J. Fox, *Gaussian 16*, Revision C.01, Gaussian, Inc., Wallingford CT, 2016.

[S18] (a) P. v. R. Schleyer, C. Maerker, A. Dransfeld, H. Jiao and N. J. R. van Eikema Hommes, *J. Am. Chem. Soc.*, 1996, **118**, 6317–6318; (b) T. Lu and F. Chen, *J. Comput. Chem.*, 2012, **33**, 580–592.

[S19] (a) R. R. Valiev, H. Fliegl and D. Sundholm, *Chem. Commun.*, 2017, **53**, 9866–9869; (b) S. Lehtola, M. Dimitrova, H. Fliegl and D. Sundholm, *J. Chem. Theory Comput.*, 2021, **17**, 1457–1468.

[S20] (a) R. Herges and D. Geuenich, *J. Phys. Chem. A*, 2001, **105**, 3214–3220; (b) D. Geuenich, K. Hess, F. Köhler and R. Herges, *Chem. Rev.*, 2005, **105**, 3758–3772.

4. Assembled behaviors

Differential scanning calorimetry (DSC). The phase transitions were measured on a differential scanning calorimetry (Shimadzu DSC-60).

Polarizing optical microscopy (POM). POM observations were carried out with a Nikon ECLIPSE LV100N-POL polarizing optical microscope equipped with a Mettler-Toledo HS82 hot stage system.

Synchrotron X-ray diffraction analysis (XRD). High-resolution XRD analyses were carried out using a synchrotron radiation X-ray beam with a wavelength of 1.00 Å on BL40B2 at SPring-8 (Hyogo, Japan). The diffractions were detected by a large Debye-Scherrer camera with a Pilatus3S 2M (Dectris Ltd., Switzerland) as a detector. The camera lengths were set at 429.5 mm for **1c**, 431.0 mm for VT-XRD of **1d** and **2c**,**e**, 428.2 mm for sheared sample of **1d**, 428.1 mm for VT-XRD of **1e**, and 427.4 mm for VT-XRD of **2d**. The diffraction patterns were obtained with a 0.01° step in 2θ. An exposure time of the X-ray beam was 10 sec.

MD simulation. All-atom molecular dynamics (MD) simulation was performed with the MD program *GROMACS 2016.3*, which is a free program used for fast and large-scale simulations (Fig. 7, Fig. S67–73). For calculating the intra- and intermolecular interactions of the molecules, generalized Amber force field[S21] parameters were used for accurate calculations applicable to various systems.[S22,23] To reproduce triple deckers of **1d**, the harmonic restraints were applied to nickel atoms between the top and middle molecules and the bottom and middle molecules, and the van del Waals radii of the atoms forming the center norcorrole and the harmonic force constants applied to the angle potential with respect to the nickel atoms were scaled by a factor of 0.95 and 0.1, respectively: the previously reported crystal structure of *meso*-phenyl-substituted Ni^{II} norcorrole was successfully reproduced by MD simulation with these modified parameters. The partial atomic charges of the simulated **1d** were obtained by applying the restrained electrostatic potential (RESP) method,[S24] based on single point DFT calculations based on the *Gaussian 16* revision C01 program[S17] with B3LYP/6-31G(d,p) level for the atoms except nickel, for which the LanL2DZ basis set was used with the associated effective core potential. Each system was composed of 384 molecules. For the initial structures of the hexagonal and rectangular columnar structures, 16 columns were positioned in the hexagonal box of 10.91 nm × 12.60 nm × 7.44 nm and in the rectangular box of 11.24 nm × 12.40 nm × 7.44 nm, respectively. Pre-equilibration runs at each temperature were performed as follows: 5 ns NVT ensemble simulation and 5 ns NPT ensemble simulation one after the other. The temperature and pressure were kept using the Berendsen thermostat and barostat,[S25] respectively, with relaxation times of 0.2 and 2.0 ps. Following the pre-equilibration runs, the equilibration run was performed for 100 ns at the set temperature and 1 bar. The Nosé-Hoover thermostat[S26] and Parrinello-Rahman barostat[S27] were used to maintain the temperature and pressure constant with relaxation times of 1.0 and 5.0 ps, respectively. The time step was set to 2 fs and all covalent bonds connected to hydrogen atoms were constrained by using the LINCS algorithm.[S28] The long-range electrostatic interactions were treated by the smooth particle mesh Ewald with a real-space cutoff of 1.2 nm and a Fourier spacing of 0.30 nm. A cutoff for the van der Waals interactions was set to 1.2 nm.

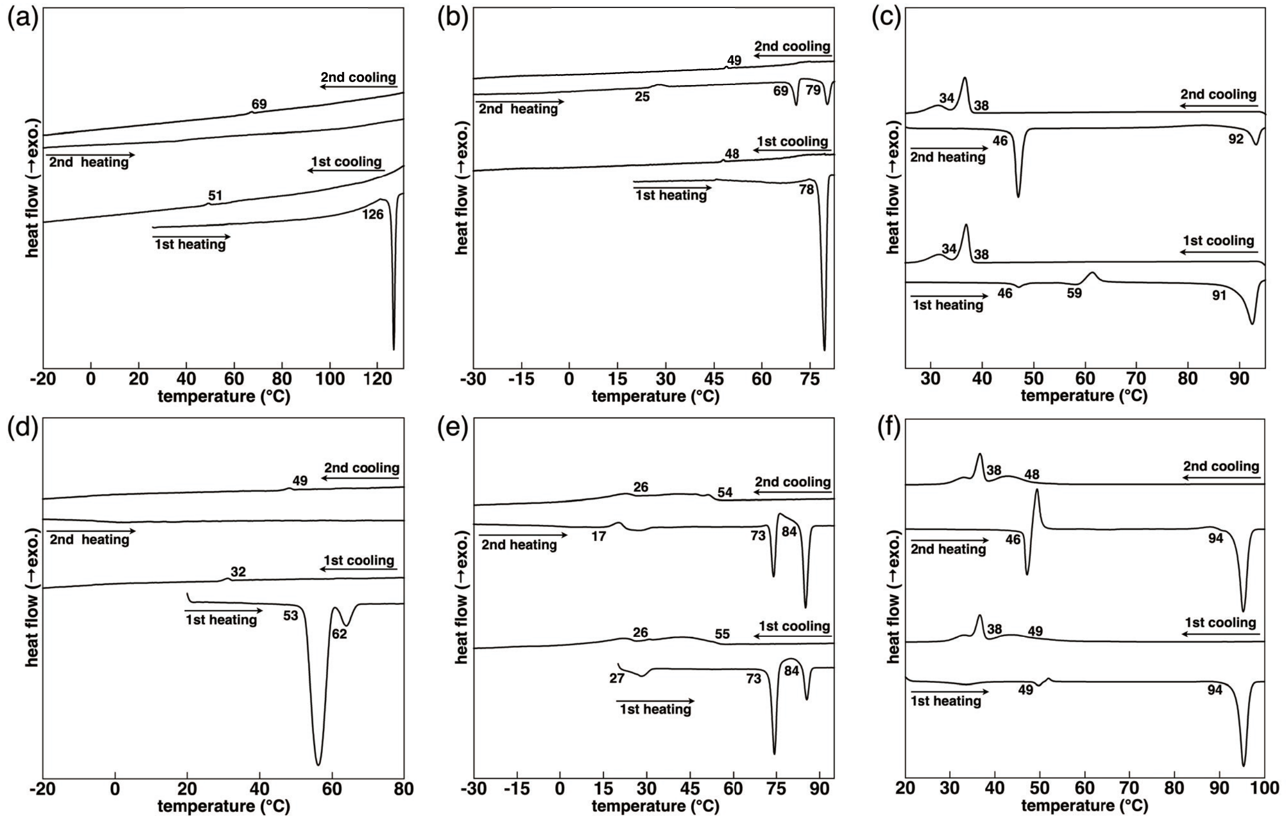

Fig. S49 DSC thermograms of (a) **1c**, (b) **1d**, (c) **1e**, (d) **2c**, (e) **2d**, and (f) **2e** (scan rate: 5 °C/min except for **1d** at 2 °C/min, which provided the transitions that are more consistent with those in XRD). Onset temperatures (°C) of phase transitions are labeled.

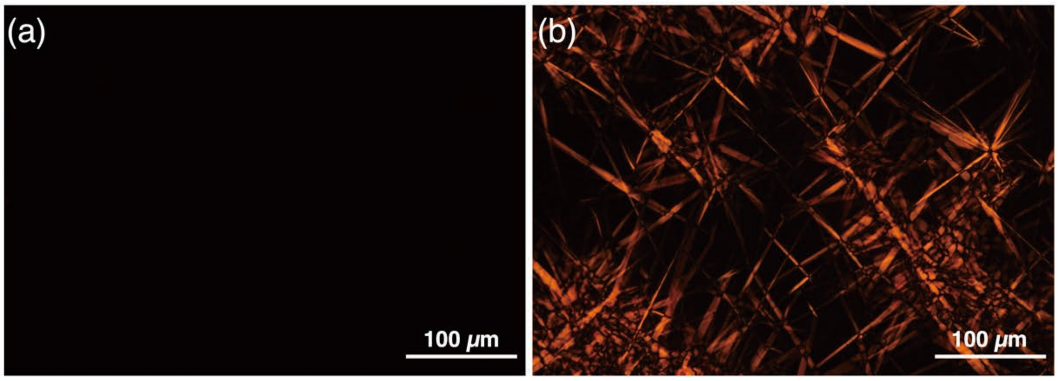

Fig. S50 POM textures of **2c** at (a) 30 °C upon cooling and (b) 55 °C upon heating after annealing at 15 min as needle-shaped crystal-like textures.

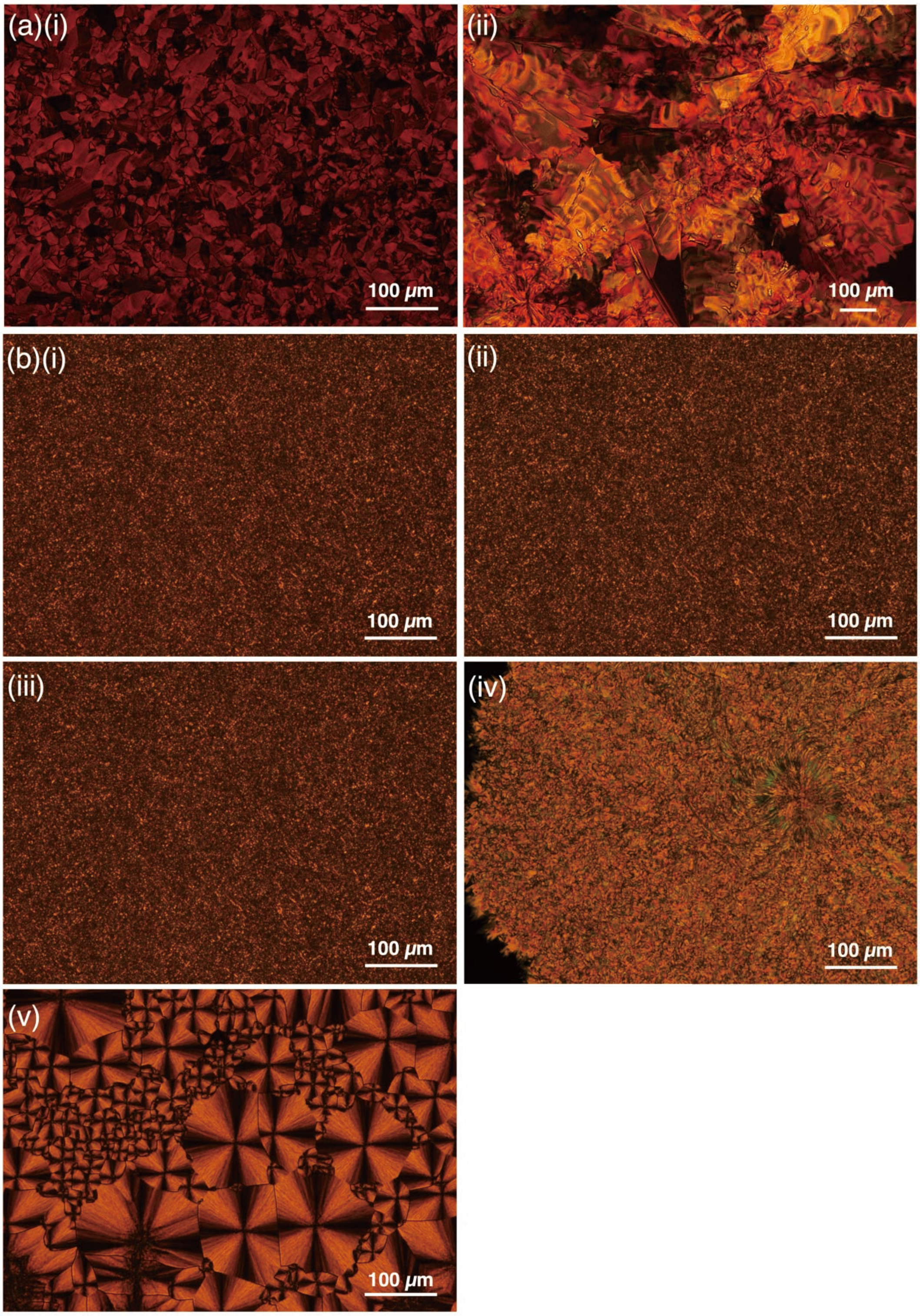

Fig. S51 POM textures of (a) **1d** at (i) 40 °C upon cooling after annealing for 30 min as a mosaic texture and (ii) 75 °C upon heating after annealing for 100 min and (b) **2d** at (i) 60 °C upon cooling after annealing for 10 min, (ii) 20 °C upon cooling, (iii) 40 °C upon heating, (iv) 78 °C upon heating after annealing for 20 min, and (v) 35 °C upon cooling without annealing over 55 °C. The Col_r phase in the lower temperature region and Col_h phase of **1d** upon cooling were not observed, as the POM observation was carried out above 20 °C due to the absence of cooling unit.

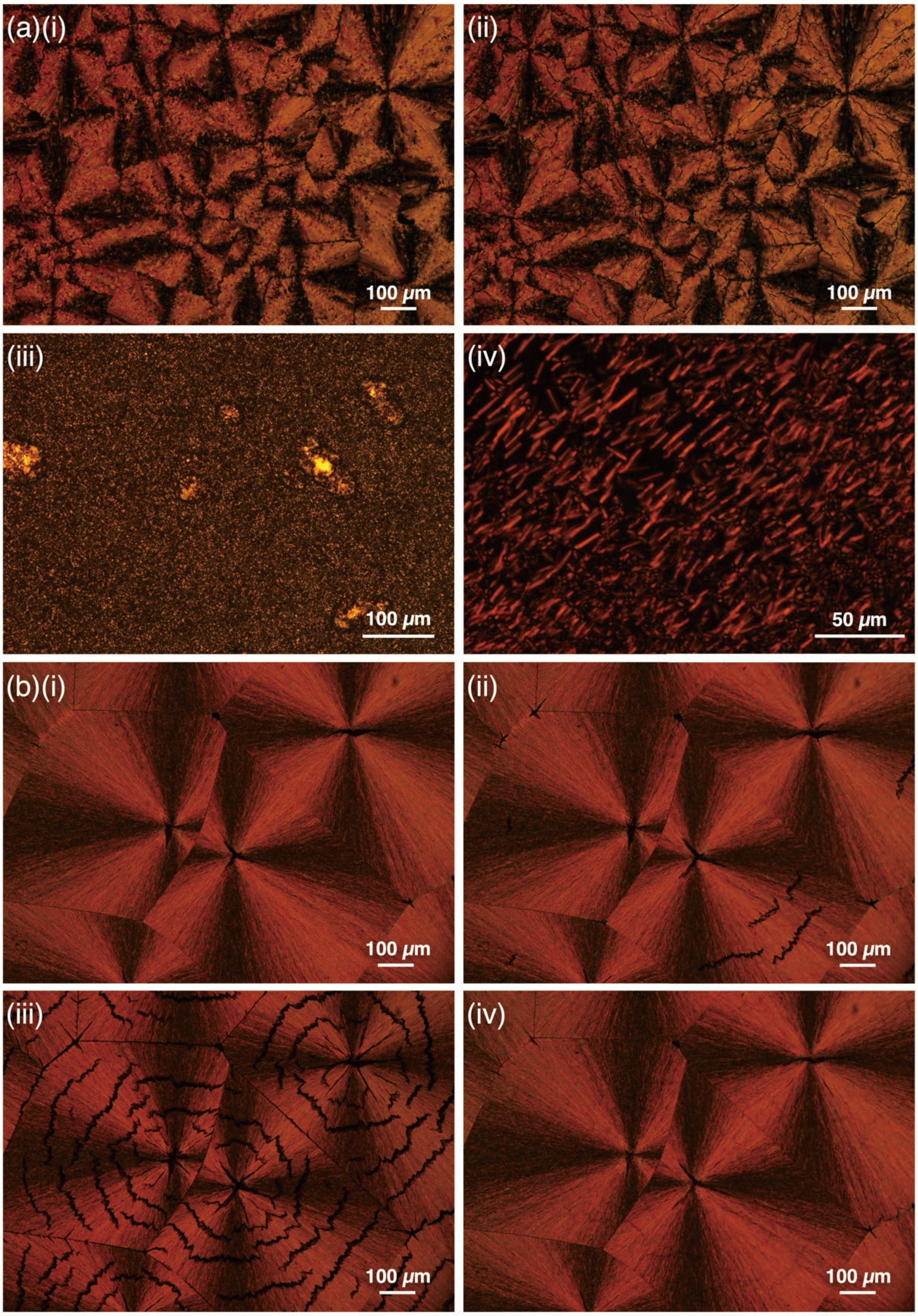

Fig. S52 POM textures of (a) **1e** at (i) 80 °C upon cooling after annealing for 20 min, (ii) 30 °C upon cooling after annealing for 60 min (no phase transition was observed), (iii) 36 °C upon cooling without annealing over 38 °C, and (iv) 50 °C upon heating from (iii) and (b) **2e** at (i) 80 °C upon cooling after annealing for 12 min, (ii) 45 °C upon cooling, (iii) 21 °C upon cooling, and (iv) 80 °C upon heating as focal conic texture. The texture in (a)(iv) was further converted to the focal conic texture shown in (a)(i,ii) at 60 °C upon heating.

Table S2 Summarized phase transition behaviors of **1c**–**e** and **2c**–**e**. The details of the XRD patterns and packing structures are shown in Fig. S54–66 and Table S3–7.

compounds	heating[a]	cooling[a]
**1c**	–[b]	–[b]
**1d**	–[c] 10[d] Col$_r$ 25[d] Col$_h$ 45[d] Col$_r$ 69 –[e] 79 Iso	–[c] –15[d] Col$_r$ 15[d] Col$_h$ 20[d] Col$_r$ 48 Iso
**1e**	lamellar 46 Col$_h$ 60 lamellar 92 Iso	lamellar 34 lamellar 38 lamellar 80[f] Iso
**2c**	–[c] 55[f] –[c] 75[f]	–[c] 32 Iso
**2d**	lamellar 17 lamellar 73 –[c] 84 Iso	lamellar 26 lamellar 55 lamellar 60[f] Iso
**2e**	lamellar 46 lamellar 94 Iso	lamellar 38 lamellar 49 lamellar 80[f] Iso

[a] Transition temperatures (°C, the onset of the peak) from DSC. 1st cooling and 2nd heating scans (5 °C/min) for **1c**,**e** and **2c**–**e**, and 1st cooling and 2nd heating scans (2 °C/min) for **1d**. [b] **1c** has a high melting point at 126 °C, at which **1c** was decomposed on the conversion to an isotropic liquid. [c] The phase showing less ordered diffractions. [d] Transition temperature from XRD. [e] The phase showing complicated diffractions. [f] Transition temperature from POM.

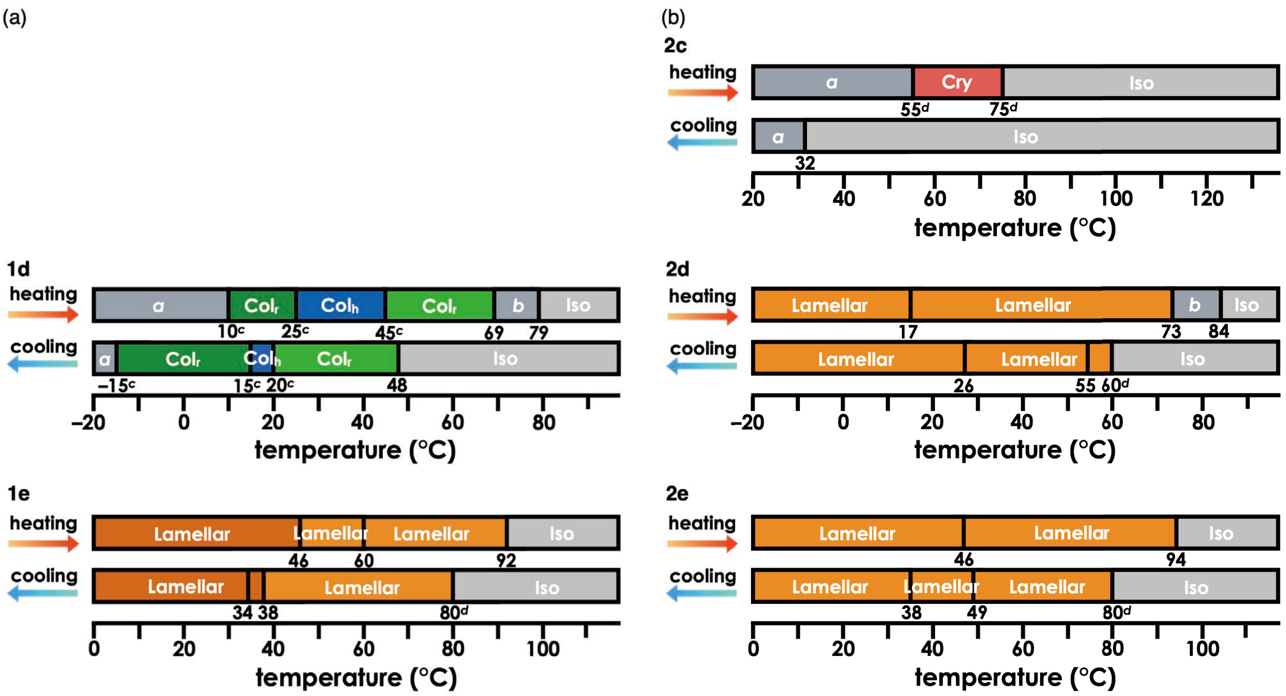

Fig. S53 Details of phase transition behaviors of (a) **1d**–**e** and (b) **2c**–**e** with transition temperatures mainly by DSC without labels and also by XRD and POM labeled with *c* and *d*, respectively. The phases *a* and *b* showing less ordered and complicated diffractions, respectively.

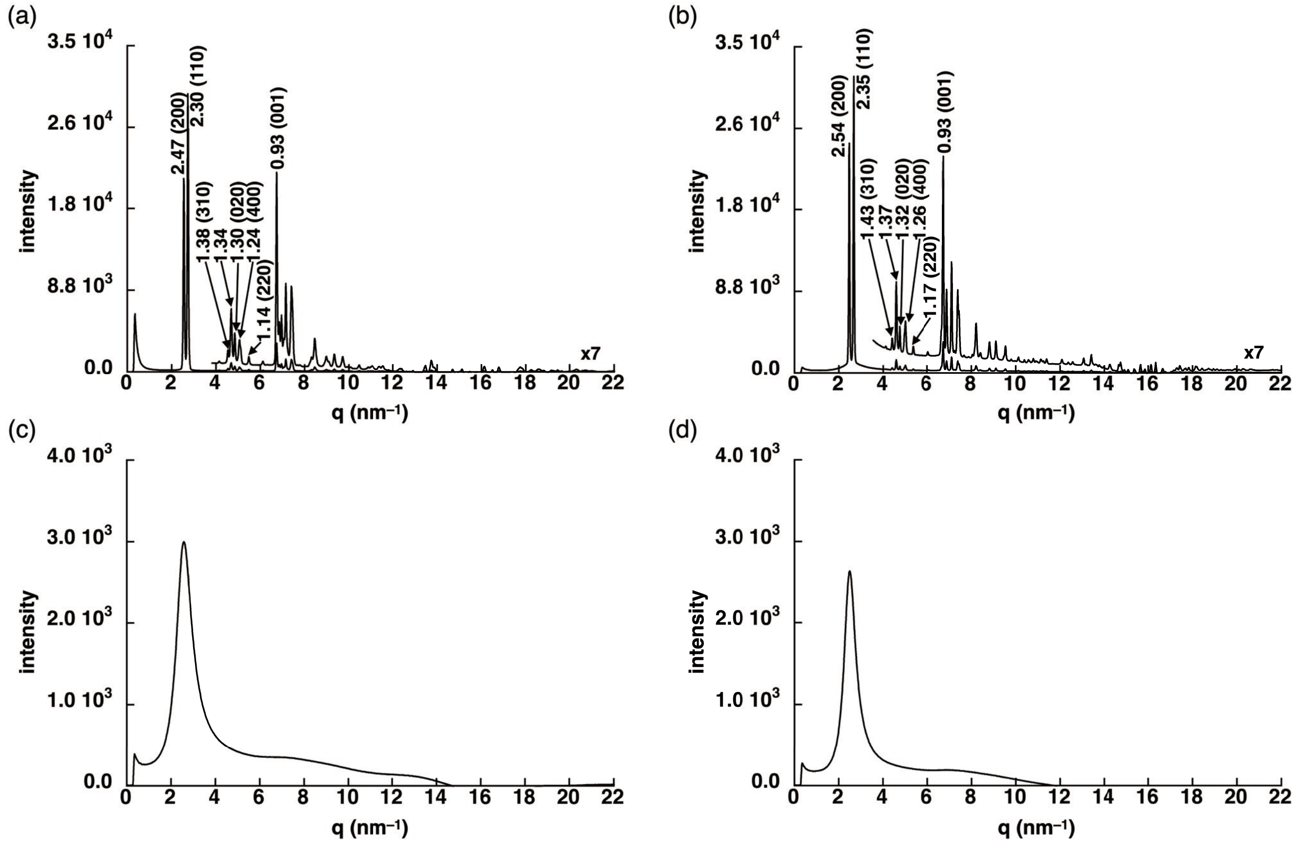

Fig. S54 XRD patterns of **1c** at (a) 25 °C, (b) 120 °C, (c) 135 °C, and (d) 25 °C upon (a–c) 1st heating and (d) 1st cooling.

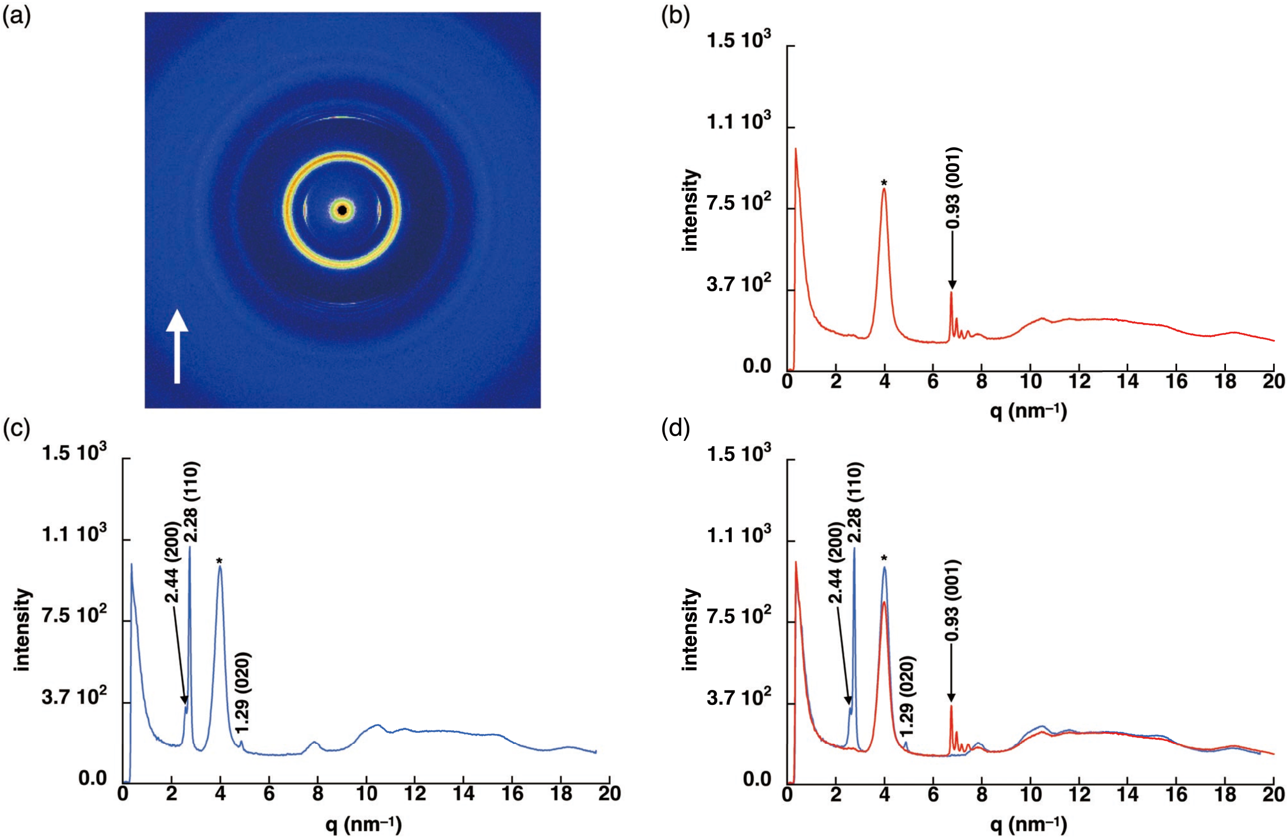

Fig. S55 XRD patterns of **1c** sheared between Kapton (polyimide) films at r.t.: (a) 2D XRD diffraction pattern with an arrow indicating sheared direction, (b) a diagram of meridional (sheared) direction (90° ± 10°), (c) a diagram of equatorial direction (0° ± 10°), and (d) a combined diagram including meridional (sheared) (red) and equatorial (blue) directions. Diffractions of asterisk indicate the diffractions from Kapton film. The diffractions at the smaller angle region assignable to the rectangular packing increased in the equatorial direction (blue line in (c,d)), whereas the diffractions at the wider angle region including the (001) peak (0.93 nm) enhanced in the meridional direction (red in (b,d)). The (001) peak (0.93 nm) is assignable to the repeating distance of trimer of **1c**. These results clearly suggest that the rectangularly assembled **1c** is highly oriented by shearing.

Table S3 Summary of XRD data of **1c**. The peaks which can be indexed are represented.

	q (nm^{-1})	*d*-spacing (nm)	ratio	ratio (calc.)	*hkl*
(a) 25 °C (1st heating)<br>Col$_r$<br>a = 4.94 nm, b = 2.60 nm, c = 0.93 nm [a]<br>Z = 6 (ρ = 1.05)	2.55	2.47	1.00	1.000	200
	2.73	2.30	0.93	0.931	110
	4.54	1.38	0.56	0.563	310
	4.85	1.30	0.52	0.526	020
	5.06	1.24	0.50	0.500	400
	5.49	1.14	0.46	0.466	220
	6.75	0.93	–	–	001
(b) 130 °C (1st cooling)<br>Col$_r$<br>a = 5.09 nm, b = 2.65 nm, c = 0.93 nm [a]<br>Z = 6 (ρ = 1.00)	2.47	2.54	1.00	1.000	200
	2.67	2.35	0.92	0.925	110
	4.40	1.43	0.56	0.562	310
	4.75	1.32	0.52	0.521	020
	5.00	1.26	0.49	0.500	400
	5.36	1.17	0.46	0.462	220
	6.73	0.93	–	–	001

[a] Stacking distance was estimated from the diffraction peak which was enhanced by shearing for **1c** (Fig. S55).

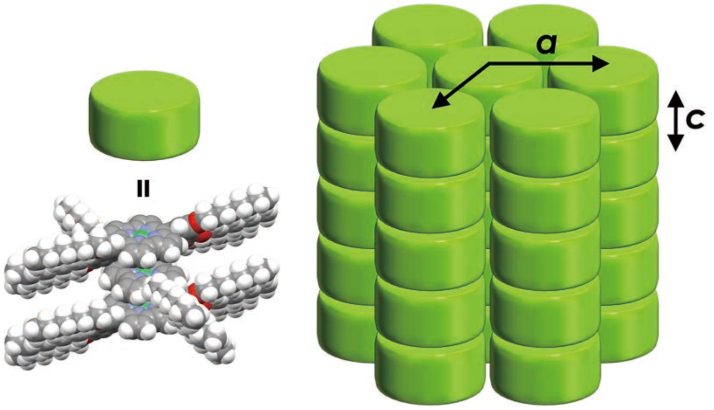

Fig. S56 Possible packing model of **1c** as a Col_r structure.

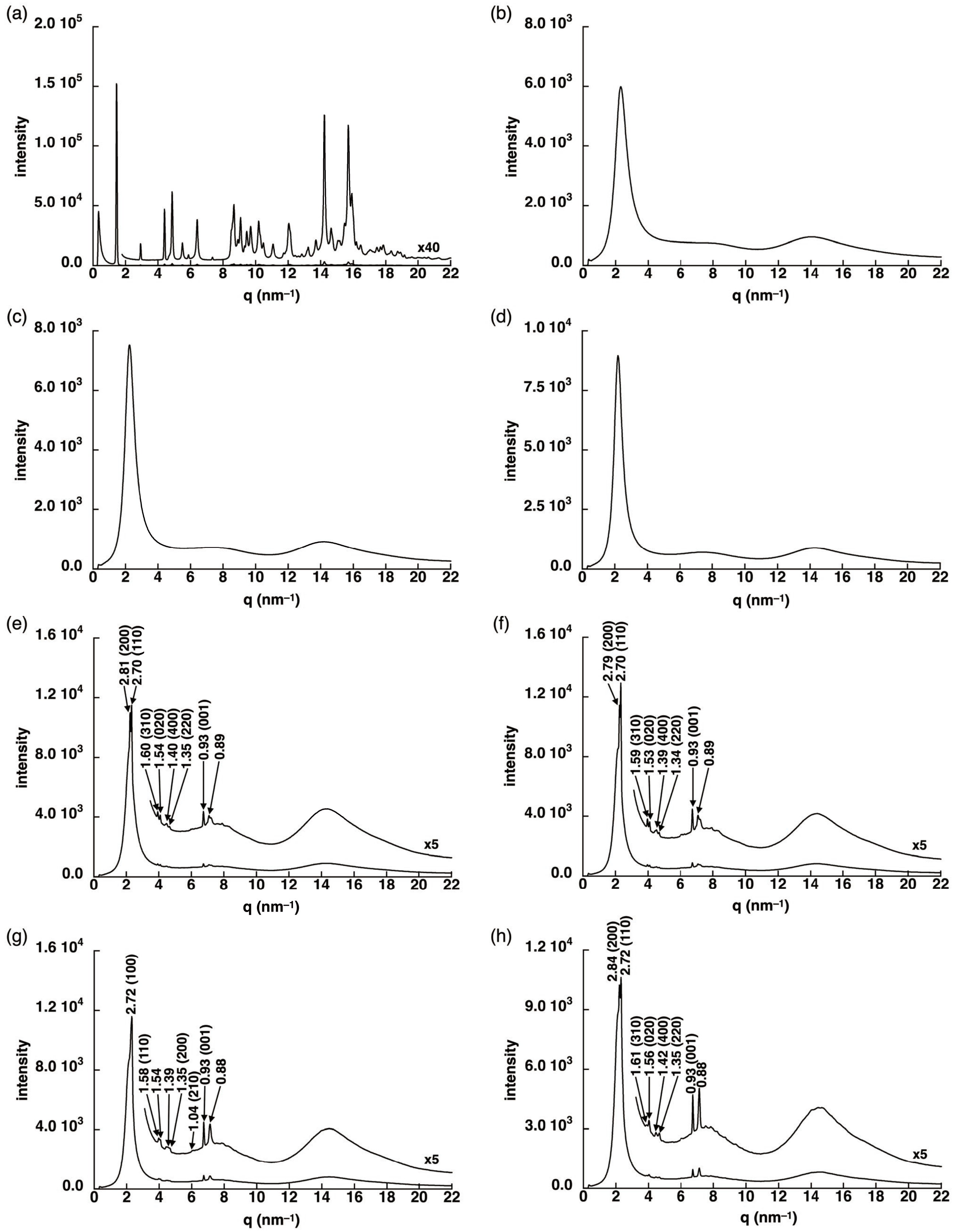

Fig. S57 XRD patterns of **1d** at (a) 25 °C, (b) 85 °C, (c) 60 °C, (d) 40 °C, (e) 40 °C (after maintaining for 15 min), (f) 25 °C, (g) 20 °C, (h) 15 °C, (i) 0 °C, (j) –10 °C, (k) –15 °C, (l) –35 °C, (m) 0 °C, (n) 10 °C, (o) 20 °C, (p) 25 °C, (q) 30 °C, (r) 40 °C, (s) 45 °C, (t) 50 °C, (u) 65 °C, (v) 75 °C, (w) 75 °C (after maintaining for 8 min), (x) 85 °C, (y) 60 °C, (z) 40 °C, (aa) 40 °C (after maintaining for 15 min), (ab) 25 °C, (ac) 20 °C, (ad) 15 °C, (ae) 0 °C, (af) –10 °C, (ag) –15 °C, and (ah) –35 °C upon (a,b) 1st heating, (c–l) 1st cooling, (m–x) 2nd heating, and (y–ah) 2nd cooling. The diffraction at 0.88–0.89 nm may be derived from the side aryl moieties as observed in the enhanced XRD peak upon shearing (Fig. S58).

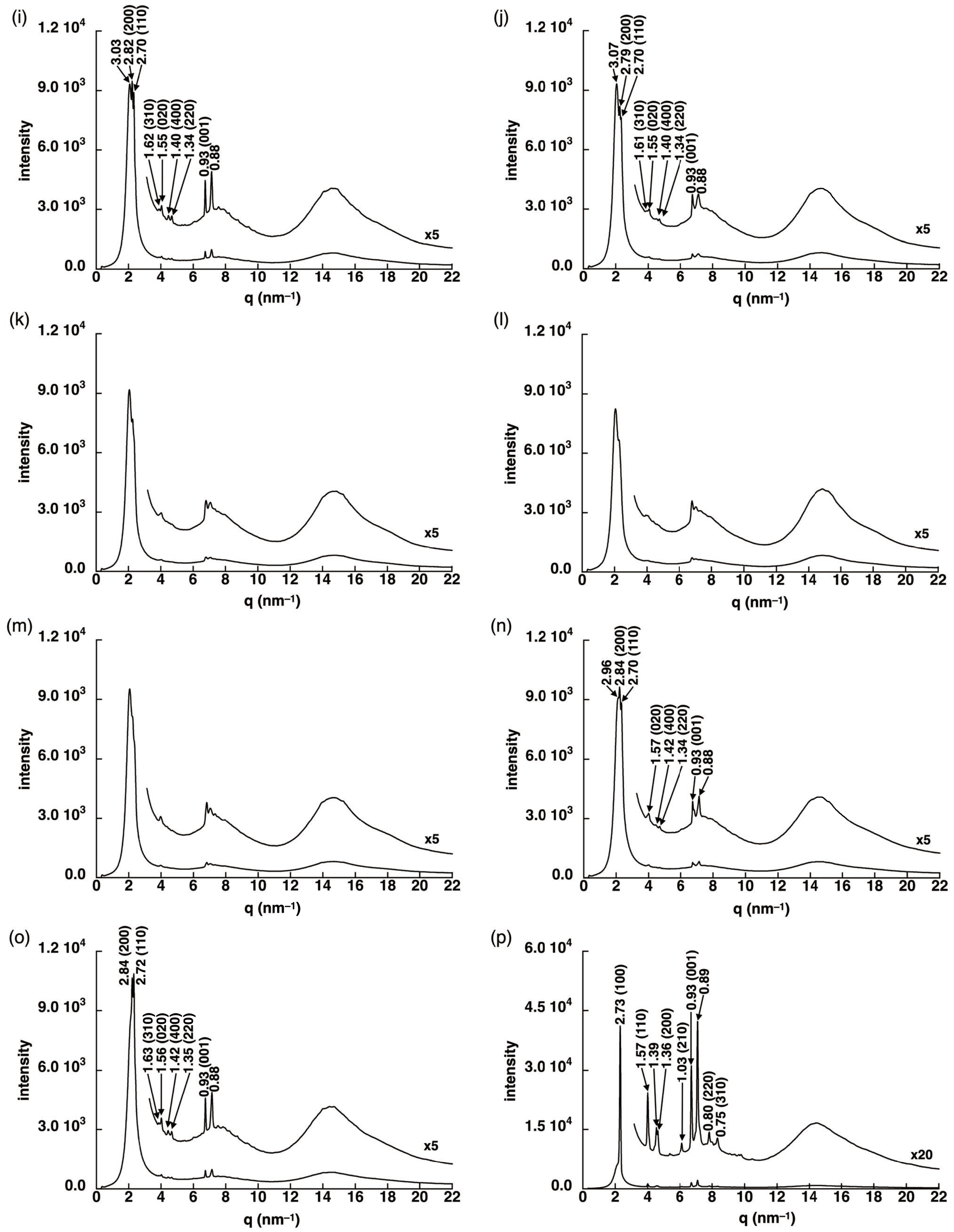

Fig. S57 (Continued)

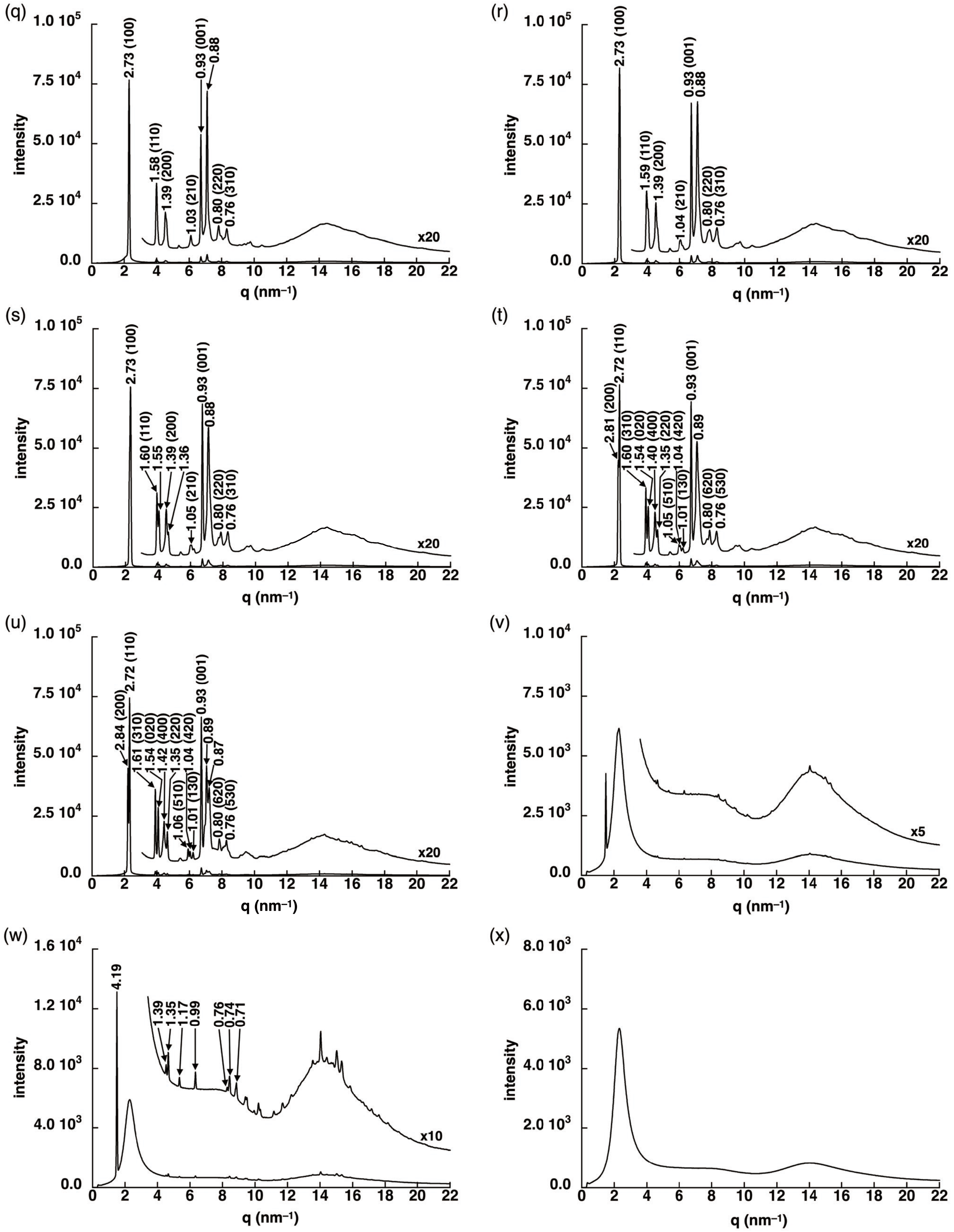

Fig. S57 (Continued)

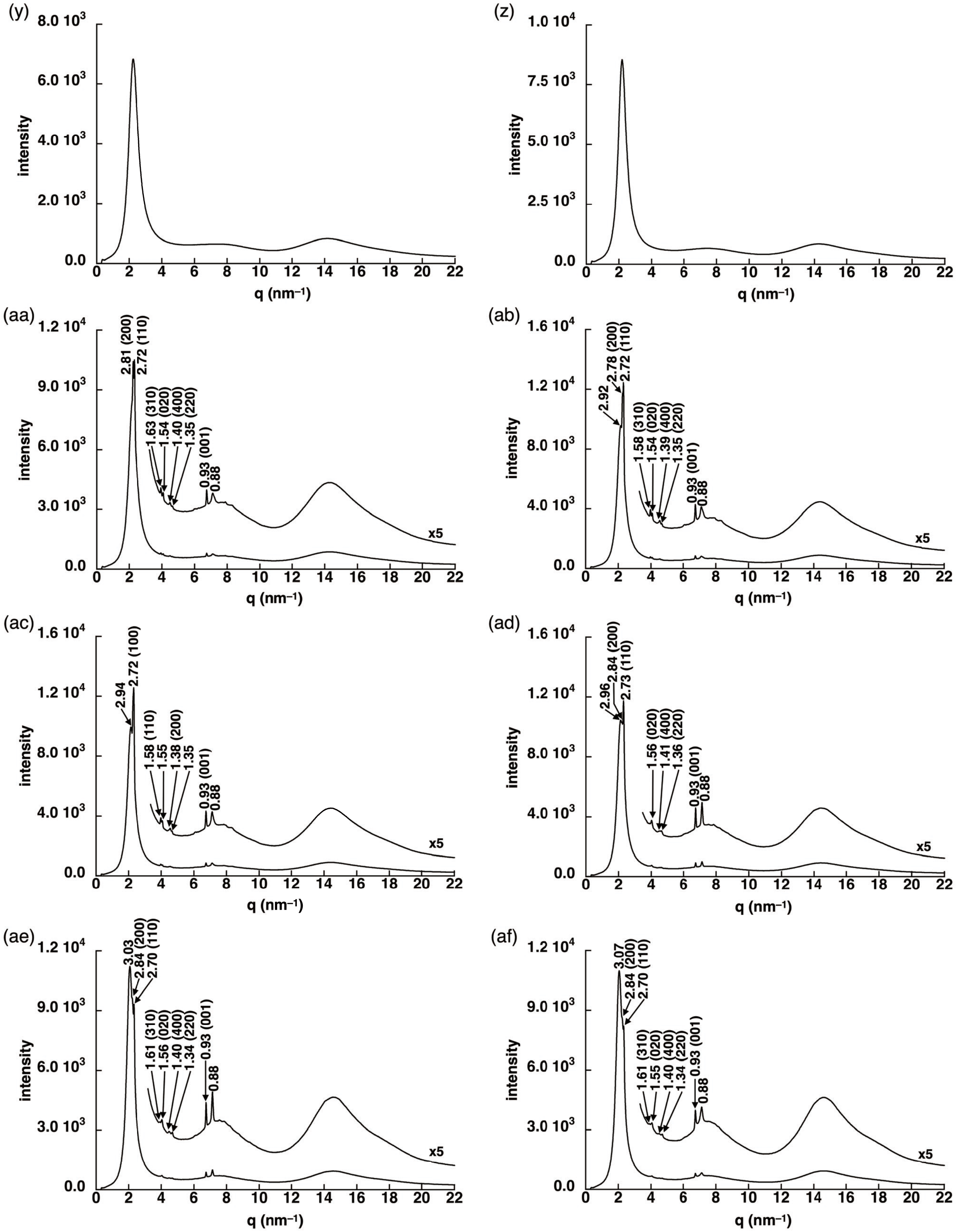

Fig. S57 (Continued)

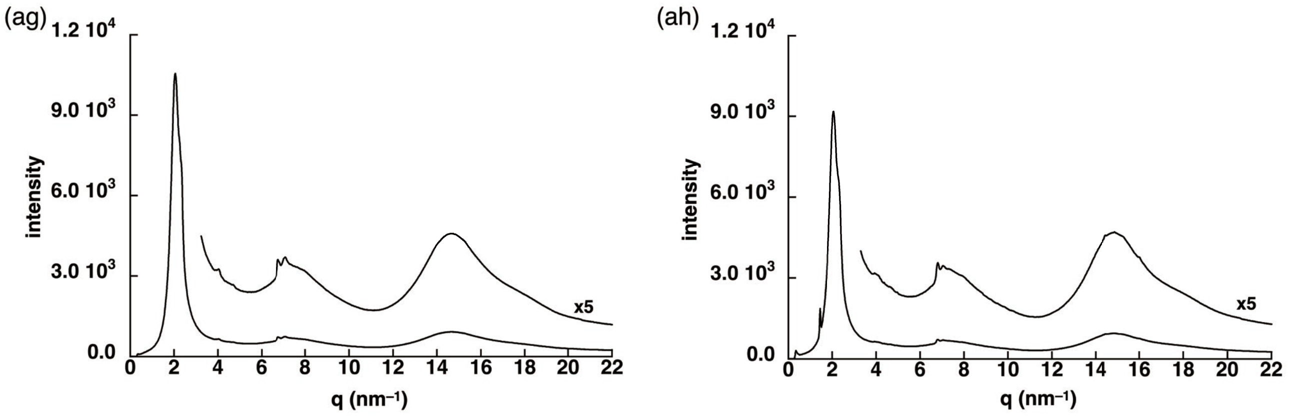

Fig. S57 (Continued)

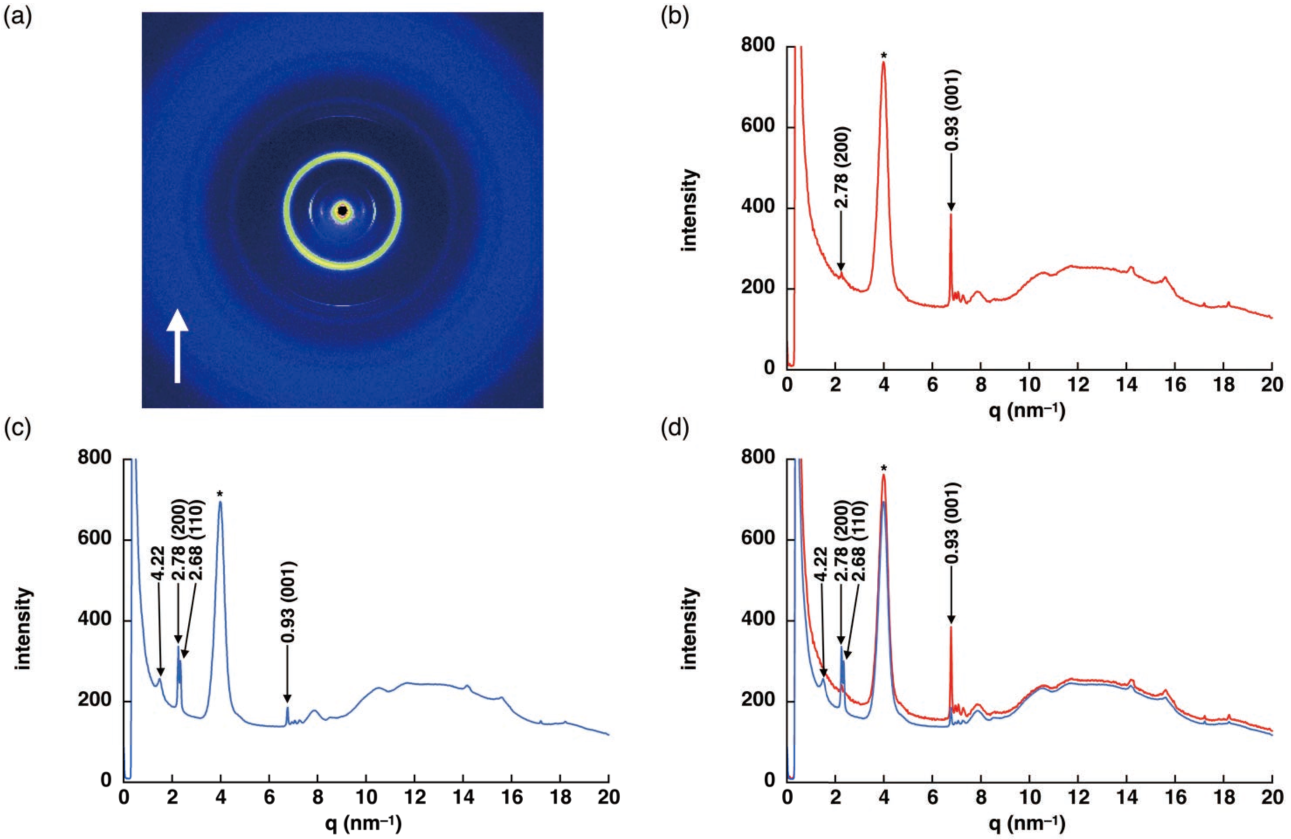

Fig. S58 XRD patterns of **1d** sheared between Kapton (polyimide) films at ca. 85 °C and cooled to r.t.: (a) 2D XRD diffraction pattern with an arrow indicating sheared direction, (b) a diagram of meridional (sheared) direction (90° ± 10°), (c) a diagram of equatorial direction (0° ± 10°), and (d) a combined diagram including meridional (sheared) (red) and equatorial (blue) directions. Diffractions of asterisk indicate the diffractions from Kapton film. The diffractions at the smaller angle region assignable to the rectangular packing increased in the equatorial direction (blue line in (c,d)), whereas the diffractions at the wider angle region including the (001) peak (0.93 nm) enhanced in the meridional direction (red in (b,d)). The (001) peak (0.93 nm) is assignable to the repeating distance of the triple decker of **1d**. These results clearly suggest that the rectangularly assembled **1d** is highly oriented by shearing.

Table S4 Summary of XRD data of **1d**. The peaks which can be indexed are represented.

	q (nm^{-1})	*d*-spacing (nm)	ratio	ratio (calc.)	hkl
(e) 40 °C after maintaining for 15 min (1st cooling)<br>Col_r<br>a = 5.62 nm, b = 3.08 nm, c = 0.93 nm[a]<br>Z = 6 (ρ = 0.99)	2.24	2.81	1.00	1.000	200
	2.32	2.70	0.96	0.962	110
	3.94	1.60	0.57	0.570	310
	4.09	1.54	0.55	0.549	020
	4.47	1.40	0.50	0.500	400
	4.66	1.35	0.48	0.481	220
	6.75	0.93	–	–	001
(f) 25 °C (1st cooling)<br>Col_r<br>a = 5.59 nm, b = 3.09 nm, c = 0.93 nm[a]<br>Z = 6 (ρ = 0.99)	2.25	2.79	1.00	1.000	200
	2.32	2.70	0.97	0.968	110
	3.96	1.59	0.57	0.571	310
	4.10	1.53	0.55	0.553	020
	4.51	1.39	0.50	0.500	400
	4.67	1.34	0.48	0.484	220
	6.75	0.93	–	–	001
(g) 20 °C (1st cooling)<br>Col_h<br>a = 3.14 nm, c = 0.93 nm[a]<br>Z = 3 (ρ = 1.00)	2.31	2.72	1.00	1.000	100
	3.97	1.58	0.58	0.577	110
	4.65	1.35	0.50	0.500	200
	6.05	1.04	0.38	0.378	210
	6.75	0.93	–	–	001
(h) 15 °C (1st cooling)<br>Col_r<br>a = 5.68 nm, b = 3.09 nm, c = 0.93 nm[a]<br>Z = 6 (ρ = 0.97)	2.21	2.84	1.00	1.000	200
	2.31	2.72	0.96	0.957	110
	3.91	1.61	0.57	0.569	310
	4.02	1.56	0.55	0.545	020
	4.44	1.42	0.50	0.500	400
	4.66	1.35	0.47	0.478	220
	6.75	0.93	–	–	001
(i) 0 °C (1st cooling)<br>Col_r<br>a = 5.65 nm, b = 3.08 nm, c = 0.93 nm[a]<br>Z = 6 (ρ = 0.99)	2.22	2.82	1.00	1.000	200
	2.32	2.70	0.96	0.957	110
	3.89	1.62	0.57	0.569	310
	4.05	1.55	0.55	0.545	020
	4.47	1.40	0.50	0.500	400
	4.69	1.34	0.47	0.478	220
	6.75	0.93	–	–	001
(j) –10 °C (1st cooling)<br>Col_r<br>a = 5.59 nm, b = 3.09 nm, c = 0.93 nm[a]<br>Z = 6 (ρ = 0.99)	2.25	2.79	1.00	1.000	200
	2.32	2.70	0.97	0.968	110
	3.90	1.61	0.58	0.571	310
	4.06	1.55	0.55	0.553	020
	4.49	1.40	0.50	0.500	400
	4.69	1.34	0.48	0.484	220
	6.76	0.93	–	–	001
(n) 10 °C (2nd heating)<br>Col_r<br>a = 5.68 nm, b = 3.07 nm, c = 0.93 nm[a]<br>Z = 6 (ρ = 0.98)	2.21	2.84	1.00	1.000	200
	2.32	2.70	0.95	0.952	110
	4.00	1.57	0.55	0.541	020
	4.44	1.42	0.50	0.500	400
	4.67	1.34	0.47	0.476	220
	6.75	0.93	–	–	001
(o) 20 °C (2nd heating)<br>Col_r<br>a = 5.68 nm, b = 3.09 nm, c = 0.93 nm[a]<br>Z = 6 (ρ = 0.97)	2.21	2.84	1.00	1.000	200
	2.31	2.72	0.96	0.957	110
	3.85	1.63	0.57	0.569	310
	4.02	1.56	0.55	0.545	020
	4.44	1.42	0.50	0.500	400
	4.66	1.35	0.47	0.478	220
	6.75	0.93	–	–	001

Table S4 (Continued)

	q (nm^{-1})	*d*-spacing (nm)	ratio	ratio (calc.)	hkl
(p) 25 °C (2nd heating)<br>Col_h<br>a = 3.15 nm, c = 0.93 nm[a]<br>Z = 3 (ρ = 0.99)	2.30	2.73	1.00	1.000	100
	4.00	1.57	0.57	0.577	110
	4.62	1.36	0.50	0.500	200
	6.13	1.03	0.38	0.378	210
	6.72	0.93	–	–	001
	7.81	0.80	0.29	0.289	220
	8.33	0.75	0.28	0.277	310
(q) 30 °C (2nd heating)<br>Col_h<br>a = 3.15 nm, c = 0.93 nm[a]<br>Z = 3 (ρ = 0.99)	2.30	2.73	1.00	1.000	100
	3.99	1.58	0.58	0.577	110
	4.54	1.39	0.51	0.500	200
	6.10	1.03	0.38	0.378	210
	6.72	0.93	0.34	–	001
	7.81	0.80	0.29	0.289	220
	8.31	0.76	0.28	0.277	310
(r) 40 °C (2nd heating)<br>Col_h<br>a = 3.15 nm, c = 0.93 nm[a]<br>Z = 3 (ρ = 0.99)	2.30	2.73	1.00	1.000	100
	3.96	1.59	0.58	0.577	110
	4.52	1.39	0.51	0.500	200
	6.07	1.04	0.38	0.378	210
	6.72	0.93	–	–	001
	7.85	0.80	0.29	0.289	220
	8.30	0.76	0.28	0.277	310
(s) 45 °C (2nd heating)<br>Col_h<br>a = 3.15 nm, c = 0.93 nm[a]<br>Z = 3 (ρ = 0.99)	2.30	2.73	1.00	1.000	100
	3.94	1.60	0.58	0.577	110
	4.64	1.36	0.50	0.500	200
	6.00	1.05	0.38	0.378	210
	6.74	0.93	–	–	001
	7.87	0.80	0.29	0.289	220
	8.30	0.76	0.28	0.277	310
(t) 50 °C (2nd heating)<br>Col_r<br>a = 5.62 nm, b = 3.10 nm, c = 0.93 nm[a]<br>Z = 6 (ρ = 0.98)	2.24	2.81	1.00	1.000	200
	2.31	2.72	0.97	0.967	110
	3.93	1.60	0.57	0.571	310
	4.07	1.54	0.55	0.553	020
	4.49	1.40	0.50	0.500	400
	4.65	1.35	0.48	0.484	220
	5.97	1.05	0.37	0.376	510
	6.05	1.04	0.37	0.371	420
	6.21	1.01	0.36	0.362	130
	6.74	0.93	–	–	001
	7.87	0.80	0.28	0.285	620
	8.29	0.76	0.27	0.271	530
(u) 65 °C (2nd heating)<br>Col_r<br>a = 5.68 nm, b = 3.09 nm, c = 0.93 nm[a]<br>Z = 6 (ρ = 0.97)	2.21	2.84	1.00	1.000	200
	2.31	2.72	0.96	0.957	110
	3.90	1.61	0.57	0.569	310
	4.07	1.54	0.54	0.545	020
	4.44	1.42	0.50	0.500	400
	4.65	1.35	0.48	0.478	220
	5.92	1.06	0.37	0.375	510
	6.04	1.04	0.37	0.368	420
	6.23	1.01	0.36	0.357	130
	6.74	0.93	–	–	001
	7.83	0.80	0.28	0.284	620
	8.28	0.76	0.27	0.269	530

Table S4 (Continued)

	q (nm^{-1})	*d*-spacing (nm)	ratio	ratio (calc.)	hkl
	2.24	2.81	1.00	1.000	200
(aa) 40 °C after maintaining for 15 min (2nd	2.31	2.72	0.97	0.967	110
cooling)	3.94	1.60	0.57	0.571	310
Col_r	4.07	1.54	0.55	0.553	020
a = 5.62 nm, b = 3.10 nm, c = 0.93 nm[a]	4.50	1.40	0.50	0.500	400
Z = 6 (ρ = 0.98)	4.65	1.35	0.48	0.484	220
	6.75	0.93	–	–	001
	2.26	2.78	1.00	1.000	200
(ab) 25 °C (2nd cooling)	2.31	2.72	0.98	0.978	110
Col_r	3.97	1.58	0.57	0.573	310
a = 5.56 nm, b = 3.12 nm, c = 0.93 nm[a]	4.07	1.54	0.56	0.561	020
Z = 6 (ρ = 0.99)	4.54	1.39	0.50	0.500	400
	4.66	1.35	0.49	0.489	220
	6.75	0.93	–	–	001
(ac) 20 °C (2nd cooling)	2.31	2.72	1.00	1.000	100
Col_h	3.97	1.58	0.58	0.577	110
a = 3.14 nm, c = 0.93 nm[a]	4.65	1.35	0.50	0.500	200
Z = 3 (ρ = 1.00)	6.75	0.93	–	–	001
(ad) 15 °C (2nd cooling)	2.21	2.84	1.00	1.000	100
Col_r	2.30	2.73	0.96	0.962	110
a = 5.68 nm, b = 3.12 nm, c = 0.93 nm[a]	4.02	1.56	0.55	0.549	020
Z = 6 (ρ = 0.97)	4.62	1.36	0.48	0.481	200
	6.75	0.93	–	–	001
	2.21	2.84	1.00	1.000	200
(ae) 0 °C (2nd cooling)	2.32	2.70	0.95	0.952	110
Col_r	3.91	1.61	0.57	0.568	310
a = 5.68 nm, b = 3.07 nm, c = 0.93 nm[a]	4.04	1.56	0.55	0.541	020
Z = 6 (ρ = 0.98)	4.47	1.40	0.49	0.500	400
	4.67	1.34	0.47	0.476	220
	6.75	0.93	–	–	001
	2.21	2.84	1.00	1.000	200
(af) –10 °C (2nd cooling)	2.32	2.70	0.95	0.952	110
Col_r	3.91	1.61	0.57	0.568	310
a = 5.68 nm, b = 3.07 nm, c = 0.93 nm[a]	4.05	1.55	0.55	0.541	020
Z = 6 (ρ = 0.98)	4.49	1.40	0.49	0.500	400
	4.69	1.34	0.47	0.476	220
	6.75	0.93	–	–	001

[a] Stacking distance was estimated from the diffraction peak which was enhanced by shearing for **1d** (Fig. S58).

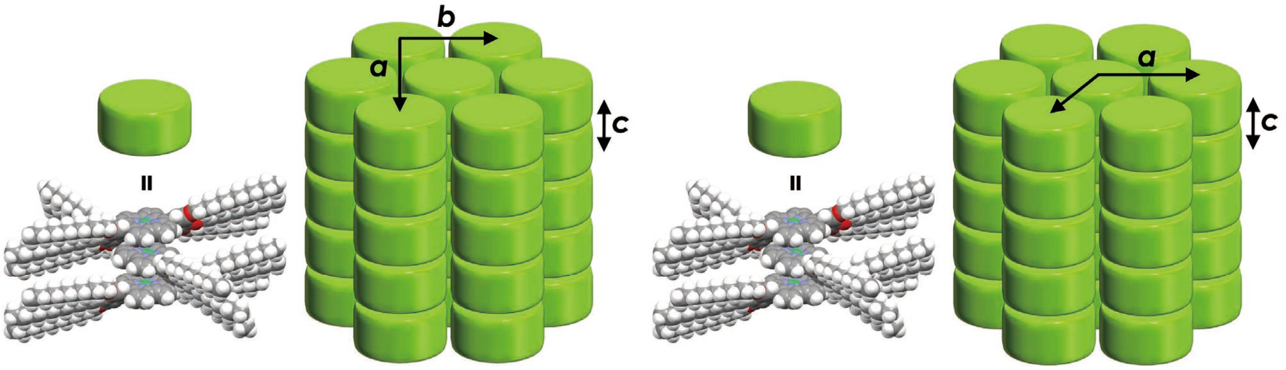

Fig. S59 Possible packing models of **1d** as Col_r and Col_h structures.

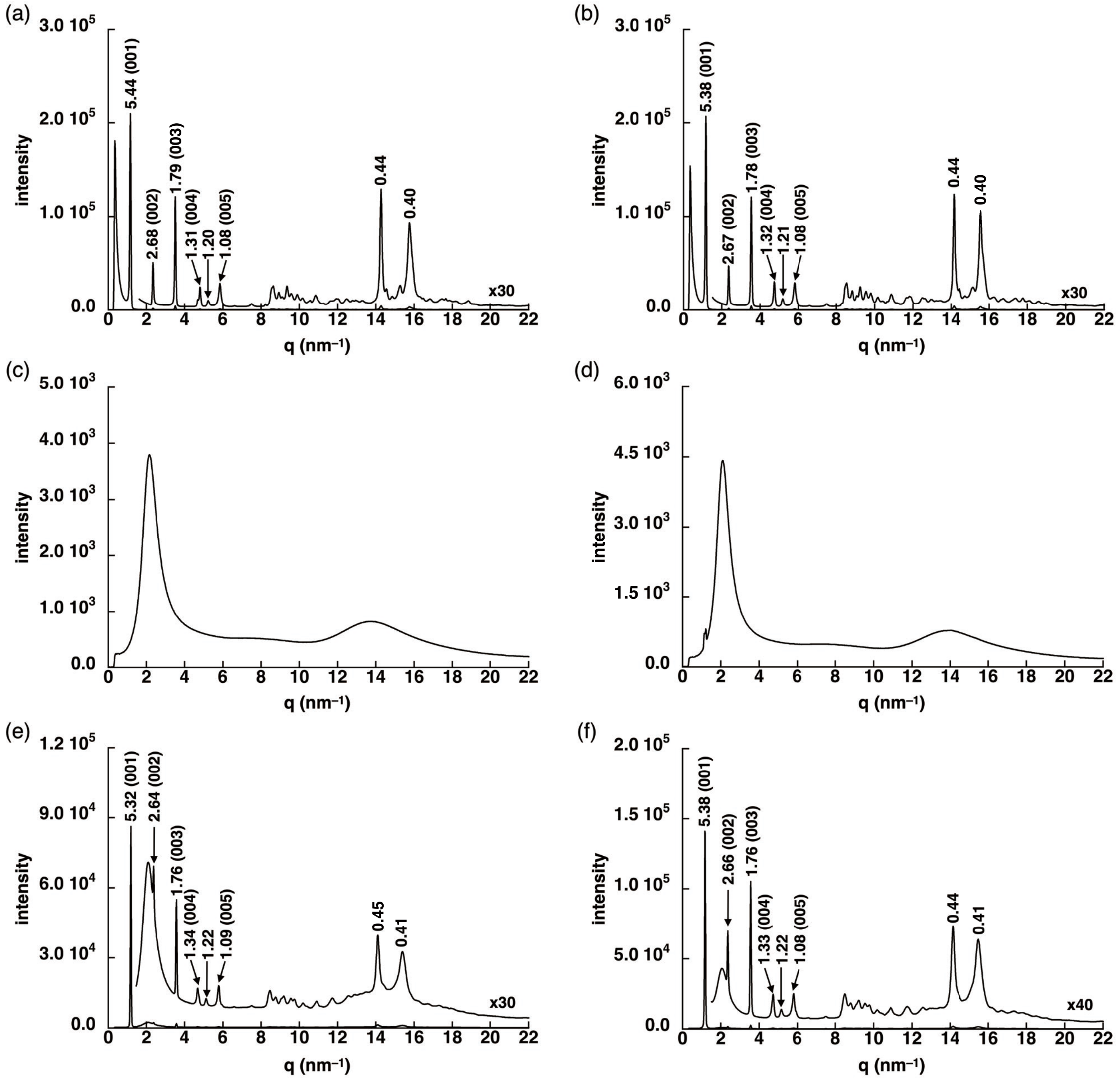

Fig. S60 XRD patterns of **1e** at (a) 25 °C, (b) 70 °C, (c) 104 °C, (d) 80 °C, (e) 80 °C (after maintaining for 15 min), (f) 70 °C (after maintaining for 15 min), (g) 60 °C (after maintaining for 15 min), (h) 45 °C, (i) 36 °C, (j) 0 °C, (k) –20 °C, (l) 35 °C, (m) 55 °C, (n) 60 °C, (o) 80 °C, (p) 104 °C, (q) 80 °C, (r) 80 °C (after maintaining for 15 min), (s) 70 °C (after maintaining for 15 min), (t) 60 °C (after maintaining for 15 min), (u) 45 °C, (v) 36 °C, (w) 0 °C, and (x) –20 °C upon (a–c) 1st heating, (d–k) 1st cooling, (l–p) 2nd heating, and (q–x) 2nd cooling. A peak at 3.08 nm in (m) can also be ascribed to diffraction derived from (110) face of a Col_h phase (a = 6.21 nm, c = 0.40 nm, Z = 4 (ρ = 0.96)). However, other unassigned peaks were observed at other temperatures. In addition, intensities of the peaks assigned to (100), (200), (300), (400), and (500) in (m) were similar to those of corresponding peaks in lamellar phases (e.g., (l) and (n)). Therefore, the phase was assigned to a lamellar phase.

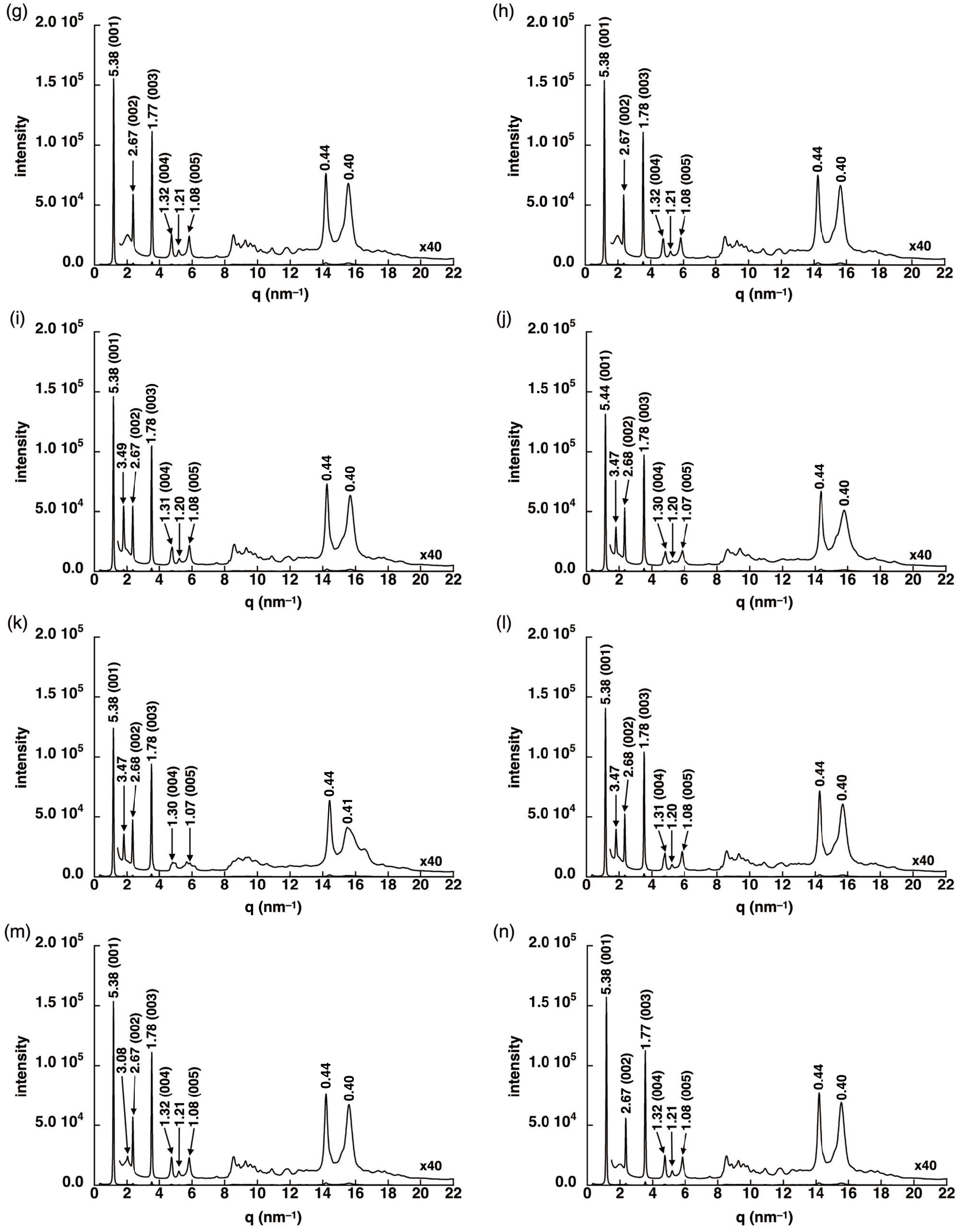

Fig. S60 (Continued)

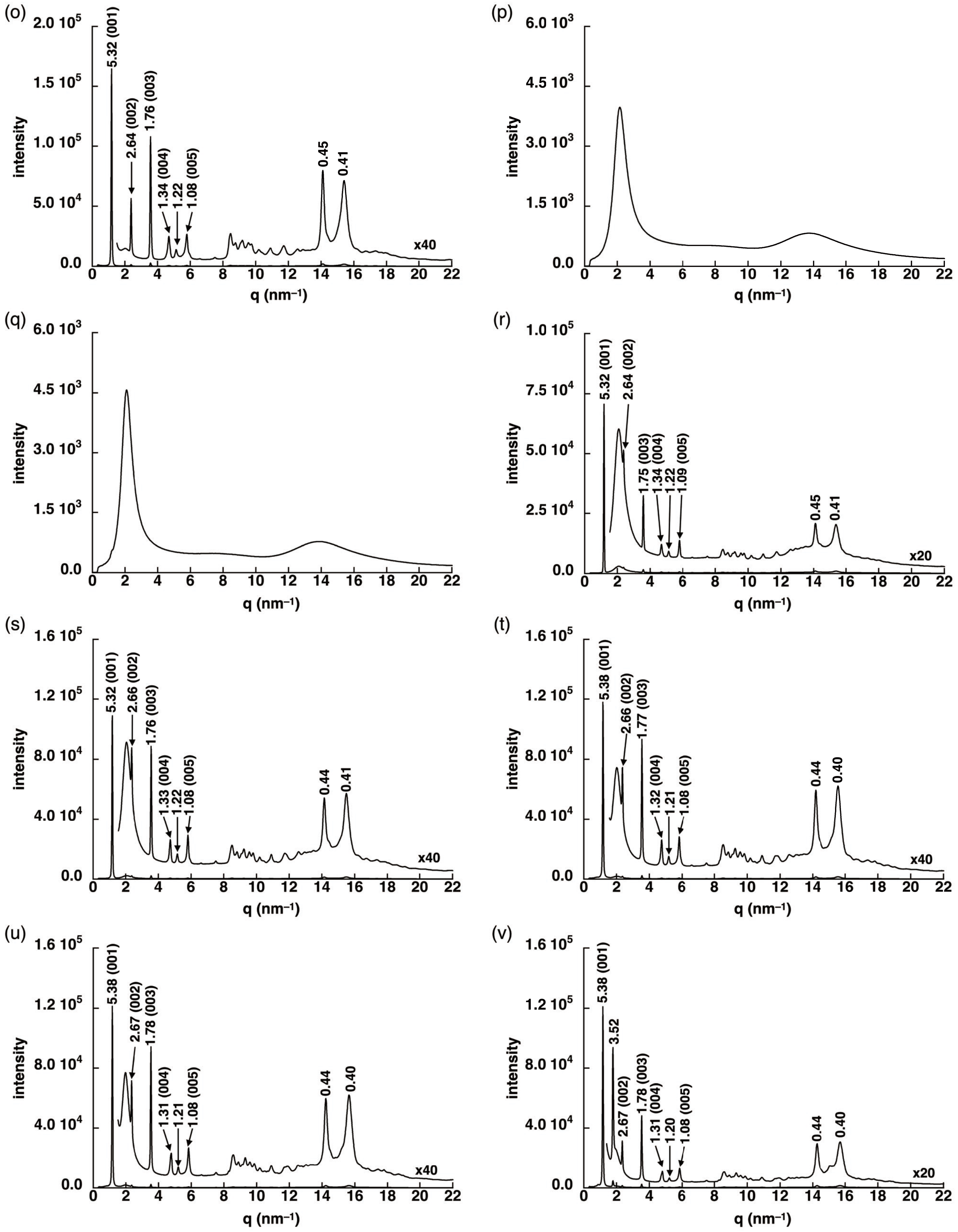

Fig. S60 (Continued)

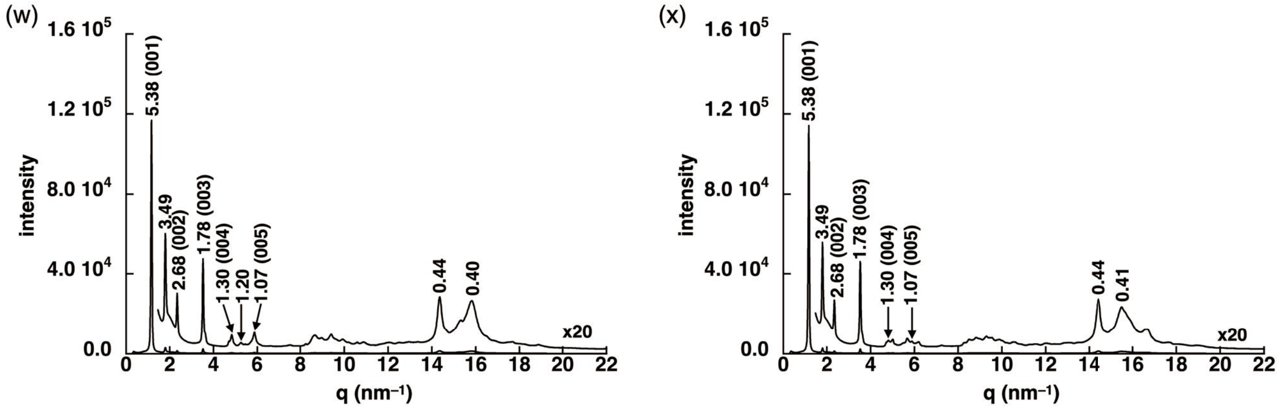

Fig. S60 (Continued)

Table S5 Summary of XRD data of **1e**. The peaks which can be indexed are represented.

	q (nm^{-1})	*d*-spacing (nm)	ratio	ratio (calc.)	*hkl*
(a) 25 °C (1st heating) lamellar	1.15	5.44	1.00	1.000	001
	2.34	2.68	0.49	0.500	002
	3.51	1.79	0.33	0.333	003
	4.80	1.31	0.24	0.250	004
	5.84	1.08	0.20	0.200	005
(b) 70 °C (1st heating) lamellar	1.17	5.38	1.00	1.000	001
	2.35	2.67	0.50	0.500	002
	3.54	1.78	0.33	0.333	003
	4.74	1.32	0.25	0.250	004
	5.81	1.08	0.20	0.200	005
(e) 80 °C after maintaining for 15 min (1st cooling) lamellar	1.18	5.32	1.00	1.000	001
	2.38	2.64	0.50	0.500	002
	3.57	1.76	0.33	0.333	003
	4.69	1.34	0.25	0.250	004
	5.78	1.09	0.20	0.200	005
(f) 70 °C after maintaining for 15 min (1st cooling) lamellar	1.17	5.38	1.00	1.000	001
	2.37	2.66	0.49	0.500	002
	3.56	1.76	0.33	0.333	003
	4.73	1.33	0.25	0.250	004
	5.81	1.08	0.20	0.200	005
(g) 60 °C after maintaining for 15 min (1st cooling) lamellar	1.17	5.38	1.00	1.000	001
	2.35	2.67	0.50	0.500	002
	3.55	1.77	0.33	0.333	003
	4.74	1.32	0.25	0.250	004
	5.82	1.08	0.20	0.200	005
(h) 45 °C (1st cooling) lamellar	1.17	5.38	1.00	1.000	001
	2.35	2.67	0.50	0.500	002
	3.54	1.78	0.33	0.333	003
	4.75	1.32	0.25	0.250	004
	5.83	1.08	0.20	0.200	005
(i) 36 °C (1st cooling) lamellar	1.17	5.38	1.00	1.000	001
	2.35	2.67	0.50	0.500	002
	3.54	1.78	0.33	0.333	003
	4.78	1.31	0.24	0.250	004
	5.84	1.08	0.20	0.200	005
(j) 0 °C (1st cooling) lamellar	1.15	5.44	1.00	1.000	001
	2.34	2.68	0.49	0.500	002
	3.52	1.78	0.33	0.333	003
	4.83	1.30	0.24	0.250	004
	5.87	1.07	0.20	0.200	005
(k) –20 °C (1st cooling) lamellar	1.17	5.38	1.00	1.000	001
	2.34	2.68	0.50	0.500	002
	3.52	1.78	0.33	0.333	003
	4.82	1.30	0.24	0.250	004
	5.88	1.07	0.20	0.200	005
(l) 35 °C (2nd heating) lamellar	1.17	5.38	1.00	1.000	001
	2.34	2.68	0.50	0.500	002
	3.52	1.78	0.33	0.333	003
	4.79	1.31	0.24	0.250	004
	5.84	1.08	0.20	0.200	005

Table S5 (Continued)

	q (nm^{-1})	*d*-spacing (nm)	ratio	ratio (calc.)	*hkl*
(m) 55 °C (2nd heating) lamellar	1.17	5.38	1.00	1.000	100
	2.04	3.08	0.57	0.577	110
	2.35	2.67	0.50	0.500	200
	3.54	1.78	0.33	0.333	300
	4.75	1.32	0.25	0.250	400
	5.83	1.08	0.20	0.200	500
	15.60	0.40	–	–	001
(n) 60 °C (2nd heating) lamellar	1.17	5.38	1.00	1.000	001
	2.35	2.67	0.50	0.500	002
	3.55	1.77	0.33	0.333	003
	4.74	1.32	0.25	0.250	004
	5.82	1.08	0.20	0.200	005
(o) 80 °C (2nd heating) lamellar	1.18	5.32	1.00	1.000	001
	2.38	2.64	0.50	0.500	002
	3.57	1.76	0.33	0.333	003
	4.69	1.34	0.25	0.250	004
	5.79	1.08	0.20	0.200	005
(r) 80 °C after maintaining for 15 min (2nd cooling) lamellar	1.18	5.32	1.00	1.000	001
	2.38	2.64	0.50	0.500	002
	3.59	1.75	0.33	0.333	003
	4.69	1.34	0.25	0.250	004
	5.78	1.09	0.20	0.200	005
(s) 70 °C after maintaining for 15 min (2nd cooling) lamellar	1.18	5.32	1.00	1.000	001
	2.37	2.66	0.50	0.500	002
	3.56	1.76	0.33	0.333	003
	4.73	1.33	0.25	0.250	004
	5.81	1.08	0.20	0.200	005
(t) 60 °C after maintaining for 15 min (2nd cooling) lamellar	1.17	5.38	1.00	1.000	001
	2.37	2.66	0.49	0.500	002
	3.55	1.77	0.33	0.333	003
	4.75	1.32	0.25	0.250	004
	5.82	1.08	0.20	0.200	005
(u) 45 °C (2nd cooling) lamellar	1.17	5.38	1.00	1.000	001
	2.35	2.67	0.50	0.500	002
	3.54	1.78	0.33	0.333	003
	4.78	1.31	0.24	0.250	004
	5.84	1.08	0.20	0.200	005
(v) 36 °C (2nd cooling) lamellar	1.17	5.38	1.00	1.000	001
	2.35	2.67	0.50	0.500	002
	3.54	1.78	0.33	0.333	003
	4.79	1.31	0.24	0.250	004
	5.84	1.08	0.20	0.200	005
(w) 0 °C (2nd cooling) lamellar	1.17	5.38	1.00	1.000	001
	2.34	2.68	0.50	0.500	002
	3.52	1.78	0.33	0.333	003
	4.84	1.30	0.24	0.250	004
	5.87	1.07	0.20	0.200	005
(x) –20 °C (2nd cooling) lamellar	1.17	5.38	1.00	1.000	001
	2.34	2.68	0.50	0.500	002
	3.52	1.78	0.33	0.333	003
	4.82	1.30	0.24	0.250	004
	5.89	1.07	0.20	0.200	005

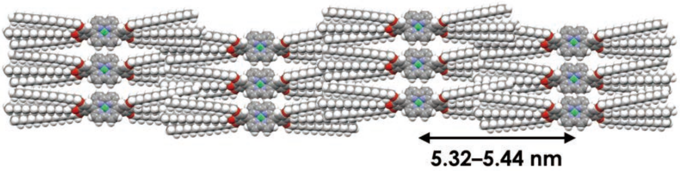

Fig. S61 Possible packing model of **1e** as a lamellar structure.

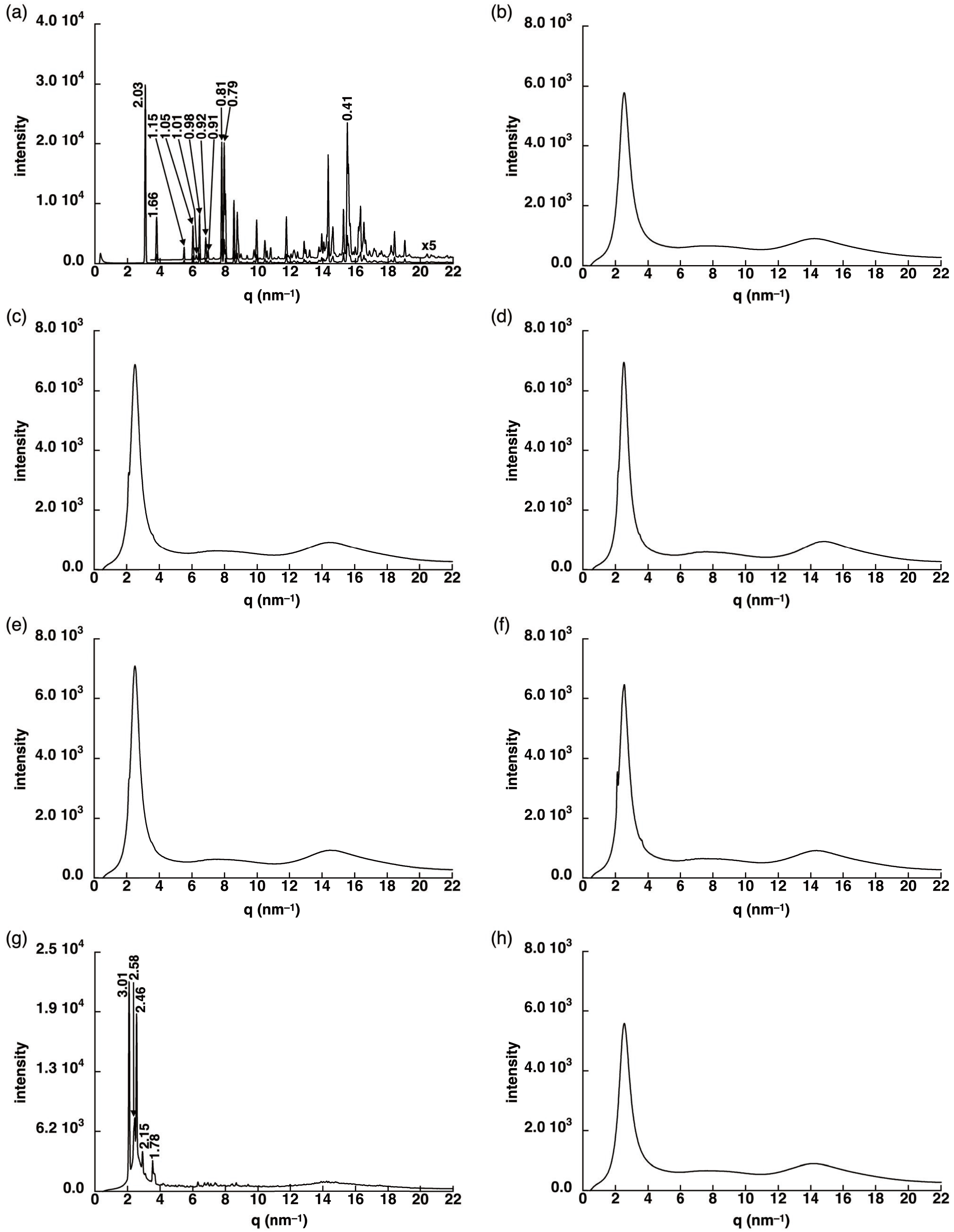

Fig. S62 XRD patterns of **2c** at (a) 25 °C, (b) 75 °C, (c) 30 °C, (d) –30 °C (after maintaining for 10 min), (e) 25 °C, (f) 55 °C, (g) 55 °C (after maintaining for 15 min), (h) 80 °C, (i) 30 °C, and (j) –30 °C upon (a,b) 1st heating, (c,d) 1st cooling, (e–h) 2nd heating, and (i,j) 2nd cooling.

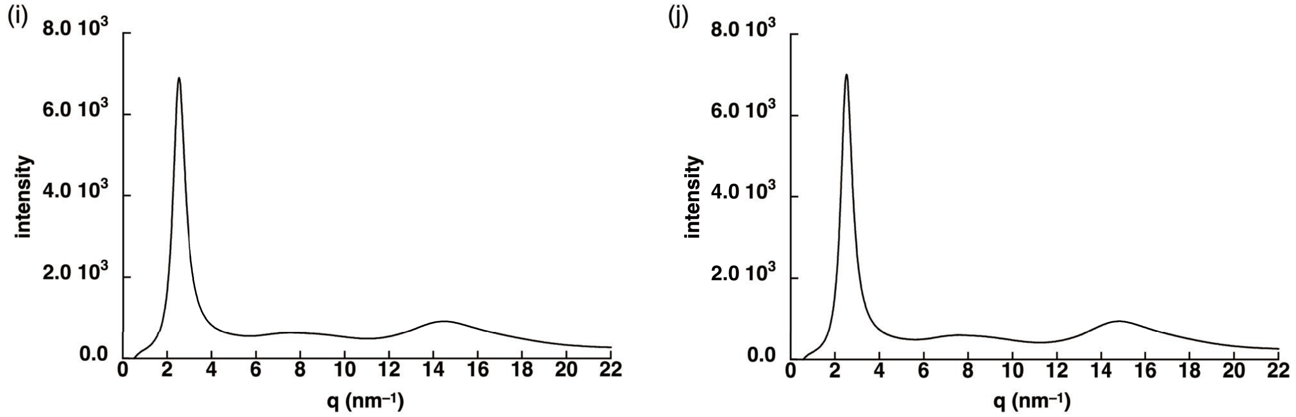

Fig. S62 (Continued)

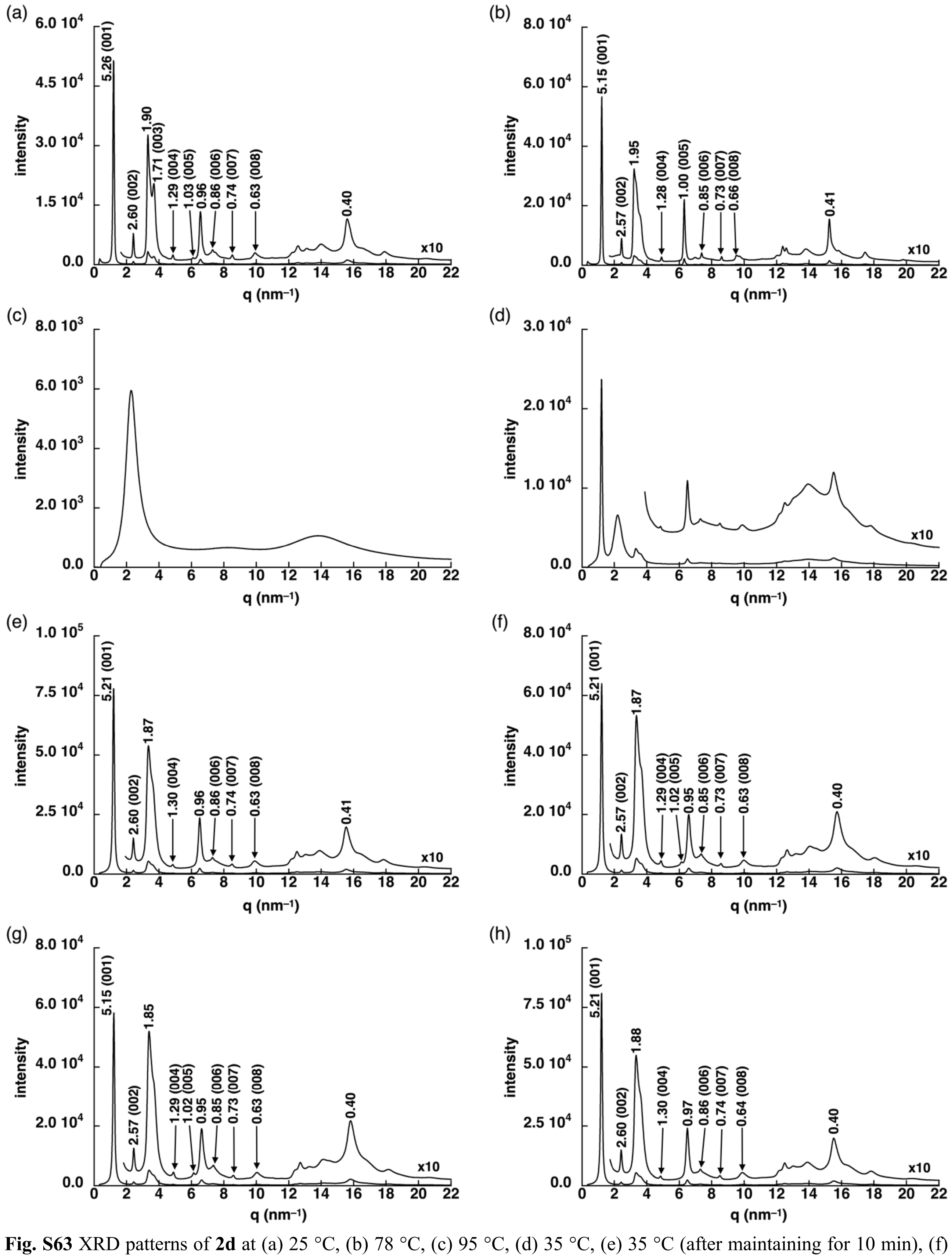

Fig. S63 XRD patterns of **2d** at (a) 25 °C, (b) 78 °C, (c) 95 °C, (d) 35 °C, (e) 35 °C (after maintaining for 10 min), (f) 0 °C, (g) –35 °C, (h) 40 °C, (i) 78 °C, (j) 78 °C (after maintaining for 10 min), (k) 95 °C, (l) 35 °C, (m) 35 °C (after maintaining for 10 min), (n) 0 °C, (o) –35 °C, and (p) 60 °C upon (a–c) 1st heating, (d–g) 1st cooling, (h–k) 2nd heating, (l–o) 2nd cooling, and (p) 3rd cooling.

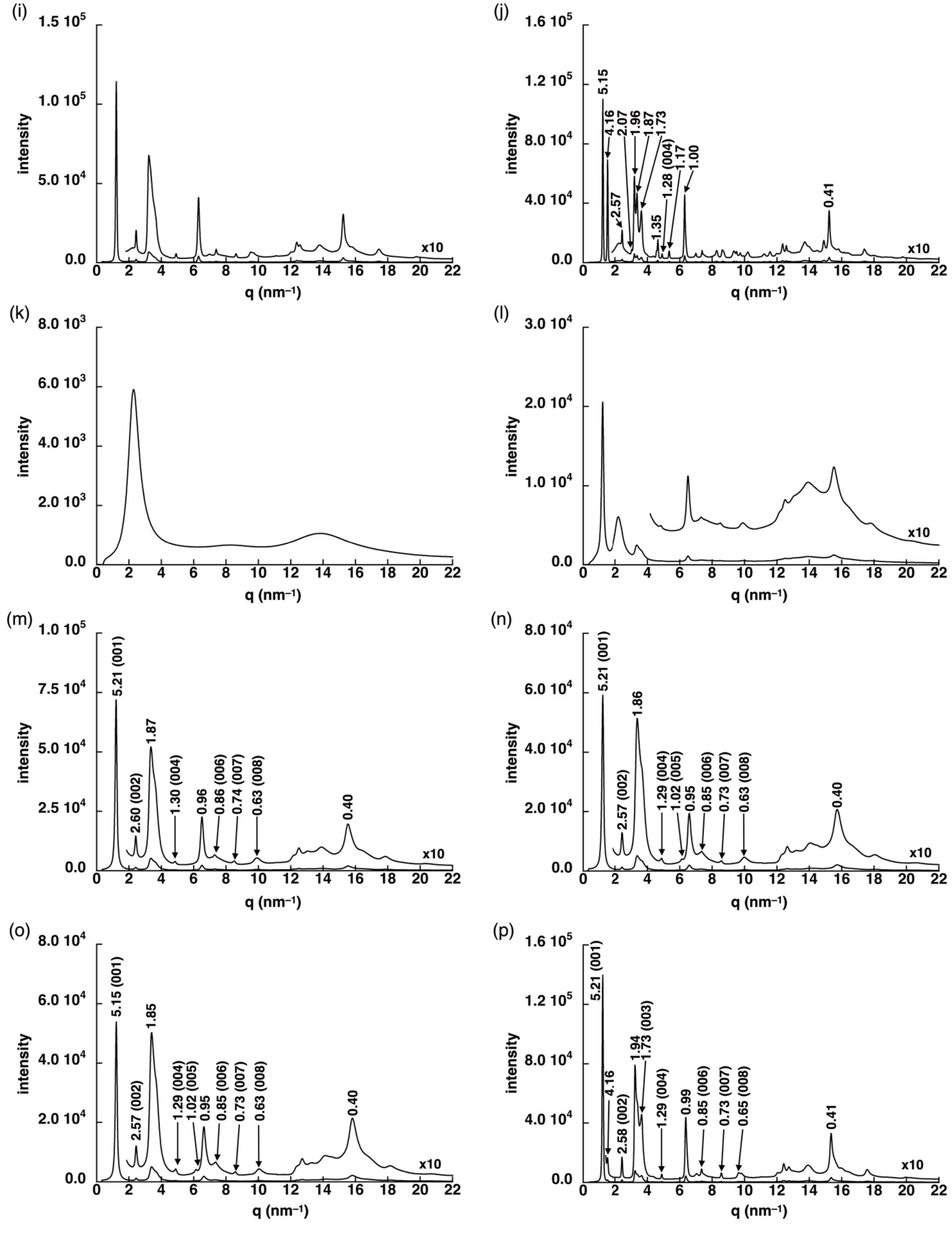

Fig. S63 (Continued)

Table S6 Summary of XRD data of **2d**. The peaks which can be indexed are represented.

	q (nm^{-1})	*d*-spacing (nm)	ratio	ratio (calc.)	*hkl*
(a) 25 °C (1st heating) lamellar	1.19	5.26	1.00	1.000	001
	2.42	2.60	0.49	0.500	002
	3.68	1.71	0.32	0.333	003
	4.86	1.29	0.25	0.250	004
	6.10	1.03	0.20	0.200	005
	7.30	0.86	0.16	0.167	006
	8.52	0.74	0.14	0.143	007
	9.92	0.63	0.12	0.125	008
(b) 78 °C (1st heating) lamellar	1.22	5.15	1.00	1.000	001
	2.44	2.57	0.50	0.500	002
	4.91	1.28	0.25	0.250	004
	6.30	1.00	0.19	0.200	005
	7.39	0.85	0.17	0.167	006
	8.62	0.73	0.14	0.143	007
	9.53	0.66	0.13	0.125	008
(e) 35 °C after maintaining for 10 min (1st cooling) lamellar	1.21	5.21	1.00	1.000	001
	2.42	2.60	0.50	0.500	002
	4.85	1.30	0.25	0.250	004
	6.52	0.96	0.19	0.200	005
	7.30	0.86	0.17	0.167	006
	8.51	0.74	0.14	0.143	007
	9.90	0.63	0.12	0.125	008
(f) 0 °C (1st cooling) lamellar	1.21	5.21	1.00	1.000	001
	2.44	2.57	0.49	0.500	002
	4.89	1.29	0.25	0.250	004
	6.14	1.02	0.20	0.200	005
	7.35	0.85	0.16	0.167	006
	8.57	0.73	0.14	0.143	007
	9.97	0.63	0.12	0.125	008
(g) –35 °C (1st cooling) lamellar	1.22	5.15	1.00	1.000	001
	2.44	2.57	0.50	0.500	002
	4.89	1.29	0.25	0.250	004
	6.14	1.02	0.20	0.200	005
	7.35	0.85	0.17	0.167	006
	8.58	0.73	0.14	0.143	007
	10.04	0.63	0.12	0.125	008
(h) 40 °C (2nd heating) lamellar	1.21	5.21	1.00	1.000	001
	2.42	2.60	0.50	0.500	002
	4.85	1.30	0.25	0.250	004
	6.50	0.97	0.19	0.200	005
	7.30	0.86	0.17	0.167	006
	8.51	0.74	0.14	0.143	007
	9.89	0.64	0.12	0.125	008
(m) 35 °C after maintaining for 10 min (2nd cooling) lamellar	1.21	5.21	1.00	1.000	001
	2.42	2.60	0.50	0.500	002
	4.85	1.30	0.25	0.250	004
	6.52	0.96	0.19	0.200	005
	7.30	0.86	0.17	0.167	006
	8.51	0.74	0.14	0.143	007
	9.90	0.63	0.12	0.125	008

Table S6 (Continued)

	q (nm^{-1})	*d*-spacing (nm)	ratio	ratio (calc.)	*hkl*
(n) 0 °C (2nd cooling) lamellar	1.21	5.21	1.00	1.000	001
	2.44	2.57	0.49	0.500	002
	4.87	1.29	0.25	0.250	004
	6.14	1.02	0.20	0.200	005
	7.35	0.85	0.16	0.167	006
	8.57	0.73	0.14	0.143	007
	9.97	0.63	0.12	0.125	008
(o) –35 °C (2nd cooling) lamellar	1.22	5.15	1.00	1.000	001
	2.44	2.57	0.50	0.500	002
	4.89	1.29	0.25	0.250	004
	6.15	1.02	0.20	0.200	005
	7.35	0.85	0.17	0.167	006
	8.58	0.73	0.14	0.143	007
	10.04	0.63	0.12	0.125	008
(p) 60 °C after maintaining for 10 min (3rd cooling)[a] lamellar	1.21	5.21	1.00	1.000	001
	2.43	2.58	0.50	0.500	002
	3.64	1.73	0.33	0.333	003
	4.89	1.29	0.25	0.250	004
	7.35	0.85	0.16	0.167	006
	8.57	0.73	0.14	0.143	007
	9.64	0.65	0.13	0.125	008

[a] Diffraction at the temperature where a texture different from that formed at 35 °C begins to appear in POM (Fig. S51b). The diffraction pattern was similar to those annealed at 35 °C.

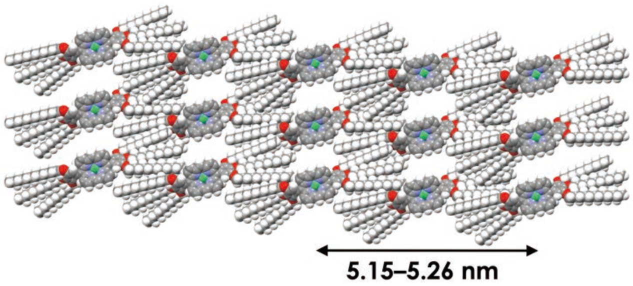

Fig. S64 Possible packing model of **2d** as a lamellar structure.

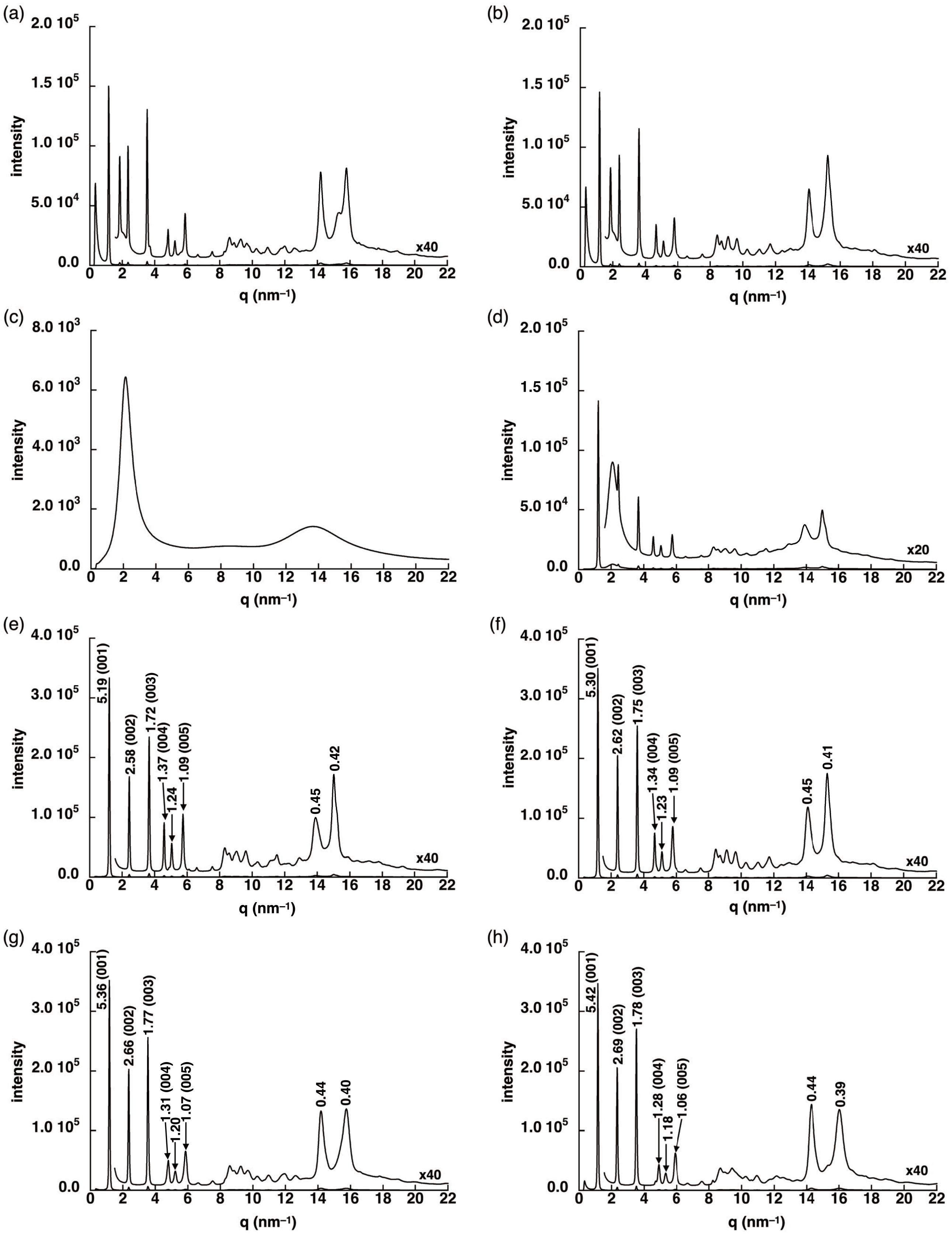

Fig. S65 XRD patterns of **2e** at (a) 25 °C, (b) 55 °C, (c) 105 °C, (d) 80 °C, (e) 80 °C (after maintaining for 10 min), (f) 40 °C, (g) 20 °C, (h) –20 °C, (i) 55 °C, (j) 105 °C, (k) 80 °C, (l) 80 °C (after maintaining for 10 min), (m) 40 °C, (n) 20 °C, and (o) –20 °C upon (a–c) 1st heating, (d–h) 1st cooling, (i,j) 2nd heating, and (k–o) 2nd cooling.

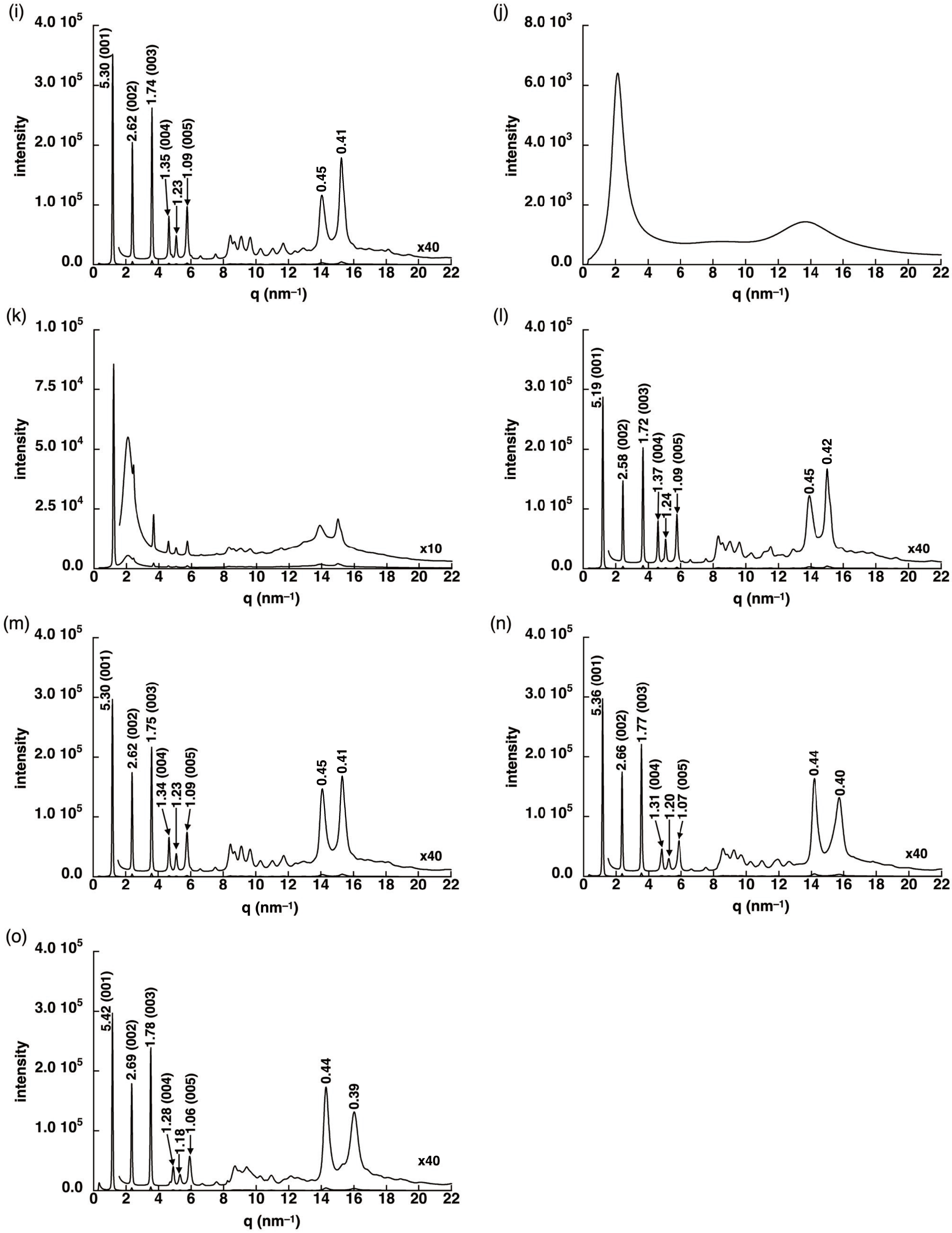

Fig. S65 (Continued)

Table S7 Summary of XRD data of **2e**. The peaks which can be indexed are represented.

	q (nm^{-1})	*d*-spacing (nm)	ratio	ratio (calc.)	*hkl*
(e) 80 °C after maintaining for 10 min (1st heating) lamellar	1.21	5.19	1.00	1.000	001
	2.44	2.58	0.50	0.500	002
	3.66	1.72	0.33	0.333	003
	4.59	1.37	0.26	0.250	004
	5.74	1.09	0.21	0.200	005
(f) 40 °C (1st heating) lamellar	1.18	5.30	1.00	1.000	001
	2.40	2.62	0.49	0.500	002
	3.60	1.75	0.33	0.333	003
	4.67	1.34	0.25	0.250	004
	5.78	1.09	0.20	0.200	005
(g) 20 °C (1st cooling) lamellar	1.17	5.36	1.00	1.000	001
	2.36	2.66	0.50	0.500	002
	3.55	1.77	0.33	0.333	003
	4.80	1.31	0.24	0.250	004
	5.87	1.07	0.20	0.200	005
(h) –20 °C (1st cooling) lamellar	1.16	5.42	1.00	1.000	001
	2.34	2.69	0.50	0.500	002
	3.53	1.78	0.33	0.333	003
	4.90	1.28	0.24	0.250	004
	5.90	1.06	0.20	0.200	005
(i) 55 °C (2nd heating) lamellar	1.18	5.30	1.00	1.000	001
	2.40	2.62	0.49	0.500	002
	3.61	1.74	0.33	0.333	003
	4.65	1.35	0.25	0.250	004
	5.77	1.09	0.21	0.200	005
(l) 80 °C after maintaining for 10 min (2nd cooling) lamellar	1.21	5.19	1.00	1.000	001
	2.44	2.58	0.50	0.500	002
	3.66	1.72	0.33	0.333	003
	4.59	1.37	0.26	0.250	004
	5.74	1.09	0.21	0.200	005
(m) 40 °C (2nd cooling) lamellar	1.18	5.30	1.00	1.000	001
	2.40	2.62	0.49	0.500	002
	3.60	1.75	0.33	0.333	003
	4.67	1.34	0.25	0.250	004
	5.77	1.09	0.21	0.200	005
(n) 20 °C (2nd cooling) lamellar	1.17	5.36	1.00	1.000	001
	2.36	2.66	0.50	0.500	002
	3.55	1.77	0.33	0.333	003
	4.80	1.31	0.24	0.250	004
	5.86	1.07	0.20	0.200	005
(o) –20 °C (2nd cooling) lamellar	1.16	5.42	1.00	1.000	001
	2.34	2.69	0.50	0.500	002
	3.53	1.78	0.33	0.333	003
	4.90	1.28	0.24	0.250	004
	5.92	1.06	0.20	0.200	005

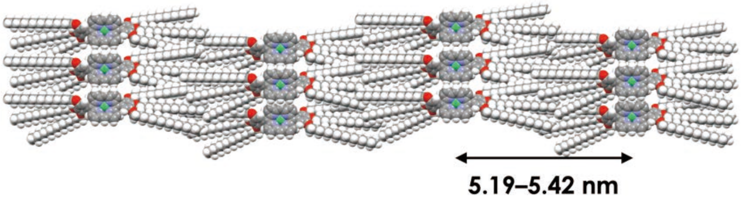

Fig. S66 Possible packing model of **2e** as a lamellar structure.

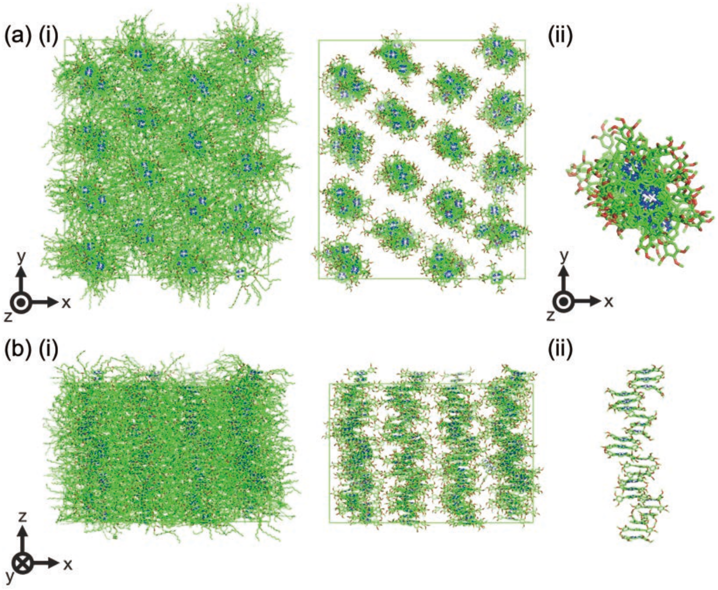

Fig. S67 (a) Top and (b) side views of MD simulation snapshots of **1d** as (i) whole structure and (ii) selected columnar structure after equilibration run at 30 °C with Col_h structure as an initial structure. Col_h structure was maintained during the equilibration. Alkyl chains in the right snapshots of (i) and in (ii) were replaced with methyl groups for clarity. The unit lattice constants after equilibration (a = 3.17 nm, c = 0.94 nm) were similar to those obtained by XRD measurements (a = 3.15 nm, c = 0.93 nm).

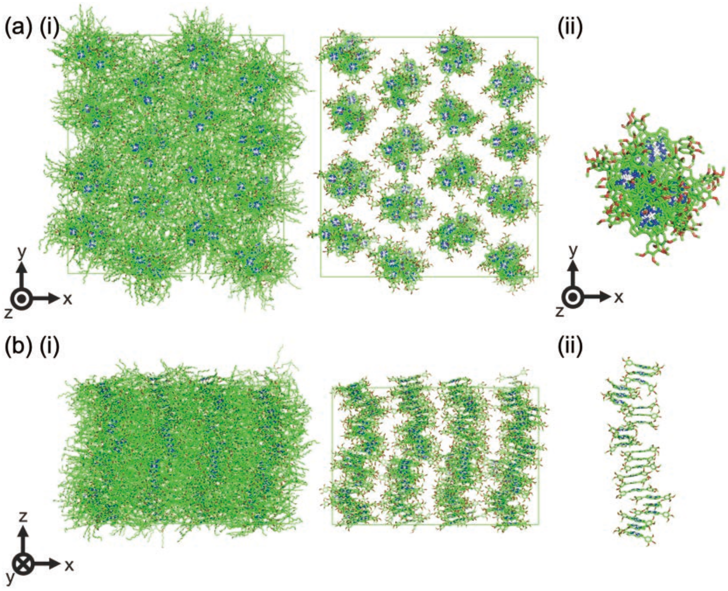

Fig. S68 (a) Top and (b) side views of MD simulation snapshots of **1d** as (i) whole structure and (ii) selected columnar structure after equilibration run at 50 °C with Col_r structure as an initial structure. Col_r structure was maintained during the equilibration. Alkyl chains in the right snapshots in (i) and in (ii) were replaced with methyl groups for clarity. The unit lattice constants after equilibration (a = 5.65 nm, b = 3.12 nm, c = 0.93 nm) were similar to those obtained by XRD measurements (a = 5.62 nm, b = 3.10 nm, c = 0.93 nm). The column packed in interdigitated arrangement, which can restrict the molecular motion and increase the activation barrier of the LC-LC transition.

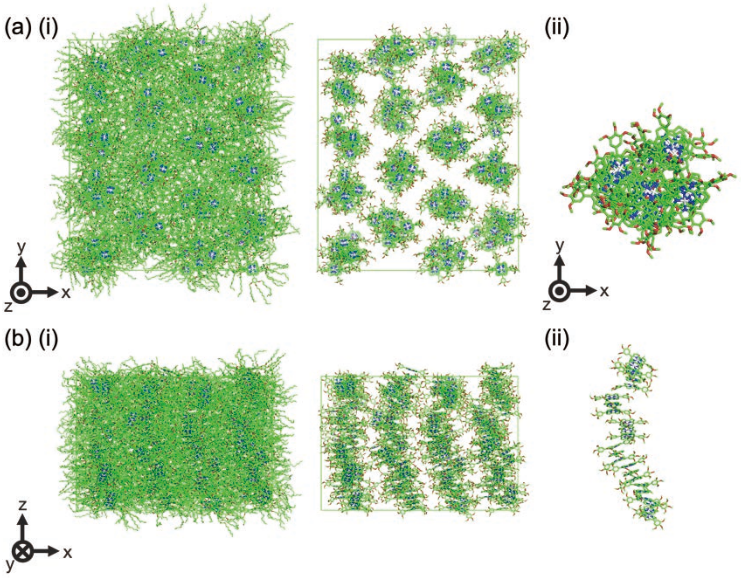

Fig. S69 (a) Top and (b) side views of MD simulation snapshots of **1d** as (i) whole structure and (ii) selected columnar structure after equilibration run at 60 °C with Col_h structure as an initial structure. Col_h structure was slightly collapsed after the equilibration. Alkyl chains in the right snapshots in (i) and in (ii) were replaced with methyl groups for clarity.

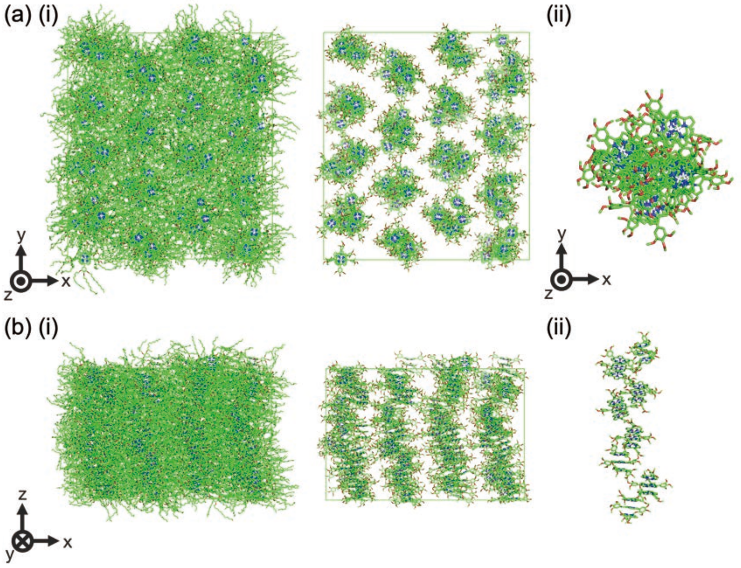

Fig. S70 (a) Top and (b) side views of MD simulation snapshots of **1d** as (i) whole structure and (ii) selected columnar structure after equilibration run at 60 °C with Col_r structure as an initial structure. Col_r structure was maintained during the equilibration. Alkyl chains in the right snapshots in (i) and in (ii) were replaced with methyl groups for clarity.

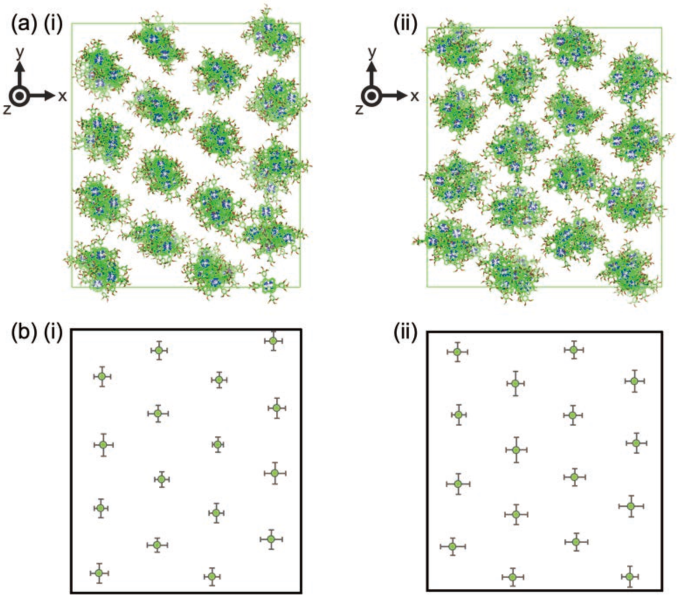

Fig. S71 (a) Top views of MD simulation snapshots of **1d** after equilibration run and (b) location of the columns (defined as the average of x and y coordinates of central Ni in the triple-decker structures in each column) with error bars: (i) Col_h at 30 °C and (ii) Col_r at 50 °C.

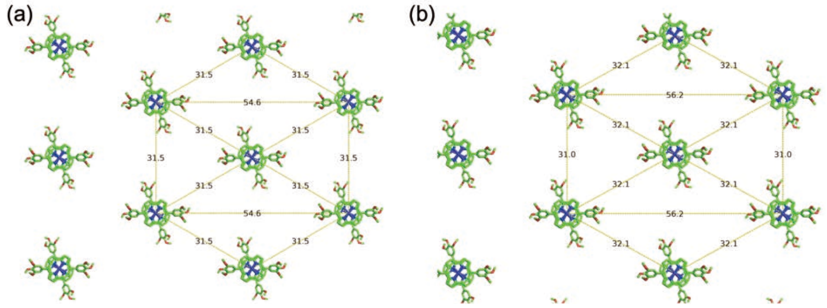

Fig. S72 Top views of initial structures of MD simulation of **1d** and intercolumnar distances: (a) Col_h and (b) Col_r.

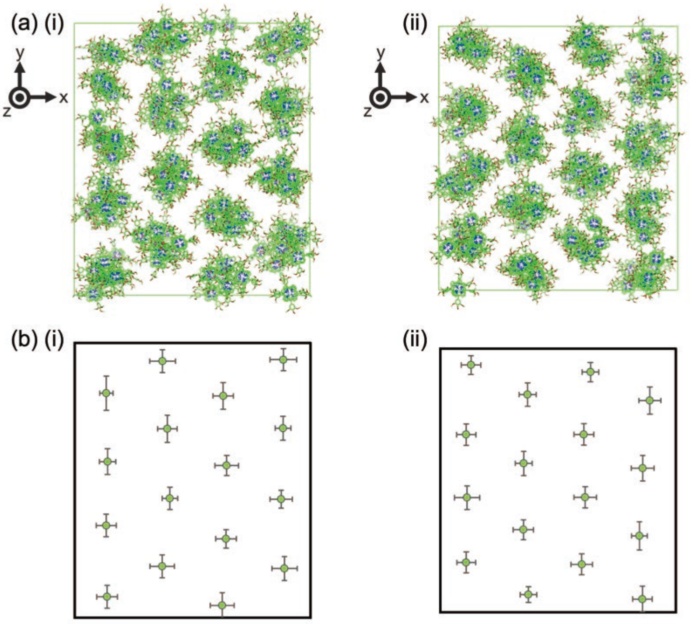

Fig. S73 (a) Top views of MD simulation snapshots of **1d** after equilibration run and (b) location of the columns (defined by the average of x and y coordinates of central Ni in the triple-decker structures in each column) with error bars: (i) Col_h at 60 °C and (ii) Col_r at 60 °C. Averages of the standard deviation along x and y axes in Col_h structure (4.89 and 5.50 Å, respectively) were larger than those in Col_r structure (4.74 and 5.30 Å, respectively), corresponding to the formation of Col_r structure at 60 °C in heating scan.

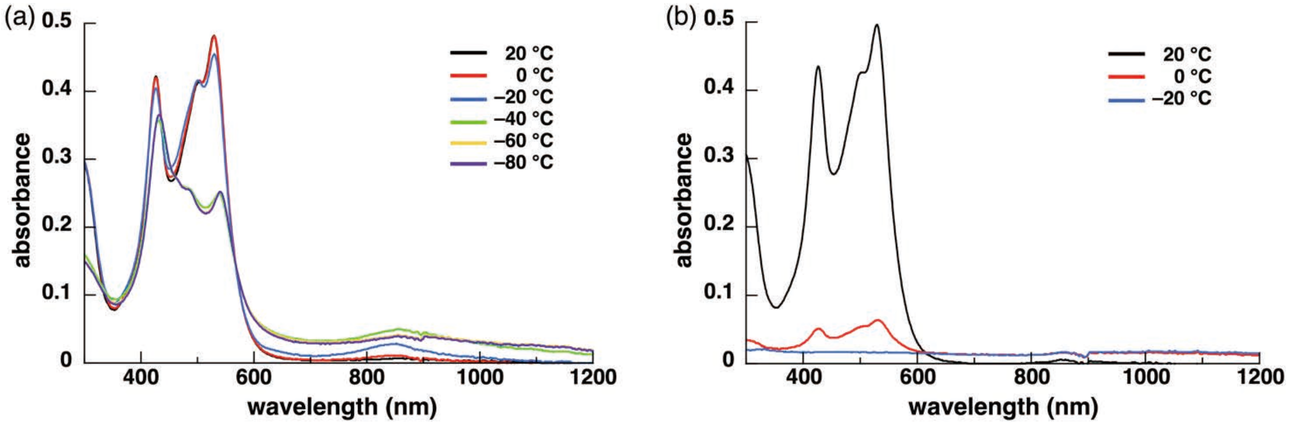

Fig. S74 VT-UV/vis absorption spectra in CH_2Cl_2 (1.0×10^{-3} M) of (a) **1d** at 20 °C (black), 0 °C (red), –20 °C (blue), –40 °C (green), –60 °C (yellow), and –80 °C (purple) and (b) **1e** at 20 °C (black), 0 °C (red), and –20 °C (blue). Appearance of the absorption band in the long wavelength region at low temperature corresponded to the results of TD-DFT calculation for stacked dimer and trimer (Fig. S43,44), suggesting the formation of stacked structures at low temperature in solution state.

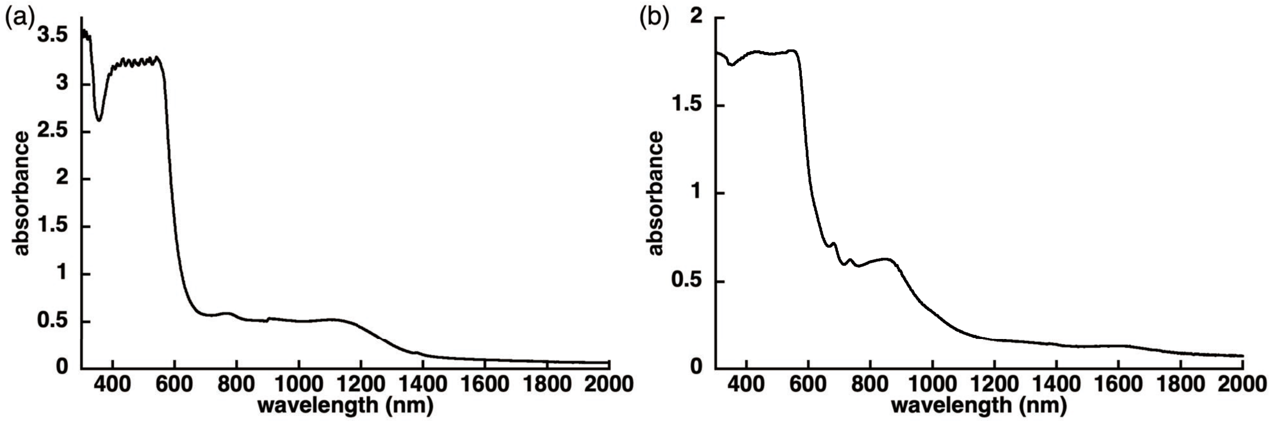

Fig. S75 UV/vis absorption spectra of (a) **1d** and (b) **1e** as film states at r.t. The **1d** film was prepared by sandwiching the powder sample with quartz plates, heating to 85 °C, and annealing at 35 °C for 52 h, whereas the **1e** film was prepared in a similar way, heating to 100 °C, annealing at 55 °C for 24 h. Previously reported stacked norcorrole dimers and aggregates exhibited the absorption in 800–900 nm region.[S7,10,15,29] The absorption band of the **1d** film was compared to that of the stacked dimer. According to TD-DFT calculation, the longest wavelength absorption band of the triple decker was more red-shifted than the double decker (Fig. S43,44), supporting the formation of the triple-decker structure of **1d** as the mesophase, whereas a weak absorption band at ~850 nm of the **1e** film implied the existence of the double decker structure as well as the monomer-like structure, which cannot be distinguished by XRD analysis.

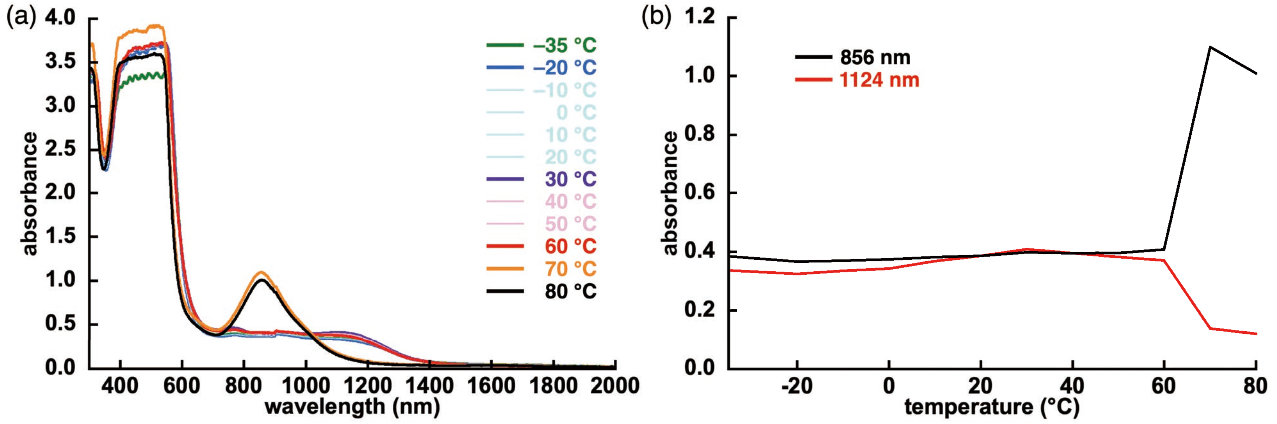

Fig. S76 (a) Variable-temperature UV/vis absorption spectra and (b) relationship between the absorbances at 856 nm (black) and 1124 nm (red) and temperature of **1d** as a film state.

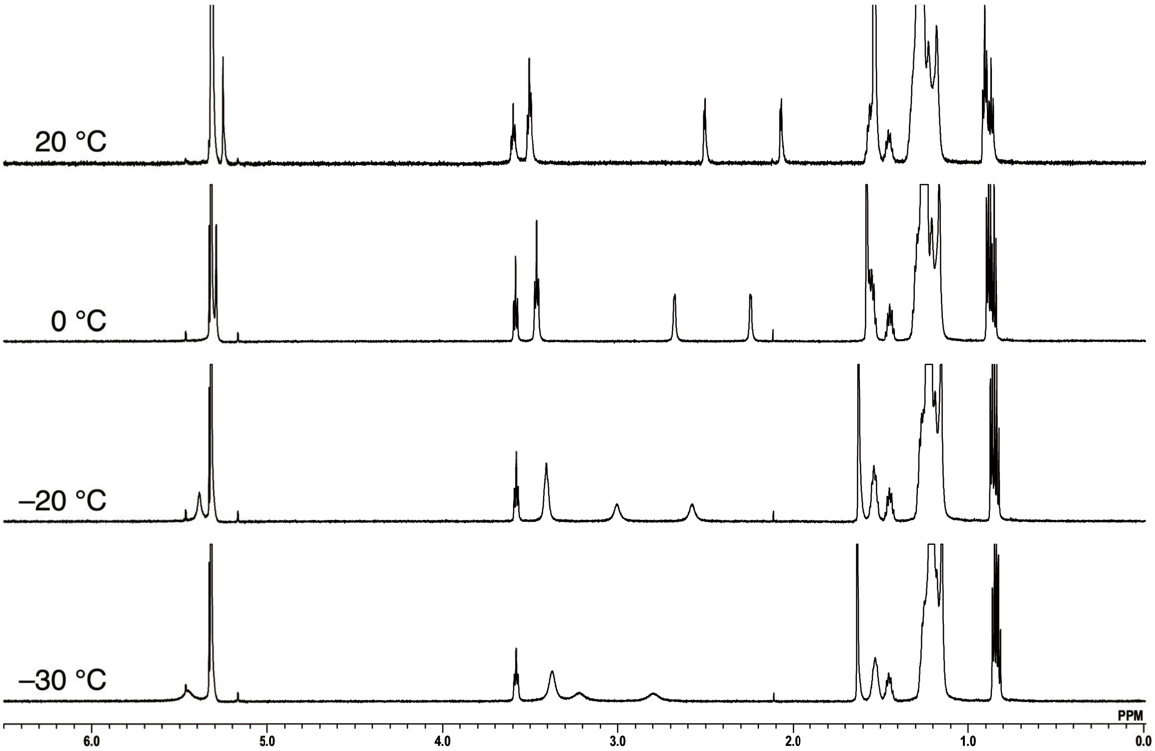

Fig. S77 VT ^1H NMR of **1d** in CD_2Cl_2 (1 mM) at 20– –30 °C. β-CH signals of the norcorrole core were shifted downfield at low temperature, ascribed to stacked-ring aromaticity.

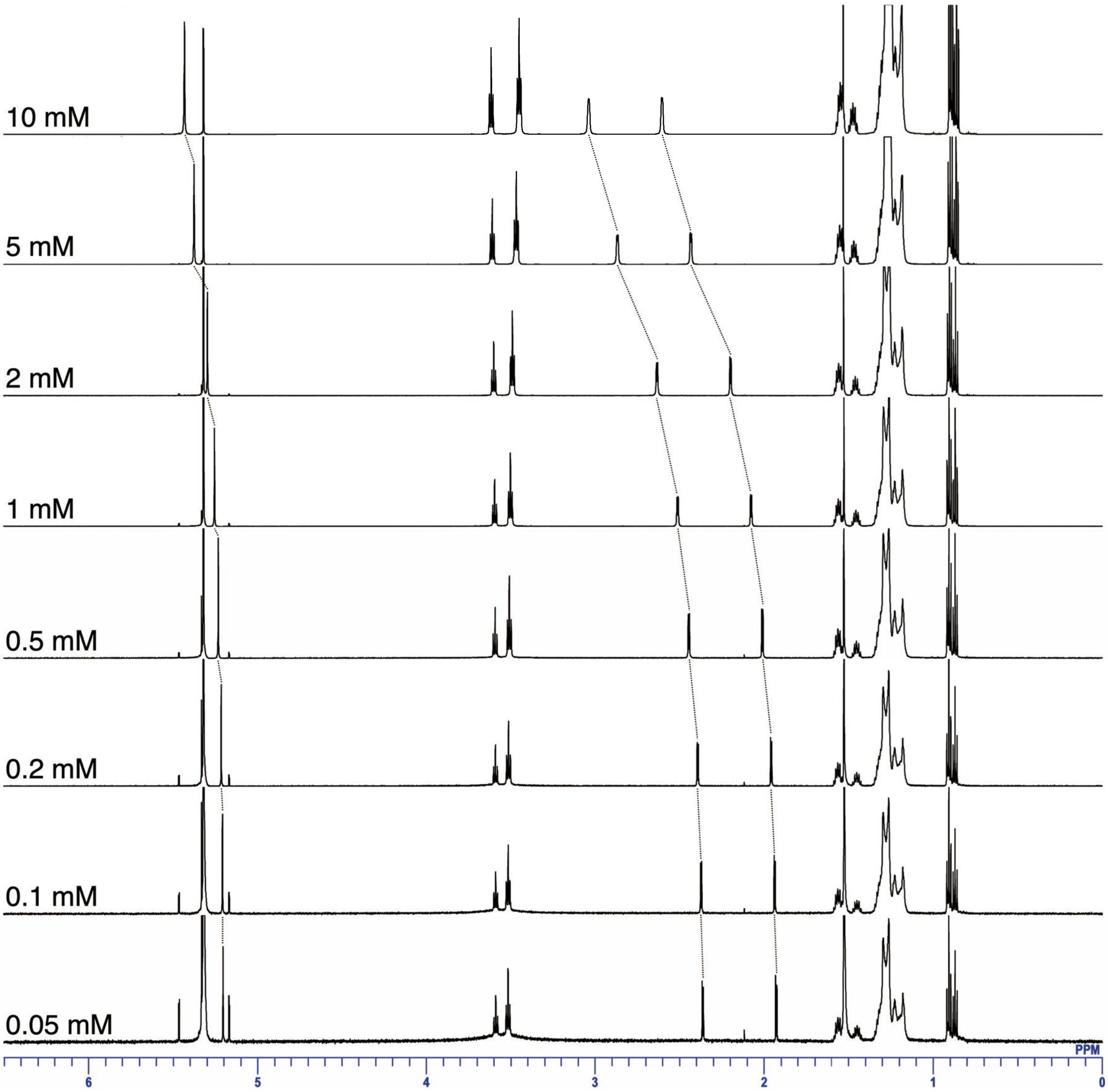

Fig. S78 ^{1}H NMR of **1d** in CD_2Cl_2 (0.05–10 mM) at 20 °C. The signals assigned to β-CHs were shifted downfield upon increasing concentrations. The change of chemical shifts enabled the estimation of the dimerization constant based on the equilibrium between monomer and dimer (Fig. S79).

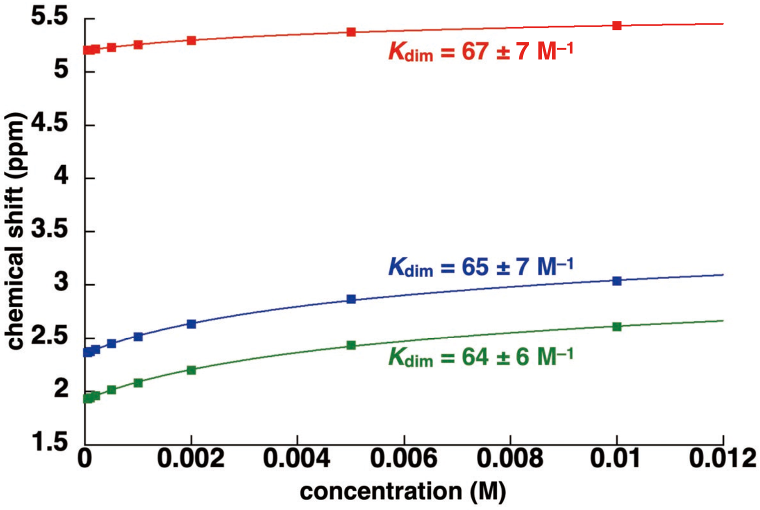

Fig. S79 Concentration-dependent plots of ^{1}H NMR chemical shifts and fitting curves of **1d** at 25 °C (red: *meso*-aryl CH; blue and green: β-CH) in CD_2Cl_2 (see also Fig. S78). Assuming that only isolated monomer and stacked dimer exist, dimerization constants were estimated by least-square curve fitting using the following formula

$$\delta_{obs} = \delta_{mono} + \frac{1 + 4K_{dim}C - \sqrt{1 + 8K_{dim}C}}{4K_{dim}C}(\delta_{dim} - \delta_{mono})$$

for the equilibrium between the monomer and the dimer showing their averaged signals (δ_{obs}: observed chemical shifts, δ_{mono}: ideal monomer chemical shift, δ_{dim}: ideal dimer chemical shift, K_{dim}: dimerization constant, C: total concentrations of **1d**). Fitting curves are consistent with the formation of the stacked dimer. The dimerization constants of **1d** were comparable to that of a previously reported norcorrole derivative (~110 M^{-1} in $CDCl_3$ as the value that was not described in the corresponding report but can be estimated based on the variable concentration ^{1}H NMR measurements).[S30,31]

[S21] J. Wang, R. M. Wolf, J. W. Caldwell, P. A. Kollman and D. A. Case, *J. Comput. Chem.*, 2004, **25**, 1157–1174.
[S22] J. Yoshida, S. Tamura, K. Hoshino, H. Yuge, H. Sato, A. Yamazaki, S. Yoneda and G. Watanabe, *J. Phys. Chem. B*, 2018, **122**, 10615–10626.
[S23] G. Watanabe, H. Watanabe, K. Suzuki, H. Yuge, S. Yoshida, T. Mandai, S. Yoneda, H. Sato, M. Hara and J. Yoshida, *Chem. Commun.*, 2020, **56**, 12134–12137.
[S24] C. I. Bayly, P. Cieplak, W. Cornell and P. A. Kollman, *J. Phys. Chem.*, 1993, **97**, 10269–10280.
[S25] H. J. C. Berendsen, J. P. M. Postma, W. F. van Gunsteren, A. Dinola and J. R. Haak, *J. Chem. Phys.*, 1984, **81**, 3684–3690.
[S26] S. Nosé, *Mol. Phys.*, 1983, **52**, 255–268.
[S27] M. Parrinello and A. Rahman, *J. Appl. Phys.*, 1981, **52**, 7182–7190.
[S28] B. Hess, H. Bekker, H. J. C. Berendsen and J. G. E. M. Fraaije, *J. Comput. Chem.*, 1997, **18**, 1463–1472.
[S29] S.-Y. Liu, N. Kishida, J. Kim, N. Fukui, R. Haruki, Y. Niwa, R. Kumai, D. Kim, M. Yoshizawa and H. Shinokubo, *J. Am. Chem. Soc.*, 2023, **145**, 2135–2141.
[S30] T. Yoshida, D. Sakamaki, S. Seki and H. Shinokubo, *Chem. Commun.*, 2017, **53**, 1112–1115.
[S31] A recent report of C_6F_5-substituted Ni^{II} norcorrole showing K_{dim} of 600 M^{-1} in $CDCl_3$: S. Kino, S. Ukai, N. Fukui, R. Haruki, R. Kumai, Q. Wang, S. Horike, Q. M. Phung, D. Sundholm and H. Shinokubo, *J. Am. Chem. Soc.*, 2024, **146**, 9311–9317.

5. Electric conductivity

Method for Time-Resolved Microwave Conductivity (TRMC) Measurements. **1d**,**e** in liquid crystals and **1a**,**b** in single crystals were placed onto quartz substrates (for quantitative analysis) and overcoated by Cytop®. The overcoated samples were dried and evacuated in vacuo at 60 °C for 1 h prior to the measurement. In addition, liquid crystals **1d**,**e** were heated to isotropic liquid states, held for 1 h, gradually cooled to r.t., rapidly cooled to –80 °C, held at –80 °C for 12 h, heated to 20 °C, and held at 20 °C for 1 h after the evacuation. The samples sandwiched by quartz plates were inserted into a TE-102 mode microwave cavity at Q-value of 2500 and were fixed at the position of electric field maximum. Time constant of the current set of flash photolysis (FP)-TRMC setup is $\tau = Q/2f \approx 100$ ns, where the films over quartz were loaded at the point of the electric field maximum. Excitation of the crystals was carried out through the quartz at 355 nm or 532 nm by 3rd or 2nd harmonic generation (THG or SHG) from a Spectra-Physics INDI Nd:YAG laser. The excitation light intensity through the sample and quartz was monitored by an Ophir VEGA power meter with a PE-25 head. Photoconductivity transients, demodulated through a GaAs crystal diode with Schottky barriers (rise time < 1 ns), were monitored by a Tektronix model TDS3054 digital oscilloscope. Inside of the cavity was filled with dry N_2, and the measurements were performed at 20–127 °C. For photo-current measurements a 0.5-mm thick glass plate was cleaned by distilled water and isopropyl alcohol for 10 min each. The dried substrates were exposed to UV/ozone for 5 min. After casting the sample, an electrode was placed in a vacuum prober and exposed to THG (355 nm) or SHG (532 nm) of an identical Nd:YAG laser to FP-TRMC measurements. The applied bias was controlled by an Advantest Corp. model R8252 digital electrometer. The transient photocurrent was measured by a Tektronix model TDS3052B digital oscilloscope equipped with termination resistance (10 kΩ).

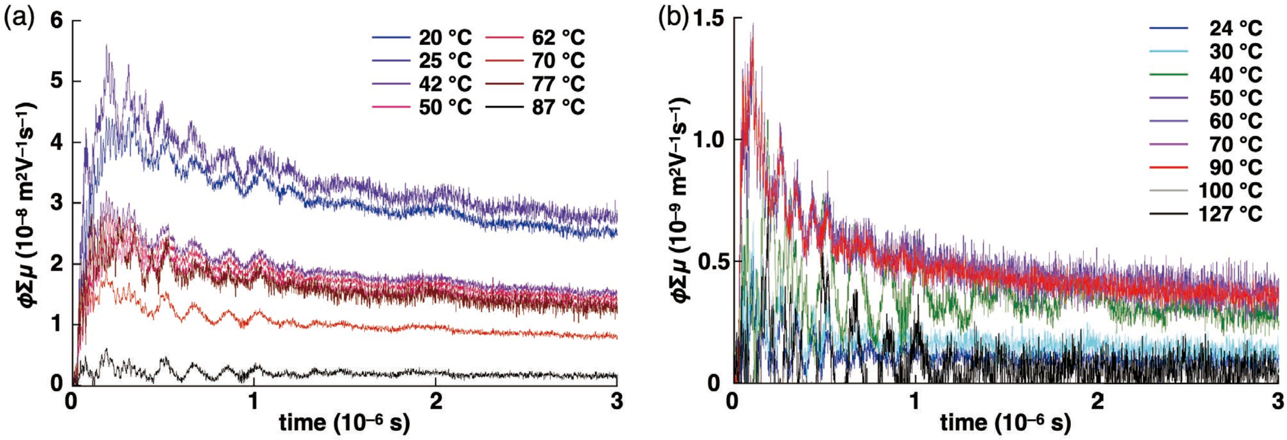

Fig. S80 FP-TRMC photoconductivity transients recorded for an (a) **1d** and (b) **1e** upon excitation at 355 nm, 2.7×10^{15} photons cm^{-2}. The liquid crystalline samples were held for more than 30 min at each temperature before the measurements. The $\varphi\Sigma\mu$ values of **1d**,**e** were suddenly decreased upon phase transitions to the isotropic liquid states. In addition, the $\varphi\Sigma\mu$ value of **1d** (5.2×10^{-8} $m^2V^{-1}s^{-1}$ at 25 °C) was notably decreased upon cooling from 25 °C to 42 °C, whereas that of **1e** was increased upon heating from 30 °C to 50 °C (1.1×10^{-9} $m^2V^{-1}s^{-1}$ at 50 °C), corresponding to the phase transition temperature between two lamellar phases.

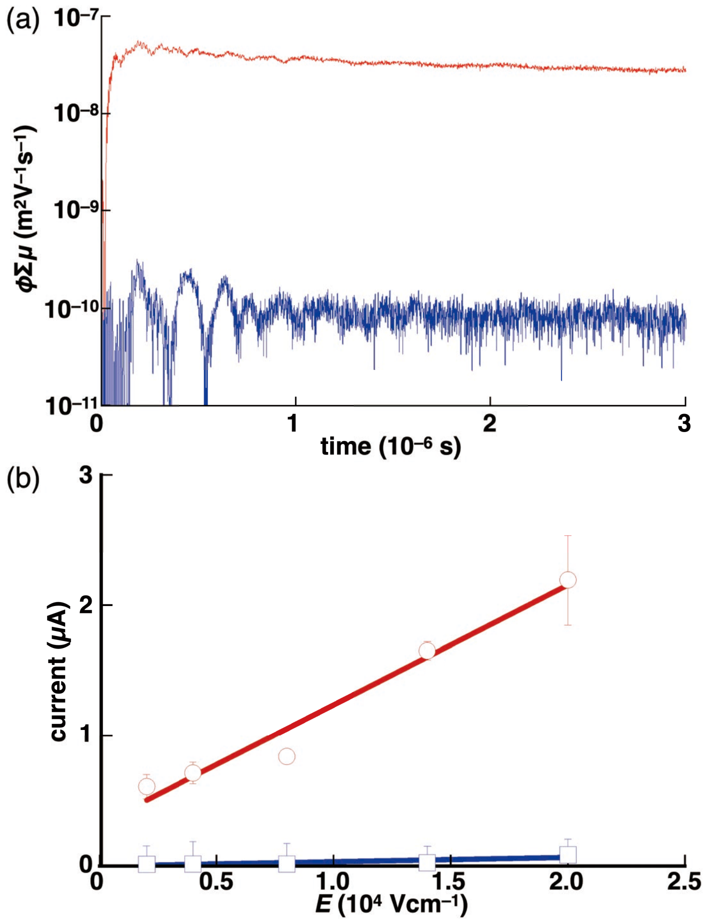

Fig. S81 (a) Transient photoconductivity observed for **1d** upon excitation at 355 nm (red) and 532 nm (blue) at 25 °C and (b) electric bias dependence of maximum photocurrent on recorded upon excitation at 355 nm (red) and 532 nm (blue) to **1d** thin film deposited onto an interdigitated comb-type electrode with 5-μm gap at 293 K. $\varphi\Sigma\mu$ value of **1d** obtained by photoexcitation at 355 nm was much higher than that excited at 532 nm. The result indicated that carriers were hardly generated due to the insufficient energy obtained by the excitation at 532 nm for charge separation. Error bar in (b) is derived from min-max signals of current recorded.
